# Electronic Patient-Reported Outcome Monitoring With Alert-Based Interventions in Metastatic Breast Cancer: A Randomized Clinical Trial

**DOI:** 10.1001/jamaoncol.2026.2661

**Published:** 2026-08-06

**Authors:** Maria M. Karsten, Pimrapat Gebert, Therese Pross, Anna M. Hage, Adam D. Dordevic, Susan Stephan, Friedrich Kühn, Lea Doppelbauer, Jens-Uwe Blohmer, Felix Fischer, Matthias Rose, Anna Pöhlmann, Bianca Materne, Morten Aagaard Petersen, Luis Pauler, Julia Ferencz, Simone Wesselmann, Clara Breidenbach, Ulrike Grittner, Christoph Kowalski

**Affiliations:** 1 Department of Gynecology with Breast Center, Charité–Universitätsmedizin Berlin, Berlin, Germany; 2 Berlin Institute of Health at Charité– Universitätsmedizin Berlin, Berlin, Germany; 3 Institute of Biometry and Clinical Epidemiology, Charité–Universitätsmedizin Berlin, Berlin, Germany; 4 Department of Psychosomatic Medicine and Psychotherapy, Theodor-Wenzel-Werk e.V., Berlin, Germany; 5 Center for Patient-Centered Outcomes Research, Department for Psychosomatic Medicine, Charité–Universitätsmedizin Berlin, Berlin, Germany; 6 Palliative Care Research Unit, Department of Geriatrics and Palliative Medicine GP, Bispebjerg and Frederiksberg Hospital, Copenhagen, Denmark; 7 OnkoZert, Neu-Ulm, Germany; 8 Deutsche Gesellschaft für Allgemein und Viszeralchirurgie e.V., Berlin, Germany; 9 German Cancer Society, Berlin, Germany

## Abstract

**Importance:**

Electronic patient-reported outcome (PRO) monitoring has shown benefits in advanced cancer, but data specific to metastatic breast cancer (mBC) remain limited. Fatigue is a common, clinically important, and functionally impairing symptom in mBC, making it a patient-centered target for electronic PRO monitoring.

**Objective:**

To determine whether digital PRO monitoring with alert-based responses reduces fatigue in patients with mBC.

**Design, Setting, and Participants:**

This multicenter randomized clinical trial was conducted from May 2021 to February 2024, with 12 months of follow-up, at 52 breast cancer centers in Germany and included women aged 18 years or older with mBC who were receiving systemic therapy at any point in the metastatic disease course, had a life expectancy of longer than 3 months, were able to read German, and had smartphone access. Data analysis started in February 2024 and lasted 6 months.

**Interventions:**

The intervention group completed weekly PRO questionnaires via a smartphone using validated short forms from the European Organization for Research and Treatment of Cancer computerized adaptive testing core item bank. Automated alerts based on predefined PRO deterioration were emailed to trained local nurses or physicians, who contacted patients by telephone within 48 hours. The control group received usual care with quarterly PRO questionnaires and no alerts.

**Main Outcomes and Measures:**

The primary outcome was fatigue at 6 months. Secondary outcomes included fatigue, physical functioning, and quality of life over 12 months. PROs were reported as standardized T scores.

**Results:**

Of 2008 patients screened, 924 (46.0%) were randomized, and 909 (45.3%) were included in the primary analysis (median age, 50 years [range, 19-83 years]). Baseline mean (SD) fatigue T scores were 57.6 (9.5) in the intervention group and 60.2 (9.0) in the control group (*P* < .001), with baseline PROs assessed after randomization but before group-specific follow-up began. At 6 months, adjusted mean fatigue was 54.5 (95% CI, 53.7- 55.4) vs 59.9 (95% CI, 59.0-60.8), yielding a mean difference of −5.4 points (95% CI, −6.6 to −4.1; *P* < .001), exceeding the minimal clinically important difference (MCID) of 3.3 points. At 6 months, physical functioning was higher in the intervention group (mean difference, 4.0; 95% CI, 2.6-5.4), exceeding its MCID (3.2), whereas the between-group difference in quality of life was 0.8 points (95% CI, 0.02-1.5), which was less than the MCID of 2.9.

**Conclusions and Relevance:**

This randomized clinical trial found that digital PRO monitoring was associated with clinically meaningful reductions in fatigue and improved physical functioning. These findings support further evaluation of alert-based PRO monitoring in routine oncology care.

**Trial Registration:**

German Clinical Trials Register: DRKS00024015

## Key Points

**Question:**

Does alert-based patient-reported outcome (PRO) monitoring reduce fatigue in patients with metastatic breast cancer (mBC)?

**Findings:**

In this randomized clinical trial of 909 patients with mBC, 6-month fatigue was significantly lower with alert-based PRO monitoring by 5.4 T-score points, exceeding the minimal clinically important difference threshold.

**Meaning:**

The trial results suggest that alert-based PRO monitoring may improve fatigue among patients with mBC, although differential follow-up and postrandomization baseline assessments warrant cautious interpretation.

## Teaser

This randomized clinical trial examines the relationship between digital patient-reported outcome monitoring with alert-based responses and fatigue in patients with metastatic breast cancer.

## Introduction

Systematic electronic patient-reported outcome (ePRO) monitoring has emerged as a strategy to improve symptom management and health-related quality of life (HRQoL) in patients with cancer.^1,2,3^ Patients complete standardized symptom and functioning questionnaires at regular intervals, and clinically important deterioration can trigger clinical review. Growing evidence has suggested that digital patient-reported outcome (PRO) monitoring in advanced cancer improves symptom control^1^ and may prolong survival.^4,5^ However, early studies were largely conducted in research-intensive centers,^4,5,6^ and more recent multicenter trials have evaluated ePRO monitoring in broader settings^1,2,3,5,6,7,8,9^; disease-specific multicenter evidence remains limited.

Metastatic breast cancer (mBC) remains incurable,^10^ and treatment aims to prolong survival while maintaining daily functioning and HRQoL. Patients with mBC may receive prolonged, sequential systemic therapies across the metastatic disease course, during which disease burden and treatment-related toxic effects can accumulate.^10,11,12,13^ As a result, patients frequently experience worsening symptoms that may be underrecognized in routine care and negatively affect HRQoL.^12,13,14^ Therefore, a disease-specific ePRO study was warranted to evaluate whether structured symptom monitoring can support care in this clinical setting.

Fatigue is a persistent and distressing sense of physical, emotional, and cognitive exhaustion that interferes with daily functioning.^15^ In patients with mBC, fatigue is among the most common and debilitating symptoms,^16,17,18^ often worsening during systemic treatment and affecting physical activity, work, social roles, and HRQoL.^16,19^ Because fatigue is highly prevalent, clinically meaningful, and potentially responsive to earlier symptom detection, we selected it as the primary outcome.^1,4,7,13,15,16,17^

The PRO in Breast Cancer (PRO B) trial was designed to evaluate alert-based digital PRO monitoring in patients with mBC. We hypothesized that weekly ePRO monitoring with automated alerts would reduce fatigue at 6 months and improve longitudinal symptom and functioning outcomes vs usual care. Because earlier symptom detection may reduce unplanned health care utilization, hospitalization and emergency department (ED) visits were evaluated as secondary outcomes in a claims data subpopulation, as claims data were unavailable for all patients. The findings of this trial may inform future disease-specific ePRO monitoring to support symptom-centered care for patients with mBC.

## Methods

### Study Design and Participants

The PRO B Trial was an investigator-initiated, open-label, randomized clinical trial that was conducted across 52 certified academic and community breast cancer centers in Germany (the trial protocol and statistical analysis plan is available in Supplement 1). Eligible patients were female with mBC, 18 years or older, were receiving systemic therapy, had a life expectancy longer than 3 months, had a clinician-rated Eastern Cooperative Oncology Group (ECOG) performance status^20^ of 0 to 2, were able to read and understand German, and had access to a smartphone. Patient-reported performance status was collected at baseline using standard ECOG category descriptions.^21^ Patients could be enrolled at any time during the metastatic disease course, before or during systemic therapy.

After providing written informed consent, patients downloaded a smartphone application (PatientConcept; NeuroSys GmbH), were randomly assigned using stratified allocation as described later in the article and completed the baseline assessment within 7 days after randomization (the intervention group completed within 2 days for weekly PRO initiation). The study was approved by the ethics committee of Charité–Universitätsmedizin Berlin (EA1/318/20). The trial was prospectively registered with the German Clinical Trials Register (DRKS00024015). The trial was according to the Consolidated Standards of Reporting Trials (CONSORT)29 and CONSORT-PRO guidelines.^22,23^ Additional methodological details have been reported previously.^24,25^

### Randomization

Patients were randomly assigned 1:1 and stratified by (1) metastatic site distribution (nonvisceral: bone, skin, or lymph node; single-organ visceral; multiorgan and/or brain metastases), (2) histological subtype (hormone receptor [HR]–positive [HR-positive/*ERBB2*-negative or HR-positive/*ERBB2*-positive] vs HR-negative [HR-negative/*ERBB2*-positive or HR-negative/*ERBB2*-negative]), and (3) study center. Adaptive randomization was implemented via secuTrial (interActive Systems Berlin).

### Study Instruments and Data Collection

#### PRO Measures

PROs were assessed using validated short forms from the European Organization for Research and Treatment of Cancer (EORTC) Quality of Life Group Computerized Adaptive Testing (CAT) core item bank,^26^ covering QLQ-C30 (5 functional and 9 symptom domains). Scores were reported as standardized T scores (mean [SD], 50 [10]) for the European general population.^27^ Higher T scores indicated better functioning for functional scales and greater symptom burden for symptom scales.

#### Intervention Group

Patients completed weekly PRO questionnaires of 51 to 57 items depending on the assessment week (eTable 1 in Supplement 2). Questionnaires were delivered on a fixed weekday that corresponded to the enrollment (Monday to Friday), with automated reminders until completion for up to 2 days. Alerts were triggered by clinically meaningful deterioration relative to the prior assessments or worsening on a single health transition item (eTable 2 in Supplement 2). Following a review of alerts generated during early implementation, the alert criteria were revised on September 1, 2022, to increase alert sensitivity and improve clinical usability; at that point, 600 patients had been enrolled (299 control [49.8%]; 301 intervention [50.2%]). All models were adjusted for the enrollment period (before vs after September 1, 2022). Automated alerts were emailed to local nurses or physicians trained on the PRO B workflow, who contacted patients by telephone within 48 hours and documented actions taken. PRO data were available to the study centers via a web portal.

#### Control Group

Patients received usual care and completed the full 57-item PRO questionnaire every 3 months via the same application. No alerts were generated, and PRO data were not visible to the patients or care team. Additional details are provided in the eMethods in Supplement 2.

### Outcomes

After an early protocol amendment on July 19, 2021, when 25 patients had been enrolled (13 control; 12 intervention), the primary outcome was changed from 12-month to 6-month fatigue because substantial 12-month attrition was anticipated. Secondary outcomes included fatigue, physical functioning (PF), and HRQoL over 12 months, as well as health care utilization, which was defined as claims-derived hospitalization and ED visits at 6 and 12 months in patients insured by 3 participating statutory health insurance companies. Exploratory clinical outcomes were time to first change in systemic therapy and overall survival (OS) at 12 months in protocol-defined subgroups. OS in the full cohort was analyzed as an additional exploratory outcome. All PROs were assessed using the CAT Core short forms and reported as T scores.

### Statistical Analysis

A sample size of 400 patients per group was estimated to detect a 5-point difference in QLQ-C30 fatigue scores (0-100 scale), assuming an SD of 25 points (Cohen *d*, 0.2),^28^ with 80.7% power and a 2-sided significance level of .05. With 20% dropout, the target enrollment was 500 patients per group (N = 1000 in total); recruitment stopped early because of funding constraints.

The trial analyses used T scores, for which no established minimally clinically important difference (MCID) thresholds were available at the time of study design; therefore, the sample size calculation was based on the QLQ-C30.

For interpretation of trial results, we used published MCIDs for advanced breast cancer that were derived for the QLQ-C30^29^ and translated them to the T-score metric, which assesses the same domains on a different standardized scale. On this basis, a fatigue difference of 3.3 T-score points (approximately 8 points on the QLQ-C30) was considered clinically important. Corresponding thresholds were 3.2 T-score points for PF and 2.9 for HRQoL; no established MCID was available for emotional functioning (EF).^29^

Analyses were conducted in the modified intent-to-treat population, including all participants who completed at least 1 postbaseline PRO assessment. Recruitment was extended from February 15, 2023, to June 15, 2023; therefore, 156 patients (78 per group) enrolled during this extension period could not complete the 9-month and 12-month assessments before the administrative study end date of February 15, 2024. These patients contributed observed data until administrative censoring. Assessments precluded by administrative censoring were considered structurally unobservable, not missing, and were not imputed.

For the primary outcome, fatigue at 6 months was compared between groups using a linear mixed-effects model with a random intercept for the center, which was adjusted for baseline fatigue and the same covariates used in the longitudinal models. The primary outcome was tested at a 2-sided significance level of .05. Planned exploratory subgroup analyses tested intervention-by-subgroup interaction terms.

Longitudinal PRO outcomes were analyzed using linear mixed-effects models, with random intercepts for study centers and patients.^29,30^ Time was modeled as a discrete variable, and estimated marginal means with 95% CIs were reported at 3, 6, 9, and 12 months. Models were adjusted for baseline PRO score, stratification factors, age, ECOG status, systemic therapy, sociodemographic factors, comorbidity index scores, depression, and enrollment period. Sidak adjustment was applied across points. Missing data were addressed using multiple imputation with chained equations (eMethods and eTables 3-5 in Supplement 2).

Time to first change in systemic therapy and OS were assessed using a Kaplan-Meier analysis and Cox proportional hazards models, with shared frailty for center. Because death before treatment change was uncommon within 12 months (23 [7.5%]), competing risk modeling was not pursued.^24^

Health care utilization outcomes were analyzed as incidence rates per 100 person-months; incidence rate ratios (IRRs) were estimated using a multilevel negative binomial regression that was adjusted for baseline clinical covariates (eMethods in Supplement 2). Analyses were conducted using Stata MP/18 (StataCorp) and R, version 4.2.2 (R Foundation).

## Results

**Figure 1.**
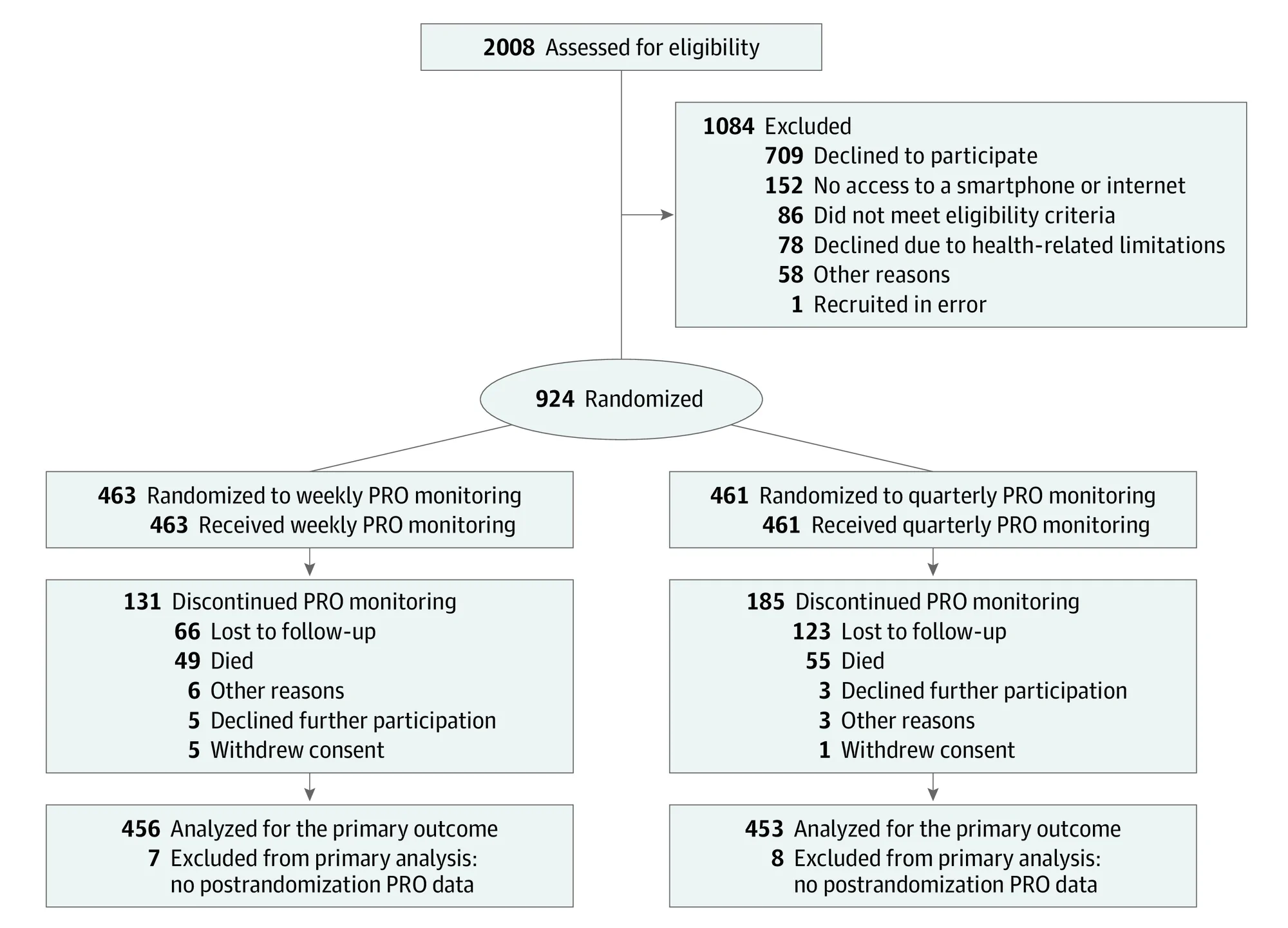
Flowchart of Trial Profile PRO indicates patient-reported outcome.

Between May 2021 and June 2023, 2008 patients, who were all women, were screened. In total, 924 (46.0%) were randomized (463 intervention [50.1%]; 461 control [49.9%]), and 909 patients (45.3%) were included in the primary analysis (456 intervention [50.2%]; 453 control [49.8%]); 15 were excluded because they did not complete any questionnaires (Figure 1). Follow-up completion among eligible patients was 761 of 846 (90.0%) at 6 months and 521 of 695 (75.0%) at 12 months. The mean (SD) age was 50.6 (10.7) years (range, 19-81 years) in the intervention group and 51.0 (11.2) years (range, 25-83 years) in the control group. HR-positive/*ERBB2*-negative disease was present in 556 patients (66.2%), and 378 (41.8%) had de novo metastatic presentation. Baseline characteristics were balanced, although baseline PRO scores favored the intervention group in most domains (Table).

**Table.** Sociodemographic Data and Clinical Baseline Characteristics

Characteristic	No. (%)		
	Total (N = 909)	Control group (n = 453)	Intervention group (n = 456)
Age at randomization, mean (SD) [range], y	50.8 (11.0) [19-83]	51.0 (11.2) [25-83]	50.6 (10.7) [19-81]
Observed sociodemographic data and patient history	890 (97.9)	444 (98.0)	446 (97.8)
German nationality	850 (95.5)	422 (95.0)	428 (96.0)
Family status			
Married/living together	581 (65.3)	295 (66.4)	286 (64.1)
Married/living separately	25 (2.8)	8 (1.8)	17 (3.8)
Single	118 (13.3)	49 (11.0)	69 (15.5)
Divorced	107 (12.0)	56 (12.6)	51 (11.4)
Widowed	59 (6.6)	36 (8.1)	23 (5.2)
With children <14 y	148 (16.6)	75 (16.9)	73 (16.4)
Education level			
Low	85 (9.6)	42 (9.5)	43 (9.6)
Intermediate	486 (54.6)	257 (57.9)	229 (51.3)
High	319 (35.8)	145 (32.7)	174 (39.0)
Self-reported ECOG status			
0	178 (20.0)	82 (18.5)	96 (21.5)
1	496 (55.7)	256 (57.8)	240 (53.7)
2	169 (19.0)	83 (18.7)	86 (19.2)
3	45 (5.1)	20 (4.5)	25 (5.6)
4	2 (0.2)	2 (0.5)	0 (0.0)
NCI comorbidity index score, mean (SD)	0.1 (0.3)	0.1 (0.3)	0.1 (0.2)
Clinical baseline characteristics^a^			
Time since metastatic diagnosis, mo			
Median (IQR)	17 (4-43)	15 (4-44)	18 (4-43)
Missing, No.	8	4	4
Clinical subtype			
HR-positive/*ERBB2*-positive	141 (16.8)	73 (17.6)	68 (16.0)
HR-positive/*ERBB2*-negative	556 (66.2)	269 (64.7)	287 (67.7)
HR-negative/*ERBB2*-positive	56 (6.7)	25 (6.0)	31 (7.3)
TNBC	87 (10.4)	49 (11.8)	38 (9.0)
Missing, No.	69	37	32
Site of metastases			
Brain or multiple metastases	503 (55.3)	250 (55.2)	253 (55.5)
Bone, lymph nodes, or skin	272 (29.9)	135 (29.8)	137 (30.0)
Visceral (only 1 organ)	134 (14.7)	68 (15.0)	66 (14.5)
Metastatic presentation^b^			
De novo	378 (41.8)	181 (40.1)	197 (43.5)
Recurrent	526 (58.2)	270 (59.9)	256 (56.5)
Unknown	5 (0.6)	2 (0.4)	3 (0.7)
Systemic therapy at baseline			
Endocrine	536 (59.0)	262 (57.8)	274 (60.1)
Chemotherapy	442 (48.6)	219 (48.3)	223 (48.9)
Anti-*ERBB2*	209 (23.0)	108 (23.8)	101 (22.1)
Another immunotherapy	32 (3.5)	16 (3.5)	16 (3.5)
Targeted therapy	317 (34.9)	150 (33.1)	167 (36.6)
Other	123 (13.5)	63 (13.9)	60 (13.2)
Observed baseline PRO data	855 (94.1)	425 (93.8)	430 (94.3)
Health transition item at baseline			
Much better	56 (6.5%)	28 (6.6%)	28 (6.5%)
A little better	187 (21.9%)	87 (20.5%)	100 (23.3%)
About the same	517 (60.5%)	261 (61.4%)	256 (59.5%)
A little worse	90 (10.5%)	45 (10.6%)	45 (10.5%)
Much worse	5 (0.6%)	4 (0.9%)	1 (0.2%)
EORTC T score, mean (SD)			
Global health status/QoL	35.9 (3.9)	36.0 (4.3)	35.9 (3.6)
Physical functioning	41.2 (9.4)	41.0 (9.1)	41.4 (9.7)
Role functioning	40.3 (10.2)	39.7 (9.9)	40.8 (10.5)
Emotional functioning^b^	41.3 (8.8)	40.4 (8.4)	42.3 (9.1)
Cognitive functioning^b^	43.7 (10.0)	42.5 (9.9)	44.8 (10.0)
Social functioning^b^	41.3 (8.9)	40.2 (8.0)	42.4 (9.5)
Fatigue^b^	58.8 (9.4)	60.2 (9.0)	57.5 (9.5)
Nausea and vomiting^b^	59.3 (10.6)	61.3 (11.8)	57.4 (9.0)
Pain^b^	54.8 (10.0)	55.7 (10.2)	53.9 (9.6)
Dyspnea^b^	58.4 (10.6)	60.0 (10.1)	56.7 (10.8)
Insomnia^b^	57.4 (8.6)	58.7 (8.6)	56.2 (8.5)
Appetite loss^b^	55.0 (11.6)	56.9 (12.1)	53.1 (10.9)
Constipation^b^	53.0 (10.9)	54.3 (11.3)	51.7 (10.4)
Diarrhea^b^	55.3 (11.7)	57.3 (12.2)	53.4 (10.9)
Financial difficulties^b^	54.0 (10.5)	55.9 (10.9)	52.1 (9.8)

Abbreviations: ECOG, Eastern Cooperative Oncology Group (classification); EORTC, European Organization for Research and Treatment of Cancer; HR, hormone receptor; NCI, National Cancer Institute; PRO, patient-reported outcome; QoL, quality of life; TNBC, triple-negative breast cancer.

^a^ Classification based on timing of first documented distant metastasis relative to initial diagnosis (≤90 days = de novo; >90 days = recurrent).

^b^ Statistically significant between-group difference at baseline. Tests were performed only for baseline PRO scores because these assessments were completed after randomization assignment.

### Questionnaire Return Rate, Alerts, and PRO B Interventions

**Figure 2.**
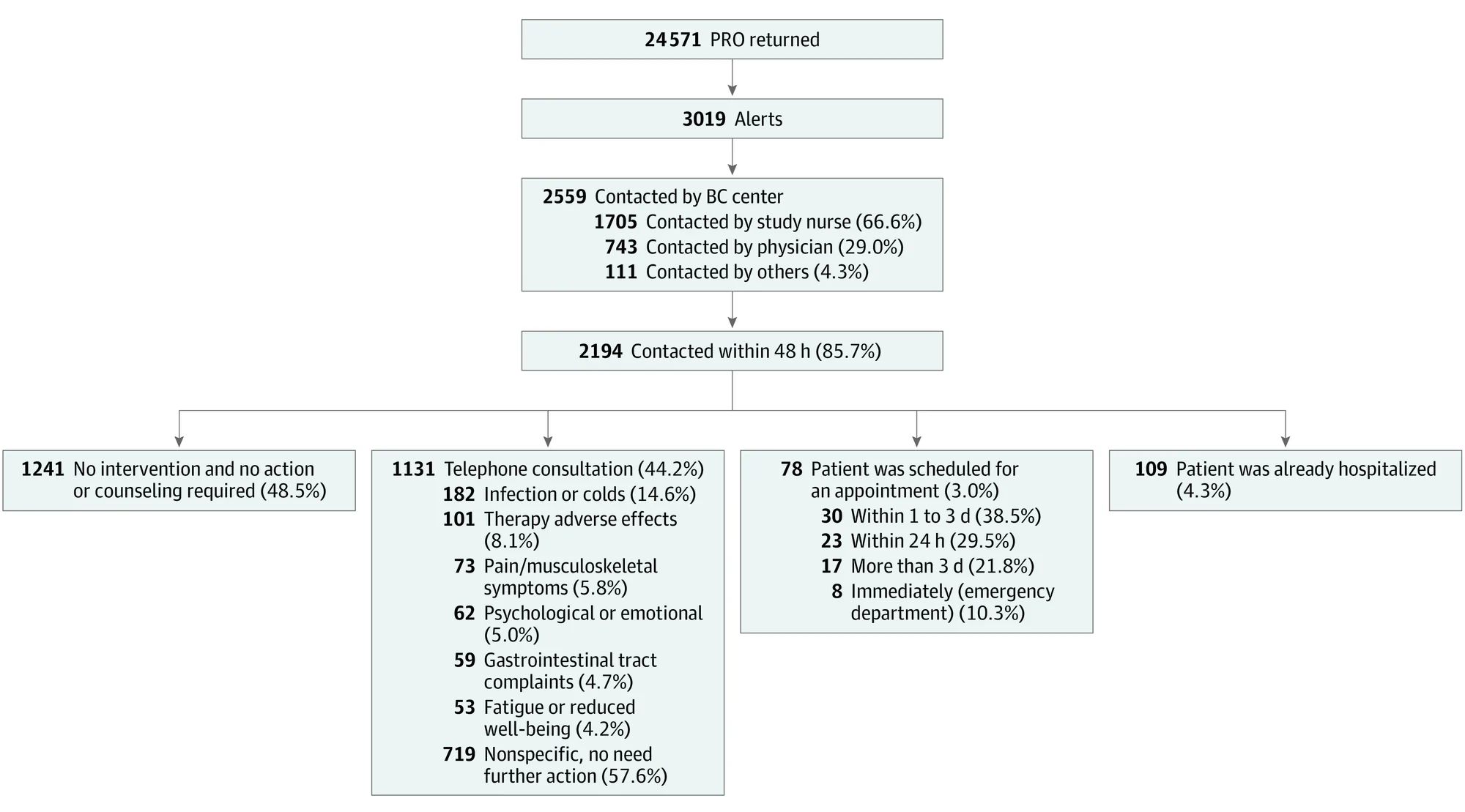
Flowchart of Overview of Patient-Reported Outcome (PRO) Responses, Alert Generation, and PRO B Interventions BC indicates breast cancer.

Median follow-up was 64 weeks (IQR, 37-100) in the intervention group and 52 weeks (IQR, 26-91) in the control group. Overall, 39 817 questionnaires were sent (36 845 intervention [92.5%]; 2972 control [7.5%]). Return rates at 6 and 12 months were 89% and 90% in the intervention group vs 74% and 73% in the control group, respectively (eFigure 1 in Supplement 2). Among 24 180 follow-up assessments in the intervention group, 3019 alerts were generated (12.5%; median of 5 per patient [IQR, 2-9]). Alerts were more frequently triggered by declines in social, role, emotional, and PF; fatigue accounted for 20.6% (eFigure 2 in Supplement 2). Of all alerts, 2559 led to patient contact, 2194 (85.7%) were within 48 hours; 1241 (48.5%) required no intervention, 1131 (44.2%) were resolved by telephone consultation, and 109 (4.3%) occurred while the patient was already hospitalized (Figure 2).

### Primary Outcome: Fatigue at 6 Months

**Figure 3.**
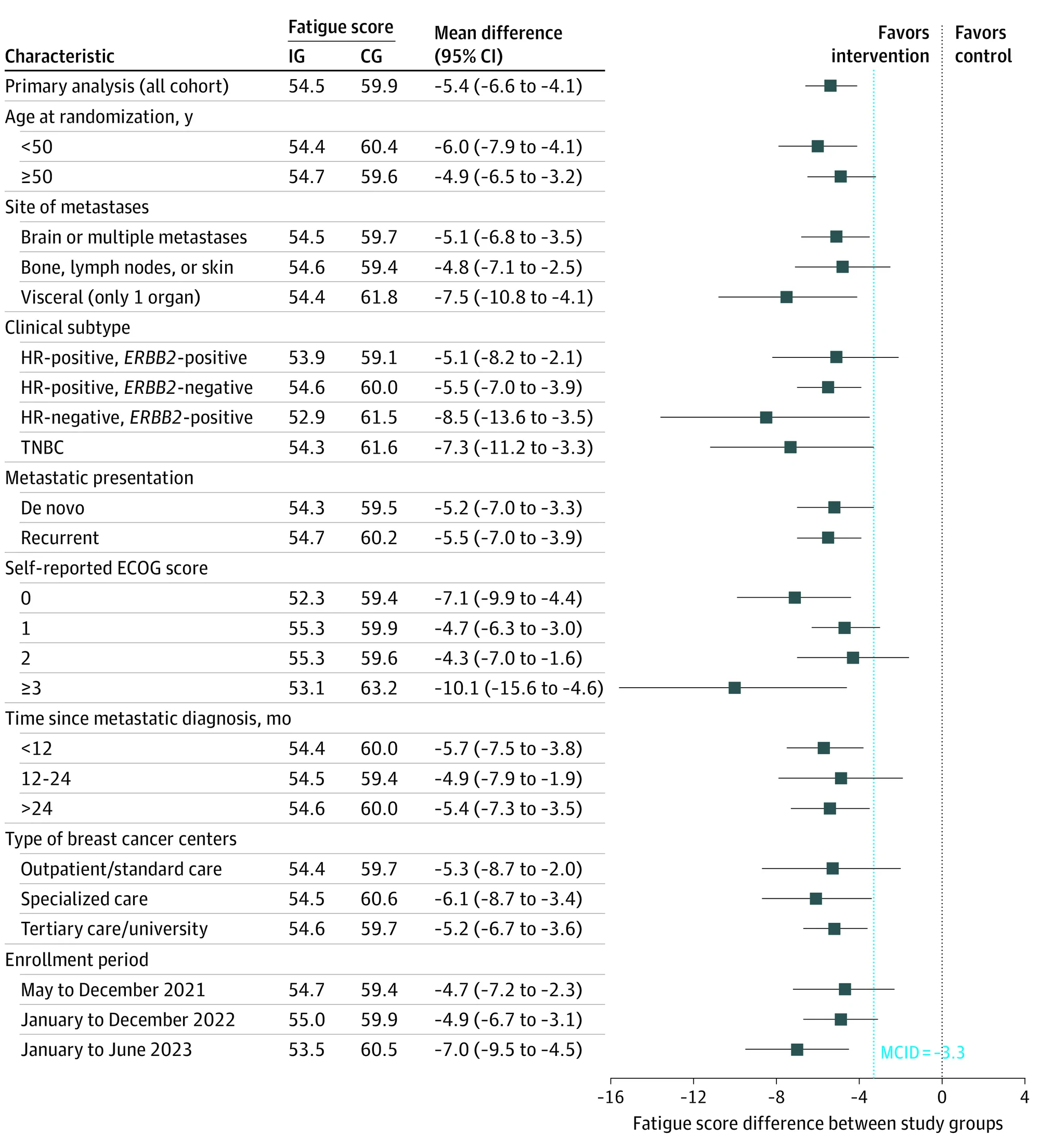
Dot Plot of Fatigue T Scores at 6 Months The squares represent the estimated mean difference in fatigue T scores between the study groups, with error bars illustrating the 95% CIs. The dashed line represents the minimally clinically important difference (MCID) of 3.3 points. The MCID is achieved when the CIs do not cross the dashed line. Higher T scores indicate greater levels of fatigue. CG indicates control group; ECOG, Eastern Cooperative Oncology Group (classification); IG, intervention group; HR, hormone receptor; TNBC, triple-negative breast cancer.

Baseline mean (SD) fatigue T scores were 57.6 (9.5) in the intervention group and 60.2 (9.0) in the control group (*P* < .001), with baseline PROs assessed after randomization but before group-specific follow-up began. At 6 months, adjusted mean fatigue was 54.5 (95% CI, 53.7- 55.4) vs 59.9 (95% CI, 59.0-60.8), yielding a mean difference of −5.4 points (95% CI, −6.6 to −4.1; *P* < .001) (Figure 3) and exceeding the MCID of 3.3 points. Sensitivity analyses assessing different missing data assumptions and analytic approaches showed that the between-group differences in fatigue remained consistent in direction and magnitude, supporting robustness (eTable 6 in Supplement 2). No treatment-by-subgroup interactions were observed; descriptive subgroup estimates consistently favored the intervention (Figure 3; eTable 7 in Supplement 2).

### Secondary Outcomes

#### Fatigue, PF, and HRQoL Over 12 Months

**Figure 4.**
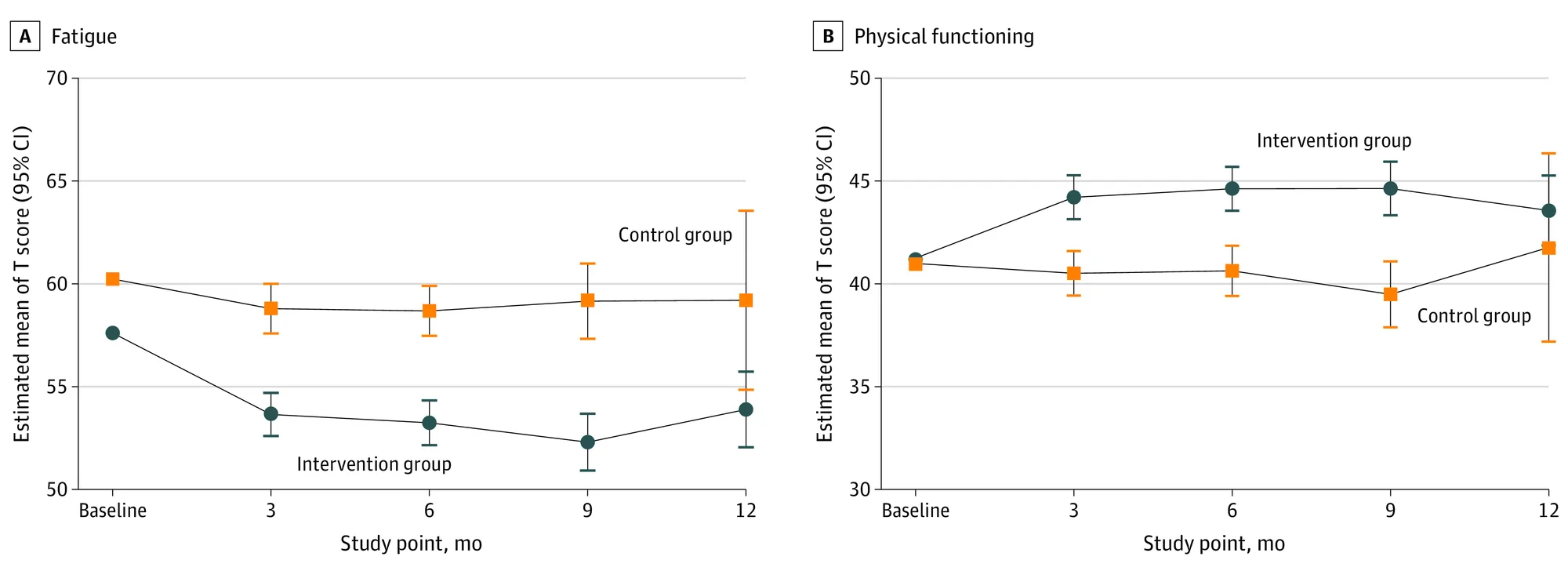
Line Graphs of Fatigue and Physical Functioning T Scores Over 12 Months Values are estimated marginal means, and error bars indicate 95% CIs. Higher T scores indicate greater levels of fatigue and better physical functioning. The minimal clinically important difference was 3.3 points for fatigue reduction and 3.2 points for improvement in physical functioning.

Between-group differences favored the intervention at all points: −5.2 (95% CI, −6.6 to −3.8) at 3 months, −6.8 (95% CI, −9.0 to −4.7) at 9 months, and −5.2 (95% CI, −9.0 to −1.4) at 12 months (Figure 4A; eTable 8 in Supplement 2). Between-group mean differences were 3.7 (95% CI, 2.4-5.0) at 3 months, 4.0 (95% CI, 2.6-5.4) at 6 months, and 5.1 (95% CI, 3.2-7.1) at 9 months (Figure 4B; eTable 8 in Supplement 2), exceeding the MCID of 3.2 points. For HRQoL over 12 months, adjusted mean HRQoL T scores ranged from 35.7 to 36.3 in the intervention group and 35.3 to 35.7 in the control group (eFigure 3 and eTable 8 in Supplement 2). At 6 months, the between-group mean difference was 0.8 (95% CI, 0.02 to 1.5), which did not reach the MCID of 2.9 points.

#### EF Over 12 Months

EF was numerically higher in the intervention group at baseline and over follow-up; at 6 months, the mean between-group difference was 4.3 points (95% CI, 2.8-5.8) (eFigure 4 and eTable 8 in Supplement 2). Sensitivity analyses showed a generally consistent pattern of results, particularly for the primary 6-month outcome (eTable 8 in Supplement 2). Later point estimates, especially at 12 months, were more sensitive to analytic assumptions because data were sparser. Stratification by enrollment period showed consistent group differences across PRO outcomes, supporting the stability of the intervention effect over time (eFigure 5A-D in Supplement 2).

#### Exploratory Clinical Outcomes

Protocol-specified subgroup analyses of OS were conducted for patients with visceral metastases and triple-negative breast cancer. Among patients with visceral metastases and triple-negative breast cancer, the adjusted hazard ratios (HRs) (aHRs) were 0.37 (95% CI, 0.12- 1.15) and 0.73 (95% CI, 0.30- 1.78), respectively (eFigure 6B and D in Supplement 2).

In an additional exploratory analysis of the full cohort, the absolute 12-month OS difference was 2.9 percentage points (87.6% in the intervention group; 84.7% in the control group; eFigure 6A in Supplement 2). The aHR was 0.72 (95% CI, 0.52- 0.99) (eTable 9 in Supplement 2). Among patients with HR-positive/*ERBB2*-negative disease, 12-month OS was 88.5% vs 82.7% (aHR, 0.69; 95% CI, 0.47- 1.00) (eFigure 6C in Supplement 2).

These analyses were exploratory and underpowered; loss to follow-up was higher in the control group (26.7% vs 14.3%), and differential censoring could be excluded. Time to first systemic therapy change did not differ between groups (aHR, 0.89; 95% CI, 0.74-1.08) (eFigure 7 in Supplement 2).

#### Health Care Utilization

In the claims data subpopulation, 235 of 909 patients (25.9%) were included, with similar proportions in both groups. At 12 months, hospitalization and ED visit rates were lower in the intervention group (IRR, 0.52; 95% CI, 0.45-0.78; and IRR, 0.76; 95% CI, 0.63-0.92, respectively) (eTable 10 in Supplement 2).

## Discussion

In this multicenter randomized clinical trial, alert-based PRO monitoring was associated with clinically meaningful and sustained reductions in fatigue and improvements in PF in patients with mBC. However, the results should be interpreted cautiously. Baseline assessments, including PROs, were collected after randomization, questionnaire completion rates were higher in the intervention group, and attrition was greater among patients with poorer self-reported performance status. Each of these features could have amplified between-group differences, especially at later points. Although the 6-month result was consistent across sensitivity analyses, later estimates were more sensitive to model assumptions because of increased missingness and fewer patients remaining at risk.

Prior randomized clinical trials of ePRO monitoring have shown improvements in symptom burden and, in some settings, OS benefit.^1,2,4,5,31^ Most of these trials enrolled heterogeneous cancer populations,^1,2,6,8^ and early survival-positive studies were not conducted in disease-specific multicenter breast cancer settings.^4,5^ The PRO B trial addressed this gap by providing disease-specific randomized evidence on patients with mBC across diverse breast cancer centers.

Fatigue was chosen as the primary end point because it is common and debilitating in patients with mBC, affects daily functioning and HRQoL, and may reflect disease burden, treatment-related adverse effects, and psychosocial stress.^13,15,16,17,18,32^ In the PRO B trial, the reduction in fatigue exceeded the MCID and persisted over follow-up, although these between-group differences should be interpreted in light of the observed baseline imbalance and potential biases noted previously. EF was also higher in the intervention group over follow-up; however, this domain showed a numerical baseline difference between groups and was not a prespecified end point.

We did not observe a difference in time to first systemic therapy change, so our data do not support earlier changes in systemic therapy as the primary mechanism of benefit. Instead, the intervention may have improved symptom outcomes through earlier clinical contact, supportive care interventions, or enhanced patient-clinician communication. Health care utilization findings were consistent with the direction of the PRO outcomes, with lower hospitalization and ED visit rates observed in the intervention group within the claims data subpopulation. These findings may reflect earlier recognition of clinical deterioration, timely telephone contact, and supportive care before symptoms required urgent evaluation. However, because claims data were available for only approximately one-quarter of the trial population and were restricted to patients who were insured by participating health insurance companies, these results should be interpreted as supportive and exploratory. The survival signal was also exploratory and should not be interpreted as confirmatory.

Alerts were generally triggered by combined deterioration across multiple PRO domains or worsening on the health transition item rather than changes in a single domain. Functional, emotional, and role domains were most commonly involved, suggesting that early multidomain decline may prompt timely clinical reassessment and that enhanced psychosocial support may have contributed to benefits. Symptom domains triggered fewer alerts, possibly because acute symptoms, such as nausea, vomiting, or diarrhea, can resolve between weekly assessments and ad hoc symptom reporting was not available. Conduct across certified breast cancer centers and the use of nonprescriptive alert responses support the relevance of the PRO B trial to routine oncology workflows, particularly in settings where digital PRO monitoring is not yet routinely implemented.

### Limitations

This study had limitations. First, only patients with smartphone access and German language proficiency could be enrolled. Second, baseline self-reported measures, including PROs, were collected after randomization. Although study group assignment was not explicitly disclosed before baseline completion, the trial was not blinded, and participants may have become aware of allocation because follow-up schedules differed. This may have introduced expectancy effects or contributed to baseline imbalances. Third, questionnaire completion rates were higher in the intervention group, and dropout patterns differed between groups, raising the possibility of selection bias despite consistent sensitivity analyses. Fourth, the primary end point was changed early in the trial from 12-month to 6-month fatigue, which should be considered when interpreting 12-month estimates. Fifth, reasons for treatment changes were not separately captured, and clinician use of the intervention web portal was not formally tracked. Finally, survival status was obtained through site follow-up without registry linkage; because loss to follow-up was higher in the control group, informative censoring could not be excluded. Survival analyses were exploratory and hypothesis generating. Health care utilization data were derived from claims and limited to 26% of the full cohort, limiting generalizability.

## Conclusions

In this randomized clinical trial, alert-based ePRO monitoring was associated with clinically meaningful reductions in fatigue and improved PF and may improve symptom-focused care for patients with mBC. Effects on longer-term PRO outcomes, health care utilization, and survival require confirmation.

## Notes

Supplement 1. Trial protocol and statistical analysis plan

Supplement 2. eMethods. eTable 1. Items included in the EORTC CAT Core short forms eTable 2. Deterioration cut-points of the EORTC T-score and health transition item eTable 3. Exploring patients’ characteristics at baseline by type of missing data and fully observed data eTable 4. Comparison of patient characteristics at baseline in patients who had missing data or loss to follow-up between the study group eTable 5. Baseline patient characteristics in observed data and after multiple imputation (imputed data set, m = 30) eTable 6. Primary outcome: Fatigue T-score at 6 months eTable 7. Subgroup analyses of fatigue T-score at 6 months post-randomization eTable 8. The estimated marginal mean and 95% CI for each time point of the T-scores eTable 9. Overall survival analysis eTable 10. Hospitalizations and emergency room visits eFigure 1. The bar graph represents the PRO questionnaire return rate at each follow-up time point eFigure 2. Frequency of PRO domains triggering alerts (number of alerts = 3,019) eFigure 3. Health-related quality of life (HRQoL) T-scores over 12 months eFigure 4. Emotional functioning T-scores over 12 months eFigure 5. Estimated marginal mean PRO T-scores over 12 months by study group, stratified by enrollment period eFigure 6. Kaplan-Meier Curves of overall survival eFigure 7. Time to first change of systemic therapy eReferences.

Supplement 3. Data sharing statement
