# Supplementary material for: Electronic Patient-Reported Outcome Monitoring With Alert-Based Interventions in Metastatic Breast Cancer: A Randomized Clinical Trial
Source: JAMA Oncol. 2026 Aug 6;12(10):1069–78. doi: 10.1001/jamaoncol.2026.2661 (PMC13448833; doi:10.1001/jamaoncol.2026.2661)
Supplement: Supplementary file 1 [file jamaoncol-e262661-s001.pdf]

This supplement contains the following items:

- **Final Protocol**
    - Summary of Changes
- **Original Protocol**
- **Original Statistical Analysis Plan (an only version)**
- **Original Protocol and SAP (German version)**

Study protocol

PRO B – Patient Reported Outcomes in Breast Diseases

New Approaches to the Care of Metastatic Breast Cancer

Short title: PRO B

Indication: metastatic breast cancer

Intervention: Patient- Reported Outcomes (PROs)

DRKS-ID: DRKS00024015 **/ UTN** U1111-1263-4946

Funding: Innovation Committee at the G-BA

Document history – Changes compared to previous version of Study protocol.

Version	Date	Changes
3.0	22.08.2023	Synopsis:<br>▪ Removed number of medical consultations, Progression-free survival after 12 months, and number of therapy changes according to no data available<br>▪ Added time to first systemic therapy changes to secondary endpoints<br>▪ Corrected inclusion criteria by removing tablet or email<br>▪ Updated project duration to 48 months<br>▪ Updated number of study centres to 52<br>Section 2.2 Secondary objective and secondary endpoints:<br>▪ Removed number of medical consultations, Progression-free survival after 12 months, and number of therapy changes according to no data available<br>▪ Added time to first systemic therapy changes<br>Section 3.2 Schedule:<br>▪ Updated a total duration of the study to 48 months due to the longer of study recruitment<br>▪ Updated recruitment period to May 2021 – June 2023<br>▪ Updated follow-up and monitoring to May 2022 – February 2024<br>▪ Updated data analysis and dissemination to February 2024 – September 2024<br>Section 4.2 Study centres:<br>▪ Updated the number of breast cancer centres to 52<br>Section 4.3 Inclusion criteria:<br>▪ Corrected the inclusion criteria by removing tablet or email<br>Section 5.3 Randomisation:<br>▪ Removed the number of study centres and stated only centres<br>Section 5.5 Possible confounders:<br>▪ Removed the number of people in the household due to no data is available<br>Table 2 Overview of primary outcome and secondary outcomes:<br>▪ Removed number of therapy changes and progression-free survival<br>▪ Added time to first systemic therapy changes<br>Section 7.4 Analysis of the primary hypothesis:<br>▪ Updated the definition of intention-to-treat analysis set by including all randomized cases and who answered at least the PRO Survey. Patients who never respond to the PRO survey are not considered to the full analysis set (modified ITT population)<br>Section 7.4 Dealing with missing values:<br>▪ Added a sentence regarding no observation after six months in late recruitment patients. Therefore,

		no multiple imputation will be applied to those patients.<br><u>Section 7.4 Statistical analyses of the subgroups:</u><br>▪ Changed age at randomization instead of age at diagnosis of metastases due to no information is available<br>▪ Removed disease free interval due to no data available<br>▪ Added therapy at study inclusion (chemotherapy, endocrine therapy, anti-Her2 therapy, immunotherapy, targeted therapy and others)<br>▪ Added change in alert criteria (before and after 01.09.2022) to the subgroup analysis<br><u>Section 7.4 Health economic evaluation:</u><br>▪ Changed the cost per year instead of per month<br>▪ Changed statistics model for the cost analysis to do a log-transformation and use linear mixed model accounting for a study centre as a random effect (random intercept)
2.3	19.07.2021	<u>Section 2.1 – Primary objective and primary endpoint:</u><br>▪ Changed the primary endpoint to “tumour-related fatigue […] 6 months after inclusion” instead of “tumour-related fatigue […] 12 months after inclusion”<br><u>Section 2.2 – Secondary objective and secondary endpoints:</u><br>▪ Added the number of “medical consultations”<br>▪ Changed secondary endpoint fatigue (EORTC Fatigue Score) “by 12 months” instead of “by six months”<br>▪ Changed secondary endpoint health-related quality of life (EORTC HRQoL score) “after six and 12 months” instead of “after 12 months”<br><u>Section 3.1. Study design</u>:<br>▪ Changed the responsible team to contact the patient in case of deteriorating PRO values to “the treating breast cancer centre” instead of “the treating physician”<br><u>Section 3.2 Schedule:</u><br>▪ Changed the preparation phase and study initiation to “October 2020 – April 2021” instead of “October 2020 – March 2021”<br>▪ Changed the recruitment of patients to “May 2021 – May 2022” instead of “April 2021 – March 2022”<br>▪ Changed the follow-up and monitoring to “May 2022 – May 2023” instead of “April 2022 – March 2023”<br>▪ Changed the data analysis and dissemination to “May 2023 – September 2023” instead of “April 2023 – September 2023”<br><u>Section 4.2 Study centres:</u><br>▪ Changed the number of breast cancer centres to “approx. 40” instead of “40”<br><u>Section 4.3 Inclusion criteria:</u><br>▪ Added a footnote to the possible health insurance companies: “* A temporary opening of the study to insured persons from other health insurance

		companies is possible after approval by the sponsor to extend the legal basis of the study."<br>▪ Specified the inclusion criteria by adding "The prerequisite for participation in the study is the patient's informed consent."<br><u>Section 5.1 Description of the intervention:</u><br>▪ Changed the responsibility of decisions regarding intervention/measures following an alarm to "the treating breast cancer centre" instead of "the treating physician"<br>▪ Changed the responsibility of contact following an alert to "the treating breast cancer centre" instead of "the treating physician"<br><u>Section 5.3 Randomization:</u><br>▪ Changed the randomization strata:<br>- "types of metastasis (3 groups: bone metastases & skin metastases & lymph node metastases; visceral metastases (single organ); brain metastases & multiple metastases)" instead of "types of metastasis (4 groups: exclusively osseous metastases; visceral metastases; brain metastases; multiple metastases)"<br>- "histological findings (2 groups: luminal (HR+/HER-) & triple positive (HR+/HER+); HR-/HER+ & triple negative)" instead of "histological findings (4 groups: luminal (HR+/HER-); triple positive (HR+/HER+); HR-/HER+; triple negative)"<br><u>Section 5.5 Possible confounders:</u><br>▪ Changed the "Charlson comorbidity index" to "NCI Comorbidity Index"<br>▪ Made changes to the comorbidities possibly influencing the primary outcome:<br>- Added "paraplegia"<br>- Removed "dysmenorrhea or hypermenorrhoea" and "ovarian cysts"<br><u>Section 6.1 Description of the course of the study:</u><br>▪ Changed the wording in the description of the intervention: "alert" instead of "alarm" system<br><u>Section 7.1 Case number calculation:</u><br>▪ Corrected the wording to "between the intervention and control groups" instead of "between the intervention and treatment groups"<br><u>Section 8.1 Data collection:</u><br>▪ Added the name of the web tool used for data collection "web tool developed by NeuroSys (PatientConcept)"<br>▪ Changed the wording for referring to the web tool: "PatientCocept-Portal" instead of "web tool"<br>▪ Changed the description of data merging and quality checking to "The sum of all project data (clinical tumour data, study data and survey data) from each individual centre is then quality-checked via functions in OncoBox. Data sets from all centres are then merged by OnkoZert using the OncoBox Research."

		instead of "The sum of all project data (clinical tumour data, study data and survey data) from each individual centre is then merged via the OncoBox, quality-checked and sent to OnkoZert as an XML or JSON file via a secure environment (e.g. Citrix environment). The XML or JSON data sets from all centres are then merged by OnkoZert using the OncoBox Research."<br>Section 8.2 Data processing, quality control, monitoring:<br>▪ Changed the description of data export and quality assessment to "Before the export of the data sets, PRO B Doc checks the data for plausibility and completeness (quality assurance) via functionality of OncoBox. In addition, OnkoZert carries out a quality control of the data sets in accordance with the OncoBox criteria." instead of "Before the data sets are sent by the study centres, OncoBox checks the data for plausibility and completeness (quality assurance). In addition, OnkoZert carries out a quality control upon receipt of the data sets in accordance with the OncoBox criteria."<br>Section 8.3 Data storage and data protection:<br>▪ Changed the wording for referring to the web tool: "PatientCocept Portal" instead of "web tool"

Table of contents

Synopsis ... 1

Responsibilities ... 3

1 Rationale ... 4

1.1 Scientific Background ... 4
1.2 Justification for the study ... 5
1.3 Benefit-risk assessment ... 5

2 Objectives of the study ... 6

2.1 Primary objective and primary endpoint ... 6
2.2 Secondary objectives and secondary endpoints ... 6

3 Study design and schedule ... 6

3.1 Study design ... 6
3.2 Schedule ... 7

4 Recruitment ... 7

4.1 Number of cases ... 7
4.2 Study centres ... 8
4.3 Inclusion criteria ... 8
4.4 Exclusion criteria ... 8
4.5 Termination criteria ... 8

5 Intervention ... 9

5.1 Description of the intervention ... 9
5.2 Risks of the intervention ... 9
5.3 Randomization ... 10
5.4 Blinding ... 10
5.5 Possible confounders ... 10

6 Course of study ... 11

6.1 Description of the course of study ... 11
6.2 Planned treatment after study end ... 12

7 Statistical approach ... 12

7.1 Sample size calculation ... 12
7.2 Variables ... 13
7.3 Pretest ... 14
7.4 Statistical Methods ... 14

8 Data management ... 16

8.1 Data collection....16
8.2 Data processing, quality control, monitoring....17
8.3 Data storage and data protection....18
9 Ethical and legal aspects....19
9.1 Legal basis....19
9.2 Ethics committee....19
9.3 Patient information and consent form....19
9.4 Expense allowance....19
9.5 Insurance....19
10 Administrative aspects....20
10.1 Study financing....20
10.2 Study reports....20
10.3 Study registration....20
10.4 Publications....20
11 Signature of the Principal Investigator....20
Bibliography....21
Abbreviations....24
Appendices....25

Synopsis

Title	**PRO B** – New approaches to the care of metastatic breast cancer
Indication	metastatic breast cancer
Study objectives	Evaluation of the effects of intensified patient-reported outcome (PRO) monitoring on quality of life, overall survival and treatment costs specifically for patients with metastatic breast cancer<br><br><u>Primary endpoint:</u><br>Fatigue after 6 months<br><br><u>Secondary endpoints:</u><br>1. Number of hospital and emergency room visits after 6 and 12 months<br>2. Physical function (EORTC Physical Functioning Score) after 6 and 12 months<br>3. Fatigue (EORTC Fatigue Score) after 12 and possibly 36 months<br>4. Overall survival with visceral metastasis after 12 months<br>5. Overall survival in triple-negative breast cancer after 12 months<br>6. Time to first systemic therapy changes<br>7. Health-related quality of life (EORTC HRQoL score) after 6 and 12 months
Study design	PRO B is a prospective, multicentre, randomised two-arm intervention study to implement and evaluate PRO measurements in patients with metastatic breast cancer.<br><br><u>Intervention arm</u>: weekly PRO survey with, if applicable, contact and, if necessary, measures by the treating team if the values deteriorate<br><br><u>Control arm</u>: PRO survey every three months without contact
Sample	<u>Inclusion criteria: (following informed consent)</u><br>- Female<br>- Statutory health insurance with one of the following health insurance companies: BARMER, DAK Gesundheit, BKK · VBU*<br>- Diagnosis of metastatic breast cancer<br>- Treatment at a participating breast cancer centre<br>- Completion of the 18th year of life and ability to consent<br>- Receiving drug treatment due to metastatic breast cancer<br>- Life expectancy at study inclusion of more than 3 months<br>- ECOG 0-2<br>- Willingness to participate in a weekly, online-based PRO survey<br>- access to the Internet (smartphone)<br><br><u>Exclusion criteria:</u> no active tumour therapy (comfort care)<br><br><u>Termination criteria</u>: Patient withdraws consent to participate in the study<br><br>*A temporary opening of the study to insured persons from other health insurance funds is possible after approval by the sponsor to extend the legal basis of the study.
Sample size	500 subjects per study arm

Project duration	48 months
Statistical approach	- Primary hypothesis: ANCOVA model<br>- Secondary hypotheses: e.g. time-event models, ANCOVA, logistic regression, Cox regression<br>- Subgroup analyses<br>- Health economic analyses
Number of study centres	52 breast cancer centres
Study financing	Innovation Committee of the Federal Joint Committee (grant number: 01NVF19013)

Responsibilities

**Principal Investigator**	**PD Dr. med. Maria Margarete Karsten**<br>Clinic for Gynecology with Breast Center<br>Charité Campus Mitte<br>Charité – Universitätsmedizin Berlin<br><br>Charitéplatz 1<br>10117 Berlin<br><br>+49 (0)30 450 664 279<br>maria-margrete.karsten@charite.de
**Evaluation**	**PD Dr. phil. Ulrike Grittner**<br>Institute for Biometry and Clinical Epidemiology<br>Charité - Universitätsmedizin Berlin<br>Charité Campus Mitte<br><br>Charitéplatz 1<br>10117 Berlin<br><br>+49 (0)30 450 562 174<br>ulrike.grittner@charite.de
**Psychometric testing**	**Prof. Dr. med. Matthias Rose**<br>Medical Clinic with Psychosomatics<br>Charité - Universitätsmedizin Berlin<br><br>Charitéplatz 1<br>10117 Berlin<br><br>+49 (0)30 450 553 001<br>matthias.rose@charite.de
**Technical implementation**	**Dr. med. Julia Ferencz**<br>OnkoZert GmbH<br><br>Gartenstraße 24<br>89231 Neu-Ulm<br><br>j.ferencz@onkozert.de
**dissemination and support in recruiting study centers**	**Clara Breidenbach**<br>German Cancer Society<br><br>Kuno-Fischer-Straße 8<br>14057 Berlin<br><br>+49 (0)30 322 932 934<br>breidenbach@krebsgesellschaft.de
**Contract Special Care and Routine Data**	BKK · VBU<br>BARMER<br>DAK Gesundheit
**Study centers**	Breast cancer centers
**Project funding**	Innovation Committee at the Federal Joint Committee

1 Rationale

1.1 Scientific Background

Breast cancer is the most common cancer and cause of cancer-related death in women worldwide. Despite all the advances in research and clinical practice, up to 30% of those affected still develop metastases (1). According to the Center for Cancer Registry Data of the Robert Koch Institute, more than 18,000 patients die each year in Germany alone as a result of metastatic breast cancer (2). When metastases appear, not only the living conditions of the patients change fundamentally, but also the therapeutic approach. The focus of treatment is no longer on curing the disease, but rather on controlling the tumour and the associated prolongation of survival while simultaneously controlling the side effects of therapy. The goal is to maintain the best possible quality of life for as long as possible.

The overall survival of patients with metastatic breast cancer has improved significantly over the last few decades thanks to more modern treatment options, and at 38 months in 2010 was already 17 months above the median overall survival in 1990 (3). However, life expectancy in the case of metastatic breast cancer depends largely on the biological properties of the underlying tumour. In a meta-analysis, Caswell-Jin et al. showed a median survival of 57 months for patients with hormone receptor-positive tumours compared to a survival time of only 33 months for triple-negative breast cancer (3). With the aim of extending the period of survival, most patients undergo systemic tumour therapy during this time. Unlike in the adjuvant situation, in which treatment decisions can be relatively easily based on the standards of the professional societies, in the metastatic situation they must be made on a much more individual basis. Taking into account the medical history, current complaints and, above all, the patient's wishes, the decision-making process is time-consuming and must be questioned again and again due to the progressive course of the disease. At this point, information collected via so-called patient-reported outcomes (PROs) is helpful in making the best possible treatment decisions. PROs enable the assessment of health-related aspects (such as symptoms of disease, psychosocial stress and quality of life) from the patient's perspective without intervening interpretation by another person (e.g. doctor) (4). They thus contribute to improving

- the early detection of possible disease progression,
- the assessment of therapy-associated toxicity and
- the detection of a deterioration in the so-called Health-related Quality of Life (HRQoL).

The particular challenge of assessing the severity of certain disease symptoms or medication side effects is often underestimated by the attending physicians (5-7). This particularly applies to non-specific symptoms such as constipation, loss of appetite and fatigue, although the latter can often be the first clinical indicator of disease progression (8). In this context, PRO measurements are helpful because they enable treating physicians to obtain a very differentiated picture of their patients' state of health within a very short time. Especially in the metastatic situation, in which patients should spend as little time as possible in the clinic and therapies are therefore usually carried out on an outpatient basis, regular PRO measurements enable real-time monitoring of symptoms and side effects as well as timely interventions by treating physicians. Müller et al. report that even a slight deterioration in HRQoL in patients with metastatic breast cancer is associated with disease progression (9). The possibility of using PRO measurements to detect such deterioration and thus, if necessary, disease

progression at an early stage can spare those affected from further cycles of tumour therapy that is no longer effective.

1.2 Justification for the study

With an average overall survival of 38 months after metastasis and 18,570 women dying of breast cancer each year (2,3), it can be assumed that there are currently more than 60,000 patients with metastatic breast cancer in Germany. Treating these seriously ill women represents a challenge for the entire clinical team from a medical, psychosocial and economic perspective. There are no curative treatment options, so disease progression is inevitable. In addition, with every change in the treatment regimen in refractory disease, the likelihood of response decreases - in previously treated patients, it can be less than 15% (10). From a psychosocial perspective, considerable care and preventive measures are necessary due to the high level of disease-related psychological and emotional stress (11). In addition, the cost of treating metastatic breast cancer is currently estimated at around 100,000 US dollars per patient per year (12, 13). The combination of the high burden of disease on those affected, the high burden on the treatment team due to the complexity of the disease and the equally high financial requirements make innovations in the care of patients with metastatic breast cancer urgently necessary.

In a ground-breaking monocentric study of 766 patients with metastatic tumour disease, Basch et al. were able to show that intensified PRO monitoring could reduce the rates of unplanned emergency room stays and hospitalizations, but above all also significantly improve the quality of life and even the overall survival of the study participants (HR: 0.83; 95% CI: 0.70-0.99; p=0.04) (14, 15). Shortly thereafter, the benefits of intensified PRO monitoring were confirmed in another study on metastatic lung cancer patients. In this study, Denis et al. showed that the overall survival of patients treated with PROs could be extended by about eight months compared to the control group (HR: 0.50; 95% CI: 0.31-0.81; p=0.005) (16).

To our knowledge, there have been no studies to date on the effects of intensified PRO monitoring on quality of life, overall survival and treatment costs specifically for patients with metastatic breast cancer. PRO B is therefore intended to examine whether the results of the previously cited authors can be transferred to the usual care of this specific patient group in Germany. In order to enable simple and cost-effective transfer to standard care in the event of positive results, existing IT elements of the certification infrastructure of the German Cancer Society (DKG) and OnkoZert are used. PRO B therefore expands a long-established, comprehensive and cross-centre standardized collection of health data in order to enable a better assessment of the course of the disease of metastatic breast cancer and the associated effects on the quality of life of the affected patients.

1.3 Benefit-risk assessment

A study-specific health risk is not to be expected, as the study does not prescribe any specific treatment that deviates from the usual clinical routine. The assumed benefit for the participating patients consists in individual monitoring of the quality of life and, on this basis, measures initiated in the event of poor quality of life (personal benefit). If the evaluation of the intervention is positive, the benefit for future patients (group benefit) consists in an individualized and patient-centred form of care.

2 Objectives of the study

With PRO B, a new, individualized and patient-centred form of care is created and evaluated, taking into account the available resources. The intervention described under point 5 is carried out during the one-year recruitment period and the following one-year intervention period. Accordingly, the intervention duration is at least 12 and a maximum of 24 months. The outcomes listed below (after 6 and 12 months) are available at the end of the recruitment and intervention period for *all* included subjects (minus dropouts). However, they are also analysed for the later measurement points (up to a maximum of 24 months after inclusion) with a smaller n in order to be able to depict the PRO course for metastatic breast cancer patients over the longest possible period.

2.1 Primary objective and primary endpoint

The study aims to reduce disease-related morbidity and improve the quality of life of patients with metastatic breast cancer. The primary endpoint is therefore tumour-related fatigue, measured using study-specific items from the CAT item bank of the European Organization for Research and Treatment of Cancer (EORTC) 6 months after inclusion.

2.2 Secondary objectives and secondary endpoints

Secondary objectives are to improve overall survival and to reduce unplanned medical consultations, hospitalizations and emergency room stays. The following secondary endpoints were defined:

- Number of hospital and emergency room stays after six and 12 months
- Physical function (EORTC Physical Functioning Score) after six and 12 months
- Fatigue (EORTC Fatigue Score) by 12 months
- Overall survival in visceral metastases after twelve months
- Overall survival in triple-negative breast cancer after twelve months
- Time to first systemic therapy changes
- Health-related quality of life (EORTC HRQoL score) after six and 12 months

3 Study design and schedule

3.1 Study design

PRO B is a prospective, multicentre, randomized two-arm intervention study to implement and evaluate PRO measurements in patients with metastatic breast cancer.

After inclusion in the study, the patients are randomly assigned to either the intervention arm or the control arm (see Figure 1). Depending on the randomization, the patients are monitored weekly (intervention arm) or every three months (control arm) with a PRO measurement and in the intervention arm they are contacted by the treating breast cancer centre if the values deteriorate (see 5.1 Description of the intervention).

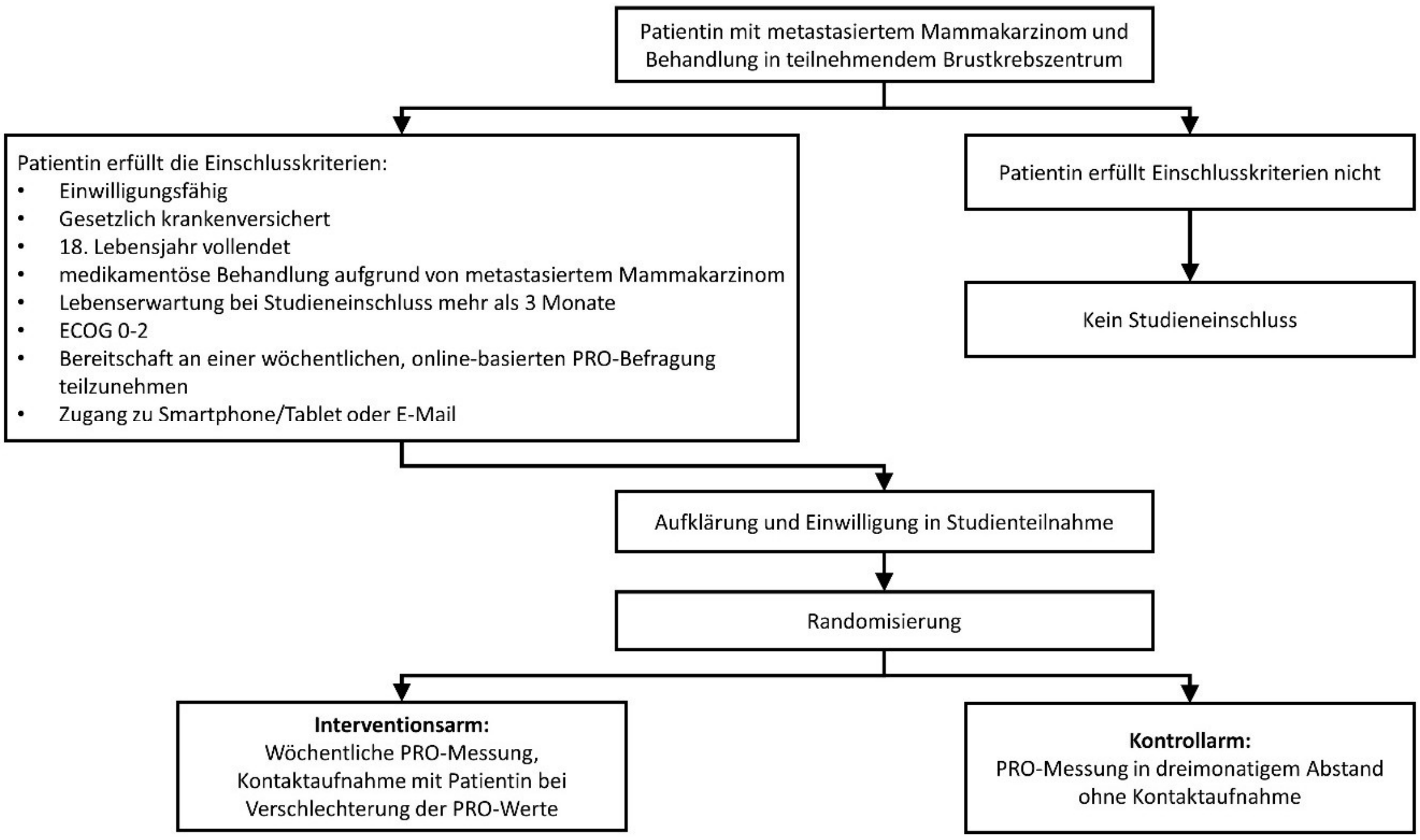

Figure 1 Study design

3.2 Schedule

The total duration of the research project is 48 months. Patient recruitment is scheduled for May 2021 and will last one year.

**Protocol**	**Version 2.3**	**Version 3.0 (updated)**
**Total duration**	36 months	48 months
**Preparation phase and study initiation**	October 2020 – April 2021	October 2020 – April 2021
**Recruitment of patients**	May 2021 – May 2022	May 2021 – June 2023
**Follow-up and monitoring**	May 2022 – May 2023	May 2022 – February 2024
**Data analysis and dissemination**	May 2023 – September 2023	February 2024 – September 2024

4 Recruitment

4.1 Number of cases

The target number of cases is 500 patients per treatment arm. The case number calculation is shown in chapter 7.1.

Reducing drop-outs/incentivising

In order to keep the drop-out rate in the control group as low as possible, patients receive a printout or a file of their survey data, i.e. their PRO course, after the end of the observation period, and patients are personally contacted if they do not respond to the three-monthly survey. Unlike in the intervention group, contact is made here only as a reminder to answer

the PRO survey. For this purpose, the participating centres are provided with individualized letters for each study patient with the request that they forward them to the patient. Alternatively, the participating centre also has the option of contacting the patient by telephone if the questionnaire is not answered and asking them to fill it out as part of their participation in the study. The PRO course can subsequently serve as a basis for discussion with the treating physicians and reveal any unnoticed abnormalities. By contacting the study centres, the patients in the control group are also made aware that they are an essential part of the study and that they are receiving no less attention from their treating physicians.

Following the feedback of the Charité breast cancer self-help group, the intensified digital monitoring of the PRO course is considered sufficient to incentivize the intervention group.

4.2 Study centres

52 breast cancer centres spread across the Federal Republic of Germany will be recruited as study centres. When recruiting the study centres, the number of cases and the proportion of patients with insurance with the cooperating health insurance companies BKK· VBU, BARMER, DAK Gesundheit will be taken into account. In addition, when recruiting, care will be taken to ensure that study centres with different degrees of urbanity, sponsorship and teaching status are included.

4.3 Inclusion criteria

Women can be included

- with metastatic breast cancer,
- who are treated in a breast cancer centre participating in the study,
- who are legally insured with one of the following health insurance companies: BARMER, DAK Gesundheit, BKK · VBU *,
- who are capable of giving consent,
- who have reached their 18th year of age,
- who are receiving drug treatment for metastatic breast cancer,
- who have a life expectancy of more than 3 months at study inclusion,
- classified as ECOG 0-2,
- who are willing to participate in a weekly, online-based PRO survey,
- and who have access to the Internet (smartphone).

The prerequisite for participation in the study is the patient's informed consent.

* A temporary opening of the study to insured persons from other health insurance companies is possible after approval by the sponsor to extend the legal basis of the study.

4.4 Exclusion criteria

Patients who are not receiving active tumour therapy (comfort care) or who do not meet all of the above inclusion criteria cannot participate in the study.

4.5 Termination criteria

Participation in the study will be terminated if the patient withdraws consent.

5 Intervention

5.1 Description of the intervention

Patients in the new form of care receive a request via app once a week, always on the same day of the week (or by email if necessary), to complete the PRO survey. Analogous to the target parameters of the study, the intervention concept is based on the operationalization of health-related quality of life of the EORTC Quality of Life group. Its core instrument, the QLQ-C30, includes five functional dimensions (Physical Function, Role Function, Emotional Function, Cognitive Function, Social Function) and 9 symptom scales (Fatigue, Nausea, Pain, Dyspnoea, Insomnia, Appetite loss, Constipation, Diarrhoea, Financial Difficulties). The named domains are collected using study-specific, optimized questionnaires from the respective EORTC item banks. For the health economic evaluation, the survey is also carried out using EQ-5D.

If the PRO scores deteriorate in the individual longitudinal course and/or in comparison to previously defined limit values, an alert is generated, which is displayed to the treating study centre and the PRO B study centre. Clinical relevance is calculated based on published information on the EORTC QLQ-C30 (e.g. MCID) and based on probabilities of actual changes in the latent variables. In order to detect an aggregation of several clinically irrelevant deteriorations over several weeks, a comparison with a previous measured value (e.g. four weeks previously) should also be made. An alert must be acknowledged and documented by the cooperating study centres within 48 hours (working days). The format of the feedback to the treating centres is developed jointly with them. In each case, it explicitly includes the domains and the extent of the change. The alert is followed by the respective centre contacting the patient to specify the deterioration in her values and to intervene if necessary. The intervention or measure that follows contact is not specified by PRO B. The type of measures is therefore left to the decision of the treating breast cancer centre .

In this respect, the study is not testing a specific therapeutic intervention for breast cancer patients, but rather a timely, individualized contact and possible intervention of various forms in the event of a deterioration in subjective quality of life parameters. The effect of the intervention is primarily due to the timely, individualized contact by the treating breast cancer centre due to alerting or alertingly deteriorating patient-oriented, quality of life-related parameters. This new form of care applies to all patients in the intervention arm for the entire duration of the study (24 months), but at least for twelve months.

5.2 Risks of the intervention

The intervention consists of entering health-related data into an app. Health risks are considered to be low.

The risk factors for the success of the intervention are the lack of reaction/intervention to a deterioration in the survey values by the treating team. In order to ensure that the patient is contacted correctly and, above all, in a timely manner, an alert system is integrated into the survey module that uses a stored alert algorithm to send an alert to the treating breast cancer centre and the study centre when the values deteriorate. If the alert is not acknowledged by the breast cancer centre within 48 hours (working days) and the intervention triggered by it is not documented, the study centre will contact the breast cancer centre by telephone.

5.3 Randomization

Randomization is carried out according to the following strata:

- Centres
- Types of metastasis (3 groups)
  - Bone metastases & skin metastases & lymph node metastases
  - Visceral metastases (single organ)
  - Brain metastases & multiple metastases)
- Histological findings (2 groups)
  - Luminal (HR+/HER-) & triple positive (HR+/HER+)
  - HR-/HER+ & triple negative

Due to the high number of strata, imbalances in the stratification variables between the intervention and control groups can arise in a simple randomization. In order to keep these as low as possible, adaptive randomization is carried out using the minimization method of Pocock and Simon (17). Randomization takes place after informed consent, download of the application and baseline survey (including sociodemographic information and comorbidities, see chapter 5.5).

5.4 Blinding

There is no blinding.

5.5 Possible confounders

As possible confounders, data on the following socioeconomic and sociodemographic parameters will be collected once during the initial survey:

- marital status
- relatives in need of care/minor children (≤14 years) in the household
- educational qualification/professional qualification
- employment status
- income
- migration background

In addition to the breast cancer-specific anamnesis (family history of cancer), the survey via the smartphone application also enables the assessment of any previous illnesses. We suspect that the following comorbidities have a significant influence on the primary endpoint of the study (fatigue). They must therefore be taken into account both in the interpretation of the measured values in the current project and in the final evaluation (e.g. as the NCI Comorbidity Index (underlined illnesses)):

- cardiac and cardiovascular pre-existing conditions (arterial hypertension, coronary heart disease, angina pectoris, <u>myocardial infarction</u>, <u>heart failure</u>, atrial fibrillation, valvular disease)
- previous vascular diseases (deep vein thrombosis, <u>peripheral arterial occlusive disease</u>, pulmonary embolism)
- cerebrovascular diseases (<u>TIA</u>, <u>stroke</u>)
- previous haematological diseases (anaemia)
- previous pulmonary diseases (<u>bronchial asthma,</u> <u>COPD</u>, sleep apnoea)
- endocrine/metabolic pre-existing diseases (hyperlipidaemia, <u>diabetes mellitus (requiring treatment with and without end-organ damage)</u>, hypothyroidism, obesity)

- previous rheumatological diseases (polymyalgia rheumatica, lupus erythematosus, rheumatoid arthritis, polymyositis)
- previous mental illnesses (depression, anxiety disorder)
- neurological pre-existing conditions (migraines, seizures, balance disorders, neuropathy, hemiplegia, paraplegia, dementia)
- previous gastrointestinal diseases (gastrointestinal ulcers, chronic inflammatory bowel disease, diverticulitis, reflux, irritable bowel syndrome, mild liver disease (liver cirrhosis without portal hypertension), severe liver disease (liver cirrhosis with portal hypertension, oesophageal variceal bleeding))
- previous gynecological diseases (infertility, abortion, , endometriosis)
- previous urogenital diseases (nephro- or urolithiasis, renal insufficiency, dialysis requirement, micturition problems, urinary tract infections, incontinence)
- infectious diseases (HIV, AIDS)
- previous oncological diseases (other tumour diseases (with and without metastases), leukaemia, lymphoma)
- previous operations
- drug history (alcohol, nicotine)

6 Course of study

6.1 Description of the course of study

Patients in the breast cancer centre who meet the inclusion criteria are informed about the study by the investigators. Provided informed consent is given, the patient is included in the study population. The app for the PRO surveys is then installed on the smartphone/tablet of the study participant, which can be used to conduct the survey. Participation by email will only be implemented if recruitment is slow. Initially, only participation by app is possible. If necessary, an amendment to the study protocol will regulate participation by email. The study participant is then randomized into a study arm.

In the intervention arm, the patient is then asked to take part in the PRO survey every week for at least 12 months and a maximum of 24 months via the app (or by email). If the values reported by the patient deteriorate, an alert is sent to the breast cancer centre via a web application, which then contacts the patient and coordinates possible measures. Alerts are acknowledged by the breast cancer centre in the web application and contacts and, if necessary, measures are documented. The PRO-B study centre also has access to the web application and can check alerts and the response of the breast cancer centres. If a breast cancer centre has not acknowledged an alert within 48 hours, the study centre contacts the breast cancer centre.

A patient in the control arm is asked every 3 months via the app or by email to take part in the PRO survey. The answers are also sent electronically to the web application immediately. The control group is not alerted if the PRO values deteriorate and therefore is not contacted. If the survey is not answered, the study centre sends a written or telephone reminder.

After the patient has given her consent, clinical data is documented during treatment in an Electronic Case Report Form (eCRF) (tumour and study documentation software PRO B Doc from OnkoZert). In addition, routine data is collected by the patient's health insurance company. Figure 2 provides an overview of the individual study process for each patient.

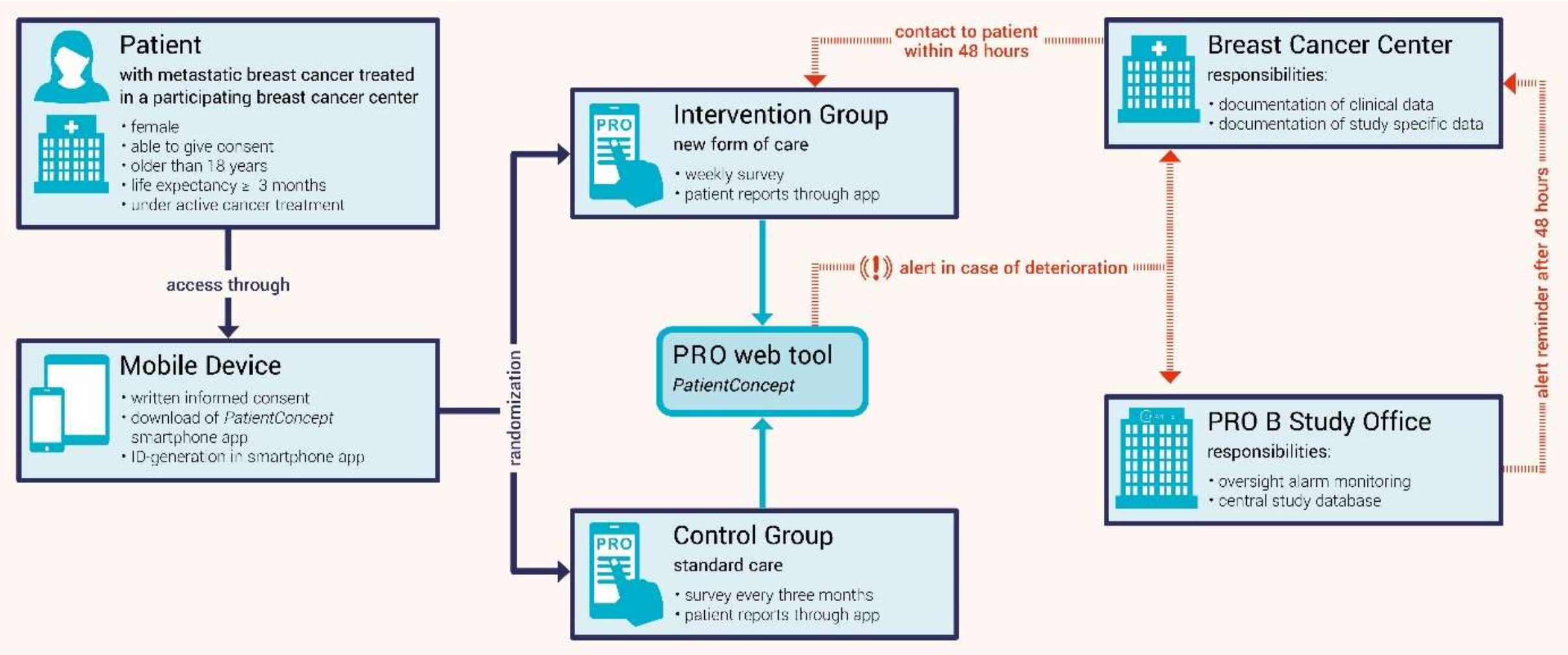

Figure 2 Individual study schedule

6.2 Planned treatment after study end

In the event of positive results, the newly created form of care can be incorporated into standard care with manageable effort due to its special structure. In this context, the patients would not receive any specific treatment that deviated from the usual clinical routine after the study, as well as during the study, but would continue to be monitored electronically with regard to their symptoms and quality of life. The care that is evaluated during PRO B could therefore be continued in a very similar form after the end of the study and expanded to other patient groups. This would also make it possible, for example, to implement and evaluate PRO-supported treatment and aftercare for breast cancer patients in the adjuvant situation.

7 Statistical approach

7.1 Sample size calculation

Cocks et al. created guidelines for the interpretation of longitudinal changes on the fatigue scale of the EORTC QLQ-C30, according to which values of an average improvement of 4 points or more are to be considered a small improvement and values of 9 points or more are to be considered a moderate improvement (18). However, PRO B is about a comparison between the treatment and control groups. The effect estimates follow the information provided by Cocks et al., but due to the two-group comparison, much larger variances are to be expected, which is why a conservative estimate of a standardized effect size of around 0.2 is to be achieved (mean difference, for example, 5 points and standard deviation 25.2 points). Such a variance is comparable to the information provided by Cortes et al. 2015 (19).

Since the analysis of certain subgroups, for example patients with visceral metastases, is particularly relevant, the aim is to have a sample size that is sufficiently large for subgroup analyses. The recruitment of 500 patients per group is considered realistic. With an expected drop-out rate of 20%, this results in 400 completed cases per treatment arm. If there is a minimal clinically relevant effect of 5 points mean difference on the fatigue scale of the EORTC QLQ-C30, as reported by Cocks et al. (18) (with a common standard deviation of 25), a two-sided t-test with a significance level of 5% has a power of 80.65% to detect a difference in the

fatigue score between the intervention and control groups with 400 patients per group. This calculation was carried out with nQuery (version 8.3.1.0). The number of cases is therefore sufficient for the detection of small effect sizes (0.2 or greater) and takes into account the number of relevant subgroups in the patient population.

As the planned sample size cannot be achieved in the originally intended recruitment period, the recruitment period will be extended by four months. The total expected sample size is 400 patients per group. With an expected drop-out rate of 20%, this results in approximately 320 completed cases per treatment group. This number of cases is therefore sufficient for the detection of effect sizes of 0.2 using a two-sided t-test for independent samples with a significance level of 5% and a power of 71.4%. If the effect size is 0.225 or larger, a power of at least 81.1% can be achieved.

7.2 Variables

The table below lists the primary outcome and the secondary outcomes of the study. Other variables that are relevant for randomization or as possible confounders are defined for the baseline survey and the study documentation system (eCRF in PRO B Doc). Data in PRO B Doc are collected every three months during the regular clinical presentation at the treating breast cancer centre.

Table 2 Overview of primary outcome and secondary outcomes

**Variable**	**Data source**	**Time of collection**			
		**Baseline**	**Weekly in intervention group; every 3 months in control group**	**6 months**	**12 months**
**Primary outcome**					
Fatigue Score (EORTC)	Study-specific questionnaire from EORTC item bank	x	x	x	x
**Secondary Outcomes**					
Physical functioning score (EORTC)	Study-specific questionnaire from EORTC item bank	x	x	x	x
Health-related Quality of Life sum score (EORTC)	Study-specific questionnaire from EORTC item bank	x	x	x	x
Number of hospital and emergency room stays	PRO B Doc/ health insurance data				x
Overall survival in visceral metastasis	PRO B Doc, health insurance data				x

Overall survival in triple negative breast cancer	PRO B Doc, health insurance data				x
Time to first systemic therapy changes	PRO B Doc				x

7.3 Pretest

The Pretest will include the measurement instruments specific to the study for monitoring the course of the study. In some cases, think-aloud techniques will be used, and in some cases a short evaluation questionnaire will be attached to the surveys, which will enable an evaluation of the survey content and the stress caused by answering it. Respondents for the pretest will be recruited in the Breast Centre of the Charité - Universitätsmedizin Berlin.

7.4 Statistical Methods

Statistical analysis of the hypotheses

The data is evaluated by the Institute of Biometry and Clinical Epidemiology (iBiKE) at the Charité Universitätsmedizin Berlin.

Analysis of the primary hypothesis

The primary question will be investigated using an intention-to-treat analysis for all randomized cases and who answered at least the PRO survey. Patients who never respond to the PRO survey are not considered to a full analysis set (modified ITT population). All randomization stratification variables will be considered as covariates in all statistical models.

A two-sided t-test was used to calculate the sample size, although an ANCOVA model is to be used in the evaluation. It was shown that this is a conservative approach for estimating the sample size of ANCOVA models, since an ANCOVA model with $(1 - 2\ \rho)*n$ observations -has at least the same power as a t-test with *n* observations (20) , where ρ is the variance inflation factor (correlation between the *baseline* measurement and the *follow-up* measurement). In the worst case of $\rho = 0$, this leads to a required number of cases equal to the t-test. The analysis is carried out in the ANCOVA model with the baseline measurement and types of metastasis and histology as covariates (analogous to the stratification of randomization). In addition, the analysis is adjusted for age, type and number of chemotherapies, ECOG status at study inclusion, computer experience and, if necessary, for other important confounders. The heterogeneity between the study centres is considered by *random effect* (mixed linear regression model, *random intercept model*).

Analyses of the Secondary Hypotheses

All secondary hypotheses are analysed analogously to the primary hypothesis and according to the respective outcome using suitable statistical methods (e.g. time-event models, ANCOVA, logistic regression, Cox regression). As with the analysis of the primary outcome, the secondary hypotheses are also adjusted for relevant confounders and the heterogeneity between the study centres is statistically considered appropriately. Additional outcomes and in particular different subgroups can be included in the analysis during the course of the study. In this case, they are listed in the statistical analysis plan (SAP) and evaluated exploratively.

Dealing with missing values

Missing values are imputed using multiple imputation methods, assuming that they are *missing at random* (MAR). In all cases, the reasons for missing values are documented and reported as best as possible. Due to the longer recruitment time of 4 months, the late recruited patients will only be fully followed for 6 months. No secondary endpoints at 12 months will be estimated for these patients using imputation methods.

Statistical analyses of the subgroups

For the statistical analyses of the subgroups, an interaction term of the treatment variable with the subgroup is initially included in the ANCOVA model (outcome: primary or secondary outcome, covariates: respective baseline variable, treatment variable, stratification variables of randomization, random intercept for the centers) in order to analyze initial indications of differential treatment effects in the subgroups. In addition, marginal effect estimates for each subgroup and 95% confidence intervals are given from the models. All subgroup analyses are exploratory.

Definition of subgroups and justification of the importance of subgroup analyses

- **metastasis** (secondary/therapy received, primary/therapy naïve)
- **tumour subtype** (HR+/HER2+, HR+/HER2–, HR–/HER2+, TNBC)
- **site of metastasis** (cerebral, visceral, bone, multiple)
- **age at randomization** (<50, ≥50)
- **therapy at study inclusion** (chemotherapy, endocrine therapy, anti-Her2 therapy, immunotherapy, targeted therapy and others)
- **relationship status** (partnership/marriage, single)
- **income** (median split)
- **depression** (yes/formerly, no)
- **BMI** (<30, ≥30 kg/m ^{2})
- **Change in alert criteria** (before and after September 1st, 2022)

Patients with **primary metastatic breast cancer** have a better prognosis than patients with recurrent metastatic disease. This difference is attributed to the fact that any adjuvant systemic therapies for the latter may lead to resistances that are not present in treatment-naive patients, i.e. those with primary metastases (21, 22). Within the group of **secondary metastatic tumours,** a longer disease-free interval (DFI) is considered to be prognostically favourable (21). Another factor that determines the prognosis is the **age** of metastases. For example, a younger age at the initial occurrence of distant metastases is associated with a better prognosis (21). In this context, the reason for the poorer survival rate of patients over 50 could be a lower response rate to chemotherapy, with possibly more side effects and more frequent dose reductions (22). Furthermore, **tumour biology** is considered a key prognostic factor, as the pattern of spread of neoplastic cells depends on it, among other things. The best prognosis is described for hormone receptor (HR) positive tumours (luminal A), followed by HER2neu (HER) expressing tumours, and the worst for luminal B and triple-negative tumours (TNBC) (23). These differences are attributed, among other things, to different metastasis tropisms. Luminal tumours (HR+/HER-) primarily have osseous metastases, whereas TNBCs have far more frequent visceral and cerebral metastases (24) , with the latter being associated with the poorest overall survival (OS) (23). Regardless of hormone receptor expression, HER2neu

positive tumours most frequently have visceral metastases, which are also associated with a shorter overall survival than bone metastases (23). HER2neu-expressing tumours also represent a risk factor for cerebral metastases, since the available HER2neu-targeted therapy (e.g., trastuzumab) is not permeable to the cerebrospinal fluid (25).

Another prognostic factor is fatigue, which was defined as the primary endpoint for the PRO B study. This is influenced by various parameters. For example, there are **therapy-dependent differences** in the occurrence and course of fatigue-specific symptoms between patients after chemotherapy and endocrine therapy (26) . **Sociodemographic characteristics** such as income and relationship status of those affected are also associated with the occurrence of tumour-related fatigue (27) . Several studies have also found that fatigue is associated with depression and **lack of physical activity/increased body mass index** (28, 29).

Health economic evaluation

Over the study period, the total treatment costs will be collected and evaluated using health insurance data for all participating women. The respective observation period will also be taken into account (average treatment costs per year per patient).

To account for the skewed distribution of the cost data, we will do a log-transformation and perform a linear mixed model using study centre as random intercept. Additional statistical methods of health economic analysis are carried out in the statistical analysis plan at the beginning of the project. Cost-effectiveness analyses are carried out if the intervention proves to be superior in terms of “quality adjusted life years (QALYs)”. A QALY takes into account both the life span and quality of life of interventions. QALYs are calculated on the basis of quality of life questionnaires (EQ-5D) that are completed at baseline and at various subsequent points in time during the project. A linear temporal change in quality of life is assumed between the measurement points. QALYs are calculated by calculating the area under the curve. If the intervention leads to better quality of life values at lower costs, it is considered cost-effective. If there are additional costs in the intervention group, the incremental cost-effectiveness ratio (ICER) is calculated by estimating the mean additional cost for an additional QALY. A threshold of €50,000 per extra QALY is often used in studies as a cut off point for the cost-effectiveness of an intervention. If the ICER is below this threshold, the intervention is considered cost-effective.

8 Data management

8.1 Data collection

The data reported by patients is collected using a web tool developed by NeuroSys (PatientConcept), which participating patients can access either via an app or, if necessary, via an email link. When initially registering with the web tool, an identification number is generated (Patient Concept ID, 6-digit consisting of numbers and upper- and lower-case letters), which can be used to collect the survey data pseudonymously and link it to the other data collected. Both the study centre and the study centre also have access to the web tool: This PatientCocept-Portal notifies the study centre of any abnormal values (alert), whereupon the study centre can acknowledge alerts and document any measures taken.

The study centre collects clinical, certification-relevant, process-related and study-relevant patient information via the tumour and study documentation system PRO B Doc. The entries in the PatientConcept Portal (survey data, alert acknowledgement, possible measures) are

downloaded from the study centre as an XML or JSON file at defined times (in each case after 6, 12 and 24 months). The sum of all project data (clinical tumor data, study data and survey data) from each individual center is then quality-checked via functions in OncoBox Data sets from all centers are then merged by OnkoZert using the OncoBox Research.

OnkoZert carries out further quality assurance of the data (completeness, etc.) and forwards it to the PRO B headquarters for evaluation via a secure environment (e.g. Citrix environment).

In addition, routine data is provided by the patients' respective health insurance companies and linked to the other data in the central study database via a data integration point (see Chapter 8.3). Figures 3 and 4 summarize the data flow in PRO B.

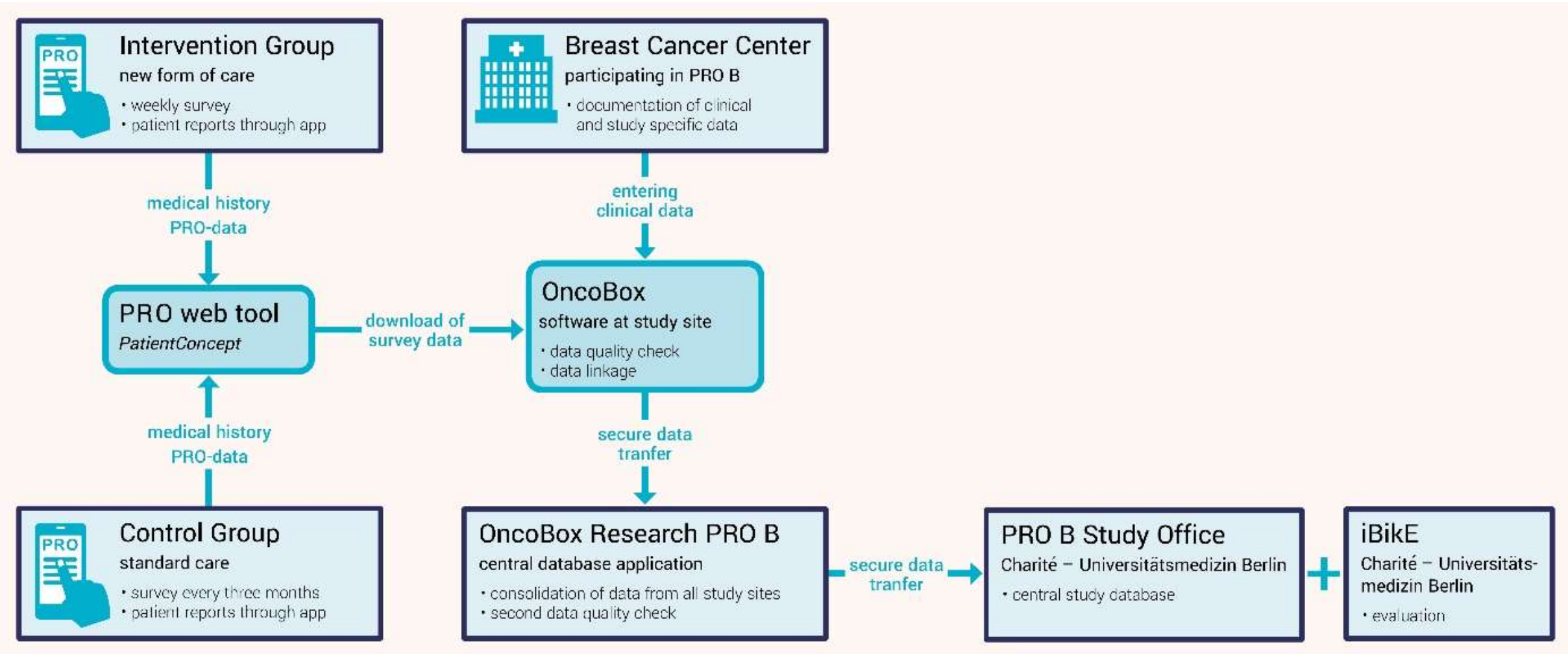

Figure 3 Data flow. *PRO* Patient-Reported Outcome, *iBiKE* Institute for Biometry and Clinical Epidemiology

8.2 Data processing, quality control, monitoring

If data are not available or not applicable during the survey, this will be noted accordingly. With regard to medications and side effects, the terminology of the international reference lists (WHO dictionary, MeDRA) will be observed.

The study documentation is monitored by the Charité-BIH Clinical Study Center. To do this, the CSC carries out a one-time in-person monitoring for each study centre, which involves a random comparison of source data and a check for completeness, consistency and plausibility. If deficiencies are identified in the study documentation, the study centre is asked to correct them. In a second in-person monitoring, the correction of the documentation deficiencies is then checked.

Before the export of the data sets, PRO B Doc checks the data for plausibility and completeness (quality assurance) via functionality of OncoBox. In addition, OnkoZert carries out a quality control of the data sets in accordance with the OncoBox criteria.

The data is evaluated by the Institute of Biometry and Clinical Epidemiology (iBiKE) at the Charité University Medicine Berlin.

8.3 Data storage and data protection

A data protection concept is available for the study, which was reviewed by the law firm Beiten Burkhardt.

As part of the PRO B study, survey data reported by patients and clinical data documented by the study centres are collected and then linked to routine data from health insurance companies.

The survey data is collected in pseudonymized form using a web tool (PatientConcept Portal) programmed by NeuroSys. The identifying data associated with a patient ID is only available to the treating study centre and is not transmitted to the central study database. Data processing and storage within the framework of the PatientConcept Portal is carried out in accordance with the data protection concept. The centres involved ensure that only authorized persons can access personal data, e.g. through access control through technical measures in secure rooms, user control through password regulations for identification and through automatic screen locking, and through access control by assigning different authorizations and differentiated access options to individual fields (confidentiality). They also ensure that personal data remains intact, complete and up-to-date during processing, e.g. by avoiding unauthorized or accidental data processing by blocking access to operating systems and/or encrypting the data and by regularly checking that the data is up-to-date (integrity); They also ensure that personal data is available in a timely manner and can be processed properly for pseudonymisation and subsequent transmission, e.g. by clearly and transparently organising the data set and granting access rights to the extent required (taking into account the requirement of confidentiality) (availability). They also ensure that personal data can be assigned to its origin at any time, e.g. by documenting the original data and its origin and traceability of the processing steps (authenticity). They also ensure that it can be determined who processed which personal data and in what way, when, e.g. by defining clear authorities and responsibilities and logging the input and further processing of the data (auditability). The procedures for processing personal data are documented completely, up to date and in such a way that they can be traced within a reasonable time.

At the end of the project, the routine data from the health insurance companies are transferred to the central study database in pseudonymised form (see Figure 4). The data is transferred via a data trusteeship office (CHA-THS) and a data integration centre: (1) The study centres send the PatientConcept ID with the insurance number to the CHA-THS. (2) The CHA-THS assigns a new health insurance pseudonym (KK pseudonym) to each PatientConcept ID and sends this together with the insurance number to the health insurance companies. (3) In conjunction with the health insurance pseudonym, the health insurance companies send the secondary data to the data integration centre. (4) The data integration centre receives the PatientConcept ID, the health insurance pseudonym and a new ID for the evaluation (evaluation ID) from the CHA-THS. (5) The health insurance data is then linked to the other study data from the PRO B headquarters. (6) Finally, the complete data set with evaluation ID is forwarded to the evaluating institute (iBiKE) after processing by the CRO.

The various data are transferred to the central study database (located at the Charité) via secure environments. The data is processed and stored in accordance with the applicable data protection regulations and data protection concepts of the Charité (see data protection concept).

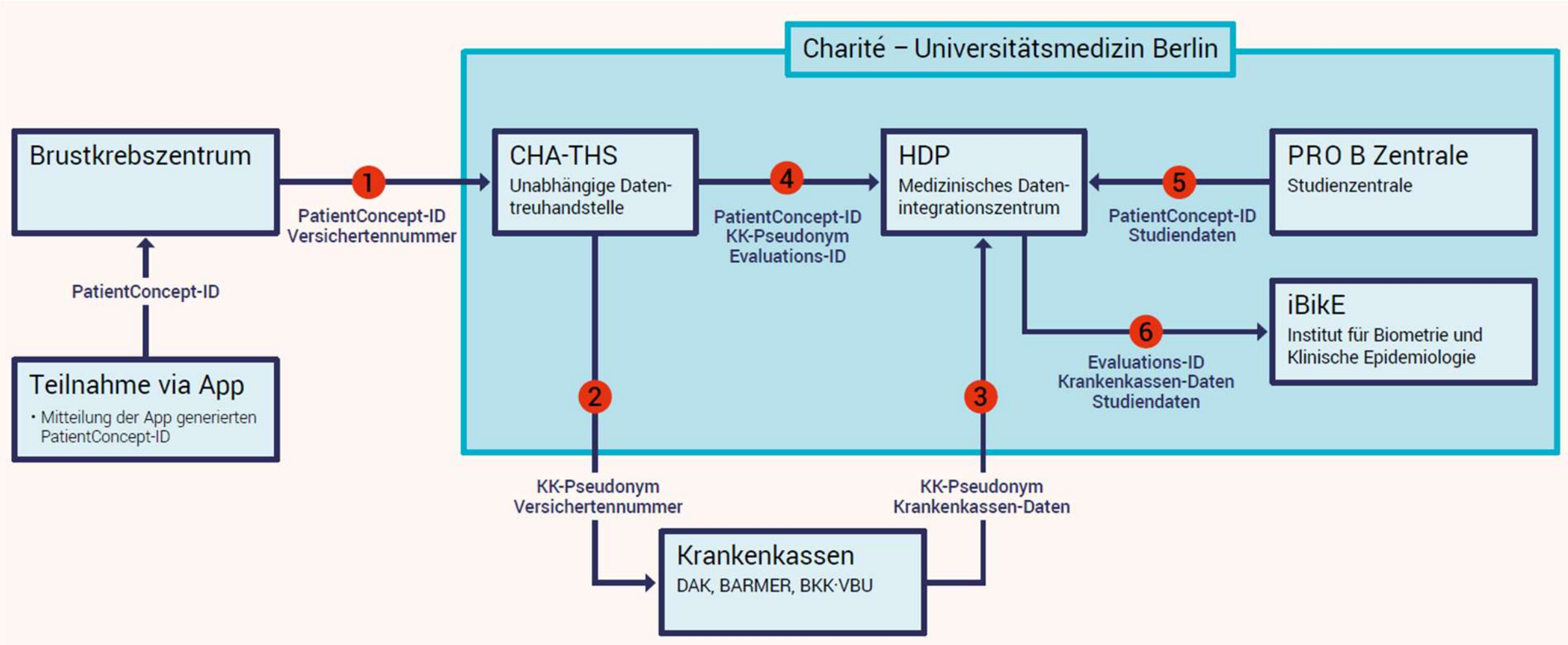

Figure 4 Transmission of health insurance data. *KK* health insurance companies

9 Ethical and legal aspects

9.1 Legal basis

Inclusion in the study is based on informed consent from the patient in accordance with the attached consent form (see Appendix 1). The intervention is carried out on the legal basis of a contract for special care in accordance with Section 140a SGBV.

9.2 Ethics committee

Before recruitment begins, the study, including patient information and consent forms, and other relevant documents, will be submitted to an independent ethics committee that will vote first. After the study centres have been determined, the documents will be submitted to the relevant local ethics committees for consultation. A positive written vote from the responsible ethics committee is a prerequisite for starting this study.

9.3 Patient information and consent form

The patient information, data protection information and consent form can be viewed in Appendix 1 and were prepared in accordance with the Ethics Committee of the Charité and the Medical Informatics Initiative (MII)[1].

9.4 Expense allowance

The participating patients will not receive any compensation for expenses.

9.5 Insurance

Study-specific insurance coverage is not provided.

[1] https://www.medizininformatik-initiative.de/de/medizininformatik-initiative-erhaelt-gruenes-licht-fuer-bundesweit-patienteneinwilligung

10 Administrative aspects

10.1 Study financing

The study is funded by the Innovation Committee of the Federal Joint Committee (grant number 01NVF19013).

10.2 Study reports

The responsible evaluating institute (iBiKE) will, together with the study management, submit an evaluation report within 6 months after the end of the study.

10.3 Study registration

Before the start of the recruitment phase, the study will be registered by the study management in the German Clinical Trials Register (DRKS) and in the study register of the US National Library of Medicine (Clinicaltrials.gov).

10.4 Publications

All information relating to the study must be treated confidentially before publication. The dissemination of the primary outcome of the study in the form of presentation(s) and/or publication(s) is reserved for the study director (PD Dr. med. Maria Margarete Karsten) or a representative chosen by her. Collaborators who have made a significant contribution to the conception and implementation of the study and/or to patient recruitment can contribute to the publication as co-authors. The selection of co-authors and the order of authorship are determined by the study director in accordance with scientific standards and in consultation with the consortium, if the selected journal is restricted. Further publications relating to the study are only permitted with the written consent and after review by the study director, whereby prevention or delay is only possible for good reasons. A draft of the abstract or manuscript must therefore be submitted to the study director at least one month before the planned submission or presentation.

At least two publications in peer-reviewed journals are planned. In addition, the study will be presented in the member magazines of the cooperating health insurance companies and the cooperating professional society (German Cancer Society). Publications in patient magazines such as Mamma Mia and mamaZone are also planned.

11 Signature of the Principal Investigator

Berlin, 22.08.2023

____

PD Dr. med. Maria Margarete Karsten

Bibliography

1. Mariotto AB, Etzioni R, Hurlbert M, Penberthy L, Mayer M. Estimation of the Number of Women Living with Metastatic Breast Cancer in the United States. Cancer Epidemiol Biomarkers Prev. 2017;26(6):809-15.

2. Robert Koch Institute. Cancer in Germany for 2015/16. Berlin2019.

3. Caswell-Jin JL, Plevritis SK, Tian L, Cadham CJ, Xu C, Stout NK, Sledge GW, Mandelblatt JS, Kurian AW. Change in Survival in Metastatic Breast Cancer with Treatment Advances: Meta-Analysis and Systematic Review. JNCI cancer spectrum. 2018;2(4):pky062.

4. Guidance for industry: patient-reported outcome measures: use in medical product development to support labeling claims: draft guidance. Health Qual Life Outcomes. 2006;4:79.

5. Basch E, Iasonos A, McDonough T, Barz A, Culkin A, Kris MG, Scher HI, Schrag D. Patient versus clinician symptom reporting using the National Cancer Institute Common Terminology Criteria for Adverse Events: results of a questionnaire-based study. The Lancet Oncology. 2006;7(11):903-9.

6. Di Maio M, Gallo C, Leighl NB, Piccirillo MC, Daniele G, Nuzzo F, Gridelli C, Gebbia V, Ciardiello F, De Placido S, Ceribelli A, Favaretto AG, de Matteis A, Feld R, Butts C, Bryce J, Signoriello S, Morabito A, Rocco G, Perrone F. Symptomatic toxicities experienced during anticancer treatment: agreement between patient and physician reporting in three randomized trials. J Clin Oncol. 2015;33(8):910-5.

7. Di Maio M, Basch E, Bryce J, Perrone F. Patient-reported outcomes in the evaluation of toxicity of anticancer treatments. Nature reviews Clinical oncology. 2016;13(5):319-25.

8. Groenvold M, Petersen MA, Idler E, Bjorner JB, Fayers PM, Mouridsen HT. Psychological distress and fatigue predicted recurrence and survival in primary breast cancer patients. Breast cancer research and treatment. 2007;105(2):209-19.

9. Müller V, Nabieva N, Häberle L, Taran FA, Hartkopf AD, Volz B, Overkamp F, Brandl AL, Kolberg HC, Hadji P, Tesch H, Ettl J, Lux MP, Lüftner D, Belleville E, Fasching PA, Janni W, Beckmann MW, Wimberger P, Hielscher C, Fehm TN, Brucker SY, Wallwiener D, Schneeweiss A, Wallwiener M. Impact of disease progression on health-related quality of life in patients with metastatic breast cancer in the PRAEGNANT breast cancer registry. The Breasts. 2018;37:154-60.

10. Park IH, Lee KS, Ro J. Effects of second and subsequent lines of chemotherapy for metastatic breast cancer. Clin Breast Cancer. 2015;15(1):e55-62.

11. Raskin W, Harle I, Hopman WM, Booth CM. Prognosis, Treatment Benefit and Goals of Care: What do Oncologists Discuss with Patients who have Incurable Cancer? Clin Oncol (R Coll Radiol). 2016;28(3):209-14.

12. Schwartz KL, Simon MS, Bylsma LC, Ruterbusch JJ, Beebe-Dimmer JL, Schultz NM, Flanders SC, Barlev A, Fryzek JP, Quek RGW. Clinical and economic burden associated with stage III to IV triple-negative breast cancer: A SEER-Medicare historical cohort study in elderly women in the United States. Cancer. 2018;124(10):2104-14.

13. Sorensen SV, Goh JW, Pan F, Chen C, Yardley D, Martín M, Knopf K, Benedict A, Giorgetti C, Iyer S. Incidence-based cost-of-illness model for metastatic breast cancer in the United States. Int J Technol Assess Health Care. 2012;28(1):12-21.

14. Basch E, Deal AM, Kris MG, Scher HI, Hudis CA, Sabbatini P, Rogak L, Bennett AV, Dueck AC, Atkinson TM, Chou JF, Dulko D, Sit L, Barz A, Novotny P, Fruscione M, Sloan JA, Schrag D. Symptom Monitoring With Patient-Reported Outcomes During Routine Cancer Treatment: A Randomized Controlled Trial. J Clin Oncol. 2016;34(6):557-65.

15. Basch E, Deal AM, Dueck AC, Scher HI, Kris MG, Hudis C, Schrag D. Overall Survival Results of a Trial Assessing Patient-Reported Outcomes for Symptom Monitoring During Routine Cancer Treatment. JAMA. 2017;318(2):197-8.

16. Denis F, Basch E, Septans AL, Bennouna J, Urban T, Dueck AC, Letellier C. Two-Year Survival Comparing Web-Based Symptom Monitoring vs Routine Surveillance Following Treatment for Lung Cancer. JAMA. 2019;321(3):306-7.

17. Pocock SJ, Simon R. Sequential treatment assignment with balancing for prognostic factors in the controlled clinical trial. Biometrics. 1975;31(1):103-15.

18. Cocks K, King MT, Velikova G, de Castro G, Jr., Martyn St-James M, Fayers PM, Brown JM. Evidence-based guidelines for interpreting change scores for the European Organization for the Research and Treatment of Cancer Quality of Life Questionnaire Core 30. Eur J Cancer. 2012;48(11):1713-21.

19. Cortes J, Hudgens S, Twelves C, Perez EA, Awada A, Yelle L, McCutcheon S, Kaufman PA, Forsythe A, Velikova G. Health-related quality of life in patients with locally advanced or metastatic breast cancer treated with eribulin mesylate or capecitabine in an open-label randomized phase 3 trial. Breast cancer research and treatment. 2015;154(3):509-20.

20. Borm GF, Fransen J, Lemmens WA. A simple sample size formula for analysis of covariance in randomized clinical trials. J Clin Epidemiol. 2007;60(12):1234-8.

21. Dawood S, Broglio K, Ensor J, Hortobagyi GN, Giordano SH. Survival differences among women with de novo stage IV and relapsed breast cancer. Annals of oncology: official journal of the European Society for Medical Oncology. 2010;21(11):2169-74.

22. Largillier R, Ferrero JM, Doyen J, Barriere J, Namer M, Mari V, Courdi A, Hannoun-Levi JM, Ettore F, Birtwisle-Peyrottes I, Balu-Maestro C, Marcy PY, Raoust I, Lallement M, Chamorey E. Prognostic factors in 1,038 women with metastatic breast cancer. Annals of oncology: official journal of the European Society for Medical Oncology. 2008;19(12):2012-9.

23. Kast K, Link T, Friedrich K, Petzold A, Niedostatek A, Schoffer O, Werner C, Klug SJ, Werner A, Gatzweiler A, Richter B, Baretton G, Wimberger P. Impact of breast cancer subtypes and patterns of metastasis on outcome. Breast cancer research and treatment. 2015;150(3):621-9.

24. Soni A, Ren Z, Hameed O, Chanda D, Morgan CJ, Siegal GP, Wei S. Breast cancer subtypes predispose the site of distant metastases. American Journal of Clinical Pathology. 2015;143(4):471-8.

25. Lin NU, Bellon JR, Winer EP. CNS Metastases in Breast Cancer. Journal of Clinical Oncology. 2004;22(17):3608-17.

26. Bower JE, Wiley J, Petersen L, Irwin MR, Cole SW, Ganz PA. Fatigue after breast cancer treatment: Biobehavioral predictors of fatigue trajectories. Health psychology : official journal of the Division of Health Psychology, American Psychological Association. 2018;37(11):1025-34.

27. Bower JE, Ganz PA, Desmond KA, Rowland JH, Meyerowitz BE, Belin TR. Fatigue in breast cancer survivors: occurrence, correlates, and impact on quality of life. J Clin Oncol. 2000;18(4):743-53.

28. Bower JE. Cancer-related fatigue--mechanisms, risk factors, and treatments. Nature reviews Clinical oncology. 2014;11(10):597-609.

29. Andrykowski MA, Donovan KA, Laronga C, Jacobsen PB. Prevalence, predictors, and characteristics of off-treatment fatigue in breast cancer survivors. Cancer. 2010;116(24):5740-8.

Abbreviations

CRO: Clinical Research Organisation;

CSC: Clinical Study Centre;

DKG: German Cancer Society (Deutsche Krebsgesellschaft);

ECOG: Eastern Cooperative Oncology Group;

EORTC: European Organisation for Research and Treatment of Cancer;

GCP: Good Clinical Practice;

HER: Human epidermal growth factor receptor 2;

HR: Hormone receptor;

HRQoL: Health-related quality of life;

iBiKE: Institute of Biometry and Clinical Epidemiology (Institut für Biometrie und Klinische Epidemiologie);

ICER: Incremental cost-effectiveness ratio;

ICMJE: International Committee of Medical Journal Editors;

MAR: Missing at random;

MBC: Metastatic breast cancer;

PRO: Patient-reported outcome;

QALY: Quality-adjusted life year;

QoL: Quality of life;

SAP: Statistical analysis plan

Appendices

1. Patient information, data protection information and consent form
2. Data protection concept

Study protocol

PRO B – Patient Reported Outcomes in Breast Diseases

New Approaches to the Care of Metastatic Breast Cancer

Short title: PRO B

Indication: metastatic breast cancer

Intervention: Patient-Reported Outcomes (PROs)

DRKS-ID/ ID US National Library of Medicine: To be added

Funding: Innovation Committee at the G-BA

Table of contents

Synopsis.......... 1
Responsibilities 3
1 Rationale.......... 4
1.1 Scientific Background.......... 4
1.2 Justification for the study.......... 5
1.3 Benefit-risk assessment 6
2 Objectives of the study.......... 6
2.1 Primary objective and primary endpoint.......... 6
2.2 Secondary objective and secondary endpoints.......... 7
3 Study design and schedule 7
3.1 Study design 7
3.2 Schedule 8
4 Recruitment 8
4.1 Number of cases 8
4.2 Study centres 9
4.3 Inclusion criteria 9
4.4 Exclusion criteria 9
4.5 Termination criteria..........10
5 Intervention 10
5.1 Description of the intervention 10
5.2 Risks of the intervention 11
5.3 Randomization 11
5.4 Blinding 11
5.5 Possible confounders 12
6 Course of study..........13
6.1 Description of the course of study 13
6.2 Planned treatment after study end..........14
7 Statistical approach..........14
7.1 Sample size calculation..........14
7.2 Variables 15
7.3 Pretest..........16
7.4 Statistical methods 16
8 Data management 19
8.1 Data collection..........19
8.2 Data processing, quality control, monitoring 20
8.3 Data storage and data protection 21

9 Ethical and legal aspects 23
9.1 Legal basis 23
9.2 Ethics committee 23
9.3 Patient information and consent form 23
9.4 Expense allowance 23
9.5 Insurance 23
10 Administrative aspects 23
10.1 Study financing 23
10.2 Study reports 24
10.3 Study registration 24
10.4 Publications 24
11 Signature of the Principal Investigator 24
Bibliography 25
Abbreviations 28
Appendices 29

Synopsis

Title	**PRO B** – New approaches to the care of metastatic breast cancer
Indication	Metastatic breast cancer
Study objectives	Evaluation of the effects of intensified patient-reported outcome (PRO) monitoring on quality of life, overall survival and treatment costs specifically for patients with metastatic breast cancer<br><br>Primary endpoint:<br><br>Fatigue (EORTC QLQ-C30, QLQ-FA12, EORTC CAT) after 12 and possibly 24 months<br><br>Secondary endpoints:<br><br>Secondary objectives are to improve overall survival and reduce unplanned medical consultations, hospitalisations and emergency room stays after 6, 12 and, if applicable, 24 months
Study design	PRO B is a prospective, multicentre, randomised two-arm intervention study to implement and evaluate PRO measurements in patients with metastatic breast cancer.<br><br>Intervention arm: weekly PRO survey with contact and, if necessary, measures by the treating team if the values deteriorate<br><br>Control arm: PRO survey every three months without contact
Sample	Inclusion criteria:<br><br>- female<br>- statutory health insurance (BARMER, DAK Gesundheit, BKK · VBU)<br>- diagnosis of metastatic breast cancer<br>- treatment at a participating breast cancer centre<br>- completion of the 18th year of life and ability to consent<br>- receiving drug treatment for metastatic breast cancer<br>- life expectancy at study inclusion of more than 3 months<br>- ECOG 0-2<br>- willingness to participate in a weekly, online-based PRO survey<br>- access to the Internet (smartphone, tablet or email)

	Exclusion criteria: no active tumor therapy (comfort care)<br>Termination criteria: patient withdraws consent to participate in the study
Sample size	500 subjects per study arm
Project duration	36 months
Statistical approach	- primary hypothesis: ANCOVA model<br>- secondary hypotheses: e.g. time-event models, ANCOVA, logistic regression, Cox regression<br>- subgroup analyses<br>- health economic analyses
Number of study centres	Up to 40 breast cancer centres
Study financing	Innovation Committee of the Federal Joint Committee (grant number: 01NVF19013)

Responsibilities

**Principal Investigator**	**Dr. med. Maria Margarete Karsten**<br>Clinic for Gynecology with Breast Centre<br>Charité Campus Mitte<br>Charité – Universitätsmedizin Berlin<br><br>Charitéplatz 1<br>10117 Berlin<br><br>+49 (0)30 450 664 279<br>maria-margrete.karsten@charite.de
**Evaluation**	**PD Dr. rer nat. phil. Ulrike Grittner**<br>Institute for Biometry and Clinical Epidemiology<br>Charité - Universitätsmedizin Berlin<br>Charité Campus Mitte<br><br>Charitéplatz 1<br>10117 Berlin<br><br>+49 (0)30 450 562 174<br>ulrike.grittner@charite.de
**Psychometric testing**	**Prof. Dr. med. Matthias Rose**<br>Medical Clinic with Psychosomatics<br>Charité - Universitätsmedizin Berlin<br><br>Charitéplatz 1<br>10117 Berlin<br><br>+49 (0)30 450 553 001<br>matthias.rose@charite.de
**Technical implementation**	**Dr. med. Julia Ferencz**<br>OnkoZert GmbH<br><br>Gartenstraße 24<br>89231 Neu-Ulm<br><br>j.ferencz@onkozert.de
**Dissemination and support in recruiting study centres**	**Clara Breidenbach**<br>German Cancer Society<br><br>Kuno-Fischer-Straße 8<br>14057 Berlin<br><br>+49 (0)30 322 932 934<br>breidenbach@krebsgesellschaft.de
**Contract Special Care and Routine Data**	BKK · VBU<br>BARMER<br>DAK Gesundheit
**Study centres**	breast cancer centres
**Project funding**	Innovation Committee at the Federal Joint Committee

1 Rationale

1.1 Scientific Background

Breast cancer is the most common cancer and cause of cancer-related death in women worldwide. Despite all the advances in research and clinical practice, up to 30% of those affected still develop metastases (1). According to the Centre for Cancer Registry Data of the Robert Koch Institute, more than 18,000 patients die each year in Germany alone as a result of metastatic breast cancer (2). When metastases appear, not only the living conditions of the patients change fundamentally, but also the therapeutic approach. The focus of treatment is no longer on curing the disease, but rather on controlling the tumour and the associated prolongation of survival while simultaneously controlling the side effects of therapy. The goal is to maintain the best possible quality of life for as long as possible.

The overall survival of patients with metastatic breast cancer has improved significantly over the last few decades thanks to more modern treatment options, and at 38 months in 2010 was already 17 months above the median overall survival in 1990 (3). However, life expectancy in the case of metastatic breast cancer depends largely on the biological properties of the underlying tumour. In a meta-analysis, Caswell-Jin et al. showed a median survival of 57 months for patients with hormone receptor-positive tumours compared to a survival time of only 33 months for triple-negative breast cancer (3). With the aim of extending the period of survival, most patients undergo systemic tumour therapy during this time. Unlike in the adjuvant situation, in which treatment decisions can be relatively easily based on the standards of the professional societies, in the metastatic situation they must be made on a much more individual basis. Taking into account the medical history, current complaints and, above all, the patient's wishes, the decision-making process is time-consuming and must be questioned again and again due to the progressive course of the disease. At this point, information collected via so-called patient-reported outcomes (PROs) is helpful in making the best possible treatment decisions. PROs enable the assessment of health-related aspects (such as symptoms of disease, psychosocial stress and quality of life) from the patient's perspective without intervening interpretation by another person (e.g. doctor) (4). They thus contribute to improving

- the early detection of possible disease progression,
- the assessment of therapy-associated toxicity and
- the detection of a deterioration in the so-called Health-related Quality of Life (HRQoL).

The particular challenge of assessing the severity of certain disease symptoms or medication side effects is often underestimated by the attending physicians (5-7). This particularly applies to non-specific symptoms such as constipation, loss of appetite and fatigue, although the latter

can often be the first clinical indicator of disease progression (8). In this context, PRO measurements are helpful because they enable treating physicians to obtain a very differentiated picture of their patients' state of health within a very short time. Especially in the metastatic situation, in which patients should spend as little time as possible in the clinic and therapies are therefore usually carried out on an outpatient basis, regular PRO measurements enable real-time monitoring of symptoms and side effects as well as timely interventions by treating physicians. Müller et al. report that even a slight deterioration in HRQoL in patients with metastatic breast cancer is associated with disease progression (9). The possibility of using PRO measurements to detect such deterioration and thus, if necessary, disease progression at an early stage can spare those affected from further cycles of tumour therapy that is no longer effective.

1.2 Justification for the study

With an average overall survival of 38 months after metastasis and 18,570 women dying of breast cancer each year (2,3), it can be assumed that there are currently more than 60,000 patients with metastatic breast cancer in Germany. Treating these seriously ill women represents a challenge for the entire clinical team from a medical, psychosocial and economic perspective. There are no curative treatment options, so disease progression is inevitable. In addition, with every change in the treatment regimen in refractory disease, the likelihood of response decreases - in previously treated patients, it can be less than 15% (10). From a psychosocial perspective, considerable care and preventive measures are necessary due to the high level of disease-related psychological and emotional stress (11). In addition, the cost of treating metastatic breast cancer is currently estimated at around 100,000 US dollars per patient per year (12, 13). The combination of the high burden of disease on those affected, the high burden on the treatment team due to the complexity of the disease and the equally high financial requirements make innovations in the care of patients with metastatic breast cancer urgently necessary.

In a ground-breaking monocentric study of 766 patients with metastatic tumour disease, Basch et al. were able to show that intensified PRO monitoring could reduce the rates of unplanned emergency room stays and hospitalisations, but above all also significantly improve the quality of life and even the overall survival of the study participants (HR: 0.83; 95% CI: 0.70-0.99; p=0.04) (14, 15). Shortly thereafter, the benefits of intensified PRO monitoring were confirmed in another study on metastatic lung cancer patients. In this study, Denis et al. showed that the overall survival of patients treated with PROs could be extended by about eight months compared to the control group (HR: 0.50; 95% CI: 0.31-0.81; p=0.005) (16).

To our knowledge, there have been no studies to date on the effects of intensified PRO monitoring on quality of life, overall survival and treatment costs specifically for patients with

metastatic breast cancer. PRO B is therefore intended to examine whether the results of the previously cited authors can be transferred to the usual care of this specific patient group in Germany. In order to enable simple and cost-effective transfer to standard care in the event of positive results, existing IT elements of the certification infrastructure of the German Cancer Society (DKG) and OnkoZert are used. PRO B therefore expands a long-established, comprehensive and cross-centre standardised collection of health data in order to enable a better assessment of the course of the disease of metastatic breast cancer and the associated effects on the quality of life of the affected patients.

1.3 Benefit-risk assessment

A study-specific health risk is not to be expected, as the study does not prescribe any specific treatment that deviates from the usual clinical routine. The assumed benefit for the participating patients consists in individual monitoring of the quality of life and, on this basis, measures initiated in the event of poor quality of life (personal benefit). If the evaluation of the intervention is positive, the benefit for future patients (group benefit) consists in an individualized and patient-centred form of care.

2 Objectives of the study

With PRO B, a new, individualized and patient-centred form of care is created and evaluated, taking into account the available resources. The intervention described under point 5 is carried out during the one-year recruitment period and the following one-year intervention period. Accordingly, the intervention duration is at least 12 and maximally 24 months. The outcomes listed below (after 6 and 12 months) are available at the end of the recruitment and intervention period for *all* included subjects (minus dropouts). However, they are also analysed for the later measurement points (up to a maximum of 24 months after inclusion) with a smaller n in order to be able to depict the PRO course for metastatic breast cancer patients over the longest possible period.

2.1 Primary objective and primary endpoint

The study aims to reduce disease-related morbidity and improve the quality of life of patients with metastatic breast cancer. The primary endpoint is therefore tumour-related fatigue, defined using the fatigue scales of the Quality of Life Questionnaire Core Module of the European Organisation for Research and Treatment of Cancer and the supplementary fatigue module (EORTC QLQ-C30, EORTC QLQ-FA12) as well as study-specific items from the EORTC CAT item database 12 months after inclusion.

2.2 Secondary objective and secondary endpoints

Secondary objectives are to improve overall survival and to reduce unplanned medical consultations, hospitalisations and emergency room stays. The following secondary endpoints were defined:

1. Number of hospital and emergency room stays after six and 12 months
2. Physical function after six and 12 months, using the Physical Functioning Score of the EORTC QLQ-C30
3. Fatigue after six months, using the fatigue scores of the EORTC QLQ-C30 and EORTC QLQ-FA12, supplementary items from EORTC CAT
4. Overall survival in visceral metastases after 12 months
5. Overall survival in triple-negative breast cancer after 12 months
6. Progression-free survival after 12 months
7. Health-related quality of life after 12 months, using the EORTC QLQ-C30 HRQoL sum score
8. number of therapy changes

3 Study design and schedule

3.1 Study design

PRO B is a prospective, multicentre, randomised two-arm intervention study to implement and evaluate PRO measurements in patients with metastatic breast cancer.

After inclusion in the study, the patients are randomly assigned to either the intervention arm or the control arm (see Figure 1). Depending on the randomisation, the patients are monitored weekly (intervention arm) or every three months (control arm) with a PRO measurement and in the intervention arm they are contacted by the treating physicians if the values deteriorate (see 5.1 Description of the intervention).

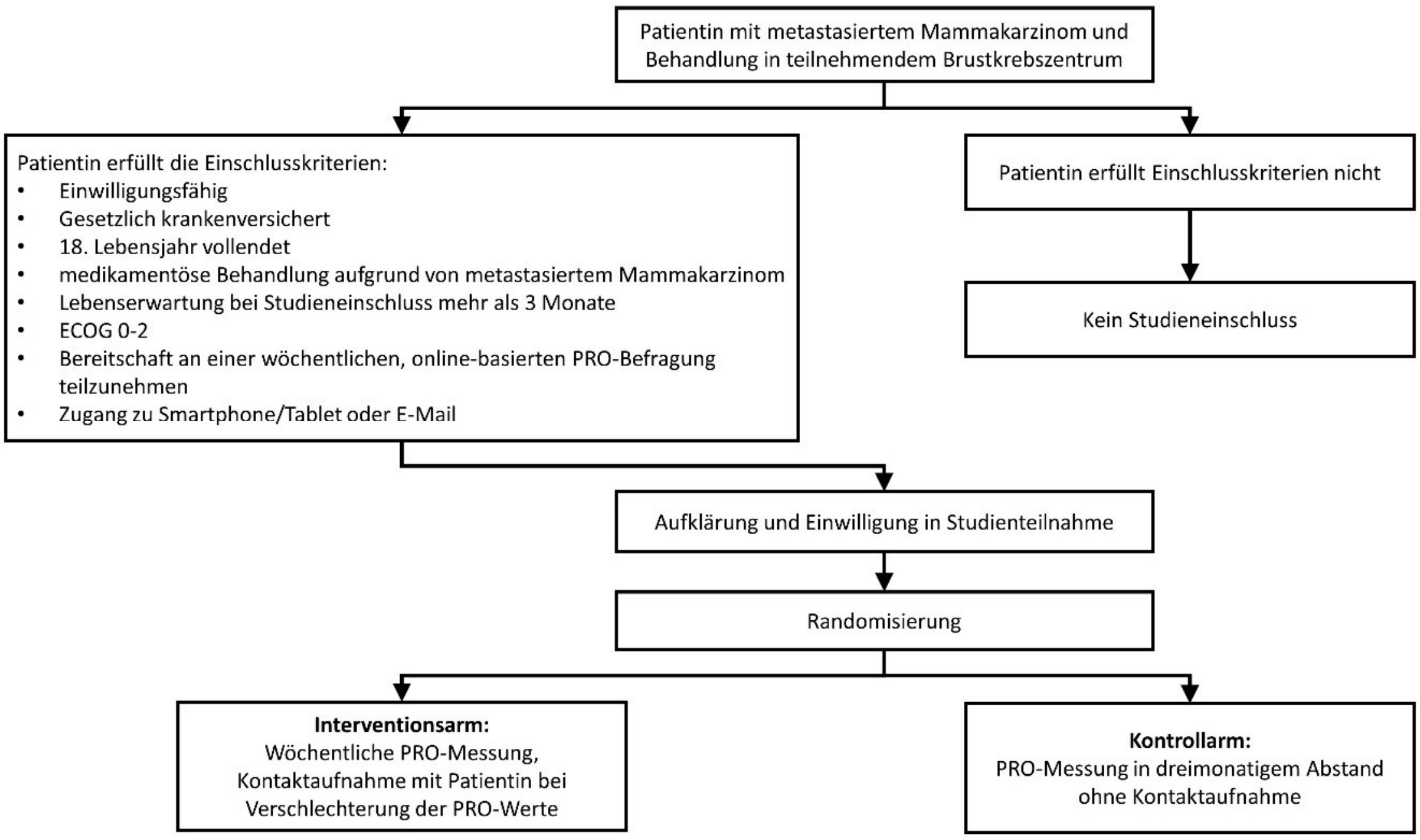

Figure 1 Study design

3.2 Schedule

The total duration of the research project is 36 months. Patient recruitment is scheduled for April 2021 and will last one year.

**total duration**	36 months
**preparation phase and study initiation**	October 2020 – March 2021
**recruitment of patients**	April 2021 – March 2022
**follow-up and monitoring**	April 2022 – March 2023
**data analysis and dissemination**	April 2023 – September 2023

4 Recruitment

4.1 Number of cases

The target number of cases is 500 patients per treatment arm. The case number calculation is shown in chapter 7.1.

Reducing drop-outs/incentivizing

In order to keep the drop-out rate in the control group as low as possible, patients receive a printout or a file of their survey data, i.e. their PRO course, after the end of the observation

period, and patients are personally contacted if they do not respond to the three-monthly survey. Unlike in the intervention group, contact is made here only as a reminder to answer the PRO survey. For this purpose, the participating centres are provided with individualised letters for each study patient with the request that they forward them to the patient. Alternatively, the participating centre also has the option of contacting the patient by telephone if the questionnaire is not answered and asking them to fill it out as part of their participation in the study. The PRO course can subsequently serve as a basis for discussion with the treating physicians and reveal any unnoticed abnormalities. By contacting the study centres, the patients in the control group are also made aware that they are an essential part of the study and that they are receiving no less attention from their treating physicians.

Following the feedback of the Charité breast cancer self-help group, the intensified digital monitoring of the PRO course is considered sufficient to incentivise the intervention group.

4.2 Study centres

A total of up to 40 breast cancer centres spread across the Federal Republic of Germany will be recruited as study centres. When recruiting the study centres, the number of cases and the proportion of patients with insurance with the cooperating health insurance companies BKK · VBU, BARMER, DAK Gesundheit will be taken into account. In addition, during recruitment, care will be taken to ensure that study centres with different degrees of urbanity, sponsorship and teaching status are included.

4.3 Inclusion criteria

Women can be included

- with metastatic breast cancer,
- who are treated in a breast cancer centre participating in the study,
- who have statutory health insurance (BARMER, DAK Gesundheit, BKK · VBU),
- who are capable of giving consent,
- who have reached their 18th year of age,
- who are receiving drug treatment for metastatic breast cancer,
- who have a life expectancy of more than 3 months at study inclusion,
- who are classified as ECOG 0-2,
- who are willing to participate in a weekly, online-based PRO survey,
- and who have access to the Internet (smartphone, tablet or email).

4.4 Exclusion criteria

Patients who are not receiving active tumour therapy (comfort care) cannot participate in the study.

4.5 Termination criteria

Participation in the study will be terminated if the patient withdraws consent.

5 Intervention

5.1 Description of the intervention

Patients in the new form of care receive a request once a week (always on the same day of the week) via app or email to complete the PRO survey. Analogous to the target parameters of the study, the intervention concept is based on the operationalisation of health-related quality of life of the EORTC QLQ-C30 and QLQ-FA12. The QLQ-C30 includes five functional dimensions (Physical Function, Role Function, Emotional Function, Cognitive Function, Social Function) and 9 symptom scales (Fatigue, Nausea, Pain, Dyspnoea, Insomnia, Appetite loss, Constipation, Diarrhoea, Financial Difficulties). In addition, the FA12 defines three aspects of fatigue (Physical Fatigue, Emotional Fatigue = Depression, Cognitive Fatigue = Cognitive Function) and the resulting stress (Daily activities, Social Sequalae). The named instruments are supplemented by study-specific items from the EORTC CAT item database.

If the PRO scores deteriorate in the individual longitudinal course and/or in comparison to previously defined limit values, an alarm is generated, which is displayed to the treating study centre and the PRO B study centre. Clinical relevance is calculated based on published information on the EORTC QLQ-C30 (e.g. MCID) and based on probabilities of actual changes in the latent variables. In order to detect an aggregation of several clinically irrelevant deteriorations over several weeks, a comparison with the measured value from four weeks ago should also be made. An alarm must be acknowledged and documented by the cooperating study centres within 48 hours (working days). The format of the feedback to the treating centres is developed jointly with them. In any case, it explicitly includes the domains and the extent of the change. The alarm is followed by the respective centre contacting the patient to specify the deterioration in her values and to intervene if necessary. The intervention or measure by the study centre following the contact is not specified by PRO B. The type of measures is therefore left to the decision of the treating physicians.

In this respect, the study does not test a specific therapeutic intervention for breast cancer patients, but rather a timely, individualised contact and possible intervention of various forms in the event of a deterioration in subjective quality of life parameters. The effect of the intervention results primarily from the timely, individualised contact of the treating physicians due to alarming or alarmingly deteriorating patient-oriented, quality of life-related parameters.

This new form of care applies to all patients in the intervention arm for the entire duration of the study (24 months), but at least for 12 months.

5.2 Risks of the intervention

The intervention consists of entering health-related data into an app. Health risks are considered to be low.

The risk factors for the success of the intervention are the lack of reaction/intervention to a deterioration in the survey values by the treating team. To ensure that contact with the patient is made correctly and, above all, in a timely manner, an alarm system is integrated into the survey module that uses a stored logarithm to send an alarm to the treating breast cancer centre and the study centre when the values deteriorate. If the alarm is not acknowledged by the breast cancer centre within 48 hours (working days) and the intervention triggered by this is not documented, the study centre will contact the breast cancer centre by telephone.

5.3 Randomization

Randomization is carried out according to the following strata:

- centres (n=40)
- types of metastasis (4 groups)
    - exclusively osseous metastases
    - visceral metastases
    - brain metastases
    - multiple metastases
- histological findings (4 groups)
    - luminal (HR+/HER-)
    - triple positive (HR+/HER+)
    - HR-/HER+
    - triple negative

Due to the high number of strata, imbalances in the stratification variables between the intervention and control groups can arise in a simple randomisation. In order to keep these as low as possible, adaptive randomisation is carried out using the minimisation method of Pocock and Simon (17). Randomisation takes place after informed consent, download of the application and baseline survey (including sociodemographic information and comorbidities, see chapter 5.5).

5.4 Blinding

There is no blinding.

5.5 Possible confounders

As possible confounders, data on the following socioeconomic and sociodemographic parameters will be collected once during the initial survey:

- marital status
- number of people in the household
- relatives in need of care/minor children (≤14 years) in the household
- educational qualification/professional qualification
- employment status
- income
- migration background.

In addition to the breast cancer-specific anamnesis (family history of cancer), the survey via the smartphone application also enables the assessment of any previous illnesses. We suspect that the following comorbidities have a significant influence on the primary endpoint of the study (fatigue). They must therefore be taken into account both in the interpretation of the measured values in the ongoing project and in the final evaluation (e.g. as the Charlson comorbidity index (underlined illnesses)):

- cardiac and cardiovascular pre-existing conditions (arterial hypertension, coronary heart disease, angina pectoris, myocardial infarction, heart failure, atrial fibrillation, valvular disease)
- previous vascular diseases (deep vein thrombosis, peripheral arterial occlusive disease, pulmonary embolism)
- cerebrovascular diseases (TIA, stroke)
- previous haematological diseases (anaemia)
- previous pulmonary diseases (bronchial asthma, COPD, sleep apnoea)
- endocrine/metabolic pre-existing diseases (hyperlipidaemia, diabetes mellitus (requiring treatment with and without end-organ damage), hypothyroidism, obesity)
- previous rheumatological diseases (polymyalgia rheumatica, lupus erythematosus, rheumatoid arthritis, polymyositis)
- previous mental illnesses (depression, anxiety disorder)
- neurological pre-existing conditions (migraines, seizures, balance disorders, neuropathy, hemiplegia, dementia)
- previous gastrointestinal diseases (gastrointestinal ulcers, chronic inflammatory bowel disease, diverticulitis, reflux, irritable bowel syndrome, mild liver disease (liver cirrhosis without portal hypertension), severe liver disease (liver cirrhosis with portal hypertension, oesophageal variceal bleeding))

- previous gynaecological diseases (infertility, abortion, dysmenorrhea or hypermenorrhoea, endometriosis, ovarian cysts)
- previous urogenital diseases (nephro- or urolithiasis, renal insufficiency, dialysis requirement, micturition problems, urinary tract infections, incontinence)
- infectious diseases (HIV, AIDS)
- previous oncological diseases (other tumour diseases (with and without metastases), leukaemia, lymphoma)
- previous operations
- drug history (alcohol, nicotine)

6 Course of study

6.1 Description of the course of study

Patients in the breast cancer centre who meet the inclusion criteria are informed about the study by the investigators. Provided informed consent is given, the patient is included in the study population. The app for the PRO surveys is then installed on the study participant's smartphone/tablet or the patients receive an email link that can be used to complete the survey. The study participant is then randomised into a study arm.

In the intervention arm, the patient is then asked weekly via the app to take part in the PRO survey for at least 12 months and maximally 24 months. If the values reported by the patient via the app deteriorate, an alarm is sent to the breast cancer centre via a web application, which then contacts the patient and coordinates possible measures. If the patient misses a weekly PRO survey, the breast cancer centre is also informed via the web application and can contact the patient. Alarms are acknowledged by the breast cancer centre in the web application and contact and, if applicable, measures are documented. The PRO-B study centre also has access to the web application and can check alarms and the response of the breast cancer centres. If a breast cancer centre has not acknowledged an alarm within 48 hours, the study centre contacts the breast cancer centre.

A patient in the control arm is asked by the app every 3 months to take part in the PRO survey. The answers are also sent electronically to the web application immediately. For the control group, no alarm is sent if the PRO values deteriorate and therefore no contact is made. If the survey is not answered, the study centre sends a written or telephone reminder.

After the patient has given their consent, the breast cancer centre documents clinical data in the tumour documentation system during treatment, as required by the certification. Additional clinical information relevant to the study that is not included in the certification requirements is documented by the breast cancer centre in an Electronic Case Report Form (eCRF) (study

documentation software TuDoc from OnkoZert). In addition, routine data is collected by the patient's health insurance company. Figure 2 provides an overview of the individual study process for each patient.

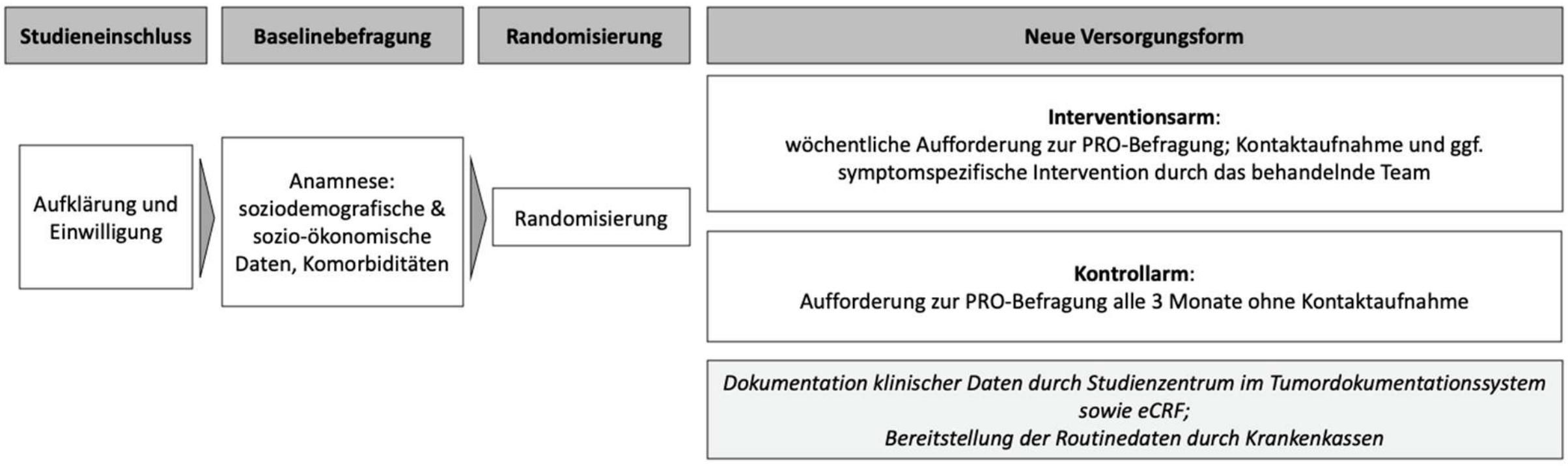

Figure 2 Individual study schedule

6.2 Planned treatment after study end

In the event of positive results, the newly created form of care can be incorporated into standard care with manageable effort due to its special structure. In this context, the patients would not receive any specific treatment that deviated from the usual clinical routine after the study, as well as during the study, but would continue to be monitored electronically with regard to their symptoms and quality of life. The care that is evaluated during PRO B could therefore be continued in a very similar form after the end of the study and expanded to other patient groups. This would also make it possible, for example, to implement and evaluate PRO-supported treatment and aftercare for breast cancer patients in the adjuvant situation.

7 Statistical approach

7.1 Sample size calculation

Cocks et al. created guidelines for the interpretation of longitudinal changes on the fatigue scale of the EORTC QLQ-C30, according to which values of an average improvement of 4 points or more are to be considered a small improvement and values of 9 points or more are to be considered a moderate improvement (18). However, PRO B is about a comparison between the treatment and control groups. The effect estimates follow the information provided by Cocks et al., but due to the two-group comparison, much larger variances are to be expected, which is why a conservative estimate of a standardised effect size of around 0.2 is

to be achieved (mean difference, for example, 5 points. Common standard deviation 25.2 points). Such a variance is comparable to the information provided by Cortes et al. 2015 (19).

Since the analysis of certain subgroups, for example patients with visceral metastases, is particularly relevant, the aim is to have a sample size that is sufficiently large for subgroup analyses. The recruitment of 500 patients per group is considered realistic. With an expected drop-out rate of 20%, this results in 400 complete data sets per treatment arm. If there is a minimal clinically relevant effect of 5 points mean difference on the fatigue scale of the EORTC QLQ-C30, as reported by Cocks et al. (18) (with a common standard deviation of 25), a two-sided t-test with a significance level of 5% has a power of 80.65% to detect a difference in the fatigue score between the intervention and treatment groups with 400 patients per group. This calculation was carried out with nQuery (version 8.3.1.0). The number of cases is therefore sufficient for the detection of small effect sizes (0.2 or greater) and takes into account the number of relevant subgroups in the patient population.

7.2 Variables

The table below lists the primary outcome and the secondary outcomes of the study. Additional variables that are relevant for randomisation or as possible confounders are defined for the baseline survey and the study documentation system (eCRF in TuDoc).

Table 2 Overview of primary outcomes and secondary outcomes

**Variable**	**Data source**	**Time of collection**			
		**Baseline**	**Weekly in intervention group; every 3 months in control group**	**6 months**	**12 months**
**Primary outcome**					
Fatigue Score (EORTC QLQ-C30, EORTC QLQ-FA12, EORTC CAT)	questionnaire	x	x	x	x
**Secondary Outcomes**					
Physical functioning score (EORTC QLQ-C30)	questionnaire		x	x	x
Health-related Quality of Life sum score (EORTC QLQ-C30)	questionnaire	x	x		x

Number of hospital and emergency room stays	eCRF/ health insurance data			x	x
Number of therapy changes	eCRF/ health insurance data				x
Overall survival in visceral metastasis	tumour documentation /eCRF				x
Overall survival in triple negative breast cancer	tumour documentation /eCRF				x
Progression-free survival	tumour documentation /eCRF				x

7.3 Pretest

The pretest will include the measurement instruments selected for follow-up. In some cases, think-aloud techniques will be used, and in some cases a short evaluation questionnaire will be attached to the surveys, which will enable an evaluation of the survey content and the stress of answering it. Respondents for the pretest will be recruited through self-help groups (e.g. the Charité breast cancer self-help group).

7.4 Statistical methods

Statistical analysis of the hypotheses

The data is evaluated by the Institute of Biometry and Clinical Epidemiology (iBiKE) at the Charité Universitätsmedizin Berlin.

Analysis of the primary hypothesis

The primary question will be investigated using an intention-to-treat analysis for all randomised individuals. All randomisation stratification variables will be considered as covariates in all statistical models.

A two-sided t-test was used to calculate the sample size, although an ANCOVA model is to be used in the evaluation. It was shown that this is a conservative approach for estimating the sample size of ANCOVA models, since an ANCOVA model with (1 - 2p)*n observations has at least the same power as a t-test with n observations (20), where p is the variance inflation

factor (correlation between the *baseline* measurement and the *follow-up* measurement). In the worst case of p = 0, this leads to a required number of cases equal to the t -test. The analysis is carried out in the ANCOVA model with the baseline measurement and types of metastasis and histology as covariates (analogous to the stratification of randomisation). In addition, the analysis is adjusted for age, type and number of chemotherapies, ECOG status at study inclusion, computer experience and, if necessary, for other important confounders. The heterogeneity between the study centres is taken into account by *random effects* (mixed linear regression model, *random intercept model*).

Analyses of the Secondary Hypotheses

All secondary hypotheses are analysed analogously to the primary hypothesis and according to the respective outcome using suitable statistical methods (e.g. time-event models, ANCOVA, logistic regression, Cox regression). As with the analysis of the primary outcome, the secondary hypotheses are also adjusted for relevant confounders and the heterogeneity between the study centres is statistically taken into account appropriately. Additional outcomes and in particular different subgroups can be included in the analysis during the course of the study. In this case, they are listed in the statistical analysis plan (SAP) and evaluated exploratively.

Dealing with missing values

Missing values are imputed using multiple imputation methods, assuming that they are *missing at random* (MAR). In all cases, the reasons for missing values are documented and reported as best as possible.

Statistical analyses of the subgroups

For the statistical analyses of the subgroups, an interaction term of the treatment variable with the subgroup is initially included in the ANCOVA model (outcome: primary or secondary outcome, covariates: respective baseline variable, treatment variable, stratification variables of randomization, random intercept for the centres) in order to analyse initial indications of differential treatment effects in the subgroups. In addition, marginal effect estimates for each subgroup and 95% confidence intervals are given from the models. All subgroup analyses are exploratory.

Definition of subgroups and justification of the importance of subgroup analyses

- **metastasis** (secondary/previous therapies, primary/therapy naivety)
- **tumour subtype** (HR+/HER2+, HR+/HER2–, HR–/HER2+, TNBC)
- **site of metastasis** (cerebral, visceral, osseous, multiple)
- **age at diagnosis of metastases** (<50, >50)
- **disease free interval (DFI)** (<5 years, >5 years)

- **therapy at study inclusion** (chemotherapy, endocrine therapy, antibody therapy/hybrid therapy)
- **relationship status** (partnership/marriage, single)
- **income** (median split)
- **depression** (yes/formerly, no)
- **BMI** (<30, ≥30 kg/m ^{2})

Patients with **primary metastatic breast cancer** have a better prognosis than patients with recurrent metastatic disease. This difference is attributed to the fact that any adjuvant systemic therapies for the latter may lead to resistances that are not present in treatment-naive patients, i.e. those with primary metastases (21, 22). Within the group of **secondary metastatic tumours,** a longer disease-free interval (DFI) is considered to be prognostically favourable (21). Another factor that determines the prognosis is the **age at the time of initial diagnosis** of metastases. For example, a younger age at the initial occurrence of distant metastases is associated with a better prognosis (21). In this context, the reason for the poorer survival rate of patients over 50 could be a lower response rate to chemotherapy, with possibly more side effects and more frequent dose reductions (22). Furthermore, **tumour biology** is considered a key prognostic factor, as the pattern of spread of neoplastic cells depends on it, among other things. The best prognosis is described for hormone receptor (HR) positive tumours (luminal A), followed by HER2neu (HER) expressing tumours, and the worst for luminal B and triple-negative tumours (TNBC) (23). These differences are attributed, among other things, to different metastasis tropisms. Luminal tumours (HR+/HER-) primarily have osseous metastases, whereas TNBCs have far more frequent visceral and cerebral metastases (24), with the latter being associated with the poorest overall survival (OS) (23). Regardless of hormone receptor expression, HER2neu positive tumours most frequently have visceral metastases, which are also associated with a shorter overall survival than bone metastases (23). HER2neu-expressing tumours also represent a risk factor for cerebral metastases, since the available HER2neu-targeted therapy (e.g., trastuzumab) is not permeable to the cerebrospinal fluid (25).

Another prognostic factor is fatigue, which was defined as the primary endpoint for the PRO B study. This is influenced by various parameters. For example, there are **therapy-dependent differences** in the occurrence and course of fatigue-specific symptoms between patients after chemotherapy and endocrine therapy (26). **Sociodemographic characteristics** such as income and relationship status of those affected are also associated with the occurrence of tumour-related fatigue (27). Several studies have also found that fatigue is associated with **depression** and lack **of physical activity/increased body mass index** (28, 29).

Health economic evaluation

Over the study period, the total treatment costs will be collected and evaluated using health insurance data for all participating women. The respective observation period will also be taken into account (average treatment costs per month per patient).

To account for the skewed distribution of the cost data, generalized linear models with gamma distribution and log-link function are used for the statistical analysis. Additional statistical methods of health economic analysis are carried out in the statistical analysis plan at the beginning of the project. Cost-effectiveness analyses are carried out if the intervention proves to be superior in terms of "quality adjusted life years (QALYs)". A QALY takes into account both the life span and quality of life of interventions. QALYs are calculated on the basis of quality of life questionnaires (EORTC QLQ-C30) that are completed at baseline and at various subsequent points in time during the project. A linear temporal change in quality of life is assumed between the measurement points. QALYs are calculated by calculating the area under the curve. If the intervention leads to better quality of life values at lower costs, it is considered cost-effective. If there are additional costs in the intervention group, the incremental cost-effectiveness ratio (ICER) is calculated by estimating the mean additional cost for an additional QALY. A threshold of €50,000 per extra QALY is often used in studies as a cut off point for the cost-effectiveness of an intervention. If the ICER is below this threshold, the intervention is considered cost-effective.

8 Data management

8.1 Data collection

The data reported by patients is collected using a web tool developed by NeuroSys, which participating patients can access either via an app or via an email link. When initially registering with the web tool, an identification number is generated (Patient Concept ID, 6 digits consisting of numbers and upper- and lower-case letters), which can be used to collect the survey data pseudonymously and link it to the other data collected. Both the study centre and the study centre also have access to the web tool: The web tool notifies the study centre and the study centre of any abnormal values (alarm), whereupon the study centre can acknowledge alarms and document any measures taken.

The study centre collects certification-relevant clinical and process-related patient information via the tumour documentation system. In addition, an eCRF (study documentation system TuDoc) is programmed by OnkoZert, in which further study-relevant information is documented by the study centre. The entries in the web tool (survey data, alarm acknowledgement, possible measures) as well as in the tumour documentation system and the eCRF are downloaded from the study centre at defined times (in any case after 12 and 24 months) as XML or JSON files.

The sum of all project data (clinical tumour data, study data and survey data) from each individual centre is then merged via the OncoBox, quality-checked and sent to OnkoZert as an XML or JSON file via a secure environment (e.g. Citrix environment). The XML or JSON data sets from all centres are then merged by OnkoZert using the OncoBox Research. OnkoZert carries out further quality assurance of the data (completeness, etc.) and forwards it to the PRO B headquarters for evaluation via a secure environment (e.g. Citrix environment).

In addition, routine data is provided by the patients' respective health insurance companies and linked to the other data in the central study database via a data integration point (see chapter 8.3). Figures 3 and 4 summarise the data flow in PRO B.

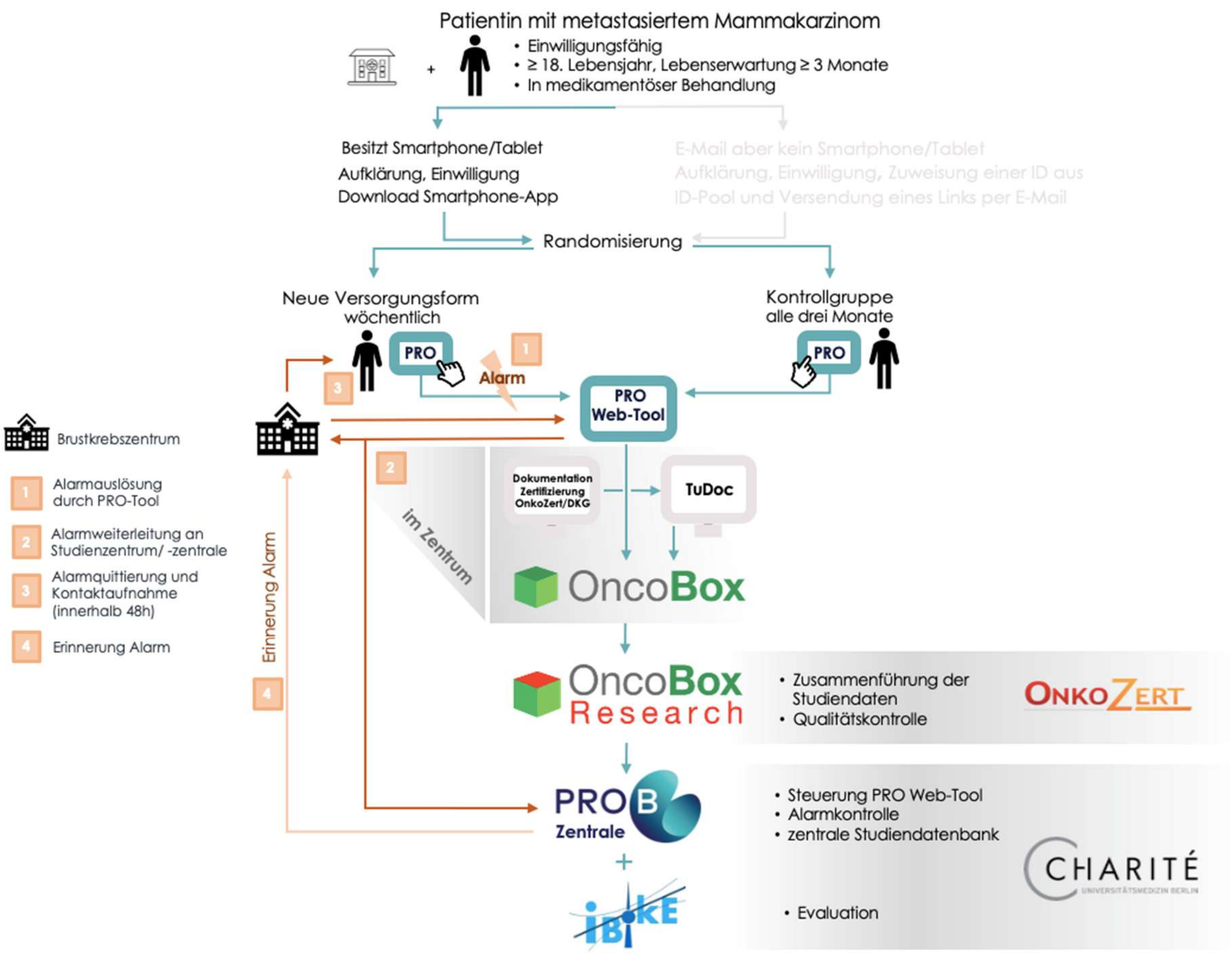

Figure 3 Data flow. *PRO* Patient-Reported Outcome, *TuDoc* study documentation system, *iBiKE* Institute for Biometry and Clinical Epidemiology

8.2 Data processing, quality control, monitoring

If data are not available or not applicable during the survey, this will be noted accordingly. With regard to medications and side effects, the terminology of the international reference lists (WHO dictionary, MeDRA) will be observed.

The study documentation is monitored by a commissioned Clinical Research Organization (CRO). To this end, the CRO carries out a one-time in-person monitoring for each study centre, in which a random sample of source data is compared and checked for completeness, consistency and plausibility. If deficiencies are identified in the study documentation, the study centre is asked to correct them. In a second in-person monitoring, the correction of the documentation deficiencies is then checked.

Before the data sets are sent by the study centres, OncoBox checks the data for plausibility and completeness (quality assurance). In addition, OnkoZert carries out a quality control upon receipt of the data sets in accordance with the OncoBox criteria.

The data is evaluated by the Institute of Biometry and Clinical Epidemiology (iBiKE) at the Charité Universitätsmedizin Berlin.

8.3 Data storage and data protection

A data protection concept will be drawn up and reviewed externally by the law firm Beiten Burkhardt (Appendix 2). The report will be available before the start of the study.

As part of the PRO B study, survey data reported by patients and clinical data documented by the study centres are collected and then linked to routine data from health insurance companies.

The survey data is collected in pseudonymized form using a web tool (PatientConcep-ID) programmed by NeuroSys. The identifying data associated with a patient ID is only available to the treating study centre and is not transmitted to the central study database. Data processing and storage within the framework of the web tool is carried out in accordance with the data protection concept. The patient-related data (tumour documentation) collected in the centres is stored locally for at least ten years after completion. The centres involved ensure that only authorized persons can access personal data, e.g. through access control through technical measures in secure rooms, user control through password regulations for legitimation and through automatic screen locking, and through access control by assigning different authorizations and differentiated access options to individual fields (confidentiality). They also guarantee that personal data remains intact, complete and up-to-date during processing, e.g. by preventing unauthorized or accidental data processing by blocking access to operating systems and/or encrypting the data and by regularly checking that the data is up-to-date (integrity); also that personal data is available in a timely manner and can be processed properly for pseudonymization and subsequent transmission, e.g. by clearly and transparently organizing the data set and granting access authorizations to the extent necessary (while balancing the requirement of confidentiality) (availability). Furthermore, they ensure that personal data can be assigned to its origin at any time, e.g. by documenting the original data

and its origin and traceability of the processing steps (authenticity). They also ensure that it can be determined who processed which personal data and in what way, when, e.g. by defining clear authorities and responsibilities and logging the input and further processing of the data (auditability). The procedures for processing personal data are documented completely, up-to-date and in such a way that they can be traced within a reasonable time.

At the end of the project, the routine data from the health insurance companies are transmitted in pseudonymized form to the central study database (see Figure 4). The data is transmitted via a trust office and a data integration centre: (1) The trust office (CHA-THS) provides the participating study centres with study IDs that are assigned to each patient. (2) The study centres transmit the study ID together with the PatientConcept ID from the app or the PatientConcep ID pool and the insurance number back to the CHA-THS. (3) The CHA-THS assigns a new health insurance pseudonym (KK pseudonym) to each study ID and transmits this together with the insurance number to the health insurance companies. (4) In conjunction with the KK pseudonym, the health insurance companies transmit the secondary data to the data integration centre. (5) The data integration centre receives the PatientConcept ID including the study ID, the KK pseudonym and a new ID for the evaluation (evaluation ID) from the CHA-THS. (6) The health insurance data is then linked to the other study data from the PRO B headquarters. (7) Finally, the complete data set with the evaluation ID is forwarded to the evaluating institute (iBiKE).

The various data are transferred to the central study database (located at the Charité) via secure environments. The data is processed and stored in accordance with the Charité's applicable data protection regulations and data protection concepts.

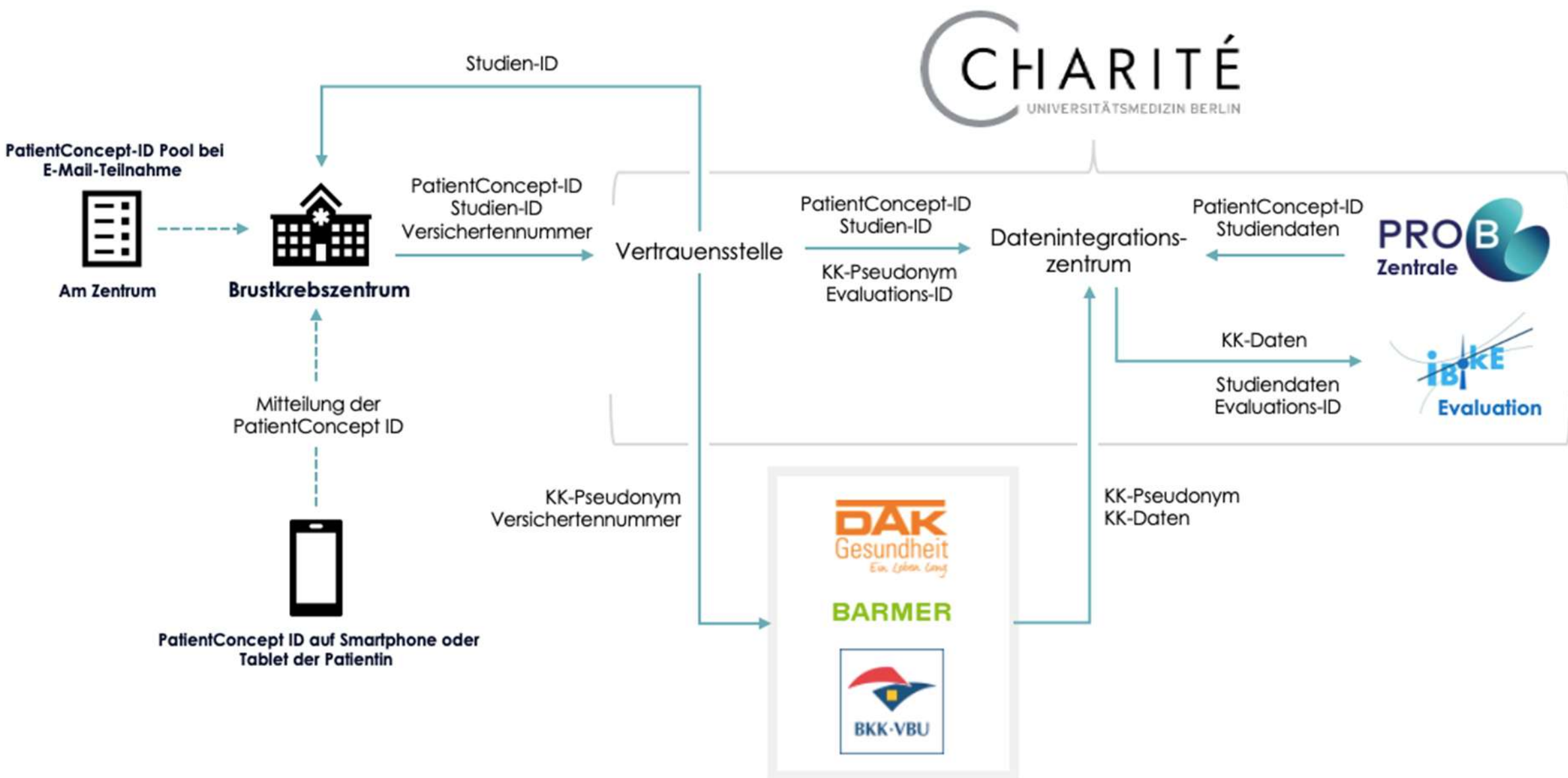

Figure 4 Transmission of health insurance data. *KK* health insurance companies

9 Ethical and legal aspects

9.1 Legal basis

Inclusion in the study is based on informed consent from the patient in accordance with the attached consent form (see Appendix 1). The intervention is carried out on the legal basis of a contract for special care in accordance with Section 140a SGBV.

9.2 Ethics committee

Before the study begins, the study protocol, patient information and consent form, and other relevant documents are submitted to an independent ethics committee that votes first. After the study centres have been determined, the documents are submitted to the relevant local ethics committees for consultation (organizational support provided by CRO). A positive written vote from the ethics committees is a prerequisite for the start of this study.

9.3 Patient information and consent form

The patient information, data protection information and consent form can be viewed in Appendix 1 and were prepared in accordance with the Ethics Committee of the Charité and the Medical Informatics Initiative (MII)[2].

9.4 Expense allowance

The participating patients will not receive any compensation for expenses.

9.5 Insurance

Study-specific insurance coverage is not provided.

10 Administrative aspects

10.1 Study financing

The study is funded by the Innovation Committee of the Federal Joint Committee (grant number 01NVF19013).

[2] https://www.medizininformatik-initiative.de/de/medizininformatik-initiative-erhaelt-gruenes-licht-fuer-bundesweit-patienteneinwilligung

10.2 Study reports

The responsible evaluating institute (iBiKE) will, together with the study management, submit an evaluation report within 6 months after the end of the study.

10.3 Study registration

Before the start of the recruitment phase, the study will be registered by the study management in the German Clinical Trials Register (DRKS) and in the study register of the US National Library of Medicine (Clinicaltrials.gov).

10.4 Publications

All information relating to the study must be treated confidentially before publication. The dissemination of the primary outcome of the study in the form of presentation(s) and/or publication(s) is reserved for the study director (Dr. med. Maria Margarete Karsten) or a representative chosen by her. Collaborators who have made a significant contribution to the conception and implementation of the study and/or to patient recruitment can contribute to the publication as co-authors. The selection of co-authors and the order of authorship are determined by the study director in accordance with scientific standards and in consultation with the consortium, if the selected journal is restricted. Further publications relating to the study are only permitted with the written consent and after review by the study director, whereby prevention or delay is only possible for good reasons. A draft of the abstract or manuscript must therefore be submitted to the study director at least one month before the planned submission or presentation.

At least two publications in peer-reviewed journals are planned. In addition, the study will be presented in the member magazines of the cooperating health insurance companies and the cooperating professional society (German Cancer Society). Publications in patient magazines such as Mamma Mia and mamaZone are also planned.

11 Signature of the Principal Investigator

Berlin, September 30, 2020

____

Dr. med. Maria Margarete Karsten

Bibliography

1. Mariotto AB, Etzioni R, Hurlbert M, Penberthy L, Mayer M. Estimation of the Number of Women Living with Metastatic Breast Cancer in the United States. Cancer Epidemiol Biomarkers Prev. 2017;26(6):809-15.

2. Robert Koch Institute. Cancer in Germany for 2015/16. Berlin2019.

3. Caswell-Jin JL, Plevritis SK, Tian L, Cadham CJ, Xu C, Stout NK, Sledge GW, Mandelblatt JS, Kurian AW. Change in Survival in Metastatic Breast Cancer with Treatment Advances: Meta-Analysis and Systematic Review. JNCI cancer spectrum. 2018;2(4):pky062.

4. Guidance for industry: patient-reported outcome measures: use in medical product development to support labeling claims: draft guidance. Health Qual Life Outcomes. 2006;4:79.

5. Basch E, Iasonos A, McDonough T, Barz A, Culkin A, Kris MG, Scher HI, Schrag D. Patient versus clinician symptom reporting using the National Cancer Institute Common Terminology Criteria for Adverse Events: results of a questionnaire-based study. The Lancet Oncology. 2006;7(11):903-9.

6. Di Maio M, Gallo C, Leighl NB, Piccirillo MC, Daniele G, Nuzzo F, Gridelli C, Gebbia V, Ciardiello F, De Placido S, Ceribelli A, Favaretto AG, de Matteis A, Feld R, Butts C, Bryce J, Signoriello S, Morabito A, Rocco G, Perrone F. Symptomatic toxicities experienced during anticancer treatment: agreement between patient and physician reporting in three randomized trials. J Clin Oncol. 2015;33(8):910-5.

7. Di Maio M, Basch E, Bryce J, Perrone F. Patient-reported outcomes in the evaluation of toxicity of anticancer treatments. Nature reviews Clinical oncology. 2016;13(5):319-25.

8. Groenvold M, Petersen MA, Idler E, Bjorner JB, Fayers PM, Mouridsen HT. Psychological distress and fatigue predicted recurrence and survival in primary breast cancer patients. Breast cancer research and treatment. 2007;105(2):209-19.

9. Müller V, Nabieva N, Häberle L, Taran FA, Hartkopf AD, Volz B, Overkamp F, Brandl AL, Kolberg HC, Hadji P, Tesch H, Ettl J, Lux MP, Lüftner D, Belleville E, Fasching PA, Janni W, Beckmann MW, Wimberger P, Hielscher C, Fehm TN, Brucker SY, Wallwiener D, Schneeweiss A, Wallwiener M. Impact of disease progression on health-related quality of life in patients with metastatic breast cancer in the PRAEGNANT breast cancer registry. The Breasts. 2018;37:154-60.

10. Park IH, Lee KS, Ro J. Effects of second and subsequent lines of chemotherapy for metastatic breast cancer. Clin Breast Cancer. 2015;15(1):e55-62.

11. Raskin W, Harle I, Hopman WM, Booth CM. Prognosis, Treatment Benefit and Goals of Care: What do Oncologists Discuss with Patients who have Incurable Cancer? Clin Oncol (R Coll Radiol). 2016;28(3):209-14.

12. Schwartz KL, Simon MS, Bylsma LC, Ruterbusch JJ, Beebe-Dimmer JL, Schultz NM, Flanders SC, Barlev A, Fryzek JP, Quek RGW. Clinical and economic burden associated with stage III to IV triple-negative breast cancer: A SEER-Medicare historical cohort study in elderly women in the United States. Cancer. 2018;124(10):2104-14.

13. Sorensen SV, Goh JW, Pan F, Chen C, Yardley D, Martín M, Knopf K, Benedict A, Giorgetti C, Iyer S. Incidence-based cost-of-illness model for metastatic breast cancer in the United States. Int J Technol Assess Health Care. 2012;28(1):12-21.

14. Basch E, Deal AM, Kris MG, Scher HI, Hudis CA, Sabbatini P, Rogak L, Bennett AV, Dueck AC, Atkinson TM, Chou JF, Dulko D, Sit L, Barz A, Novotny P, Fruscione M, Sloan JA, Schrag D. Symptom Monitoring With Patient-Reported Outcomes During Routine Cancer Treatment: A Randomized Controlled Trial. J Clin Oncol. 2016;34(6):557-65.

15. Basch E, Deal AM, Dueck AC, Scher HI, Kris MG, Hudis C, Schrag D. Overall Survival Results of a Trial Assessing Patient-Reported Outcomes for Symptom Monitoring During Routine Cancer Treatment. JAMA. 2017;318(2):197-8.

16. Denis F, Basch E, Septans AL, Bennouna J, Urban T, Dueck AC, Letellier C. Two-Year Survival Comparing Web-Based Symptom Monitoring vs Routine Surveillance Following Treatment for Lung Cancer. JAMA. 2019;321(3):306-7.

17. Pocock SJ, Simon R. Sequential treatment assignment with balancing for prognostic factors in the controlled clinical trial. Biometrics. 1975;31(1):103-15.

18. Cocks K, King MT, Velikova G, de Castro G, Jr., Martyn St-James M, Fayers PM, Brown JM. Evidence-based guidelines for interpreting change scores for the European Organization for the Research and Treatment of Cancer Quality of Life Questionnaire Core 30. Eur J Cancer. 2012;48(11):1713-21.

19. Cortes J, Hudgens S, Twelves C, Perez EA, Awada A, Yelle L, McCutcheon S, Kaufman PA, Forsythe A, Velikova G. Health-related quality of life in patients with locally advanced or metastatic breast cancer treated with eribulin mesylate or capecitabine in an open-label randomized phase 3 trial. Breast cancer research and treatment. 2015;154(3):509-20.

20. Borm GF, Fransen J, Lemmens WA. A simple sample size formula for analysis of covariance in randomized clinical trials. J Clin Epidemiol. 2007;60(12):1234-8.

21. Dawood S, Broglio K, Ensor J, Hortobagyi GN, Giordano SH. Survival differences among women with de novo stage IV and relapsed breast cancer. Annals of oncology: official journal of the European Society for Medical Oncology. 2010;21(11):2169-74.

22. Largillier R, Ferrero JM, Doyen J, Barriere J, Namer M, Mari V, Courdi A, Hannoun-Levi JM, Ettore F, Birtwisle-Peyrottes I, Balu-Maestro C, Marcy PY, Raoust I, Lallement M, Chamorey E. Prognostic factors in 1,038 women with metastatic breast cancer. Annals of oncology: official journal of the European Society for Medical Oncology. 2008;19(12):2012-9.

23. Kast K, Link T, Friedrich K, Petzold A, Niedostatek A, Schoffer O, Werner C, Klug SJ, Werner A, Gatzweiler A, Richter B, Baretton G, Wimberger P. Impact of breast cancer subtypes and patterns of metastasis on outcome. Breast cancer research and treatment. 2015;150(3):621-9.

24. Soni A, Ren Z, Hameed O, Chanda D, Morgan CJ, Siegal GP, Wei S. Breast cancer subtypes predispose the site of distant metastases. American Journal of Clinical Pathology. 2015;143(4):471-8.

25. Lin NU, Bellon JR, Winer EP. CNS Metastases in Breast Cancer. Journal of Clinical Oncology. 2004;22(17):3608-17.

26. Bower JE, Wiley J, Petersen L, Irwin MR, Cole SW, Ganz PA. Fatigue after breast cancer treatment: Biobehavioral predictors of fatigue trajectories. Health psychology : official journal of the Division of Health Psychology, American Psychological Association. 2018;37(11):1025-34.

27. Bower JE, Ganz PA, Desmond KA, Rowland JH, Meyerowitz BE, Belin TR. Fatigue in breast cancer survivors: occurrence, correlates, and impact on quality of life. J Clin Oncol. 2000;18(4):743-53.

28. Bower JE. Cancer-related fatigue--mechanisms, risk factors, and treatments. Nature reviews Clinical oncology. 2014;11(10):597-609.

29. Andrykowski MA, Donovan KA, Laronga C, Jacobsen PB. Prevalence, predictors, and characteristics of off-treatment fatigue in breast cancer survivors. Cancer. 2010;116(24):5740-8.

Abbreviations

CRO: Clinical Research Organisation;

CSC: Clinical Study Centre;

DKG: German Cancer Society (Deutsche Krebsgesellschaft);

ECOG: Eastern Cooperative Oncology Group;

EORTC: European Organisation for Research and Treatment of Cancer;

GCP: Good Clinical Practice;

HER: Human epidermal growth factor receptor 2;

HR: Hormone receptor;

HRQoL: Health-related quality of life;

iBiKE: Institute of Biometry and Clinical Epidemiology (Institut für Biometrie und Klinische Epidemiologie);

ICER: Incremental cost-effectiveness ratio;

ICMJE: International Committee of Medical Journal Editors;

MAR: Missing at random;

MBC: Metastatic breast cancer;

PRO: Patient-reported outcome;

QALY: Quality-adjusted life year;

QoL: Quality of life;

SAP: Statistical analysis plan

Appendices

1. Patient information, data protection information and consent form
2. Data protection concept

STATISTICAL ANALYSIS PLAN

PRO B – Patient Reported Outcomes in Breast Diseases

New Approaches to the Care of Metastatic Breast Cancer

Short title: PRO B

Intervention: Weekly PRO survey via a smartphone app, contact with a doctor in case of deterioration, possible individual treatment measures (intervention group)

Control: Quarterly PRO survey via a smartphone app

Study population: Women with metastatic breast cancer

Funding: Innovation Committee at the G-BA (funding code: 01NVF19013)

Registration of the study: DRKS (German Clinical Trials Register) DRKS00024015. Registration on February 15, 2021

Analysis plan version: Version 1.0 (Final)

Date of the analysis plan: January 18, 2024

Principal Investigator: PD Dr. med. Maria Margarete Karsten

Biostatisticians:

PD Dr.phil. Ulrike Grittner **Signature**

Dr.rer.medic. Pimrapat Gebert **Signature**

Table of contents

1. Introduction 3
  1.1 Aim of the study 3
  1.2 Study design 3
  1.3 Inclusion and exclusion criteria 4
  1.4 Randomization 4
  1.5 Study group 5
    1.5.1 Intervention group 5
    1.5.2 Control group 5
  1.6 Sample size calculation 5
2. Analysis sets 6
  2.1 Definitions 6
  2.2 Application 6
3. Variables for analysis 7
  3.1 Primary and secondary outcomes 7
  3.2 Definition for time-to-event analysis 7
    3.2.1 Last contact date 7
    3.2.2 Time to first systemic therapy changes 7
  3.3 Baseline characteristics and possible confounders 8
4. Dealing with missing data 10
5. Statistical evaluations 10
  5.1 Analysis of the primary endpoint 10
  5.2 Analyses of secondary endpoints 13
  5.3 Sensitivity analyses 14
  5.4 Statistical analyses of the subgroups 14
6. Health economic evaluation 15
  6.1 Objectives 15
  6.2 Sample size estimation 15
  6.3 Health economic outcomes 16
  6.4 Use of health resources and costs 16
  6.5 Statistical evaluation 17
    6.5.1 Analyses of health resource use and costs 17
    6.5.2 Quality-adjusted life years 18
    6.5.3 Cost-effectiveness analysis 18
    6.5.4 Software 18
7. Examples of the presentation of the tables 19
8. References 23
9. Abbreviations 25

1. Introduction

1.1 Aim of the study

Breast cancer is the most common malignancy in women. The development of metastases in breast cancer patients compromises the overall survival and quality of life of patients. Nowadays, electronic patient-reported outcomes (PROs) are used to collect real-time data from patients, such as medication use, symptoms or health status. Intensified PRO monitoring has been shown to improve quality of life and reduce unplanned hospitalizations and emergency department visits in patients with metastatic breast cancer [1]. In addition, a positive impact of intensified PRO monitoring on survival has been demonstrated [1,2]. Therefore, early detection of deterioration in quality of life could spare patients treatment cycles with therapy that is no longer effective.

The PRO B study aims to investigate whether the benefits of PRO monitoring from a monocentric study setting can be transferred to routine German care and whether the positive effects on overall survival and quality of life can also be demonstrated in breast cancer patients. The study objectives according to the protocol are presented in Table 1.

Table 1 Objectives of the study

**Primary objective**
Patients who receive weekly PRO surveys in combination with a physician contact in case of deterioration have lower fatigue scores after 6 months than patients who receive quarterly PRO surveys
**Secondary objectives**
Patients in the intervention group compared to the control group
… after 6 and 12 months a higher total score of health-related quality of life (HRQoL)
… after 6 and 12 months a higher score in physical function
… after 12 months a lower fatigue score
… after 6 and 12 months, a lower number of hospitalizations and emergency room visits
… have a shorter time to first systemic therapy changes due to the early detection of therapy intolerance or disease progression
… after 12 months an increased survival rate in visceral metastasis
… after 12 months an increased survival rate in triple negative breast cancer

1.2 Study design

PRO B is a prospective, multicenter, randomized, controlled, non-blinded two-arm study with 1:1 randomization. In the intervention group, patients receive weekly PRO surveys and an alert

is generated if the PRO values deteriorate. This alert is acknowledged and documented by the cooperating study centres within 48 hours. The doctor decides together with the patient about possible further interventions. The patients in the control group receive PRO surveys every 3 months and are treated as standard and without an alert being generated. The entire study design is shown graphically in Figure 1.

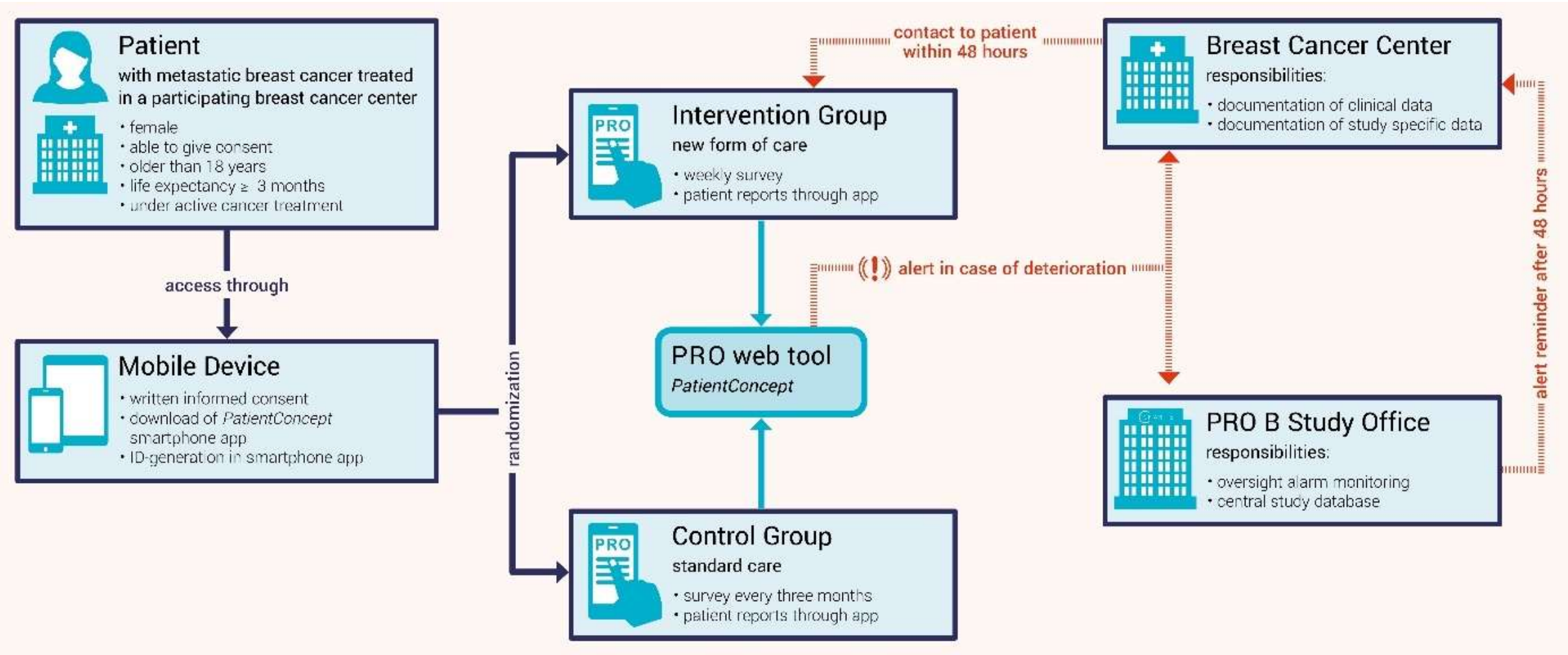

Figure 1 Study design

1.3 Inclusion and exclusion criteria

Eligible patients are female, older than 18 years, able to read and understand German, receiving drug treatment for MBC with a life expectancy of more than 3 months at the time of participation, insured with one of the three participating health insurance companies and being treated in a participating breast cancer centre. In addition, Internet access via a smartphone or tablet, an ECOG performance status (Eastern Cooperative Oncology Group) score of 0 to 2 and willingness to participate in a weekly, online-based PRO survey were required.

Patients who do not receive active tumor therapy (comfort care) or who do not meet all of the above inclusion criteria will be excluded.

1.4 Randomization

A stratified 1:1 randomization will be performed according to the following strata:

- Centers
- Types of distant metastasis (3 groups)
  - Bone metastases or skin metastases or lymph node metastases
  - Visceral metastases (one organ)
  - Brain metastases or multiple metastases

- Histological findings (2 groups)
  - HR+/HER− or HR+/HER+
  - HR−/HER+ or HR−/HER−

Adaptive randomization is performed using electronic randomization software secuTrial®, which is managed by the Clinical Study Centre (CSC) of the Charité - Universitätsmedizin Berlin. Randomisation takes place after written consent, downloading the application and before the app-based baseline survey (including socio-demographic information and medical history). Group allocation by secuTrial® is carried out automatically for individual patients without a predefined order after the study team has checked the inclusion criteria, given the consent and entered the patient characteristics of the strata variables.

1.5 Study group

1.5.1 Intervention group

Patients in the intervention group receive a weekly PRO survey via the app (always on the same day of the week). The PRO survey is based on the HRQoL measurement providing by the European Organization of Research and Treatment of Cancer (EORTC) Quality of Life Group. Its core instrument, the EORTC QLQ-C30, includes five functional dimensions (physical function, role function, emotional function, cognitive function, social function) and nine symptom scales (fatigue, nausea, pain, dyspnea, insomnia, loss of appetite, constipation, diarrhea, financial difficulties)[3]. These domains are assessed using item response theory, short forms of different length and item content were constructed for each domain, tailored to achieve maximum accuracy in the expected range of scores[4]. PRO scores are automatically compared to the previous week's scores to identify short-term changes and to the average of scores 3, 4, and 5 weeks earlier to identify long-term changes in HRQoL. An alert is triggered in the event of a clinically relevant deterioration in PRO scores.

1.5.2 Control group

Patients in the control arm receive a PRO survey via the app every 3 months, but no alert is triggered if PRO values worsen and no contact.

1.6 Sample size calculation

In a meta-analysis of studies on changes in the EORTC QLQ-C30, Cocks et al. analyzed 35 studies that reported an improvement in the fatigue scores[5]. Cocks et al. created guidelines for the interpretation of longitudinal changes on the fatigue score, with scores of an average improvement of 4 points or more being considered a small improvement and scores of 9 points or more being considered a medium improvement. However, our study is about a group comparison between treatment and control group. We have based our effect estimates on the information provided by Cocks et al. However, due to the two-group comparison, we expect

much larger measures of dispersion and thus conservatively estimate a standardized effect size of around 0.2 (mean difference, for example, 5 points. Common standard deviation 25.2 points). Such a dispersion is comparable to the information provided by Cortes et al. 2015 [6].

Since the analysis of certain subgroups, for example patients with visceral metastases, is particularly relevant, a sample size that is sufficiently large for subgroup analyses is aimed for. The recruitment of approximately 500 patients per group is considered realistic. With an expected drop-out rate of 20%, this results in approximately 400 completed data sets per study group. If there is a minimal clinically relevant effect of 5 points mean difference on the fatigue scale of the EORTC QLQ-C30, as reported by Cocks et al. (with a common standard deviation of 25), a two-sided t-test with a significance level of 5% has a power of 80.65%. This calculation was performed with nQuery (version 8.3.1.0)[7]. The number of cases is therefore sufficient for the detection of small effect sizes (0.2 or larger) and considers the number of relevant subgroups in the patient population.

Since the planned sample size cannot be achieved in the originally intended recruitment period, the recruitment period will be extended by four months. The total expected sample size is 400 patients per group. With an expected drop-out rate of 20%, this results in approximately 320 completed cases per study group. This sample size is therefore sufficient to detect effect sizes of 0.2 using a two-sided t-test for independent samples with a significance level of 5% and a power of 71.4%. If the effect size is 0.225 or larger, a power of at least 81.1% can be achieved.

2. Analysis sets

2.1 Definitions

The full analysis set includes all subjects who were randomized and who answered at least the PRO survey. Patients who never respond to the PRO survey are not considered to a full analysis set (modified ITT population). In case of missing values, multiple imputation methods are used to estimate the missing values. The analysis set per protocol (per protocol analysis set) includes all subjects who received an intervention or control treatment for at least 6 months as planned.

2.2 Application

The primary analysis is performed using the full analysis set, including the estimated values from multiple imputations for missing values (ITT). An analysis of the primary outcome from the analysis set according to the protocol is used as a sensitivity analysis.

3. Variables for analysis

3.1 Primary and secondary outcomes

The primary endpoint and secondary endpoints of the study are listed in Table 2 below. Additional variables that are relevant in connection with randomization or as possible confounders are defined for the baseline survey and the study documentation system (eCRF in PRO B Doc). Data collection in PRO B Doc takes place every three months as part of the regular clinical presentation at the treating breast cancer centre.

The outcomes (after 6 and 12 months, mentioned above) are available at the end of the recruitment and intervention phase for all included patients (excluding dropouts). The primary and secondary outcomes are also analyzed for later measurement points (up to a maximum of 24 months after inclusion), each with a smaller *n* in order to be able to depict the PRO course for metastatic breast cancer patients over the longest possible period.

<u>Note on the time of measurement of the PROs</u>

If the patient missed the weekly PRO assessment, PRO scores will be replaced by the PRO scores observed before or after (+/- 1 week) the time points analyzed at baseline (0 week), 3 months (12 weeks), 6 months (24 weeks), 9 months (36 weeks), and 12 months (48 weeks). For example, if the patient did not respond to the PRO-B survey at week 12 but PRO data were observed at week 11, PRO data for week 12 will be used. If both assessments are observed (before and after the missing time point), the score obtained before the missing time point will be used.

3.2 Definition for time-to-event analysis

3.2.1 Last contact date

The date of last contact for living patients is determined based on the last of the following dates:

- Examination data (e.g. laboratory, discharge date, etc.)
- Last date of response to the PRO survey
- Date of the study end

The date of last contact is applied to censored patient in the time-to-event analysis.

3.2.2 Time to first systemic therapy changes

Time to first systemic therapy change will be defined as the time lag between the date of randomization and the date of the first systemic therapy change, and censored will be considered at the last follow-up date or the date of the study end.

Table 2 Overview of primary and secondary outcomes

Variable	Data source	Time of collection				
		**Baseline**	**Weekly in intervention group; every 3 months in control group**	**6 months**	**12 months**	**24 months**
**Primary outcome**						
Fatigue Score (EORTC)	Study-specific questionnaire from EORTC Itembank	x	x	x	x	x
**Secondary Outcomes**						
Physical functioning score (EORTC)	Study-specific questionnaire from EORTC Itembank	x	x	x	x	x
Health-related Quality of Life sum score (EORTC)	Study-specific questionnaire from EORTC Itembank	x	x	x	x	x
Number of hospital and emergency room stays	health insurance data				x	
Overall survival in visceral metastasis	PRO B Doc, health insurance data				x	x*
Overall survival in triple negative breast cancer	PRO B Doc, health insurance data				x	x*
Time until the first change of the systemic therapy	PRO B Doc				x	x*
EQ-5D		x		x	x	x

*from PRO B Doc

3.3 Baseline characteristics and possible confounders

Potential confounding factors that are considered to adjust for all the analyses are listed below:

- Age at randomization (years)
- Type of metastases (bone, lymph node, visceral, brain, or multiple sites)
- Hormone-receptor status (HR+ or HR-)
- Types of systemic therapy at randomization (endocrine, chemotherapy, anti-Her2 therapy, immunotherapy, targeted therapy, and others)
- ECOG status at enrolment (ECOG 0, 1, 2)
- Changing alarm criteria (before and after 01.09.2022)
- Socio-economic and anamnesis parametersas possible confounders once during the initial survey:
    - Marital status (multiple categories)
    - Having dependents in need of care or underage children (≤14 years) in the household (yes or no)

- Educational status (according to ISCED [8])
- Employment status (yes or no)
- Income (multiple categories)
- Migration background (yes or no)
- National Cancer Institute (NCI) comorbidity index (numeric)

In addition to the breast cancer-specific medical history (e.g. family history of cancer), the survey via the smartphone application also enables the collection of any previous illnesses (preliminary questionnaire below):

- Cardiac and cardiovascular pre-existing conditions (arterial hypertension, coronary heart disease, angina pectoris, myocardial infarction, heart failure, atrial fibrillation, valvular disease)
- Previous vascular diseases (deep vein thrombosis, peripheral arterial occlusive disease, pulmonary embolism)
- Cerebrovascular diseases (TIA, stroke)
- Previous hematological diseases (anemia)
- Pulmonary diseases (bronchial asthma, COPD, sleep apnea)
- Endocrine/metabolic pre-existing diseases (hyperlipidemia, diabetes mellitus (requiring treatment with and without end-organ damage), hypothyroidism, obesity)
- Rheumatological pre-existing conditions (polymyalgia rheumatica, lupus erythematosus, rheumatoid arthritis, polymyositis)
- Previous mental illnesses (depression, anxiety disorder)
- Neurological pre-existing conditions (migraines, seizures, balance disorders, neuropathy, hemiplegia, dementia)
- Previous gastrointestinal diseases (gastrointestinal ulcers, chronic inflammatory bowel disease, diverticulitis, reflux, irritable bowel syndrome, mild liver disease (liver cirrhosis without portal hypertension), severe liver disease (liver cirrhosis with portal hypertension, esophageal variceal bleeding))
- Previous gynecological diseases (infertility, abortion, dysmenorrhea or hypermenorrhea, endometriosis, ovarian cysts)
- Previous urogenital diseases (nephro- or urolithiasis, renal insufficiency, dialysis requirement, micturition problems, urinary tract infections, incontinence)
- Infectious diseases (HIV, AIDS)
- Previous oncological diseases (other tumor diseases (with and without metastases), leukemia, lymphoma)
- Previous operations

- Drug history (alcohol, nicotine)

We suspect that the comorbidities described above have a significant influence on the primary endpoint of the study (fatigue). They must therefore be considered both in the interpretation of the measured values in the current project and in the final evaluation (e.g. as the National Cancer Institute (NCI) comorbidity index (underlined diseases)) should be considered.

4. Dealing with missing data

The handling of missing data is based on an estimand framework developed by the ICH guideline (International Council for Harmonization of Technical Requirements for Pharmaceuticals for Human Use) [9]. Table 3 shows the analysis plan of the PRO B data based on an estimation framework for our primary outcome. For patients who died or became too ill during the study period, the lowest possible values are used for measures such as quality of life or physical functioning (missing not at random, MNAR)[10]. Missing data are estimated using multiple imputation with chained equations (MICE) assuming missing data are missing at random (MAR). Thirty complete data sets are created. For patients who died during the study period, the lowest possible values are used for values such as quality of life or physical function. MICE is performed based on predictive mean matching for the continuous variables, ordinal logistic regression models for ordinal variables, and binary logistic regression models for binary variables. Due to the longer recruitment time of 4 months, the late recruited patients will only be fully followed for 6 months. No secondary endpoints at 12 months will be estimated for these patients using imputation methods.

5. Statistical evaluations

For each group, descriptive statistics and the number of participants included in the calculation (n) are given. For continuous variables, the mean with standard deviation (SD), median, interquartile range (IQR), minimum and maximum are given depending on the distribution. For categorical data, the absolute and relative frequencies for each category are shown. Graphical methods are used to visualize the data, e.g. box plots and line graphs.

All results will be analyzed using an intention-to-treat analysis as described in 2.2. This is the primary analysis set for the evaluation analyses.

5.1 Analysis of the primary endpoint

The primary endpoint is the EORTC T-score for fatigue at 6 months, which is based on a tailored short form from the EORTC item bank. The primary research question will be addressed in an intention-to-treat analysis. All randomization stratification variables will be considered as covariates in all statistical models.

A two-sided t-test was used to calculate the sample size, although an ANCOVA model is to be used in the evaluation. It has been shown that this is a conservative approach for estimating the sample size of ANCOVA models, since an ANCOVA model with $(1 - 2\rho)*n$ observations has at least the same power as a t-test with *n* observations [11], where *ρ* is the variance inflation factor (correlation between the *baseline* measurement and the *follow-up* measurement). In the worst case of $\rho = 0$, this leads to a required sample size equal to the t-test. The analysis is carried out in the ANCOVA model with baseline measurement as a covariate and adjustment for types of metastasis and histology (as in the stratification of randomization). In addition, the analysis is adjusted for age, type and number of chemotherapies, ECOG status at study inclusion, computer experience[1] and, if applicable, other important *confounders*. Heterogeneity between study centers is controlled for by considering as a *random effect* (mixed linear regression model, *random intercept model*). Effect estimates for the intervention effect and 95%CI will be reported.

Table 3 Analysis of PRO B data in an estimand framework

**Scientific research question**	**Is the mean fatigue score measured with the EORTC CAT Core lower (superior) in the intervention group than in the control group after six months?**
**Characteristics of the estimate**	
**Intervention**	Intervention: Patients receive weekly PRO surveys with an alert contact in case of deterioration of PRO values<br>Control: Patients receive quarterly PRO surveys without alert contact in case of deterioration of PRO values
**Target population**	Eligible patients are female, over 18 years of age, able to read and understand German, receiving medication for MBC and with a life expectancy of more than 3 months at the time of participation. In addition, Internet access via a smartphone, ECOG performance status score of 0 to 2, and willingness to participate in a weekly PRO survey were required.
**Endpoint of interest**	Fatigue T-scores after 6 months
**Treatment of intercurrent events**	
**Discontinuation of treatment**	
- Overload due to the PRO survey	Assumption of missing data according to MAR, implicit imputation of data according to MICE with *m* = 30 imputations
- No longer of interest or relevance	Assumption of missing data according to MAR, implicit imputation of data according to MICE with *m* = 30 imputations
- Patients feel too ill to continue participating in the study	Assuming that the absence is not random, the worst -case scenario is applied
**Loss to follow-up**	Assumption of missing data according to MAR, implicit imputation of the data according to the MI method with *m* = 30 imputations
**Death**	Assuming that the absence is not random, the worst-case scenario is applied
**Estimator**	Linear mixed model adjusted for baseline and stratification factors. The study center becomes as random intercept set
**Summary measure**	Mean difference in fatigue scores (IG - CG) and 95%CI (two-sided)
**Interpretation**	The mean difference in fatigue scores and the upper 95% CI limit are less than 0, meaning that the intervention group is superior to the control group

MAR= missing at random, MBC = metastatic breast cancer, ECOG = Eastern Cooperative Oncology Group, MICE = multiple imputation by chained equation, PRO = patient-reported outcome

5.2 Analyses of secondary endpoints

For the secondary endpoints (subscales of the EORTC T-score based on an adapted short form generated from the EORTC item bank, e.g. global health status/QoL, functioning, symptoms at different time points), mixed linear regression models similar to the model described above for the primary endpoint will be performed. Study centers and patients are treated as random effects (random intercept). An interaction term between study group and time points will be included in the models to estimate time-specific intervention effects. The shortening of follow-up for part of the sample with regard to secondary endpoints because patients are not fully followed for 12 months results in no PRO scores. For these secondary endpoints, we will not imputed data for those late-recruited patients because we did not collect any subsequent data from them at all. Therefore, the analyses of the secondary endpoints after 12 months will be based on a smaller sample size than the analyses for the primary endpoint.

A Cox proportional hazards model will be performed to compare overall survival and time-to-first systemic therapy change between study groups, adjusting for type of metastases, histology and other potentially confounding factors. Additionally, a random effect term (random intercept) for the center will be included to account for heterogeneity across centers. Moreover, the time-to-event analyses are considered so that the shorter observation times of the later recruited patients are adequately addressed. All available information is used and different observation periods do not pose a problem. Therefore, no imputation for missing data is made for those late-recruited patients. Hazards Ratios (HR) with two-sided 95% CI are presented. Kaplan-Meier curves and adjusted survival curves based on the Cox models are shown as descriptive diagrams.

We will estimate the subdistribution hazards ratio (SHR) of the time-to-first systemic therapy change and its 95%CI using the subdistribution proportional hazards models of Fine and Gray, assuming that death is a competing risk. The cumulative incidence of the first systemic therapy change is presented. We will apply the Cox proportional hazards model if the competing event is less than 10% or not more than the proportion of the first systemic therapy change, so the competing risk model is not required[12].

The primary hypothesis is tested with a two-sided significance level of $\alpha = 0.05$. All secondary hypotheses are analyzed as part of an exploratory study. No adjustment for multiple testing is made for the analyses of the secondary outcomes.

All statistical tests are performed using Stata MP18 (StataCorp, 2023, College Station, TX, USA).

5.3 Sensitivity analyses

If a significant result is found for the primary endpoint, a sensitivity analysis is performed to assess the robustness of the result of the primary analysis under the assumption of missing not at random (MNAR) by using a reference-based sensitivity analysis and a delta-adjusted sample mixture approach for tipping point analysis (TPA) [13,14]. The reference-based sensitivity analysis is an imputation approach for patients who discontinue the intervention, and the missing values are imputed as in the control group [14]. TPA assumes that there is a systematic difference between the conditional distributions of the missing and observed data. To reflect such a systematic difference, a shift parameter is applied δ to the imputation model when the missing data points are imputed. For each value of δ, multiply imputed data sets are generated, each data set is analyzed using the same method for the primary analysis, and the analysis results are then combined using Rubin's rule. By varying the values of δ, the effects of missing data on the analysis results are examined to identify tipping points (i.e., the values at which the conclusions change from significant to nonsignificant between study groups).

5.4 Statistical analyses of the subgroups

The subgroup analyses are conducted in an exploratory framework.

The following subgroups will be analyzed for possible differential intervention effects:

- Metastasis (secondary/therapy received, primary/therapy naïve)
- Tumor subtype (HR+/HER2+, HR+/HER2–, HR–/HER2+, TNBC)
- Site of metastasis (cerebral, visceral, bone, multiple)
- Age at randomization (<50, ≥50)
- Therapy at study inclusion (chemotherapy, endocrine therapy, anti-Her2 therapy, immunotherapy, targeted therapy and others)
- Relationship status (partnership/marriage, single)
- Income (median split)
- Depression (yes/formerly, no)
- BMI (<30, ≥30 kg/m^2)
- Change in alert criteria (before and after September 1st, 2022)

For the statistical analyses of the subgroups, the linear mixed model is first used (outcome primary or secondary outcomes, covariates: respective baseline variable, treatment variable, stratification variables of randomization, and intercept for the centers). An interaction term of the treatment variable with the subgroup is also included in order to obtain indications of differential treatment effects in the subgroups. In addition, marginal effect estimates for each subgroup and 95% confidence intervals are provided from the models.

Subgroup analyses of the overall survival between intervention and control groups will be performed separately for patients with visceral metastases and patients with triple-negative breast cancer.

6. Health economic evaluation

The health economic evaluation is only carried out on patients who are insured by the health insurance companies BARMER, DAK Gesundheit, and BKK·VBU.

6.1 Objectives

- Comparison of average costs between the intervention group and the control group
- Comparison of the frequency of hospital stays and emergency room visits between both groups
- Comparison of cost-effectiveness between the intervention group and the control group
- Comparison of the length of hospital stay between the study groups

6.2 Sample size estimation

The sample size estimate for the health economic analysis is based on the information from Basch et al. (2016)[1] on the intervention effect on quality of life measured with the EQ-5D in patients with cancer with PRO monitoring compared to standard care. Basch et al. (2016) report mean EQ-5D values of 84.8 (95% confidence interval: 83.2-86.4) in the intervention group and 79.5 (95% CI: 76.7-82.2) in the control group at 6 months after the start of the intervention (baseline values: intervention: 86.2, control 86.6). After scaling the values on a scale of 0-1 (instead of 0-100), the formula from Matthews et al. (1990) results in the calculation of the QALYs (quality adjusted life years) as area under the curve a value of 0.428 for the intervention group ((0.848+0.862)*0.5*0.5 years) and of 0.415 for the control group ((0.795+0.866)*0.5*0.5 years). This results in an intervention effect of 0.012 QALYs. Based on 12 months this would be an estimated effect of 0.0245 QALYs. We think that we can achieve a similar effect in our study.

We also assume that the new intervention will cost an additional €1000. To calculate the mean incremental cost-effectiveness ratio (ICER), the mean cost difference is divided by the estimated QALY difference: ICER=€1000/0.0245QALYs = €40.816. This is the mean estimated amount that must be raised for one additional QALY.

The upper limit for these expenses is often set at €50,000. The study initially only results in an estimate of the ICER, as it is based on mean values of the cost difference and the QALY difference between the groups. Therefore, no direct estimate of the number of cases is possible for the ICER. The 95% confidence interval (CI) for the ICER is determined using a bootstrap

procedure by bootstrapping the ICER separately for *n*=5000. Samples are calculated from the original data.

The sample size estimate for the health economic analysis is based on the EQ-5D values: Based on the values of Basch et al. (2016) regarding the EQ-5D values in both groups after 6 months, an effect size for the intervention effect of 0.331 (Cohen's d) can be calculated. A sample size of 200 people in each group has a power of more than 90% to detect a mean difference of 5.3 (the difference between the intervention group mean of 84.8 and the control group mean of 79.5), assuming that the common standard deviation is 16.01 (calculated based on the information on the 95% CI in Basch et al. 2016 and the sample sizes), using a t-test for two groups with a two-sided significance level of 5%.

Due to the expected drop-out rate of 20%, only 320 completed cases could be obtained from the 400 patients to be included from the participating health insurance companies. Incomplete cases will be excluded if there are missing values within the framework of the “completely missing at random (MCAR)” or “missing at random (MAR)” can be assumed using multiple imputation method[15] using the available data, so that 400 cases are available for the health economic question including estimated data. Even if only 320 complete data sets are available, the significance of the study is greater than 80% (significance = 83.9%). Consequently, it is sufficient to recruit 400 patients from the participating health insurance companies for the health economic question.

Since the originally planned sample size was not achieved, a sample size of around 150 patients per group is expected for the health economic analysis. With an expected drop-out rate of 20%, this results in around 120 completed cases per study group. The number of cases is therefore sufficient to demonstrate effect sizes of at least 0.331 with a power of 72.4%. If the actual effect size is 0.37 or greater, the power of the study is at least 81.4%.

We assume that this power consideration for the EQ-5D, which is used as the basis for calculating QALYs, forms the basis for a robust estimate of the ICER, for which no direct sample size estimate is possible.

6.3 Health economic outcomes

The following aspects are of interest: total treatment costs and the frequency of hospitalizations and emergency room visits, the length of hospital stay and the quality of life (EQ-5D).

6.4 Use of health resources and costs

Health resource utilization (HRU) and costs are calculated from claim data providing by the health insurance companies. HRU will be assessed by the number of outpatient and inpatient visits and the length of stay (days) in the hospital.

Patient co-payments are not considered. To determine direct costs, these are calculated separately as follows: outpatient care, inpatient care (outpatient, day-care and inpatient), medication prescriptions, therapeutic aids and remedies. Outpatient care includes all costs for services. Inpatient care summarizes all costs for services and administered medication during inpatient stays. Medication prescriptions include the costs for medication in the outpatient sector. Aids such as walking aids or wheelchairs are devices to support the patient in recovery and daily care. Remedies are services such as massages or physiotherapy that are provided by medical staff. Overall, health costs are calculated as the sum of the costs of all areas. Due to unequal observation times, all costs are averaged over the observation period so that the costs are presented as yearly costs.

6.5 Statistical evaluation

Baseline characteristics were presented using absolute frequencies and percentages for categorical variables and mean, standard deviation (SD), median, and interquartile range (IQR) for continuous variables.

6.5.1 Analyses of health resource use and costs

Due to the typical right-skewed distribution of LOS and cost data, we used log-transformed LOS (ln -LOS) and costs (ln-Cost) for the analysis. The exponent of the mean observed ln-LOS and ln-Cost corresponds to the geometric mean (GM) of the LOS and costs. A linear mixed model (LMM) is performed to compare the difference in ln-LOS and ln-Cost between the groups with patients and centers as random intercepts. The cost data are usually characterized by a significant proportion of zero-cost observations for people who did not use any health resources in a given period. If the proportion of zero costs in the study is high, a two-part model will be applied[16,17]. In the first part of the model, the probability of using a health service is estimated using a logistic regression model. The second part of the model, generalized linear models (GLMs) with a gamma distribution and a logarithmic relationship, estimates the costs under the condition that positive costs have been incurred. The Akaike information criteria (AIC), which measure the goodness of fit, are used to compare the different models, e.g. log transformation.

The cumulative incidence rate (IR) is presented for each HRU outcome. HRU outcomes are compared using the Poisson regression model or negative binomial regression model and the results are presented as unadjusted and adjusted incidence rate ratios (IRRs) with 95% confidence intervals (CIs). Overdispersion is checked by comparing the Poisson regression and negative binomial regression models using the likelihood ratio test. The best-fitting model is selected.

6.5.2 Quality-adjusted life years

QALYs are a measure that combines HRQoL and life expectancy in one index. In this study, the QALYs gained due to an improvement in quality of life are referred to as QALY weights and are assessed using the EQ-5D utility index.

The EQ-5D is collected at baseline, 6 months and 12 months after randomization. QALYs are estimated individually for each patient as the area under the curve (AUC) using the time-weighted QALY[18]. Total QALYs are estimated as the mean of the time-weighted individual QALYs. QALYs are set to 0 if premature death occurs at that time. In addition, the utility index is estimated using a regression-based adjustment for the utility index at baseline[18]. Incomplete quality of life data are considered missing values under the completely missing values for quality of life are assumed to be missing completely at random (MCAR) or missing at random (MAR) using imputation methods[15]. Missing values for quality of life are calculated using multiple imputation with chained equation (MICE) as in 4.

The AUC using the time-weighted QALY is calculated as follows:

$$time - weighted\ QALY = \frac{1}{12}\sum_{i=0}^{2}\frac{(t_{i+1} - t_i)}{2}(y_i + y_{i+1})$$

y_i is the utility index value at measurements *i*=0, 1, 2 and at time (t_i) = 0, 6, 12 months

6.5.3 Cost-effectiveness analysis

The cost-effectiveness of the intervention is compared with the control group on the basis of the incremental cost-effectiveness ratio (ICER). The ICER is defined as the difference in costs between the two groups divided by the difference in their effects. Thus, the ICER can be interpreted as the additional cost associated with an additional QALY. The ICER is How follows calculated:

$$ICER = \frac{Cost_{IG} - Cost_{KG}}{QALYs_{IG} - QALYs_{KG}}$$

Only direct health care costs are considered in the cost-effectiveness analysis. Costs and health outcomes are not discounted due to the one-year time horizon.

6.5.4 Software

All statistical analyses are performed using Stata MP18 (StataCorp, 2023, College Station, TX, USA).

7. Examples of the presentation of the tables

To comply with the guidelines for presenting scientific results, the data are presented in the following tables.

Table 7.1 Baseline characteristics

Baseline characteristics	In total (n=…)	Intervention group (n=…)	Control group (n=…)
**Age at randomization (years)**			
Mean (SD) [Min, Max]			
**BMI (kg/m^2)** – Mean (SD)			
<30			
≥30			
**Family status**			
Married			
Single			
Divorced / widowed			
**Education**			
low			
Medium			
High			
**ECOG**			
0			
1			
2			
**Metastasis**			
Primary/therapy naïve			
Secondary/therapy received			
**Site of metastasis**			
Cerebral			
Visceral			
Bone			
Multiple			
**Tumor subtype**			
HR+/HER2+			
HR+/HER2-			
HR-/HER2+			
TNBC			
**Therapy at randomization**			
Chemotherapy			
endocrine therapy			
Antibody therapy / hybrid therapy			

Table 7.2 Primary endpoint - fatigue score after 6 months

Study group	Fatigue score after 6 months mean (95% CI)	Mean difference (IG – KG) (95% CI)	p-values
**Linear mixed model**			
IG			
KG			
**Sensitivity analysis**			
IG			
KG			

IG= intervention group, KG= control group

Table 7.3 Secondary endpoints

EORTC	Mean (95%CI)		Mean difference (IG – KG) (95% CI)	p-values
	Intervention group (IG)	Control group (CG)		
**Global Health Status/ QoL**				
baseline				
3 months				
6 months				
9 months				
12 months				
**Functional scales**				
baseline				
3 months				
6 months				
9 months				
12 months				
**Symptom/item scales**				
baseline				
3 months				
6 months				
9 months				
12 months				

Table 7.4 Number of alerts and contacts per patient (intervention group only)

**Number of alerts**	
Mean (SD)	
Median (Min, Max)	
**Number of contacts**	
mean (SD)	
Median (Min, Max)	
**Duration of contact since the alert (hours)**	
Median (Min, Max)	
Immediately	
Within 24 hours	
Within 1 to 3 days	
More than 3 days	
**Symptoms or problem report from the contact**	**n (%)**
Not relevant	
Quality of life/Health status	
Physical function	
Role function	
Emotional function	
Cognitive function	
Social function	
Fatigue	
Nausea/Vomiting	
Pain	
Dyspnea	
Insomnia	
Loss of appetite	
Constipation	
Diarrhea	
Financial difficulties	
Other	

Table 7.5 The EQ-5D utility values by study group and measurement time

Time of measurement	Number of observations	Number of missing values	Mean (SD)	Minimum	Maximum
**Baseline**					
Intervention group					
Control group					
**6 months**					
Intervention group					
Control group					
**12 months**					
Intervention group					
Control group					

Table 7.6 QALY weights based on the EQ-5D by study group

	Intervention group (n=…)	Control group (n=…)	Mean difference (IG – KG) (95% CI)	p-values
Baseline				
6 months				
12 months				
Mean time-weighted QALYs (95% CI)				

QALY=quality-adjusted life year

Table 7.7 Results of the cost-effectiveness analysis (intervention vs. control)

Study group	Total costs (€)	Total QALYs	Incremental costs (€)	Incremental QALY	ICER (€)
Intervention group					
Control group					

8. References

1. Basch E, Deal AM, Kris MG, et al. Symptom Monitoring With Patient-Reported Outcomes During Routine Cancer Treatment: A Randomized Controlled Trial. J Clin Oncol 2016;34(6):557-65. DOI: 10.1200/JCO.2015.63.0830.
2. Denis F, Basch E, Septans AL, et al. Two-Year Survival Comparing Web-Based Symptom Monitoring vs Routine Surveillance Following Treatment for Lung Cancer. JAMA 2019;321(3):306-307. DOI: 10.1001/jama.2018.18085.
3. Aaronson NK, Ahmedzai S, Bergman B, et al. The European Organization for Research and Treatment of Cancer QLQ-C30: A Quality-of-Life Instrument for Use in International Clinical Trials in Oncology. JNCI: Journal of the National Cancer Institute 1993;85(5):365-376. DOI: 10.1093/jnci/85.5.365.
4. Petersen MA, Aaronson NK, Arraras JI, et al. The EORTC CAT Core-The computer adaptive version of the EORTC QLQ-C30 questionnaire. Eur J Cancer 2018;100:8-16. DOI: 10.1016/j.ejca.2018.04.016.
5. Cocks K, King MT, Velikova G, et al. Evidence-based guidelines for interpreting change scores for the European Organisation for the Research and Treatment of Cancer Quality of Life Questionnaire Core 30. European Journal of Cancer 2012;48(11):1713-1721. DOI: https://doi.org/10.1016/j.ejca.2012.02.059.
6. Cortes J, Hudgens S, Twelves C, et al. Health-related quality of life in patients with locally advanced or metastatic breast cancer treated with eribulin mesylate or capecitabine in an open-label randomized phase 3 trial. Breast Cancer Res Treat 2015;154(3):509-20. DOI: 10.1007/s10549-015-3633-7.
7. nQuery. Sample size and power calculation. Cork: Statistical Solutions Ltd; 2017.
8. UNESCO. International Standard Classification of Education. ISCED 1997. Paris2006.
9. ICH. Integrated Addendum to ICH E6(R1); 2016. Guideline for Good Clinical Practice E6(R2). Available from: https://database.ich.org/sites/default/files/E6_R2_Addendum.pdf. Accessed 29 November 2023.
10. Grobler AC, Lee KJ, Wong A, Currow DC, Braat S. Handling Missing Data and Drop Out in Hospice/Palliative Care Trials Through the Estimand Framework. J Pain Symptom Manage 2022;63(4):e431-e439. DOI: 10.1016/j.jpainsymman.2021.12.022.
11. Borm GF, Fransen J, Lemmens WA. A simple sample size formula for analysis of covariance in randomized clinical trials. J Clin Epidemiol 2007;60(12):1234-8. (In eng). DOI: 10.1016/j.jclinepi.2007.02.006.
12. Maradit Kremers H, Devick KL, Larson DR, Lewallen DG, Berry DJ, Crowson CS. Competing Risk Analysis: What Does It Mean and When Do We Need It in Orthopedics Research? J Arthroplasty 2021;36(10):3362-3366. DOI: 10.1016/j.arth.2021.04.015.

13. Ratitch B, O'Kelly M, Tosiello R. Missing data in clinical trials: from clinical assumptions to statistical analysis using pattern mixture models. Pharm Stat 2013;12(6):337-47. DOI: 10.1002/pst.1549.
14. Bell ML, Floden L, Rabe BA, et al. Analytical approaches and estimands to take account of missing patient-reported data in longitudinal studies. Patient Relat Outcome Meas 2019;10:129-140. DOI: 10.2147/PROM.S178963.
15. Rubin DB. Multiple Imputation for Nonresponse in Surveys. New York, NY: John Wiley & Sons, Inc, 1987.
16. Glick HA, Doshi JA, Sonnad SS, Polsky D. Economic evaluation in clinical trials: OUP Oxford, 2014.
17. Manning WG, Basu A, Mullahy J. Generalized modeling approaches to risk adjustment of skewed outcomes data. Journal of health economics 2005;24(3):465-488.
18. Manca A, Hawkins N, Sculpher MJ. Estimating mean QALYs in trial-based cost-effectiveness analysis: the importance of controlling for baseline utility. Health Econ 2005;14(5):487-96. DOI: 10.1002/hec.944.

9. Abbreviations

CAT: Computer Adaptive Testing;

CI: Confidence interval;

EORTC: European Organisation for Research and Treatment of Cancer;

GCP: Good Clinical Practice;

HER: Human epidermal growth factor receptor;

HR: Hormone receptor;

HRQoL: Health-related quality of life;

mITT: Modified intention-to-treat;

MAR: Missing at random;

MBC: Metastatic breast cancer;

MI: Multiple imputation;

MAR: Missing at random;

MCAR: Missing completely at random;

MNAR: Missing not at random;

PRO: Patient-reported outcome;

SAP: Statistical analysis plan;

SD: Standard deviation;

TPA: Tipping point analysis

Original Protocol and SAP

(German version)

Studienprotokoll

PRO B – Patient Reported Outcomes bei Brusterkrankungen

Neue Wege in der Versorgung bei metastasiertem Brustkrebs

Kurztitel: PRO B

Indikation: metastasierter Brustkrebs

Intervention: Patient-Reported Outcomes (PROs)

DRKS-ID/ ID U.S. National Library of Medicine: Wird ergänzt

Förderung: Innovationsausschuss beim G-BA

Inhaltsverzeichnis

Synopse **1**

Verantwortlichkeiten **2**

1 Rationale **3**

1.1 Wissenschaftlicher Hintergrund 3

1.2 Begründung für die Studie 4

1.3 Nutzen-Risiko-Abwägung 4

2 Ziele der Studie **4**

2.1 Primäres Ziel und primärer Endpunkt 5

2.2 Sekundäres Ziel und sekundäre Endpunkte 5

3 Studiendesign und Zeitplan **5**

3.1 Studiendesign 5

3.2 Zeitplan 6

4 Rekrutierung **6**

4.1 Fallzahl 6

4.2 Studienzentren 7

4.3 Einschlusskriterien 7

4.4 Ausschlusskriterien 7

4.5 Abbruchkriterien 7

5 Intervention **7**

5.1 Beschreibung der Intervention 7

5.2 Risiken der Intervention 8

5.3 Randomisation 8

5.4 Verblindung 8

5.5 Mögliche Confounder 9

6 Studienablauf **9**

6.1 Beschreibung des Studienablaufs 9

6.2 Geplante Behandlung nach Studienende 10

7 Statistische Vorgehensweise **11**

7.1 Fallzahlkalkulation 11

7.2 Variablen 11

7.3 Pretest 12

7.4 Statistische Methoden 12

8 Datenmanagement **14**

8.1 Datenerhebung 14

8.2 Datenverarbeitung, Qualitätskontrolle, Monitoring 15

8.3 Datenspeicherung und Datenschutz 16

9 Ethische und juristische Aspekte **17**

9.1 Juristische Grundlage 17
9.2 Ethikkommission 17
9.3 Patientinneninformation und Einwilligungserklärung 17
9.4 Aufwandsentschädigung 17
9.5 Versicherung 17
10 Administrative Aspekte 18
10.1 Studienfinanzierung 18
10.2 Studienberichte 18
10.3 Studienregistrierung 18
10.4 Publikationen 18
11 Signatur der Studienleitung 18
Literaturverzeichnis 19
Abkürzungsverzeichnis A
Anlagen B
Anlage 1. Information für Patientinnen, Datenschutzinformation, Einwilligungserklärung C
Anlage 2. Datenschutzkonzept D

Synopse

Titel	**PRO B** – Neue Wege in der Versorgung bei metastasiertem Brustkrebs
Indikation	Metastasierter Brustkrebs
Studienziele	Evaluation der Auswirkungen eines intensivierten Patient-Reported Outcome (PRO)-Monitorings auf Lebensqualität, Gesamtüberleben und Behandlungskosten speziell für Patientinnen mit metastasiertem Mammakarzinom<br><br><u>Primärer Endpunkt:</u><br>Fatigue (EORTC QLQ-C30, QLQ-FA12, EORTC CAT) nach 12 und ggf. 24 Monaten<br><br><u>Sekundäre Endpunkte:</u><br>Sekundäre Ziele sind die Verbesserung des Gesamtüberlebens sowie die Reduktion ungeplanter ärztlicher Konsultationen, Hospitalisierungen und Rettungsstellenaufenthalte nach 6, 12 und ggf. 24 Monaten
Studiendesign	Bei PRO B handelt sich um eine prospektive, multizentrische, randomisierte zweiarmige Interventionsstudie zur Implementierung und Evaluation von PRO-Messungen bei Patientinnen mit metastasiertem Mammakarzinom.<br><br><u>Interventionsarm</u>: wöchentliche PRO-Befragung mit Kontaktaufnahme und ggf. Maßnahmen durch das behandelnde Team bei Verschlechterung der Werte<br><br><u>Kontrollarm</u>: PRO-Befragung in dreimonatigem Abstand ohne Kontaktaufnahme
Stichprobe	<u>Einschlusskriterien:</u><br>- Weiblich<br>- Gesetzlich krankenversichert (BARMER, DAK Gesundheit, BKK·VBU)<br>- Diagnose metastasierter Brustkrebs<br>- Behandlung in einem teilnehmenden Brustkrebszentrum<br>- Vollendung des 18. Lebensjahres sowie Einwilligungsfähigkeit<br>- Erhalt einer medikamentösen Behandlung aufgrund eines metastasierten Mammakarzinoms<br>- Lebenserwartung bei Studieneinschluss von mehr als 3 Monaten<br>- ECOG 0-2<br>- Bereitschaft, an einer wöchentlichen, online-basierten PRO-Befragung teilzunehmen<br>- Zugang zu Internet (Smartphone, Tablet oder E-Mail)<br><br><u>Ausschlusskriterien:</u> keine aktive Tumortherapie (Comfort Care)<br><br><u>Abbruchkriterien</u>: Patientin zieht Einwilligung zur Studienteilnahme zurück
Stichprobengröße	500 Probandinnen pro Studienarm
Projektdauer	36 Monate
Statistische Vorgehensweise	- Primäre Hypothese: ANCOVA-Modell<br>- Sekundäre Hypothesen: z. B. Zeit-Ereignis-Modelle, ANCOVA, logistische Regression, Cox Regression<br>- Subgruppen-Analysen<br>- Gesundheitsökonomische Analysen
Anzahl der Studienzentren	Bis zu 40 Brustkrebszentren
Studienfinanzierung	Innovationsausschuss des Gemeinsamen Bundesausschusses (Fördernummer: 01NVF19013)

Verantwortlichkeiten

**Studienleitung**	**Dr. med. Maria Margarete Karsten**<br>Klinik für Gynäkologie mit Brustzentrum<br>Charité Campus Mitte<br>Charité – Universitätsmedizin Berlin<br><br>Charitéplatz 1<br>10117 Berlin<br><br>+49 (0)30 450 664 279<br>maria-margrete.karsten@charite.de
**Evaluation**	**PD Dr. rer nat. phil. Ulrike Grittner**<br>Institut für Biometrie und Klinische Epidemiologie<br>Charité - Universitätsmedizin Berlin<br>Charité Campus Mitte<br><br>Charitéplatz 1<br>10117 Berlin<br><br>+49 (0)30 450 562 174<br>ulrike.grittner@charite.de
**Psychometrische Testung**	**Prof. Dr. med. Matthias Rose**<br>Medizinische Klinik m. S. Psychosomatik<br>Charité - Universitätsmedizin Berlin<br><br>Charitéplatz 1<br>10117 Berlin<br><br>+49 (0)30 450 553 001<br>matthias.rose@charite.de
**Technische Umsetzung**	**Dr. med. Julia Ferencz**<br>OnkoZert GmbH<br><br>Gartenstraße 24<br>89231 Neu-Ulm<br><br>j.ferencz@onkozert.de
**Disseminierung und Unterstützung bei der Rekrutierung der Studienzentren**	**Clara Breidenbach**<br>Deutsche Krebsgesellschaft e. V.<br><br>Kuno-Fischer-Straße 8<br>14057 Berlin<br><br>+49 (0)30 322 932 934<br>breidenbach@krebsgesellschaft.de
**Vertrag Besondere Versorgung und Routinedaten**	BKK·VBU<br>BARMER<br>DAK Gesundheit
**Studienzentren**	Brustkrebszentren
**Projektförderung**	Innovationsausschuss beim Gemeinsamen Bundesausschuss

1 Rationale

1.1 Wissenschaftlicher Hintergrund

Brustkrebs ist weltweit die häufigste Krebserkrankung und krebsbedingte Todesursache bei Frauen. Trotz aller Fortschritte in Forschung und klinischer Praxis entwickeln auch heute noch bis zu 30% der Betroffenen Metastasen (1). Laut dem Zentrum für Krebsregisterdaten des Robert-Koch-Instituts sterben damit allein in Deutschland jährlich mehr als 18.000 Patient*innen an den Folgen eines metastasierten Mammakarzinoms (2). Mit dem Auftreten von Metastasen ändern sich nicht nur die Lebensumstände der Patient*innen, sondern auch das therapeutische Vorgehen grundlegend. Im Vordergrund der Behandlung steht nun nicht mehr die Heilung als vielmehr die Kontrolle des Tumors und die damit verbundene Verlängerung des Überlebens unter gleichzeitiger Kontrolle der Therapienebenwirkungen. Ziel ist es, die bestmögliche Lebensqualität über einen möglichst langen Zeitraum aufrecht zu erhalten.

Das Gesamtüberleben der Patient*innen mit metastasiertem Brustkrebs hat sich durch modernere Therapieoptionen über die letzten Jahrzehnte deutlich verbessert und lag mit 38 Monaten in 2010 bereits 17 Monate über dem medianen Gesamtüberleben von 1990 (3). Die Lebenserwartung im Falle einer metastasierten Brustkrebs-Erkrankung hängt jedoch wesentlich von den biologischen Eigenschaften des zugrundeliegenden Tumors ab. Caswell-Jin et al. zeigten in einer Metaanalyse ein medianes Überleben von 57 Monaten für Patientinnen mit Hormonrezeptor-positivem Tumor verglichen mit einer Überlebenszeit von lediglich 33 Monaten bei triple-negativen Mammakarzinomen (3). Mit dem Ziel, den Zeitraum des Überlebens zu verlängern, unterziehen sich die meisten Patient*innen während dieser Zeit einer systemischen Tumortherapie. Anders als in der adjuvanten Situation, in der sich Therapieentscheidungen relativ einfach an den Standards der Fachgesellschaften orientieren können, müssen sie in der metastasierten Situation jedoch auf viel individuellerer Basis getroffen werden. Unter Berücksichtigung der medizinischen Vorgeschichte, der aktuellen Beschwerden und vor allem des Patient*innenwunsches ist der Entscheidungsprozess dahingehend zeitaufwendig und muss aufgrund des fortschreitenden Krankheitsverlaufs immer wieder neu hinterfragt werden. An dieser Stelle sind Informationen, die über sogenannte Patient-Reported Outcomes (PROs) erfasst werden, hilfreich, um die bestmöglichen Therapieentscheidungen treffen zu können. PROs ermöglichen die Bewertung gesundheitsbezogener Aspekte (wie Krankheitssymptome, psychosoziale Belastungen und Lebensqualität) aus Patient*innensicht ohne zwischengeschaltete Interpretation durch eine andere Person (z. B. Arzt oder Ärztin) (4). Sie tragen damit zur Verbesserung

- der frühzeitigen Erkennung eines möglichen Krankheitsfortschritts,
- der Einschätzung Therapie-assoziierter Toxizität und
- der Detektion einer Verschlechterung der sogenannten Health-related Quality of Life (HRQoL)

bei.

Die besondere Herausforderung der Bewertung des Schweregrades bestimmter Krankheitssymptome oder Medikamentennebenwirkungen wird von den betreuenden Ärzt*innen häufig unterschätzt (5-7). Dies betrifft insbesondere unspezifische Symptome wie Konstipation, Appetitlosigkeit und Fatigue, obwohl letztere oftmals erster klinischer Indikator für einen Krankheitsprogress sein kann (8). In diesem Zusammenhang sind PRO-Messungen hilfreich, da sie den Behandelnden ermöglichen, sich binnen kürzester Zeit ein sehr differenziertes Bild über den Gesundheitszustand ihrer Patient*innen zu verschaffen. Besonders in der metastasierten Situation, in der die Patient*innen so wenig Zeit wie möglich in der Klinik verbringen sollen und die Therapien daher in der Regel ambulant durchgeführt werden, ermöglichen regelmäßige PRO-Messungen ein Echtzeit-Monitoring von Symptomen und Nebenwirkungen sowie zeitnahe Interventionen der Behandelnden. Müller et al. berichten, dass schon eine geringe Verschlechterung der HRQoL bei Patientinnen mit metastasiertem Mammakarzinom mit einem Progress der Erkrankung assoziiert ist (9). Die Möglichkeit, mittels PRO-Messungen eine solche Verschlechterung und damit ggf. einen Krankheitsprogress frühzeitig festzustellen, kann den Betroffenen weitere Zyklen einer nicht mehr effektiven Tumortherapie ersparen.

1.2 Begründung für die Studie

Bei einem durchschnittlichen Gesamtüberleben von 38 Monaten nach Metastasierung und jährlich 18.570 an Brustkrebs verstorbenen Frauen (2,3), kann davon ausgegangen werden, dass in Deutschland derzeit mehr als 60.000 Patientinnen mit metastasiertem Mammakarzinom leben. Die Behandlung dieser schwer erkrankten Frauen stellt sowohl aus medizinischer, aber auch aus psychosozialer und ökonomischer Sicht eine Herausforderung für das gesamte klinische Team dar. Es bestehen keine kurativen Behandlungsoptionen, sodass ein Krankheitsprogress im Verlauf unausweichlich ist. Zudem sinkt mit jeder Änderung des Therapieschemas bei refraktärer Erkrankung die Wahrscheinlichkeit des Ansprechens – sie kann bei vorbehandelten Patientinnen weniger als 15% betragen (10). Aus psychosozialer Sicht werden aufgrund des hohen Maßes krankheitsbezogener psychischer und emotionaler Belastungen erhebliche Betreuungs- und Vorsorgemaßnahmen notwendig (11). Zudem werden die Kosten der Behandlung eines metastasierten Mammakarzinoms derzeit auf jährlich etwa 100.000 US Dollar pro Patientin geschätzt (12, 13). Die Kombination aus hoher Krankheitslast der Betroffenen, hoher Belastung des Behandlungsteams aufgrund der Komplexität der Erkrankung und der ebenfalls hohen finanziellen Erfordernisse machen Neuerungen in der Versorgung der an einem metastasierten Mammakarzinom Erkrankten dringend notwendig.

Basch et al. konnten in einer richtungsweisenden monozentrischen Studie an 766 Patient*innen mit metastasierter Tumorerkrankung zeigen, dass ein intensiviertes PRO-Monitoring die Raten ungeplanter Rettungsstellenaufenthalte und Hospitalisierungen verringern, vor allem aber auch die Lebensqualität und sogar das Gesamtüberleben der Studienteilnehmer*innen signifikant verbessern konnte (HR: 0,83; 95%CI: 0,70-0,99; p=0,04)(14, 15). Kurz darauf konnten die Vorteile des intensivierten PRO-Monitorings in einer weiteren Studie an metastasierten Lungenkrebspatient*innen bestätigt werden. In dieser Studie zeigten Denis et al., dass das Gesamtüberleben der mittels PROs betreuten Patient*innen gegenüber der Kontrollgruppe um etwa acht Monate verlängert werden konnte (HR: 0,50; 95%CI: 0,31-0,81; p=0,005)(16).

Unserer Kenntnis nach gibt es bisher keine Untersuchungen zu den Auswirkungen eines intensivierten PRO-Monitorings auf Lebensqualität, Gesamtüberleben und Behandlungskosten speziell für Patientinnen mit metastasiertem Mammakarzinom. Durch PRO B soll daher geprüft werden, ob die Ergebnisse der zuvor zitierten Autor*innen auf die in Deutschland übliche Versorgung dieser spezifischen Patientinnengruppe übertragbar sind. Um im Falle positiver Ergebnisse eine einfache und kostengünstige Übertragung in die Regelversorgung zu ermöglichen, werden hierbei bereits vorhandene IT-Elemente der Zertifizierungsinfrastruktur der Deutschen Krebsgesellschaft e. V. (DKG) und OnkoZert genutzt. PRO B erweitert daher gleichsam eine schon seit langem etablierte, flächendeckend und zentrumsübergreifend standardisierte Erhebung von Gesundheitsdaten, um somit eine bessere Einschätzung des Krankheitsverlaufs metastasierter Mammakarzinome und damit verbundener Auswirkungen auf die Lebensqualität der betroffenen Patientinnen zu ermöglichen.

1.3 Nutzen-Risiko-Abwägung

Ein studienspezifisches gesundheitliches Risiko ist nicht zu erwarten, da keine von der üblichen klinischen Routine abweichende spezifische Behandlung durch die Studie vorgegeben wird. Der angenommene Nutzen für die teilnehmenden Patientinnen besteht in einem individuellen Monitoring der Lebensqualität und auf dieser Grundlage veranlasste Maßnahmen bei schlechter Lebensqualität (Eigennutzen). Bei einer positiven Evaluation der Intervention besteht der Nutzen für zukünftige Patientinnen (Gruppennutzen) in einer individualisierten und Patientinnen-zentrierten Versorgungsform.

2 Ziele der Studie

Mit PRO B wird unter Berücksichtigung der zur Verfügung stehenden Ressourcen eine neue, individualisierte und Patientinnen-zentrierte Versorgungsform geschaffen und evaluiert. Die unter Punkt 5. beschriebene Intervention wird während des einjährigen Rekrutierungszeitraums und des folgenden einjährigen Interventionszeitraums durchgeführt. Dementsprechend beträgt die Interventionsdauer mindestens 12 und maximal 24 Monate. Die unten aufgeführten Outcomes (nach 6 und 12 Monaten)

liegen zum Abschluss des Rekrutierungs- und Interventionszeitraums für *alle* eingeschlossenen Probandinnen (abzüglich des Dropouts) vor. Sie werden jedoch auch für die späteren Messzeitpunkte (bis zu max. 24 Monaten nach Einschluss) jeweils mit kleinerem n analysiert, um den PRO-Verlauf für metastasierte Brustkrebspatientinnen über einen möglichst langen Zeitraum abbilden zu können

2.1 Primäres Ziel und primärer Endpunkt

Studienziele sind die Reduktion krankheitsbedingter Morbidität sowie die Verbesserung der Lebensqualität von Patientinnen mit metastasiertem Mammakarzinom. Als primärer Endpunkt ist daher die tumorbedingte Fatigue anhand der Fatigue-Skalen des Quality of Life Questionnaire Core Modules der European Organisation for Research and Treatment of Cancer und des ergänzenden Fatigue Modules (EORTC QLQ-C30, EORTC QLQ-FA12) sowie studienspezifischer Items aus der EORTC CAT-Itemdatenbank 12 Monate nach Einschluss definiert.

2.2 Sekundäres Ziel und sekundäre Endpunkte

Sekundäre Ziele sind die Verbesserung des Gesamtüberlebens sowie die Reduktion ungeplanter ärztlicher Konsultationen, Hospitalisierungen und Rettungsstellenaufenthalte. Somit wurden als sekundäre Endpunkte definiert:

1. Anzahl der Krankenhaus- und Rettungsstellenaufenthalte nach sechs und zwölf Monaten
2. körperliche Funktion nach sechs und zwölf Monaten, anhand des Physical Functioning Scores des EORTC QLQ-C30
3. Fatigue nach sechs Monaten, anhand der Fatigue Scores des EORTC QLQ-C30 und EORTC QLQ-FA12, ergänzende Items aus EORTC CAT
4. Gesamtüberleben bei viszeraler Metastasierung nach zwölf Monaten
5. Gesamtüberleben bei triple-negativem Mammakarzinom nach zwölf Monaten
6. Progressionsfreies Überleben nach zwölf Monaten
7. die gesundheitsbezogene Lebensqualität nach zwölf Monaten, anhand des EORTC QLQ-C30 HRQoL Summenscores
8. Anzahl der Therapieänderungen

3 Studiendesign und Zeitplan

3.1 Studiendesign

Bei PRO B handelt sich um eine prospektive, multizentrische, randomisierte zweiarmige Interventionsstudie zur Implementierung und Evaluation von PRO-Messungen bei Patientinnen mit metastasiertem Mammakarzinom.

Die Patientinnen werden nach Einschluss in die Studie durch Randomisierung entweder dem Interventionsarm oder dem Kontrollarm zugeordnet (siehe Abbildung 1). Abhängig von der Randomisierung werden die Patientinnen wöchentlich (Interventionsarm) oder alle drei Monate (Kontrollarm) mit einer PRO-Messung monitoriert und im Interventionsarm bei Verschlechterung der Werte durch die behandelnden Ärzt*innen kontaktiert (siehe 5.1 Beschreibung der Intervention).

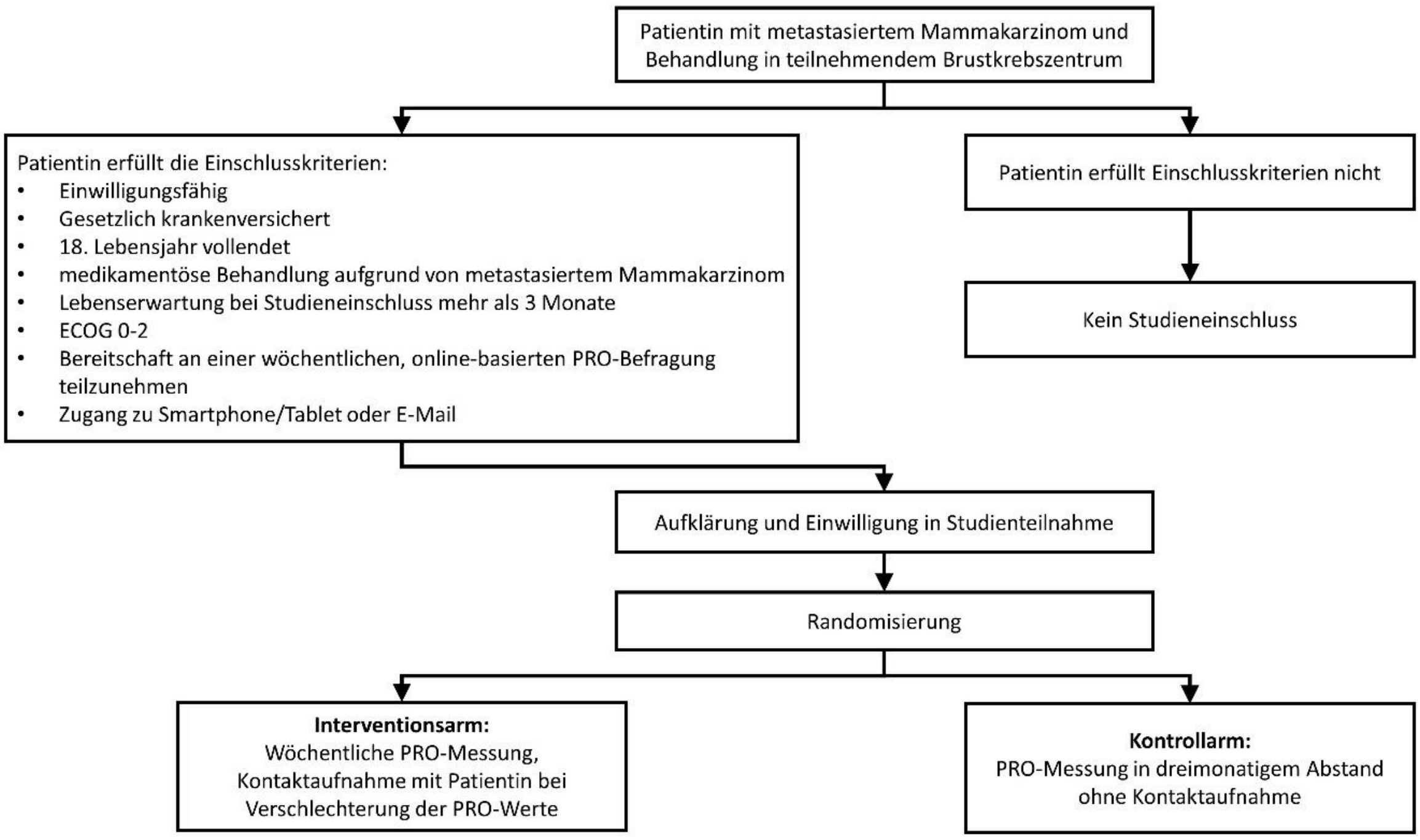

Abbildung 1. Studiendesign

3.2 Zeitplan

Die Gesamtdauer des Forschungsvorhabens beträgt 36 Monate. Die Rekrutierung der Patientinnen ist für April 2021 angesetzt und wird ein Jahr andauern.

**Gesamtdauer**	36 Monate
**Vorbereitungsphase und Studieninitiierung**	Oktober 2020 – März 2021
**Rekrutierung der Patientinnen**	April 2021 – März 2022
**Nachbeobachtung und Monitoring**	April 2022 – März 2023
**Datenauswertung und Dissemination**	April 2023 – September 2023

4 Rekrutierung

4.1 Fallzahl

Die angestrebte Fallzahl beträgt 500 Patientinnen pro Behandlungsarm. Die Fallzahlkalkulation ist in Kapitel 7.1. dargestellt.

Verringerung des Drop-Outs/ Inzentivierung

Um die Drop-out-Rate in der Kontrollgruppe möglichst gering zu halten, erhalten Patientinnen einerseits nach Abschluss des Beobachtungszeitraums einen Ausdruck oder eine Datei ihrer Befragungsdaten, d. h. ihres PRO-Verlaufs, und andererseits werden Patientinnen bei der dreimonatlichen Befragung ausbleibender Beantwortung persönlich kontaktiert. Die Kontaktaufnahme erfolgt hierbei anders als in der Interventionsgruppe lediglich zur Erinnerung an die Beantwortung der PRO-Befragung. Hierzu werden den teilnehmenden Zentren individualisierte Schreiben für die jeweilige Studienpatientin zur Verfügung gestellt mit der Bitte, diese an die Patientin weiterzuleiten. Alternativ hat das teilnehmende Zentrum auch die Möglichkeit, die Patientin bei Nichtbeantwortung des Fragebogens telefonisch zu kontaktieren und zu bitten, diesen im Rahmen der Studienteilnahme auszufüllen. Der PRO-Verlauf kann im Nachhinein als Gesprächsgrundlage mit den Behandelnden dienen und etwaige unbemerkte Auffälligkeiten aufdecken. Mit der Kontaktaufnahme durch die Studienzentren wird auch den

Patientinnen der Kontrollgruppe vermittelt, dass sie ein wesentlicher Teil der Studie sind und sie nicht weniger Aufmerksamkeit seitens ihrer Behandelnden erfahren.

Dem Feedback der Selbsthilfegruppe Mammakarzinom der Charité folgend, wird das intensivierte digitale Monitoring des PRO-Verlaufs als Inzentivierung der Interventionsgruppe für ausreichend erachtet.

4.2 Studienzentren

Insgesamt werden bis zu 40 Brustkrebszentren, verteilt über die Bundesrepublik Deutschland, als Studienzentren rekrutiert. Bei der Rekrutierung der Studienzentren werden die Fallzahlen sowie die Anteile der Patientinnen mit einer Versicherung bei den kooperierenden Krankenkassen BKK·VBU, BARMER, DAK Gesundheit berücksichtigt. Zusätzlich wird bei der Rekrutierung darauf geachtet, dass Studienzentren mit unterschiedlichen Urbanitätsgraden, Trägerschaften sowie Lehrstatus einbezogen werden.

4.3 Einschlusskriterien

Eingeschlossen werden können Frauen

- mit metastasiertem Brustkrebs,
- die in einem an der Studie teilnehmenden Brustkrebszentrum behandelt werden,
- die gesetzlich krankenversichert sind (BARMER, DAK Gesundheit, BKK·VBU),
- die einwilligungsfähig sind,
- die ihr 18. Lebensjahr vollendet haben,
- die eine medikamentöse Behandlung aufgrund des metastasierten Mammakarzinoms erhalten,
- die eine Lebenserwartung bei Studieneinschluss von mehr als 3 Monaten haben,
- die als ECOG 0-2 eingestuft werden,
- die bereit sind, an einer wöchentlichen, online-basierten PRO-Befragung teilzunehmen,
- und die Zugang zu Internet (Smartphone, Tablet oder E-Mail) haben.

4.4 Ausschlusskriterien

Nicht an der Studie teilnehmen können Patientinnen, die keine aktive Tumortherapie erhalten (Comfort Care).

4.5 Abbruchkriterien

Die Studienteilnahme wird abgebrochen, wenn die Patientin die Einwilligung zurückzieht.

5 Intervention

5.1 Beschreibung der Intervention

Die Patientinnen in der neuen Versorgungsform erhalten einmal wöchentlich (immer am gleichen Wochentag) eine Aufforderung via App oder per E-Mail, die PRO-Befragung durchzuführen. Analog zu den Zielparametern der Studie basiert das Interventionskonzept auf der Operationalisierung der gesundheitsbezogenen Lebensqualität des EORTC QLQ-C30 und QLQ-FA12. Der QLQ-C30 umfasst fünf Funktionsdimensionen (Physical Function, Role Function, Emotional Function, Cognitive Function, Social Function) und 9 Symptomskalen (Fatigue, Nausea, Pain, Dyspnoe, Insomnia, Appetite loss, Constipation, Diarrhoea, Financial Difficulties). Zusätzlich definiert der FA12 drei Aspekte der Fatigue (Physical Fatigue, Emotional Fatigue = Depressivität, Cognitive Fatigue = kognitive Funktionsfähigkeit) und daraus folgende Belastungen (Daily activities, Social sequlae). Die benannten Instrumente werden durch studienspezifische Items aus der EORTC CAT-Itemdatenbank ergänzt.

Bei Verschlechterung der PRO-Scores im individuellen longitudinalen Verlauf und/oder im Vergleich zu zuvor definierten Grenzwerten wird ein Alarm generiert, der dem behandelnden Studienzentrum sowie

der PRO B-Studienzentrale angezeigt wird. Klinische Relevanz wird anhand publizierter Informationen zum EORTC QLQ-C30 (z.B. MCID) und anhand von Wahrscheinlichkeiten über tatsächliche Veränderung der latenten Variablen berechnet. Um eine Aggregation mehrerer, klinisch irrelevanter Verschlechterungen über mehrere Wochen zu erkennen, soll außerdem ein Vergleich mit dem Messwert von vor vier Wochen erfolgen. Ein Alarm muss durch die kooperierenden Studienzentren innerhalb von 48 Stunden (bezogen auf Werktage) quittiert und dokumentiert werden. Das Format der Rückmeldung an die behandelnden Zentren wird mit diesen gemeinsam entwickelt. In jedem Fall beinhaltet es explizit die Domänen und das Ausmaß der Veränderung. Auf den Alarm folgt eine Kontaktaufnahme des jeweiligen Zentrums mit der Patientin, um die Verschlechterung ihrer Werte zu spezifizieren und ggf. zu intervenieren. Die auf die Kontaktaufnahme folgende Intervention bzw. Maßnahme durch das Studienzentrum wird durch PRO B nicht vorgegeben. Die Art der Maßnahmen bleibt somit den behandelnden Ärzt*innen überlassen.

Insofern testet die Studie nicht eine spezifische therapeutische Intervention bei Brustkrebspatientinnen, sondern eine zeitnahe individualisierte Kontaktaufnahme und mögliche Intervention verschiedener Ausprägung bei Verschlechterung subjektiver Lebensqualitätsparameter. Der Effekt der Intervention ergibt sich vor allem durch die zeitnahe individualisierte Kontaktaufnahme der behandelnden Ärzt*innen aufgrund alarmierender oder sich alarmierend verschlechternder patientinnenorientierter, lebensqualitätsbezogener Parameter. Diese neue Versorgungsform gilt für alle Patientinnen im Interventionsarm für die gesamte Dauer der Studie (24 Monate), mindestens jedoch für zwölf Monate.

5.2 Risiken der Intervention

Die Intervention besteht aus der Eingabe gesundheitsbezogener Daten in eine App. Gesundheitliche Risiken sind dabei als gering einzuschätzen.

Die Risikofaktoren für das Gelingen der Intervention bestehen in der mangelnden Reaktion/Intervention auf eine Verschlechterung der Befragungswerte seitens des behandelnden Teams. Um eine korrekte und vor allem in zeitlichem Bezug stehende Kontaktaufnahme mit der Patientin sicherzustellen, wird in das Befragungsmodul ein Alarmsystem integriert, das anhand eines hinterlegten Logarithmus bei Verschlechterung der Werte einen Alarm an das behandelnde Brustkrebszentrum und die Studienzentrale meldet. Wird der Alarm nicht innerhalb von 48 Stunden (bezogen auf Werktage) durch das Brustkrebszentrum quittiert und die dadurch ausgelöste Intervention dokumentiert, erfolgt eine telefonische Kontaktaufnahme mit dem Brustkrebszentrum durch die Studienzentrale.

5.3 Randomisation

Die Randomisation erfolgt nach folgenden Strata:

- Zentren (n=40)
- Arten der Metastasierung (4 Gruppen: ausschließlich ossäre Metastasen; viszerale Metastasen; Gehirnmetastasen; multiple Metastasierung)
- histologischer Befund (4 Gruppen: Luminal (HR+/HER-); triple positiv (HR+/HER+); HR-/HER+; Triple negativ)

Durch die hohe Anzahl der Strata kann es zu Unbalanciertheiten bei einer einfachen Randomisation hinsichtlich der Stratifizierungsvariablen zwischen der Interventions- und der Kontrollgruppe kommen. Um diese möglichst gering zu halten, wird unter Verwendung der Minimierungsmethode von Pocock und Simon adaptiv randomisiert (17). Die Randomisierung erfolgt nach Aufklärung und Einwilligung, Download der Applikation und Baseline-Befragung (inkl. soziodemografische Angaben und Komorbiditäten, siehe Kapitel 5.5.).

5.4 Verblindung

Es findet keine Verblindung statt.

5.5 Mögliche Confounder

Als mögliche Confounder werden einmalig im Rahmen der Erstbefragung Daten zu folgenden sozioökonomischen und soziodemographischen Parametern erhoben:

- Familienstand
- Anzahl der Personen im Haushalt
- Pflegebedürftige Angehörige/ minderjährige Kinder (≤14 Jahren) im Haushalt
- Bildungsabschluss/ Berufsabschluss
- Erwerbsstatus
- Einkommen
- Migrationshintergrund

Neben der Brustkrebs-spezifischen Anamnese (familiäre Krebsbelastung) ermöglicht die Befragung über die Smartphone-Applikation auch die Erhebung eventueller Vorerkrankungen. Wir vermuten einen nicht unerheblichen Einfluss folgender Komorbiditäten auf den primären Endpunkt der Studie (Fatigue). Sie müssen daher sowohl in der Interpretation der Messwerte im laufenden Projekt als auch in der abschließenden Auswertung (z. B. als Charlson Komorbiditätsindex (unterstrichene Erkrankungen)) berücksichtigt werden:

- kardiale und kardiovaskuläre Vorerkrankungen (arterielle Hypertonie, koronare Herzerkrankung, Angina pectoris, <u>Myokardinfarkt</u>, <u>Herzinsuffizienz</u>, Vorhofflimmern, Klappenvitien)
- vaskuläre Vorerkrankungen (tiefe Venenthrombose, <u>periphere arterielle Verschlusskrankheit</u>, Lungenarterienembolie)
- cerebrovaskuläre Erkrankungen (<u>TIA</u>, <u>Apoplex</u>)
- hämatologische Vorerkrankungen (Anämie)
- pneumologische Vorerkrankungen (<u>Asthma bronchiale</u>, <u>COPD</u>, Schlafapnoe)
- endokrine/ metabolische Vorerkrankungen (Hyperlipidämie, <u>Diabetes mellitus (therapiebedürftig ohne und mit Endorganschäden)</u>, Hypothyreose, Adipositas)
- rheumatologische Vorerkrankungen (<u>Polymyalgia rheumatica</u>, <u>Lupus erythematodes,</u> <u>rheumatoide Arthritis</u>, <u>Polymyositis</u>)
- psychische Vorerkrankungen (Depression, Angststörung)
- neurologische Vorerkrankungen (Migräne, Krampfanfälle, Gleichgewichtsstörungen, Neuropathie, <u>Hemiplegie</u>, <u>Demenz</u>)
- gastrointestinale Vorerkrankungen (<u>gastrointestinale Ulcera</u>, chronisch entzündliche Darmerkrankung, Divertikulitis, Reflux, Reizdarmsyndrom, <u>leichte Lebererkrankung</u> (Leberzirrhose ohne portale Hypertonie), <u>schwere Lebererkrankung</u> (Leberzirrhose mit portaler Hypertonie, Ösophagusvarizenblutung))
- gynäkologische Vorerkrankungen (Infertilität, Abort, Dys- bzw. Hypermenorrhoe, Endometriose, Ovarialzysten)
- urogenitale Vorerkrankungen (Nephro- bzw. Urolithiasis, <u>Niereninsuffizienz</u>, <u>Dialysepflicht</u>, Miktionsbeschwerden, Harnwegsinfekte, Inkontinenz)
- Infektionserkrankungen (HIV, <u>AIDS</u>)
- onkologische Vorerkrankungen (andere <u>Tumorerkrankungen (mit und ohne Metastasen)</u>, <u>Leukämie</u>, <u>Lymphom</u>)
- Voroperationen
- Suchtmittelanamnese (Alkohol, Nikotin)

6 Studienablauf

6.1 Beschreibung des Studienablaufs

Die Einschlusskriterien erfüllende Patientinnen im Brustkrebszentrum werden von den Prüfärzt*innen über die Studie informiert. Die informierte Einwilligung vorausgesetzt, erfolgt die Aufnahme in die

Studienpopulation. Anschließend wird die App für die PRO-Befragungen auf dem Smartphone/Tablet der Studienteilnehmerin installiert bzw. die Patientinnen erhalten einen E-Mail-Link, über den die Befragung durchgeführt werden kann. Dann erfolgt die Randomisierung der Studienteilnehmerin in einen Studienarm.

Im Interventionsarm wird die Patientin anschließend für mindestens 12 Monate und maximal 24 Monate wöchentlich durch die App aufgefordert, an der PRO-Befragung teilzunehmen. Bei einer Verschlechterung der über die App durch die Patientin rückgemeldeten Werte wird durch eine Web-Anwendung ein Alarm an das Brustkrebszentrum gesendet, welches dann mit der Patientin Kontakt aufnimmt und mögliche Maßnahmen abstimmt. Sollte die Patientin eine wöchentliche PRO-Befragung versäumen, wird das Brustkrebszentrum ebenfalls durch die Web-Anwendung informiert und kann mit der Patientin Kontakt aufnehmen. Alarme werden vom Brustkrebszentrum in der Web-Anwendung quittiert und Kontaktaufnahmen sowie ggf. Maßnahmen dokumentiert. Die PRO-B Studienzentrale hat ebenfalls Zugriff zu der Web-Anwendung und kann Alarme sowie die Reaktion der Brustkrebszentren überprüfen. Sollte ein Brustkrebszentrum innerhalb von 48 Stunden einen Alarm nicht quittiert haben, nimmt die Studienzentrale Kontakt zu dem Brustkrebszentrum auf.

Eine Patientin im Kontrollarm wird alle 3 Monate durch die App aufgefordert, an der PRO-Befragung teilzunehmen. Die Antworten werden ebenfalls unmittelbar elektronisch an die Web-Anwendung übermittelt. Für die Kontrollgruppe erfolgt keine Alarmierung bei Verschlechterung der PRO-Werte und damit auch keine Kontaktaufnahme. Bei Nicht-Beantwortung der Befragung erfolgt eine schriftliche oder telefonische Erinnerung durch das Studienzentrum.

Nach Einwilligung durch die Patientin erfolgt während der Behandlung die Dokumentation klinischer Daten im Tumordokumentationssystem durch das Brustkrebszentrum, wie durch die Zertifizierung vorgesehen. Zusätzliche studienrelevante klinische Angaben, die nicht in den Vorgaben der Zertifizierung berücksichtigt sind, werden durch das Brustkrebszentrum in einem Electronic Case Report Form (eCRF) (Studiendokumentationssoftware TuDoc der Firma OnkoZert) dokumentiert. Darüber hinaus erfolgt die Erhebung der Routinedaten durch die Krankenkasse der Patientin. Abbildung 2 gibt einen Überblick über den individuellen Studienablauf der Patientinnen.

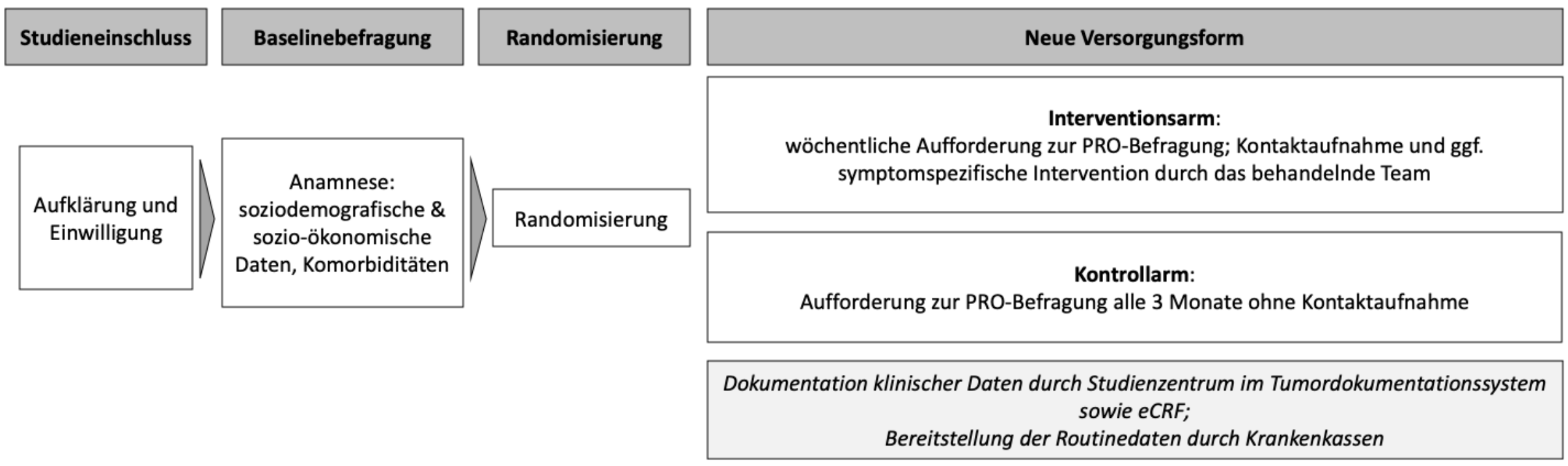

Abbildung 2. Individueller Studienablauf

6.2 Geplante Behandlung nach Studienende

Im Falle positiver Ergebnisse kann die neu geschaffene Versorgungsform aufgrund ihrer besonderen Struktur mit überschaubarem Aufwand in die Regelversorgung übernommen werden. Die Patientinnen erhielten in diesem Zusammenhang nach der Studie, wie auch während des Studienbetriebs, keine von der üblichen klinischen Routine abweichende spezifische Behandlung, würden jedoch bezüglich ihrer Symptome und Lebensqualität weiterhin elektronisch monitoriert. Die Versorgung, die während PRO B evaluiert wird, könnte demnach in sehr ähnlicher Form nach Studienende weitergeführt und für andere Patient*innengruppen ausgeweitet werden. Hierüber wäre zum Beispiel auch die Implementierung und Evaluation einer PRO-gestützten Behandlung und Nachsorge von Brustkrebspatient*innen in der adjuvanten Situation möglich.

7 Statistische Vorgehensweise

7.1 Fallzahlkalkulation

Cocks et al. erstellten Guidelines für die Interpretation längsschnittlicher Veränderungen auf der Fatigue-Skala des EORTC QLQ-C30, nach denen Werte ab durchschnittlich 4 Punkten Verbesserung als kleine Verbesserung und Werte ab 9 Punkten als mittlere Verbesserung einzuschätzen sind (18). In PRO B geht es jedoch um einen Vergleich zwischen Behandlungs- und Kontrollgruppe. Die Effektschätzungen folgen den Angaben von Cocks et al., allerdings sind aufgrund des Zwei-Gruppen-Vergleichs sehr viel größere Streuungsmaße zu erwarten weshalb konservativ eine zu erreichende standardisierte Effektstärke von etwa 0,2 geschätzt wird (mittlere Differenz zum Beispiel 5 Punkte. Gemeinsame Standardabweichung 25,2 Punkte). Eine solche Streuung ist vergleichbar mit den Angaben von Cortes et al. 2015 (19).

Da außerdem die Analyse bestimmter Subgruppen, zum Beispiel Patientinnen mit viszeralen Metastasen, besonders relevant ist, wird eine Fallzahl angestrebt, die auch für Subgruppenanalysen ausreichend groß ist. Die Rekrutierung von 500 Patientinnen pro Gruppe wird als realistisch angesehen. Bei einer erwarteten Drop-Out-Rate von 20% resultiert dies in 400 vollständigen Datensätzen pro Behandlungsarm. Wenn ein minimaler klinisch relevanter Effekt von 5 Punkten Mittelwertunterschied auf der Fatigue-Skala des EORTC QLQ-C30 vorliegt, wie von Cocks et al. (18) berichtet (bei einer gemeinsamen Standardabweichung von 25), hat ein zweiseitiger t-Test mit einem Signifikanzniveau von 5% eine Power von 80,65%, einen Unterschied im Fatigue Score zwischen Interventions- und Behandlungsgruppe mit 400 Patientinnen pro Gruppe nachzuweisen. Diese Berechnung wurde mit nQuery (Version 8.3.1.0) durchgeführt. Die Fallzahl ist somit auch für die Detektion kleiner Effektstärken (0,2 oder größer) ausreichend und trägt der Anzahl relevanter Subgruppen in der Patientinnenpopulation Rechnung.

7.2 Variablen

In der untenstehenden Tabelle werden der primäre Outcome sowie die sekundären Outcomes der Studie aufgeführt. Weitere Variablen, die im Rahmen der der Randomisierung oder als mögliche Confounder relevant sind, werden für die Baseline-Befragung und das Studiendokumentationssystem (eCRF in TuDoc) definiert.

Tabelle 2. Übersicht primärer Outcome und sekundäre Outcomes

**Variable**	**Datenquelle**	**Erhebungszeitpunkt**			
		**Baseline**	**Wöchentlich in Interventionsgruppe; alle 3 Monate in Kontrollgruppe**	**6 Monate**	**12 Monate**
**Primärer Outcome**					
Fatigue Score (EORTC QLQ-C30, EORTC QLQ-FA12, EORTC CAT)	Fragebogen	x	x	x	x
**Sekundäre Outcomes**					
Physical functioning score (EORTC QLQ-C30)	Fragebogen		x	x	x
Health-related Quality of Life Summenscore (EORTC QLQ-C30)	Fragebogen	x	x		x
Anzahl der Krankenhaus- und Rettungsstellenaufenthalte	eCRF/ Krankenkassen-daten			x	x

Anzahl der Therapieänderungen	eCRF/ Krankenkassen-daten				x
Gesamtüberleben bei viszeraler Metastasierung	Tumordoku-mentation/eCRF				x
Gesamtüberleben bei triple negativem Mammakarzinom	Tumordoku-mentation/eCRF				x
Progressionsfreies Überleben	Tumordoku-mentation/eCRF				x

7.3 Pretest

Die Probebefragung (Pretest) wird die für die Verlaufsbeobachtung ausgewählten Messinstrumente beinhalten. Dazu werden teilweise Think-Aloud-Techniken angewandt, teilweise wird den Befragungen ein kurzer Evaluationsfragebogen angehängt, der eine Auswertung zu Befragungsinhalten und Belastungen durch die Beantwortung ermöglicht. Respondentinnen für die Probebefragung werden über Selbsthilfegruppen (z. B. Selbsthilfegruppe Mammakarzinom der Charité) rekrutiert.

7.4 Statistische Methoden

Statistische Analyse der Hypothesen

Die Auswertung der Daten erfolgt durch das Institut für Biometrie und Klinische Epidemiologie (iBiKE) der Charité Universitätsmedizin Berlin.

Analyse der primären Hypothese

Die primäre Fragestellung wird im Rahmen einer Intention to Treat Analyse für alle randomisierten Individuen untersucht. Alle Stratifizierungsvariablen der Randomisation werden als Kovariaten in allen statistischen Modellen berücksichtigt.

Für die Berechnung der Fallzahl wurde ein zweiseitiger t-Test verwendet, obwohl in der Auswertung ein ANCOVA-Modell verwendet werden soll. Es wurde gezeigt, dass dies eine konservative Vorgehensweise für die Schätzung der Fallzahl von ANCOVA-Modellen ist, da ein ANCOVA-Modell mit (1 - 2p)*n Beobachtungen mindestens die gleiche Power wie ein t-Test mit n Beobachtungen hat (20), wobei p der Varianzinflationsfaktor (Korrelation zwischen der *Baseline*-Messung und der *Follow-up*-Messung) ist. Im schlechtesten Fall von p = 0 führt dies zu einer benötigten Fallzahl gleich dem t-Test. Die Analyse erfolgt im ANCOVA-Modell mit der Baseline-Messung sowie Arten der Metastasierung und Histologie als Kovariaten (analog der Stratifizierung der Randomisation). Außerdem wird die Analyse adjustiert für das Alter, Art und Anzahl der Chemotherapien, dem ECOG-Status bei Studieneinschluss, der Computer-Erfahrung und gegebenenfalls für weitere wichtige C*onfounder*. Die Heterogenität zwischen den Studienzentren wird durch *random effects* berücksichtigt (gemischtes lineares Regressions-Modell, *random intercept model*).

Analysen der Sekundären Hypothesen

Alle sekundären Hypothesen werden analog zur primären Hypothese und entsprechend des jeweiligen Outcomes mittels geeigneter statistischer Methoden (z. B. Zeit-Ereignis-Modelle, ANCOVA, logistische Regression, Cox Regression) analysiert. Wie bei der Analyse des primären Outcomes wird auch bei den sekundären Hypothesen für relevante Confounder adjustiert und die Heterogenität zwischen den Studienzentren statistisch angemessen berücksichtigt. Weitere Outcomes und insbesondere verschiedene Subgruppen können im Laufe der Studie noch zusätzlich in die Analyse aufgenommen werden. In diesem Fall werden sie im statistischen Analyseplan (SAP) aufgeführt und explorativ ausgewertet.

Umgang mit fehlenden Werten

Fehlende Werte werden unter der Annahme, dass sie „*missing at random*" (MAR) sind, mittels multipler Imputationsmethoden imputiert. In jedem Fall werden die Gründe für das Fehlen der Werte so gut wie möglich dokumentiert und berichtet.

Statistische Analysen der Subgruppen

Für die statistischen Analysen der Subgruppen wird zunächst im ANCOVA-Modell (Outcome: primäres oder sekundäres Outcome, Kovariablen: jeweilige Baseline Variable, Treatment-Variable, Stratifizierungsvariablen der Randomisation, random Intercept für die Zentren) zusätzlich ein Interaktionsterm der Treatment-Variable mit der Subgruppe eingeschlossen, um erste Hinweise für differentielle Treatment-Effekte in den Subgruppen zu analysieren. Zusätzlich werden aus den Modellen marginale Effektschätzer für jede Subgruppe und 95%-Konfidenzintervalle angegeben. Alle Subgruppenanalysen sind explorativ.

Definition von Subgruppen und Begründung der Bedeutung von Subgruppenanalysen

- **Metastasierung** (sekundär/stattgehabte Therapien, primär/Therapienaivität)
- **Tumorsubtyp** (HR+/HER2+, HR+/HER2–, HR–/HER2+, TNBC)
- **Ort der Metastasierung** (cerebral, viszeral, ossär, multipel)
- **Alter bei Diagnose der Metastasen** (<50, >50)
- **Disease free interval (DFI)** (<5 Jahre, >5 Jahre)
- **Therapie bei Studieneinschluss** (Chemotherapie, endokrine Therapie, Antikörpertherapie/Hybridtherapie)
- **Beziehungsstatus** (Partnerschaft/Ehe, ledig)
- **Einkommen** (Mediansplit)
- **Depression** (ja/ehemals, nein)
- **BMI** (<30, ≥30 kg/m^2)

Patientinnen mit **primär metastasiertem Brustkrebs** haben eine bessere Prognose als Patientinnen mit rezidivierter metastasierter Erkrankung. Dieser Unterschied wird darauf zurückgeführt, dass etwaige adjuvante Systemtherapien der letzteren ggf. zu Resistenzen führen, die bei therapienaiven Patientinnen, also primär Metastasierten nicht vorliegen (21, 22) . Innerhalb der Gruppe der **sekundär metastasierten Tumoren** gilt ein längeres disease free interval (DFI) als prognostisch günstig (21). Einen weiteren Prognose-entscheidenden Faktor stellt das **Alter bei Erstdiagnose** der Metastasen dar. So ist ein jüngeres Alter bei initialem Auftreten von Fernmetastasen mit einer besseren Prognose assoziiert (21). Grund für die schlechtere Überlebensrate der Patientinnen über 50 könnte in diesem Zusammenhang eine geringere Ansprechrate auf Chemotherapien mit ggf. mehr Nebenwirkungen und häufigerer Dosisreduktion sein (22). Weiterhin gilt die **Tumorbiologie** als wesentlicher prognostischer Faktor, da von ihr unter anderem auch das Streuungsmuster der neoplastischen Zellen abhängt. Die beste Prognose wird für Hormonrezeptor (HR) positive (Luminal A), gefolgt von HER2neu (HER) exprimierenden und die schlechteste für Luminal B und triple-negative Tumoren (TNBC) beschrieben (23). Diese Unterschiede werden unter anderem auf unterschiedliche Metastasentropismen zurückgeführt. Bei den Luminaltumoren (HR+/HER-) liegen vordergründig ossäre Metastasen vor, TNBC dagegen weisen weitaus häufiger viszerale und cerebrale Metastasen auf (24), wobei mit letzteren mithin das schlechteste Overall Survival (OS) assoziiert ist (23). Unabhängig von der Hormonrezeptorexpression liegt bei HER2neu positiven Tumoren am häufigsten eine viszerale Metastasierung vor, die ebenfalls mit einem kürzeren Gesamtüberleben als Knochenmetastasen einhergeht (23). HER2neu exprimierende Tumoren stellen zudem einen Risikofaktor für cerebrale Metastasen dar, da die zur Verfügung stehende HER2neu gerichtete Therapie (z. B. Trastuzumab) nicht liquorgängig ist (25).

Einen weiteren mitunter auch prognostischen Faktor stellt die für die PRO B Studie als primärer Endpunkt definierte Fatigue dar. Diese wird durch unterschiedliche Parameter beeinflusst. So zeigen sich z. B. **Therapie-abhängige Unterschiede** des Auftretens und Verlaufs Fatigue-spezifischer

Symptome zwischen Patientinnen nach Chemotherapie und endokriner Therapie (26). Auch **soziodemographische Charakteristika** wie das Einkommen und der Beziehungsstatus der Betroffenen sind mit dem Auftreten Tumor-bedingter Fatigue assoziiert (27). In mehreren Studien wurde außerdem festgestellt, dass ein Zusammenhang von Fatigue mit **Depression** und **mangelnder körperlicher Aktivität/erhöhtem Body-Maß-Index** besteht (28, 29).

Gesundheitsökonomische Auswertung

Über den Studienzeitraum werden die Gesamtkosten der Behandlung über die Krankenkassendaten für alle teilnehmenden Frauen gesammelt und ausgewertet. Dabei wird die jeweilige Beobachtungszeit ebenfalls berücksichtigt (mittlere Behandlungskosten pro Monat pro Patientin).

Um die schiefe Verteilung der Kostendaten zu berücksichtigen, werden für die statistische Analyse generalisierte lineare Modelle mit Gamma-Verteilung und log-link-Funktion verwendet. Weitere statistische Methoden der gesundheitsökonomischen Analyse werden im statistischen Analyseplan zu Beginn der Projektlaufzeit ausgeführt. Kosteneffektivitätsanalysen werden dann durchgeführt, wenn sich die Intervention als überlegen in Bezug auf „quality adjusted life years (QALYs)" erweist. Ein QALY berücksichtigt sowohl Lebenszeit als auch Lebensqualität von Interventionen. QALYs werden auf der Grundlage von Lebensqualitätsfragebögen (EORTC QLQ-C30) berechnet, die zu Baseline und zu verschiedenen folgenden Zeitpunkten während der Projektlaufzeit ausgefüllt werden. Zwischen den Messzeitpunkten wird eine lineare zeitliche Veränderung der Lebensqualität angenommen. QALYs ergeben sich aus der Berechnung der Fläche unter der Kurve. Falls die Intervention zu besseren Lebensqualitätswerten bei gleichzeitig geringeren Kosten führt, gilt sie als kosteneffektiv. Falls es zu zusätzlichen Kosten in der Interventionsgruppe kommt, wird der „incremental cost-effctiveness ratio (ICER)" berechnet, indem die mittleren zusätzlichen Kosten für ein zusätzliches QALY geschätzt werden. Eine Grenze von 50.000 € pro extra QALY wird in Studien oft als Grenzwert für die Kosteneffektivität einer Intervention verwendet. Ist der ICER unter dieser Grenze, gilt die Intervention als kosteneffektiv.

8 Datenmanagement

8.1 Datenerhebung

Die Erhebung der patientinnenseitig berichteten Daten erfolgt mittels eines durch die Firma NeuroSys entwickelten Web-Tools, auf das die teilnehmenden Patientinnen entweder via App oder über einen E-Mail-Link zugreifen können. Bei der initialen Anmeldung bei dem Web-Tool wird eine Identifikationsnummer generiert (Patient Concept ID, 6-stellig bestehend aus Zahlen sowie Groß- und Kleinbuchstaben), durch die die Befragungsdaten pseudonymisiert erhoben werden können und eine Verknüpfung mit den weiteren erhobenen Daten ermöglicht wird. Sowohl die Studienzentrale als auch das Studienzentrum haben ebenfalls Zugriff auf das Web-Tool: Das Web-Tool benachrichtigt das Studienzentrum und die Studienzentrale bei auffälligen Werten (Alarm), woraufhin das Studienzentrum Alarme quittieren sowie ggf. durchgeführte Maßnahmen dokumentieren kann.

Das Studienzentrum erhebt zertifizierungsrelevante klinische und prozessbezogene Patientinnenangaben über das Tumordokumentationssystem. Zusätzlich wird ein eCRF (Studiendokumentationssystem TuDoc) durch OnkoZert programmiert, in dem weitere studienrelevante Angaben durch das Studienzentrum dokumentiert werden. Die Eingaben im Web-Tool (Befragungsdaten, Alarmquittierung, eventuelle Maßnahmen) sowie im Tumordokumentationssystem und dem eCRF werden zu definierten Zeitpunkten (in jedem Fall nach 12 und nach 24 Monaten) vom Studienzentrum jeweils als XML- oder JSON Datei heruntergeladen. Danach wird die Summe aller Projektdaten (klin. Tumordaten, Studiendaten und Befragungsdaten) von jedem einzelnen Zentrum über die OncoBox zusammengeführt, qualitätsgeprüft und als XML- oder JSON-Datei über eine sichere Umgebung (z. B. Citrix-Umgebung) an OnkoZert gesendet. Die XML- bzw. JSON-Datensätze aller Zentren werden dann durch OnkoZert mittels der OncoBox Research zusammengeführt. OnkoZert führt eine weitere Qualitätssicherung der Daten durch (Vollständigkeit etc.) und leitet sie über eine sichere Umgebung (z. B. Citrix-Umgebung) an die PRO B-Zentrale zur Auswertung weiter.

Zusätzlich werden durch die entsprechenden Krankenkassen der Patientinnen Routinedaten zur Verfügung gestellt und über eine Datenintegrationsstelle (siehe Kapitel 8.3) in der zentralen Studiendatenbank mit den anderen Daten verknüpft. Die Abbildungen 3 und 4 fassen den Datenfluss bei PRO B zusammen.

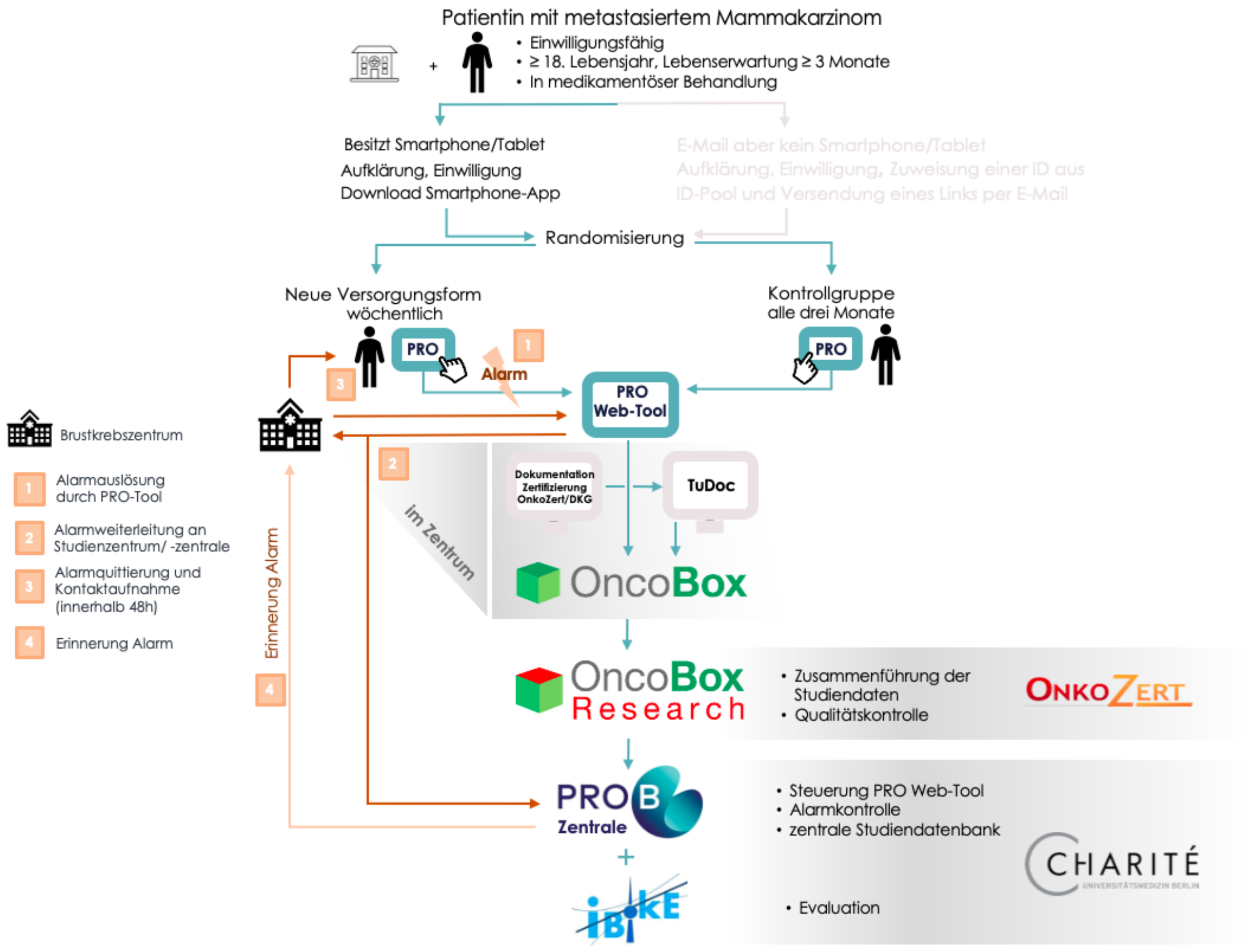

Abbildung 3. Datenfluss. *PRO* Patient-Reported Outcome, *TuDoc* Studiendokumentationssystem, *iBiKE* Institut für Biometrie und Klinische Epidemiologie

8.2 Datenverarbeitung, Qualitätskontrolle, Monitoring

Wenn Daten während der Erhebung nicht verfügbar oder nicht zutreffend sind, wird dies entsprechend vermerkt. In Bezug auf Medikamente und Nebenwirkungen wird die Terminologie der internationalen Referenzlisten (WHO dictionary, MeDRA) beachtet.

Die Studiendokumentation wird durch eine beauftragte Clinical Research Organization (CRO) monitoriert. Dazu führt die CRO pro Studienzentrum ein einmaliges in-person Monitoring durch, bei dem ein stichprobenartiger Quelldatenabgleich samt Überprüfung auf Vollständigkeit, Konsistenz und Plausibilität erfolgt. Sollten Mängel bei der Studiendokumentation festgestellt werden, wird das Studienzentrum zur Behebung dieser aufgefordert. In einem zweiten in-person Monitoring wird dann die Behebung der Dokumentationsmängel überprüft.

Vor dem Versand der Datensätze durch die Studienzentren überprüft die OncoBox die Daten auf Plausibilität und Vollständigkeit (Qualitätssicherung). Zusätzlich führt OnkoZert analog der Kriterien der OncoBox eine Qualitätskontrolle bei Eingang der Datensätze durch.

Die Auswertung der Daten erfolgt durch das Institut für Biometrie und Klinische Epidemiologie (iBiKE) der Charité Universitätsmedizin Berlin.

8.3 Datenspeicherung und Datenschutz

Es wird ein Datenschutzkonzept erstellt, welches extern durch die Kanzlei Beiten Burkhardt begutachtet wird (Anlage 2). Das Gutachten wird vor Studienbeginn vorliegen.

Im Rahmen der PRO B-Studie werden patientinnenseitig berichtete Befragungsdaten sowie durch die Studienzentren dokumentierte klinische Daten erhoben und anschließend mit Routinedaten der Krankenkassen verknüpft.

Die Erhebung der Befragungsdaten erfolgt in pseudonymisierter Form über ein Web-Tool (PatientConcep-ID), das durch die Firma NeuroSys programmiert wird. Die identifizierenden Daten, die zu einer Patient-ID gehören, liegen ausschließlich dem behandelnden Studienzentrum vor und werden nicht an die zentrale Studiendatenbank übermittelt. Die Datenverarbeitung und -speicherung im Rahmen des Web-Tools erfolgt nach dem Datenschutzkonzept. Die in den Zentren erhobenen patientinnenbezogen Daten (Tumordokumentation) werden lokal für mindestens zehn Jahre nach Abschluss gespeichert. Die beteiligten Zentren tragen dafür Sorge, dass nur Befugte personenbezogene Daten zur Kenntnis nehmen können, z. B. durch Zutrittskontrolle durch technische Maßnahmen in gesicherten Räumen, Benutzer*innenkontrolle durch Passwortregelung zur Legitimation und durch automatische Bildschirmsperrung sowie durch Zugriffskontrolle durch Vergabe unterschiedlicher Berechtigungen und differenzierter Zugriffsmöglichkeiten auf einzelne Felder (Vertraulichkeit). Sie gewährleisten zudem, dass personenbezogene Daten während der Verarbeitung unversehrt, vollständig und aktuell bleiben, z. B. indem sie unbefugte oder zufällige Datenverarbeitung durch Sperre des Zugriffs auf Betriebssysteme und/oder Verschlüsselung der Daten vermeiden und durch regelmäßige Kontrolle der Aktualität der Daten prüfen (Integrität); außerdem, dass personenbezogene Daten zeitgerecht zur Verfügung stehen und ordnungsgemäß für die Pseudonymisierung und nachfolgende Übermittlung verarbeitet werden können, z.B. durch klare und übersichtliche Ordnung des Datenbestandes und Vergabe von Zugriffsbefugnissen im erforderlichen Umfang (unter Abwägung gegenüber dem Gebot der Vertraulichkeit) (Verfügbarkeit). Des Weiteren stellen sie sicher, dass jederzeit personenbezogene Daten ihrem Ursprung zugeordnet werden können, z. B. durch Dokumentation der Ursprungsdaten und ihrer Herkunft und Nachvollziehbarkeit der Verarbeitungsschritte (Authentizität). Außerdem sorgen sie dafür, dass festgestellt werden kann, wer wann welche personenbezogenen Daten in welcher Weise verarbeitet hat, z. B. durch Festlegung klarer Zuständigkeiten und Verantwortlichkeiten sowie die Protokollierung der Eingabe und weiteren Verarbeitung der Daten (Revisionsfähigkeit). Die Verfahrensweisen bei der Verarbeitung personenbezogener Daten werden vollständig, aktuell und in einer Weise dokumentiert, dass sie in zumutbarer Zeit nachvollzogen werden können.

Die Routinedaten der Krankenkassen werden am Ende des Projekts in pseudonymisierter Form in die zentrale Studiendatenbank übermittelt (siehe Abbildung 4). Die Datenübermittlung erfolgt über eine Vertrauensstelle sowie ein Datenintegrationszentrum: (1) Den teilnehmenden Studienzentren werden durch die Vertrauensstelle (CHA-THS) Studien-IDs zur Verfügung gestellt, die jeder Patientin zugeorndet werden. (2) Die Studienzentren übermitteln die Studien-ID samt der PatientConcept-ID aus der App oder dem PatientConcep-ID-Pool und der Versichertennummer zurück an die CHA-THS. (3) Die CHA-THS teilt jeder Studien-ID ein neues Krankenkassen(KK)-Pseudonym zu und übermittelt dieses gemeinsam mit der Versichertennummer an die Krankenkassen. (4) In Verbindung mit dem KK-Pseudonym übermitteln die Krankenkassen die Sekundärdaten an das Datenintegrationszentrum. (5) Das Datenintegrationszentrum erhält von der CHA-THS die PatientConcept-ID samt Studien-ID, das KK-Pseudonym und eine neue ID für die Evaluation (Evaluations-ID). (6) Im Anschluss erfolgt die Verknüpfung der KK-Daten mit den anderen Studiendaten aus der PRO B Zentrale. (7) Abschließend wird der vollständige Datensatz mit Evaluations-ID an das evaluierende Institut (iBiKE) weitergeleitet.

Die Übermittlungen der verschiedenen Daten in die zentrale Studiendatenbank (in der Charité ansässig) erfolgen über sichere Umgebungen. Die Daten werden gemäß der geltenden Datenschutzbestimmungen und Datenschutzkonzepte der Charité verarbeitet und gespeichert.

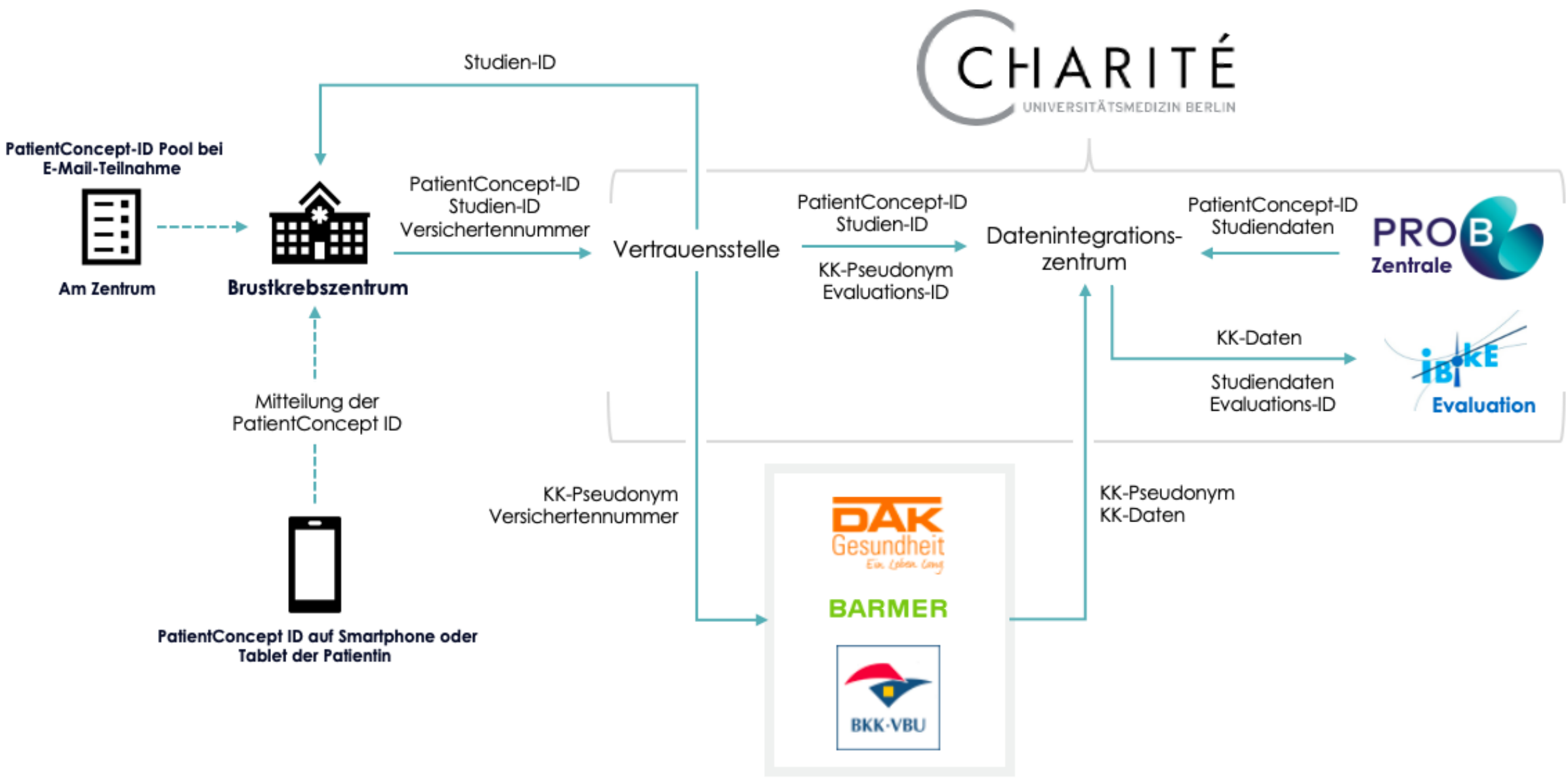

Abbildung 4. Übermittlung der Krankenkassendaten. *KK* Krankenkassen

9 Ethische und juristische Aspekte

9.1 Juristische Grundlage

Die Aufnahme in die Studie erfolgt nach informierter Einwilligung durch die Patientin gemäß anhängender Einwilligungserklärung (siehe Anlage 1). Die Intervention wird auf Rechtsgrundlage eines Vertrags zur Besonderen Versorgung gemäß § 140a SGBV durchgeführt.

9.2 Ethikkommission

Vor Beginn der Studie werden das Studienprotokoll, die Patientinneninformation und Einwilligungserklärung sowie entsprechende weitere Dokumente einer erstvotierenden unabhängigen Ethikkommission vorgelegt. Nach Bestimmung der Studienzentren werden die Dokumente den entsprechenden lokalen Ethikkommissionen zur Beratung vorgelegt (Organisatorische Unterstützung durch CRO). Eine schriftliche positive Abstimmung der Ethikkommissionen ist eine Voraussetzung für den Beginn dieser Studie.

9.3 Patientinneninformation und Einwilligungserklärung

Die Patientinneninformation, Datenschutzinformation und Einwilligungserklärung sind in Anlage 1 einsehbar und wurden nach Vorlage der Ethikkommission der Charité sowie der Medizininformatik-Initiative (MII)[1] erstellt.

9.4 Aufwandsentschädigung

Die teilnehmenden Patientinnen erhalten keine Aufwandsentschädigung.

9.5 Versicherung

Ein studienspezifischer Versicherungsschutz ist nicht vorgesehen.

[1] https://www.medizininformatik-initiative.de/de/medizininformatik-initiative-erhaelt-gruenes-licht-fuer-bundesweite-patienteneinwilligung

10 Administrative Aspekte

10.1 Studienfinanzierung

Die Studie wird durch Förderung des Innovationsauschusses des Gemeinsamen Bundesausschusses (Fördernummer 01NVF19013) finanziert.

10.2 Studienberichte

Das verantwortliche evaluierende Institut (iBiKE) wird gemeinsam mit der Studienleitung innerhalb von 6 Monaten nach Studienende einen Evaluationsbericht vorlegen.

10.3 Studienregistrierung

Die Studie wird vor Beginn der Rekrutierungsphase durch die Studienleitung im Deutschen Register Klinischer Studien (DRKS) und im Studienregister der U. S. National Library of Medicine (Clinicaltrials.gov) registriert.

10.4 Publikationen

Alle die Studie betreffenden Informationen sind vor Publikation vertraulich zu behandeln. Die Dissemination des primären Outcomes der Studie in Form von Präsentation(en) und/oder Publikation(en) sind der Studienleitung (Dr. med. Maria Margarete Karsten) oder einer von ihr gewählten Vertretung vorenthalten. Kooperierende, die maßgebend zur Konzeption und Durchführung der Studie und/oder zur Patientinnenrekrutierung beigetragen haben, können als Co-Autor*innen zur Publikation beitragen. Die Auswahl der Co-Autor*innen als auch die Reihenfolge der Autor*innenschaft werden bei etwaiger Beschränkung des ausgewählten Journals nach wissenschaftlichem Standard und in Rücksprache mit dem Konsortium durch die Studienleitung festgelegt. Weitere, die Studie betreffende Publikationen sind nur mit schriftlichem Einverständnis und nach Sichtung der Studienleitung gestattet, wobei eine Verhinderung oder Verzögerung nur mit triftigen Gründen möglich ist. Der Studienleitung ist daher mindestens einen Monat vor geplanter Einreichung oder Präsentation ein Entwurf des Abstracts bzw. Manuskripts vorzulegen.

Es sind mindestens zwei Publikationen in Peer-reviewed Journals geplant. Zudem soll die Studie in den Mitgliedermagazinen der kooperierenden Krankenkassen und der kooperierenden Fachgesellschaft (Deutsche Krebsgesellschaft e. V.) vorgestellt werden. Weiterhin sind Veröffentlichungen in Patientinnenmagazinen wie Mamma Mia und mamaZone vorgesehen.

11 Signatur der Studienleitung

Berlin, den 30.09.2020

Dr. med. Maria Margarete Karsten

Literaturverzeichnis

1. Mariotto AB, Etzioni R, Hurlbert M, Penberthy L, Mayer M. Estimation of the Number of Women Living with Metastatic Breast Cancer in the United States. Cancer Epidemiol Biomarkers Prev. 2017;26(6):809-15.
2. Robert-Koch-Institut. Krebs in Deutschland für 2015/16. Berlin2019.
3. Caswell-Jin JL, Plevritis SK, Tian L, Cadham CJ, Xu C, Stout NK, Sledge GW, Mandelblatt JS, Kurian AW. Change in Survival in Metastatic Breast Cancer with Treatment Advances: Meta-Analysis and Systematic Review. JNCI cancer spectrum. 2018;2(4):pky062.
4. Guidance for industry: patient-reported outcome measures: use in medical product development to support labeling claims: draft guidance. Health Qual Life Outcomes. 2006;4:79.
5. Basch E, Iasonos A, McDonough T, Barz A, Culkin A, Kris MG, Scher HI, Schrag D. Patient versus clinician symptom reporting using the National Cancer Institute Common Terminology Criteria for Adverse Events: results of a questionnaire-based study. The Lancet Oncology. 2006;7(11):903-9.
6. Di Maio M, Gallo C, Leighl NB, Piccirillo MC, Daniele G, Nuzzo F, Gridelli C, Gebbia V, Ciardiello F, De Placido S, Ceribelli A, Favaretto AG, de Matteis A, Feld R, Butts C, Bryce J, Signoriello S, Morabito A, Rocco G, Perrone F. Symptomatic toxicities experienced during anticancer treatment: agreement between patient and physician reporting in three randomized trials. J Clin Oncol. 2015;33(8):910-5.
7. Di Maio M, Basch E, Bryce J, Perrone F. Patient-reported outcomes in the evaluation of toxicity of anticancer treatments. Nature reviews Clinical oncology. 2016;13(5):319-25.
8. Groenvold M, Petersen MA, Idler E, Bjorner JB, Fayers PM, Mouridsen HT. Psychological distress and fatigue predicted recurrence and survival in primary breast cancer patients. Breast cancer research and treatment. 2007;105(2):209-19.
9. Müller V, Nabieva N, Häberle L, Taran F-A, Hartkopf AD, Volz B, Overkamp F, Brandl AL, Kolberg H-C, Hadji P, Tesch H, Ettl J, Lux MP, Lüftner D, Belleville E, Fasching PA, Janni W, Beckmann MW, Wimberger P, Hielscher C, Fehm TN, Brucker SY, Wallwiener D, Schneeweiss A, Wallwiener M. Impact of disease progression on health-related quality of life in patients with metastatic breast cancer in the PRAEGNANT breast cancer registry. The Breast. 2018;37:154-60.
10. Park IH, Lee KS, Ro J. Effects of second and subsequent lines of chemotherapy for metastatic breast cancer. Clin Breast Cancer. 2015;15(1):e55-62.
11. Raskin W, Harle I, Hopman WM, Booth CM. Prognosis, Treatment Benefit and Goals of Care: What do Oncologists Discuss with Patients who have Incurable Cancer? Clin Oncol (R Coll Radiol). 2016;28(3):209-14.
12. Schwartz KL, Simon MS, Bylsma LC, Ruterbusch JJ, Beebe-Dimmer JL, Schultz NM, Flanders SC, Barlev A, Fryzek JP, Quek RGW. Clinical and economic burden associated with stage III to IV triple-negative breast cancer: A SEER-Medicare historical cohort study in elderly women in the United States. Cancer. 2018;124(10):2104-14.
13. Sorensen SV, Goh JW, Pan F, Chen C, Yardley D, Martín M, Knopf K, Benedict A, Giorgetti C, Iyer S. Incidence-based cost-of-illness model for metastatic breast cancer in the United States. Int J Technol Assess Health Care. 2012;28(1):12-21.
14. Basch E, Deal AM, Kris MG, Scher HI, Hudis CA, Sabbatini P, Rogak L, Bennett AV, Dueck AC, Atkinson TM, Chou JF, Dulko D, Sit L, Barz A, Novotny P, Fruscione M, Sloan JA, Schrag D. Symptom Monitoring With Patient-Reported Outcomes During Routine Cancer Treatment: A Randomized Controlled Trial. J Clin Oncol. 2016;34(6):557-65.
15. Basch E, Deal AM, Dueck AC, Scher HI, Kris MG, Hudis C, Schrag D. Overall Survival Results of a Trial Assessing Patient-Reported Outcomes for Symptom Monitoring During Routine Cancer Treatment. JAMA. 2017;318(2):197-8.
16. Denis F, Basch E, Septans A-L, Bennouna J, Urban T, Dueck AC, Letellier C. Two-Year Survival Comparing Web-Based Symptom Monitoring vs Routine Surveillance Following Treatment for Lung Cancer. JAMA. 2019;321(3):306-7.

17. Pocock SJ, Simon R. Sequential treatment assignment with balancing for prognostic factors in the controlled clinical trial. Biometrics. 1975;31(1):103-15.
18. Cocks K, King MT, Velikova G, de Castro G, Jr., Martyn St-James M, Fayers PM, Brown JM. Evidence-based guidelines for interpreting change scores for the European Organisation for the Research and Treatment of Cancer Quality of Life Questionnaire Core 30. Eur J Cancer. 2012;48(11):1713-21.
19. Cortes J, Hudgens S, Twelves C, Perez EA, Awada A, Yelle L, McCutcheon S, Kaufman PA, Forsythe A, Velikova G. Health-related quality of life in patients with locally advanced or metastatic breast cancer treated with eribulin mesylate or capecitabine in an open-label randomized phase 3 trial. Breast cancer research and treatment. 2015;154(3):509-20.
20. Borm GF, Fransen J, Lemmens WA. A simple sample size formula for analysis of covariance in randomized clinical trials. J Clin Epidemiol. 2007;60(12):1234-8.
21. Dawood S, Broglio K, Ensor J, Hortobagyi GN, Giordano SH. Survival differences among women with de novo stage IV and relapsed breast cancer. Annals of oncology : official journal of the European Society for Medical Oncology. 2010;21(11):2169-74.
22. Largillier R, Ferrero JM, Doyen J, Barriere J, Namer M, Mari V, Courdi A, Hannoun-Levi JM, Ettore F, Birtwisle-Peyrottes I, Balu-Maestro C, Marcy PY, Raoust I, Lallement M, Chamorey E. Prognostic factors in 1,038 women with metastatic breast cancer. Annals of oncology : official journal of the European Society for Medical Oncology. 2008;19(12):2012-9.
23. Kast K, Link T, Friedrich K, Petzold A, Niedostatek A, Schoffer O, Werner C, Klug SJ, Werner A, Gatzweiler A, Richter B, Baretton G, Wimberger P. Impact of breast cancer subtypes and patterns of metastasis on outcome. Breast cancer research and treatment. 2015;150(3):621-9.
24. Soni A, Ren Z, Hameed O, Chanda D, Morgan CJ, Siegal GP, Wei S. Breast Cancer Subtypes Predispose the Site of Distant Metastases. American Journal of Clinical Pathology. 2015;143(4):471-8.
25. Lin NU, Bellon JR, Winer EP. CNS Metastases in Breast Cancer. Journal of Clinical Oncology. 2004;22(17):3608-17.
26. Bower JE, Wiley J, Petersen L, Irwin MR, Cole SW, Ganz PA. Fatigue after breast cancer treatment: Biobehavioral predictors of fatigue trajectories. Health psychology : official journal of the Division of Health Psychology, American Psychological Association. 2018;37(11):1025-34.
27. Bower JE, Ganz PA, Desmond KA, Rowland JH, Meyerowitz BE, Belin TR. Fatigue in breast cancer survivors: occurrence, correlates, and impact on quality of life. J Clin Oncol. 2000;18(4):743-53.
28. Bower JE. Cancer-related fatigue--mechanisms, risk factors, and treatments. Nature reviews Clinical oncology. 2014;11(10):597-609.
29. Andrykowski MA, Donovan KA, Laronga C, Jacobsen PB. Prevalence, predictors, and characteristics of off-treatment fatigue in breast cancer survivors. Cancer. 2010;116(24):5740-8.

Abkürzungsverzeichnis

CRO: Clinical Research Organisation (Klinische Forschungsorganisation);

CSC: Clinical Study Centre (Klinisches Studienzentrum);

DKG: Deutsche Krebsgesellschaft;

ECOG: Eastern Cooperative Oncology Group;

EORTC: European Organisation for Research and Treatment of Cancer;

GCP: Good Clinical Practice (Gute klinische Praxis);

HER2: Human epidermal growth factor receptor 2;

HR: Hormonrezeptor;

HRQoL: Health-related quality of life (Gesundheitsbezogene Lebensqualität);

iBiKE: Institut für Biometrie und Klinische Epidemiologie;

ICER: Incremental cost-effectiveness ratio (Inkrementelles Kosten-Nutzen-Verhältnis);

ICMJE: International Committee of Medical Journal Editors;

MAR: Missing at random;

MBC: Metastatic breast cancer (Metastasierter Brustkrebs);

PRO: Patient-reported outcome;

QALY: Quality-adjusted life year (Qualitätsadjustiertes Lebensjahr);

QoL: Quality of life (Lebensqualität);

SAP: Statistischer Analyseplan

Anlagen

1. Information für Patientinnen, Datenschutzinformation und Einwilligungserklärung
2. Datenschutzkonzept

Anlage 1. Information für Patientinnen, Datenschutzinformation, Einwilligungserklärung

Anlage 2. Datenschutzkonzept

Studienprotokoll

PRO B – Patient Reported Outcomes bei Brusterkrankungen

Neue Wege in der Versorgung bei metastasiertem Brustkrebs

Kurztitel: PRO B

Indikation: metastasierter Brustkrebs

Intervention: Patient-Reported Outcomes (PROs)

DRKS-ID: DRKS00024015 **/ UTN** U1111-1263-4946

Förderung: Innovationsausschuss beim G-BA

Inhaltsverzeichnis

Synopse **1**
Verantwortlichkeiten **3**
1 Rationale **4**
1.1 Wissenschaftlicher Hintergrund 4
1.2 Begründung für die Studie 5
1.3 Nutzen-Risiko-Abwägung 5
2 Ziele der Studie **5**
2.1 Primäres Ziel und primärer Endpunkt 6
2.2 Sekundäres Ziel und sekundäre Endpunkte 6
3 Studiendesign und Zeitplan **6**
3.1 Studiendesign 6
3.2 Zeitplan 7
4 Rekrutierung **7**
4.1 Fallzahl 7
4.2 Studienzentren 8
4.3 Einschlusskriterien 8
4.4 Ausschlusskriterien 8
4.5 Abbruchkriterien 8
5 Intervention **8**
5.1 Beschreibung der Intervention 8
5.2 Risiken der Intervention 9
5.3 Randomisation 9
5.4 Verblindung 10
5.5 Mögliche Confounder 10
6 Studienablauf **11**
6.1 Beschreibung des Studienablaufs 11
6.2 Geplante Behandlung nach Studienende 11
7 Statistische Vorgehensweise **12**
7.1 Fallzahlkalkulation 12
7.2 Variablen 12
7.3 Pretest 13
7.4 Statistische Methoden 13
8 Datenmanagement **16**
8.1 Datenerhebung 16
8.2 Datenverarbeitung, Qualitätskontrolle, Monitoring 16
8.3 Datenspeicherung und Datenschutz 17
9 Ethische und juristische Aspekte **18**

9.1 Juristische Grundlage 18
9.2 Ethikkommission 18
9.3 Patientinneninformation und Einwilligungserklärung 18
9.4 Aufwandsentschädigung 19
9.5 Versicherung 19
10 Administrative Aspekte 19
10.1 Studienfinanzierung 19
10.2 Studienberichte 19
10.3 Studienregistrierung 19
10.4 Publikationen 19
11 Signatur der Studienleitung 19
Literaturverzeichnis 20
Abkürzungsverzeichnis 22
Anlagen 23

Synopse

Titel	**PRO B** – Neue Wege in der Versorgung bei metastasiertem Brustkrebs
Indikation	Metastasierter Brustkrebs
Studienziele	Evaluation der Auswirkungen eines intensivierten Patient-Reported Outcome (PRO)-Monitorings auf Lebensqualität, Gesamtüberleben und Behandlungskosten speziell für Patientinnen mit metastasiertem Mammakarzinom<br><br><u>Primärer Endpunkt:</u><br>Fatigue nach 6 Monaten<br><br><u>Sekundäre Endpunkte:</u><br>• Anzahl der Krankenhaus- und Rettungsstellenaufenthalte nach 6 und 12 Monaten<br>• körperliche Funktion (EORTC Physical Functioning Score) nach 6 und 12 Monaten<br>• Fatigue (EORTC Fatigue Score) nach 12 und ggf. 36 Monaten<br>• Gesamtüberleben bei viszeraler Metastasierung nach 12 Monaten<br>• Gesamtüberleben bei triple-negativem Mammakarzinom nach 12 Monaten<br>• Zeit bis zum ersten Wechsel der systemischen Therapie<br>• die gesundheitsbezogene Lebensqualität (EORTC HRQoL Score) nach 6 und 12 Monaten
Studiendesign	Bei PRO B handelt sich um eine prospektive, multizentrische, randomisierte zweiarmige Interventionsstudie zur Implementierung und Evaluation von PRO-Messungen bei Patientinnen mit metastasiertem Mammakarzinom.<br><br><u>Interventionsarm</u>: wöchentliche PRO-Befragung mit ggf. Kontaktaufnahme und ggf. Maßnahmen durch das behandelnde Team bei Verschlechterung der Werte<br><br><u>Kontrollarm</u>: PRO-Befragung in dreimonatigem Abstand ohne Kontaktaufnahme
Stichprobe	<u>Einschlusskriterien: (nach informierter Einwilligung)</u><br>- Weiblich<br>- Gesetzlich bei einer der folgenden Krankenkassen versichert: BARMER, DAK Gesundheit, BKK· VBU*<br>- Diagnose metastasierter Brustkrebs<br>- Behandlung in einem teilnehmenden Brustkrebszentrum<br>- Vollendung des 18. Lebensjahres sowie Einwilligungsfähigkeit<br>- Erhalt einer medikamentösen Behandlung aufgrund des metastasierten Mammakarzinoms<br>- Lebenserwartung bei Studieneinschluss von mehr als 3 Monaten<br>- ECOG 0-2<br>- Bereitschaft, an einer wöchentlichen, online-basierten PRO-Befragung teilzunehmen<br>- Zugang zum Internet (Smartphone)<br><br><u>Ausschlusskriterien:</u> keine aktive Tumortherapie (Comfort Care)<br><br><u>Abbruchkriterien</u>: Patientin zieht Einwilligung zur Studienteilnahme zurück<br><br>* Eine zeitlich begrenzte Öffnung der Studie für Versicherte anderer Krankenkassen ist nach Zustimmung des Förderers zur Erweiterung der Rechtsgrundlage der Studie möglich
Stichprobengröße	500 Probandinnen pro Studienarm

Projektdauer	48 Monate
Statistische Vorgehensweise	- Primäre Hypothese: ANCOVA-Modell<br>- Sekundäre Hypothesen: z. B. Zeit-Ereignis-Modelle, ANCOVA, logistische Regression, Cox Regression<br>- Subgruppen-Analysen<br>- Gesundheitsökonomische Analysen
Anzahl der Studienzentren	52 Brustkrebszentren
Studienfinanzierung	Innovationsausschuss des Gemeinsamen Bundesausschusses (Fördernummer: 01NVF19013)

Verantwortlichkeiten

**Studienleitung**	**PD Dr. med. Maria Margarete Karsten**<br>Klinik für Gynäkologie mit Brustzentrum<br>Charité Campus Mitte<br>Charité – Universitätsmedizin Berlin<br><br>Chariteplatz 1<br>10117 Berlin<br><br>+49 (0)30 450 664 279<br>maria-margrete.karsten@charite.de
**Evaluation**	**PD Dr. phil. Ulrike Grittner**<br>Institut für Biometrie und Klinische Epidemiologie<br>Charité - Universitätsmedizin Berlin<br>Charité Campus Mitte<br><br>Chariteplatz 1<br>10117 Berlin<br><br>+49 (0)30 450 562 174<br>ulrike.grittner@charite.de
**Psychometrische Testung**	**Prof. Dr. med. Matthias Rose**<br>Medizinische Klinik m. S. Psychosomatik<br>Charité - Universitätsmedizin Berlin<br><br>Chariteplatz 1<br>10117 Berlin<br><br>+49 (0)30 450 553 001<br>matthias.rose@charite.de
**Technische Umsetzung**	**Dr. med. Julia Ferencz**<br>OnkoZert GmbH<br><br>Gartenstraße 24<br>89231 Neu-Ulm<br><br>j.ferencz@onkozert.de
**Disseminierung und Unterstützung bei der Rekrutierung der Studienzentren**	**Clara Breidenbach**<br>Deutsche Krebsgesellschaft e. V.<br><br>Kuno-Fischer-Straße 8<br>14057 Berlin<br><br>+49 (0)30 322 932 934<br>breidenbach@krebsgesellschaft.de
**Vertrag Besondere Versorgung und Routinedaten**	BKK·VBU<br>BARMER<br>DAK Gesundheit
**Studienzentren**	Brustkrebszentren
**Projektförderung**	Innovationsausschuss beim Gemeinsamen Bundesausschuss

1 Rationale

1.1 Wissenschaftlicher Hintergrund

Brustkrebs ist weltweit die häufigste Krebserkrankung und krebsbedingte Todesursache bei Frauen. Trotz aller Fortschritte in Forschung und klinischer Praxis entwickeln auch heute noch bis zu 30% der Betroffenen Metastasen (1). Laut dem Zentrum für Krebsregisterdaten des Robert-Koch-Instituts sterben damit allein in Deutschland jährlich mehr als 18.000 Patient*innen an den Folgen eines metastasierten Mammakarzinoms (2). Mit dem Auftreten von Metastasen ändern sich nicht nur die Lebensumstände der Patient*innen, sondern auch das therapeutische Vorgehen grundlegend. Im Vordergrund der Behandlung steht nun nicht mehr die Heilung als vielmehr die Kontrolle des Tumors und die damit verbundene Verlängerung des Überlebens unter gleichzeitiger Kontrolle der Therapienebenwirkungen. Ziel ist es, die bestmögliche Lebensqualität über einen möglichst langen Zeitraum aufrecht zu erhalten.

Das Gesamtüberleben der Patient*innen mit metastasiertem Brustkrebs hat sich durch modernere Therapieoptionen über die letzten Jahrzehnte deutlich verbessert und lag mit 38 Monaten in 2010 bereits 17 Monate über dem medianen Gesamtüberleben von 1990 (3). Die Lebenserwartung im Falle einer metastasierten Brustkrebs-Erkrankung hängt jedoch wesentlich von den biologischen Eigenschaften des zugrundeliegenden Tumors ab. Caswell-Jin et al. zeigten in einer Metaanalyse ein medianes Überleben von 57 Monaten für Patientinnen mit Hormonrezeptor-positivem Tumor verglichen mit einer Überlebenszeit von lediglich 33 Monaten bei triple-negativen Mammakarzinomen (3). Mit dem Ziel, den Zeitraum des Überlebens zu verlängern, unterziehen sich die meisten Patient*innen während dieser Zeit einer systemischen Tumortherapie. Anders als in der adjuvanten Situation, in der sich Therapieentscheidungen relativ einfach an den Standards der Fachgesellschaften orientieren können, müssen sie in der metastasierten Situation jedoch auf viel individuellerer Basis getroffen werden. Unter Berücksichtigung der medizinischen Vorgeschichte, der aktuellen Beschwerden und vor allem des Patient*innenwunsches ist der Entscheidungsprozess dahingehend zeitaufwendig und muss aufgrund des fortschreitenden Krankheitsverlaufs immer wieder neu hinterfragt werden. An dieser Stelle sind Informationen, die über sogenannte Patient-Reported Outcomes (PROs) erfasst werden, hilfreich, um die bestmöglichen Therapieentscheidungen treffen zu können. PROs ermöglichen die Bewertung gesundheitsbezogener Aspekte (wie Krankheitssymptome, psychosoziale Belastungen und Lebensqualität) aus Patient*innensicht ohne zwischengeschaltete Interpretation durch eine andere Person (z. B. Arzt oder Ärztin) (4). Sie tragen damit zur Verbesserung

- der frühzeitigen Erkennung eines möglichen Krankheitsfortschritts,
- der Einschätzung Therapie-assoziierter Toxizität und
- der Detektion einer Verschlechterung der sogenannten Health-related Quality of Life (HRQoL)

bei.

Die besondere Herausforderung der Bewertung des Schweregrades bestimmter Krankheitssymptome oder Medikamentennebenwirkungen wird von den betreuenden Ärzt*innen häufig unterschätzt (5-7). Dies betrifft insbesondere unspezifische Symptome wie Konstipation, Appetitlosigkeit und Fatigue, obwohl letztere oftmals erster klinischer Indikator für einen Krankheitsprogress sein kann (8). In diesem Zusammenhang sind PRO-Messungen hilfreich, da sie den Behandelnden ermöglichen, sich binnen kürzester Zeit ein sehr differenziertes Bild über den Gesundheitszustand ihrer Patient*innen zu verschaffen. Besonders in der metastasierten Situation, in der die Patient*innen so wenig Zeit wie möglich in der Klinik verbringen sollen und die Therapien daher in der Regel ambulant durchgeführt werden, ermöglichen regelmäßige PRO-Messungen ein Echtzeit-Monitoring von Symptomen und Nebenwirkungen sowie zeitnahe Interventionen der Behandelnden. Müller et al. berichten, dass schon eine geringe Verschlechterung der HRQoL bei Patientinnen mit metastasiertem Mammakarzinom mit einem Progress der Erkrankung assoziiert ist (9). Die Möglichkeit, mittels PRO-Messungen eine solche Verschlechterung und damit ggf. einen Krankheitsprogress frühzeitig festzustellen, kann den Betroffenen weitere Zyklen einer nicht mehr effektiven Tumortherapie ersparen.

1.2 Begründung für die Studie

Bei einem durchschnittlichen Gesamtüberleben von 38 Monaten nach Metastasierung und jährlich 18.570 an Brustkrebs verstorbenen Frauen (2,3), kann davon ausgegangen werden, dass in Deutschland derzeit mehr als 60.000 Patientinnen mit metastasiertem Mammakarzinom leben. Die Behandlung dieser schwer erkrankten Frauen stellt sowohl aus medizinischer, aber auch aus psychosozialer und ökonomischer Sicht eine Herausforderung für das gesamte klinische Team dar. Es bestehen keine kurativen Behandlungsoptionen, sodass ein Krankheitsprogress im Verlauf unausweichlich ist. Zudem sinkt mit jeder Änderung des Therapieschemas bei refraktärer Erkrankung die Wahrscheinlichkeit des Ansprechens – sie kann bei vorbehandelten Patientinnen weniger als 15% betragen (10). Aus psychosozialer Sicht werden aufgrund des hohen Maßes krankheitsbezogener psychischer und emotionaler Belastungen erhebliche Betreuungs- und Vorsorgemaßnahmen notwendig (11). Zudem werden die Kosten der Behandlung eines metastasierten Mammakarzinoms derzeit auf jährlich etwa 100.000 US Dollar pro Patientin geschätzt (12, 13). Die Kombination aus hoher Krankheitslast der Betroffenen, hoher Belastung des Behandlungsteams aufgrund der Komplexität der Erkrankung und der ebenfalls hohen finanziellen Erfordernisse machen Neuerungen in der Versorgung der an einem metastasierten Mammakarzinom Erkrankten dringend notwendig.

Basch et al. konnten in einer richtungsweisenden monozentrischen Studie an 766 Patient*innen mit metastasierter Tumorerkrankung zeigen, dass ein intensiviertes PRO-Monitoring die Raten ungeplanter Rettungsstellenaufenthalte und Hospitalisierungen verringern, vor allem aber auch die Lebensqualität und sogar das Gesamtüberleben der Studienteilnehmer*innen signifikant verbessern konnte (HR: 0,83; 95%CI: 0,70-0,99; p=0,04)(14, 15). Kurz darauf konnten die Vorteile des intensivierten PRO-Monitorings in einer weiteren Studie an metastasierten Lungenkrebspatient*innen bestätigt werden. In dieser Studie zeigten Denis et al., dass das Gesamtüberleben der mittels PROs betreuten Patient*innen gegenüber der Kontrollgruppe um etwa acht Monate verlängert werden konnte (HR: 0,50; 95%CI: 0,31-0,81; p=0,005)(16).

Unserer Kenntnis nach gibt es bisher keine Untersuchungen zu den Auswirkungen eines intensivierten PRO-Monitorings auf Lebensqualität, Gesamtüberleben und Behandlungskosten speziell für Patientinnen mit metastasiertem Mammakarzinom. Durch PRO B soll daher geprüft werden, ob die Ergebnisse der zuvor zitierten Autor*innen auf die in Deutschland übliche Versorgung dieser spezifischen Patientinnengruppe übertragbar sind. Um im Falle positiver Ergebnisse eine einfache und kostengünstige Übertragung in die Regelversorgung zu ermöglichen, werden hierbei bereits vorhandene IT-Elemente der Zertifizierungsinfrastruktur der Deutschen Krebsgesellschaft e. V. (DKG) und OnkoZert genutzt. PRO B erweitert daher gleichsam eine schon seit langem etablierte, flächendeckend und zentrumsübergreifend standardisierte Erhebung von Gesundheitsdaten, um somit eine bessere Einschätzung des Krankheitsverlaufs metastasierter Mammakarzinome und damit verbundener Auswirkungen auf die Lebensqualität der betroffenen Patientinnen zu ermöglichen.

1.3 Nutzen-Risiko-Abwägung

Ein studienspezifisches gesundheitliches Risiko ist nicht zu erwarten, da keine von der üblichen klinischen Routine abweichende spezifische Behandlung durch die Studie vorgegeben wird. Der angenommene Nutzen für die teilnehmenden Patientinnen besteht in einem individuellen Monitoring der Lebensqualität und auf dieser Grundlage veranlasste Maßnahmen bei schlechter Lebensqualität (Eigennutzen). Bei einer positiven Evaluation der Intervention besteht der Nutzen für zukünftige Patientinnen (Gruppennutzen) in einer individualisierten und Patientinnen-zentrierten Versorgungsform.

2 Ziele der Studie

Mit PRO B wird unter Berücksichtigung der zur Verfügung stehenden Ressourcen eine neue, individualisierte und Patientinnen-zentrierte Versorgungsform geschaffen und evaluiert. Die unter Punkt 5. beschriebene Intervention wird während des einjährigen Rekrutierungszeitraums und des folgenden einjährigen Interventionszeitraums durchgeführt. Dementsprechend beträgt die Interventionsdauer mindestens 12 und maximal 24 Monate. Die unten aufgeführten Outcomes (nach 6 und 12 Monaten)

liegen zum Abschluss des Rekrutierungs- und Interventionszeitraums für *alle* eingeschlossenen Probandinnen (abzüglich des Dropouts) vor. Sie werden jedoch auch für die späteren Messzeitpunkte (bis zu max. 24 Monaten nach Einschluss) jeweils mit kleinerem n analysiert, um den PRO-Verlauf für metastasierte Brustkrebspatientinnen über einen möglichst langen Zeitraum abbilden zu können

2.1 Primäres Ziel und primärer Endpunkt

Studienziele sind die Reduktion krankheitsbedingter Morbidität sowie die Verbesserung der Lebensqualität von Patientinnen mit metastasiertem Mammakarzinom. Als primärer Endpunkt ist daher die tumorbedingte Fatigue erhoben mittels studienspezifischer Items aus der CAT-Itembank der European Organisation of Research and Treatment of Cancer (EORTC) 6 Monate nach Einschluss definiert.

2.2 Sekundäres Ziel und sekundäre Endpunkte

Sekundäre Ziele sind die Verbesserung des Gesamtüberlebens sowie die Reduktion ungeplanter ärztlicher Konsultationen, Hospitalisierungen und Rettungsstellenaufenthalte. Somit wurden als sekundäre Endpunkte definiert:

- Anzahl der Krankenhaus- und Rettungsstellenaufenthalte nach sechs und zwölf Monaten
- körperliche Funktion (EORTC Physical Functioning Score) nach sechs und zwölf Monaten
- Fatigue (EORTC Fatigue Score) nach zwölf Monaten
- Gesamtüberleben bei viszeraler Metastasierung nach zwölf Monaten
- Gesamtüberleben bei triple-negativem Mammakarzinom nach zwölf Monaten
- Zeit bis zum ersten Wechsel der systemischen Therapie
- die gesundheitsbezogene Lebensqualität (EORTC HRQoL Score) nach sechs und zwölf Monaten

3 Studiendesign und Zeitplan

3.1 Studiendesign

Bei PRO B handelt sich um eine prospektive, multizentrische, randomisierte zweiarmige Interventionsstudie zur Implementierung und Evaluation von PRO-Messungen bei Patientinnen mit metastasiertem Mammakarzinom.

Die Patientinnen werden nach Einschluss in die Studie durch Randomisierung entweder dem Interventionsarm oder dem Kontrollarm zugeordnet (siehe Abbildung 1). Abhängig von der Randomisierung werden die Patientinnen wöchentlich (Interventionsarm) oder alle drei Monate (Kontrollarm) mit einer PRO-Messung monitoriert und im Interventionsarm bei Verschlechterung der Werte durch das behandelnde Brustkrebszentrum kontaktiert (siehe 5.1 Beschreibung der Intervention).

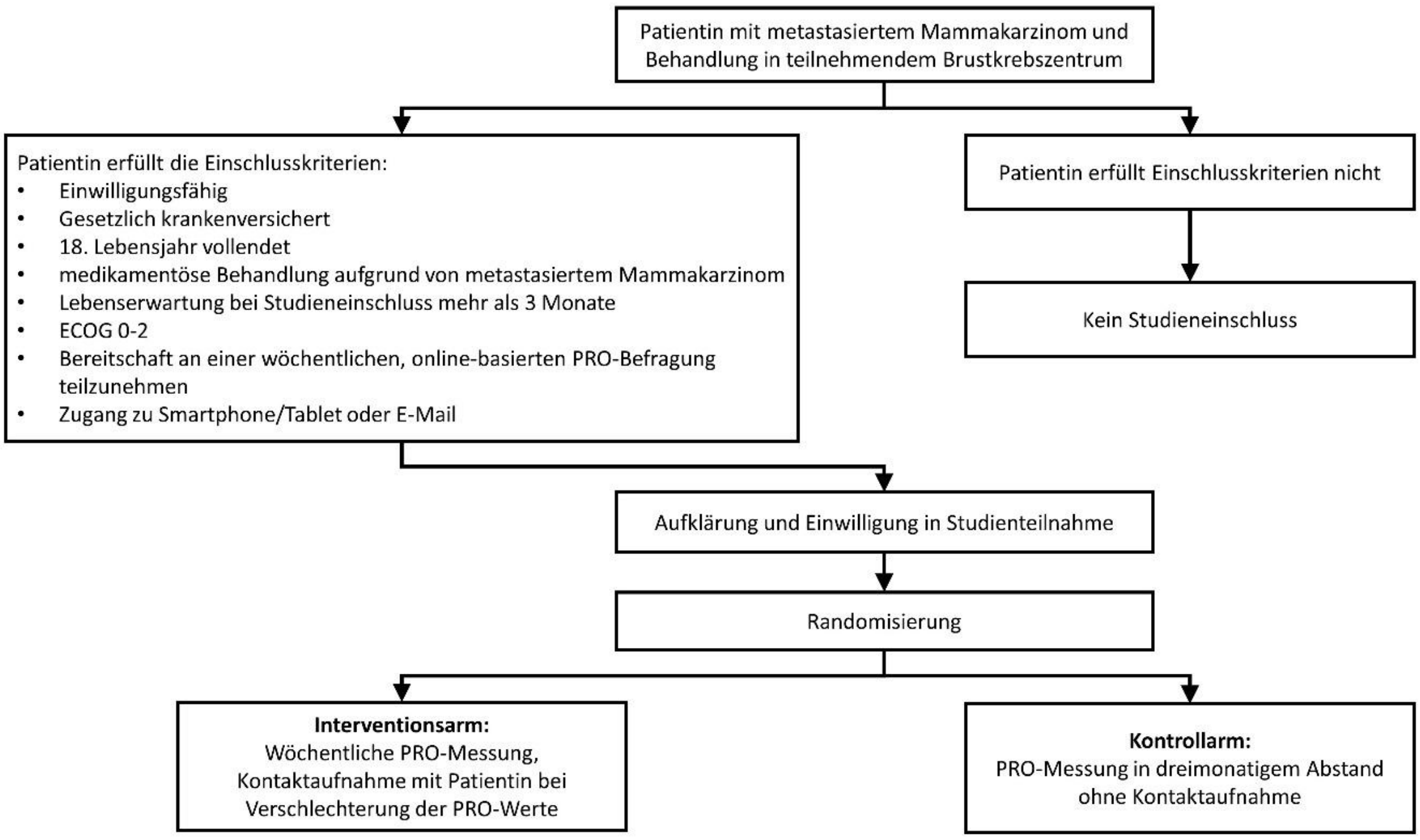

Abbildung 1. Studiendesign

3.2 Zeitplan

Die Gesamtdauer des Forschungsvorhabens beträgt 48 Monate. Die Rekrutierung der Patientinnen ist für Mai 2021 angesetzt und wird ein Jahr andauern.

**Protokoll**	**Version 2.3**	**Version 3.0 (updated)**
**Gesamtdauer**	36 Monate	48 Monate
**Vorbereitungsphase und Studieninitiierung**	Oktober 2020 – April 2021	Oktober 2020 – April 2021
**Rekrutierung der Patientinnen**	Mai 2021 – Mai 2022	Mai 2021 – Juni 2023
**Nachbeobachtung und Monitoring**	Mai 2022 – Mai 2023	Mai 2022 – Februar 2024
**Datenauswertung und Dissemination**	Mai 2023 – September 2023	Februar 2024 – September 2024

4 Rekrutierung

4.1 Fallzahl

Die angestrebte Fallzahl beträgt 500 Patientinnen pro Behandlungsarm. Die Fallzahlkalkulation ist in Kapitel 7.1. dargestellt.

Verringerung des Drop-Outs/ Inzentivierung

Um die Drop-out-Rate in der Kontrollgruppe möglichst gering zu halten, erhalten Patientinnen einerseits nach Abschluss des Beobachtungszeitraums einen Ausdruck oder eine Datei ihrer Befragungsdaten, d. h. ihres PRO-Verlaufs, und andererseits werden Patientinnen bei der dreimonatlichen Befragung ausbleibender Beantwortung persönlich kontaktiert. Die Kontaktaufnahme erfolgt hierbei anders als in der Interventionsgruppe lediglich zur Erinnerung an die Beantwortung der PRO-Befragung. Hierzu werden den teilnehmenden Zentren individualisierte Schreiben für die jeweilige Studienpatientin zur Verfügung gestellt mit der Bitte, diese an die Patientin weiterzuleiten. Alternativ hat das teilnehmende

Zentrum auch die Möglichkeit, die Patientin bei Nichtbeantwortung des Fragebogens telefonisch zu kontaktieren und zu bitten, diesen im Rahmen der Studienteilnahme auszufüllen. Der PRO-Verlauf kann im Nachhinein als Gesprächsgrundlage mit den Behandelnden dienen und etwaige unbemerkte Auffälligkeiten aufdecken. Mit der Kontaktaufnahme durch die Studienzentren wird auch den Patientinnen der Kontrollgruppe vermittelt, dass sie ein wesentlicher Teil der Studie sind und sie nicht weniger Aufmerksamkeit seitens ihrer Behandelnden erfahren.

Dem Feedback der Selbsthilfegruppe Mammakarzinom der Charité folgend, wird das intensivierte digitale Monitoring des PRO-Verlaufs als Inzentivierung der Interventionsgruppe für ausreichend erachtet.

4.2 Studienzentren

Insgesamt werden 52 Brustkrebszentren, verteilt über die Bundesrepublik Deutschland, als Studienzentren rekrutiert. Bei der Rekrutierung der Studienzentren werden die Fallzahlen sowie die Anteile der Patientinnen mit einer Versicherung bei den kooperierenden Krankenkassen BKK·VBU, BARMER, DAK Gesundheit berücksichtigt. Zusätzlich wird bei der Rekrutierung darauf geachtet, dass Studienzentren mit unterschiedlichen Urbanitätsgraden, Trägerschaften sowie Lehrstatus einbezogen werden.

4.3 Einschlusskriterien

Eingeschlossen werden können Frauen

- mit metastasiertem Brustkrebs,
- die in einem an der Studie teilnehmenden Brustkrebszentrum behandelt werden,
- die gesetzlich bei einer der nachfolgenden Krankenkassen versichert sind: BARMER, DAK Gesundheit, BKK·VBU*,
- die einwilligungsfähig sind,
- die ihr 18. Lebensjahr vollendet haben,
- die eine medikamentöse Behandlung aufgrund des metastasierten Mammakarzinoms erhalten,
- die eine Lebenserwartung bei Studieneinschluss von mehr als 3 Monaten haben,
- die als ECOG 0-2 eingestuft werden,
- die bereit sind, an einer wöchentlichen, online-basierten PRO-Befragung teilzunehmen,
- und die Zugang zum Internet (Smartphone) haben.

Die Voraussetzung für die Teilnahme an der Studie ist die informierte Einwilligung der Patientin.

* Eine zeitlich begrenzte Öffnung der Studie für Versicherte anderer Krankenkassen ist nach Zustimmung des Förderers zur Erweiterung der Rechtsgrundlage der Studie möglich.

4.4 Ausschlusskriterien

Nicht an der Studie teilnehmen können Patientinnen, die keine aktive Tumortherapie erhalten (Comfort Care) oder nicht alle der oben genannten Einschlusskriterien erfüllen.

4.5 Abbruchkriterien

Die Studienteilnahme wird abgebrochen, wenn die Patientin die Einwilligung zurückzieht.

5 Intervention

5.1 Beschreibung der Intervention

Die Patientinnen in der neuen Versorgungsform erhalten einmal wöchentlich immer am gleichen Wochentag eine Aufforderung via App (ggf. im Verlauf per E-Mail), die PRO-Befragung durchzuführen. Analog zu den Zielparametern der Studie basiert das Interventionskonzept auf der Operationalisierung der gesundheitsbezogenen Lebensqualität der EORTC Quality of Life Gruppe. Deren Kerninstrument,

der QLQ-C30, umfasst fünf Funktionsdimensionen (Physical Function, Role Function, Emotional Function, Cognitive Function, Social Function) und 9 Symptomskalen (Fatigue, Nausea, Pain, Dyspnoe, Insomnia, Appetite loss, Constipation, Diarrhoea, Financial Difficulties). Die benannten Domänen werden durch studienspezifisch optimierte Fragebögen aus den jeweiligen EORTC Itembanken erhoben. Für die gesundheitsökonomische Auswertung erfolgt zudem die Befragung mittels EQ-5D.

Bei Verschlechterung der PRO-Scores im individuellen longitudinalen Verlauf und/oder im Vergleich zu zuvor definierten Grenzwerten wird ein Alarm generiert, der dem behandelnden Studienzentrum sowie der PRO B-Studienzentrale angezeigt wird. Klinische Relevanz wird anhand publizierter Informationen zum EORTC QLQ-C30 (z.B. MCID) und anhand von Wahrscheinlichkeiten über tatsächliche Veränderung der latenten Variablen berechnet. Um eine Aggregation mehrerer, klinisch irrelevanter Verschlechterungen über mehrere Wochen zu erkennen, soll außerdem ein Vergleich mit einem vorigen Messwert (z. B. vier Wochen zuvor) erfolgen. Ein Alarm muss durch die kooperierenden Studienzentren innerhalb von 48 Stunden (bezogen auf Werktage) quittiert und dokumentiert werden. Das Format der Rückmeldung an die behandelnden Zentren wird mit diesen gemeinsam entwickelt. In jedem Fall beinhaltet es explizit die Domänen und das Ausmaß der Veränderung. Auf den Alarm folgt eine Kontaktaufnahme des jeweiligen Zentrums mit der Patientin, um die Verschlechterung ihrer Werte zu spezifizieren und ggf. zu intervenieren. Die auf die Kontaktaufnahme folgende Intervention bzw. Maßnahme durch das Studienzentrum wird durch PRO B nicht vorgegeben. Die Art der Maßnahmen bleibt somit dem behandelnden Brustkrebszentrum überlassen.

Insofern testet die Studie nicht eine spezifische therapeutische Intervention bei Brustkrebspatientinnen, sondern eine zeitnahe individualisierte Kontaktaufnahme und mögliche Intervention verschiedener Ausprägung bei Verschlechterung subjektiver Lebensqualitätsparameter. Der Effekt der Intervention ergibt sich vor allem durch die zeitnahe individualisierte Kontaktaufnahme des behandelnden Brustkrebszentrums aufgrund alarmierender oder sich alarmierend verschlechternder patientinnenorientierter, lebensqualitätsbezogener Parameter. Diese neue Versorgungsform gilt für alle Patientinnen im Interventionsarm für die gesamte Dauer der Studie (24 Monate), mindestens jedoch für zwölf Monate.

5.2 Risiken der Intervention

Die Intervention besteht aus der Eingabe gesundheitsbezogener Daten in eine App. Gesundheitliche Risiken sind dabei als gering einzuschätzen.

Die Risikofaktoren für das Gelingen der Intervention bestehen in der mangelnden Reaktion/Intervention auf eine Verschlechterung der Befragungswerte seitens des behandelnden Teams. Um eine korrekte und vor allem in zeitlichem Bezug stehende Kontaktaufnahme mit der Patientin sicherzustellen, wird in das Befragungsmodul ein Alarmsystem integriert, das anhand eines hinterlegten Algorithmus bei Verschlechterung der Werte einen Alarm an das behandelnde Brustkrebszentrum und die Studienzentrale meldet. Wird der Alarm nicht innerhalb von 48 Stunden (bezogen auf Werktage) durch das Brustkrebszentrum quittiert und die dadurch ausgelöste Intervention dokumentiert, erfolgt eine telefonische Kontaktaufnahme mit dem Brustkrebszentrum durch die Studienzentrale.

5.3 Randomisation

Die Randomisation erfolgt nach folgenden Strata:

- Zentren
- Arten der Fernmetastasierung (3 Gruppen: ossäre Metastasen & Hautmetastasen & Lymphknotenmetastasen; viszerale Metastasen (ein Organ); Gehirnmetastasen & multiple Metastasen)
- histologischer Befund (2 Gruppen: Luminal (HR+/HER-) & triple positiv (HR+/HER+); HR-/HER+ & Triple negativ)

Durch die hohe Anzahl der Strata kann es zu Unbalanciertheiten bei einer einfachen Randomisation hinsichtlich der Stratifizierungsvariablen zwischen der Interventions- und der Kontrollgruppe kommen.

Um diese möglichst gering zu halten, wird unter Verwendung der Minimierungsmethode von Pocock und Simon adaptiv randomisiert (17). Die Randomisierung erfolgt nach Aufklärung und Einwilligung, Download der Applikation und Baseline-Befragung (inkl. soziodemografische Angaben und Komorbiditäten, siehe Kapitel 5.5.).

5.4 Verblindung

Es findet keine Verblindung statt.

5.5 Mögliche Confounder

Als mögliche Confounder werden einmalig im Rahmen der Erstbefragung Daten zu folgenden sozioökonomischen und soziodemographischen Parametern erhoben:

- Familienstand
- Pflegebedürftige Angehörige/ minderjährige Kinder (≤14 Jahren) im Haushalt
- Bildungsabschluss/ Berufsabschluss
- Erwerbsstatus
- Einkommen
- Migrationshintergrund

Neben der Brustkrebs-spezifischen Anamnese (familiäre Krebsbelastung) ermöglicht die Befragung über die Smartphone-Applikation auch die Erhebung eventueller Vorerkrankungen. Wir vermuten einen nicht unerheblichen Einfluss folgender Komorbiditäten auf den primären Endpunkt der Studie (Fatigue). Sie müssen daher sowohl in der Interpretation der Messwerte im laufenden Projekt als auch in der abschließenden Auswertung (z. B. als NCI Comorbidity Index (unterstrichene Erkrankungen)) berücksichtigt werden:

- kardiale und kardiovaskuläre Vorerkrankungen (arterielle Hypertonie, koronare Herzerkrankung, Angina pectoris, <u>Myokardinfarkt</u>, <u>Herzinsuffizienz</u>, Vorhofflimmern, Klappenvitien)
- vaskuläre Vorerkrankungen (tiefe Venenthrombose, <u>periphere arterielle Verschlusskrankheit</u>, Lungenarterienembolie)
- cerebrovaskuläre Erkrankungen (<u>TIA</u>, <u>Apoplex</u>)
- hämatologische Vorerkrankungen (Anämie)
- pneumologische Vorerkrankungen (<u>Asthma bronchiale</u>, <u>COPD</u>, Schlafapnoe)
- endokrine/ metabolische Vorerkrankungen (Hyperlipidämie, <u>Diabetes mellitus (therapiebedürftig ohne und mit Endorganschäden)</u>, Hypothyreose, Adipositas)
- rheumatologische Vorerkrankungen (<u>Polymyalgia rheumatica</u>, <u>Lupus erythematodes,</u> <u>rheumatoide Arthritis</u>, <u>Polymyositis</u>)
- psychische Vorerkrankungen (Depression, Angststörung)
- neurologische Vorerkrankungen (Migräne, Krampfanfälle, Gleichgewichtsstörungen, Neuropathie, <u>Hemiplegie</u>, Paraplegie, <u>Demenz</u>)
- gastrointestinale Vorerkrankungen (<u>gastrointestinale Ulcera</u>, chronisch entzündliche Darmerkrankung, Divertikulitis, Reflux, Reizdarmsyndrom, <u>leichte Lebererkrankung</u> (Leberzirrhose ohne portale Hypertonie), <u>schwere Lebererkrankung</u> (Leberzirrhose mit portaler Hypertonie, Ösophagusvarizenblutung))
- gynäkologische Vorerkrankungen (Infertilität, Abort, , Endometriose,)
- urogenitale Vorerkrankungen (Nephro- bzw. Urolithiasis, <u>Niereninsuffizienz</u>, <u>Dialysepflicht</u>, Miktionsbeschwerden, Harnwegsinfekte, Inkontinenz)
- Infektionserkrankungen (HIV, <u>AIDS</u>)
- onkologische Vorerkrankungen (andere Tumorerkrankungen (mit und ohne Metastasen), Leukämie, Lymphom)
- Voroperationen
- Suchtmittelanamnese (Alkohol, Nikotin)

6 Studienablauf

6.1 Beschreibung des Studienablaufs

Die Einschlusskriterien erfüllende Patientinnen im Brustkrebszentrum werden von den Prüfärzt*innen über die Studie informiert. Die informierte Einwilligung vorausgesetzt, erfolgt die Aufnahme in die Studienpopulation. Anschließend wird die App für die PRO-Befragungen auf dem Smartphone/Tablet der Studienteilnehmerin installiert, über die die Befragung durchgeführt werden kann. Die Teilnahme per E-Mail wird nur im Falle einer schleppenden Rekrutierung implementiert. Zunächst ist nur eine Teilnahme per App möglich. Soweit erforderlich, regelt ein Amendment des Studienprotokolls die Teilnahme per E-Mail. Dann erfolgt die Randomisierung der Studienteilnehmerin in einen Studienarm.

Im Interventionsarm wird die Patientin anschließend für mindestens 12 Monate und maximal 24 Monate wöchentlich durch die App (oder per E-Mail) aufgefordert, an der PRO-Befragung teilzunehmen. Bei einer Verschlechterung der durch die Patientin rückgemeldeten Werte wird durch eine Web-Anwendung ein Alarm an das Brustkrebszentrum gesendet, welches dann mit der Patientin Kontakt aufnimmt und mögliche Maßnahmen abstimmt. Alarme werden vom Brustkrebszentrum in der Web-Anwendung quittiert und Kontaktaufnahmen sowie ggf. Maßnahmen dokumentiert. Die PRO-B Studienzentrale hat ebenfalls Zugriff zu der Web-Anwendung und kann Alarme sowie die Reaktion der Brustkrebszentren überprüfen. Sollte ein Brustkrebszentrum innerhalb von 48 Stunden einen Alarm nicht quittiert haben, nimmt die Studienzentrale Kontakt zu dem Brustkrebszentrum auf.

Eine Patientin im Kontrollarm wird alle 3 Monate durch die App oder per Mail aufgefordert, an der PRO-Befragung teilzunehmen. Die Antworten werden ebenfalls unmittelbar elektronisch an die Web-Anwendung übermittelt. Für die Kontrollgruppe erfolgt keine Alarmierung bei Verschlechterung der PRO-Werte und damit auch keine Kontaktaufnahme. Bei Nicht-Beantwortung der Befragung erfolgt eine schriftliche oder telefonische Erinnerung durch das Studienzentrum.

Nach Einwilligung durch die Patientin erfolgt während der Behandlung die Dokumentation klinischer Daten in einem Electronic Case Report Form (eCRF) (Tumor- und Studiendokumentationssoftware PRO B Doc der Firma OnkoZert). Darüber hinaus erfolgt die Erhebung der Routinedaten durch die Krankenkasse der Patientin. Abbildung 2 gibt einen Überblick über den individuellen Studienablauf der Patientinnen.

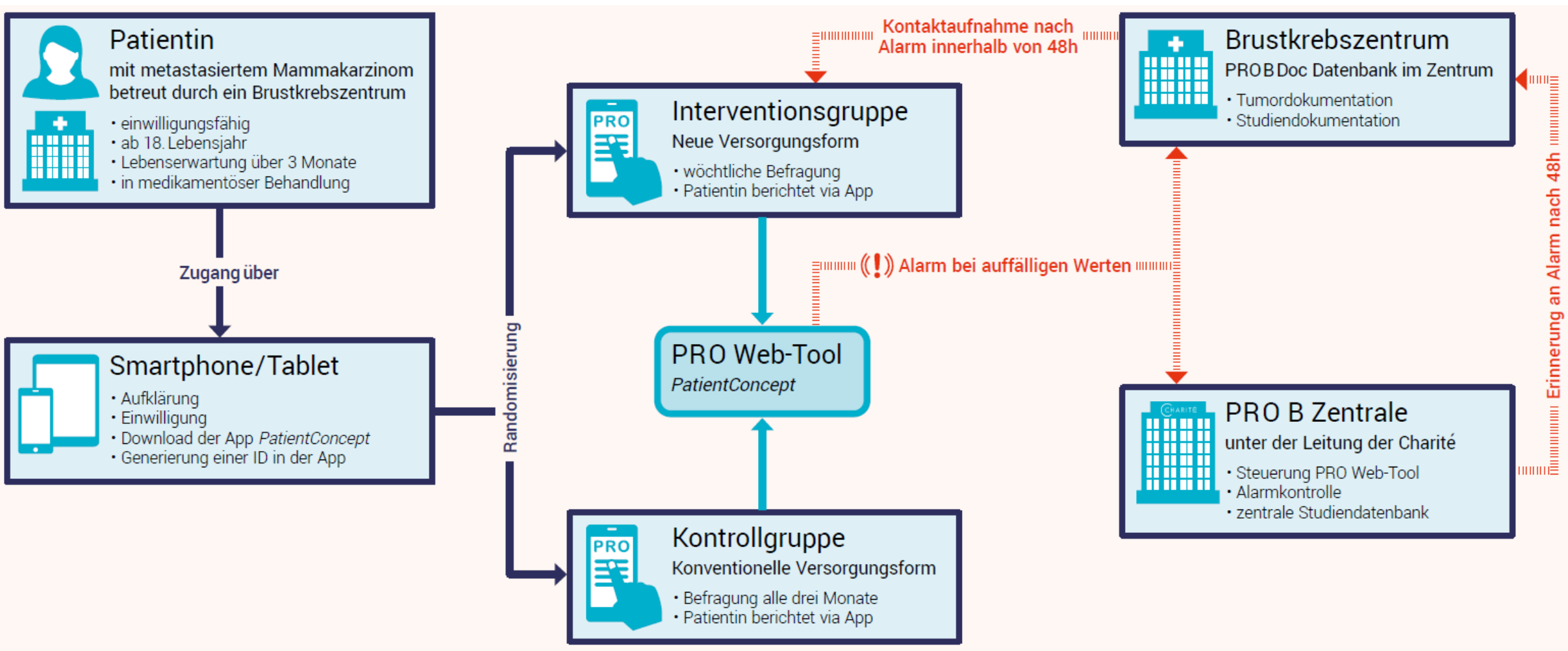

Abbildung 2. Individueller Studienablauf

6.2 Geplante Behandlung nach Studienende

Im Falle positiver Ergebnisse kann die neu geschaffene Versorgungsform aufgrund ihrer besonderen Struktur mit überschaubarem Aufwand in die Regelversorgung übernommen werden. Die Patientinnen erhielten in diesem Zusammenhang nach der Studie, wie auch während des Studienbetriebs, keine von

der üblichen klinischen Routine abweichende spezifische Behandlung, würden jedoch bezüglich ihrer Symptome und Lebensqualität weiterhin elektronisch monitoriert. Die Versorgung, die während PRO B evaluiert wird, könnte demnach in sehr ähnlicher Form nach Studienende weitergeführt und für andere Patient*innengruppen ausgeweitet werden. Hierüber wäre zum Beispiel auch die Implementierung und Evaluation einer PRO-gestützten Behandlung und Nachsorge von Brustkrebspatient*innen in der adjuvanten Situation möglich.

7 Statistische Vorgehensweise

7.1 Fallzahlkalkulation

Cocks et al. erstellten Guidelines für die Interpretation längsschnittlicher Veränderungen auf der Fatigue-Skala des EORTC QLQ-C30, nach denen Werte ab durchschnittlich 4 Punkten Verbesserung als kleine Verbesserung und Werte ab 9 Punkten als mittlere Verbesserung einzuschätzen sind (18). In PRO B geht es jedoch um einen Vergleich zwischen Behandlungs- und Kontrollgruppe. Die Effektschätzungen folgen den Angaben von Cocks et al., allerdings sind aufgrund des Zwei-Gruppen-Vergleichs sehr viel größere Streuungsmaße zu erwarten weshalb konservativ eine zu erreichende standardisierte Effektstärke von etwa 0,2 geschätzt wird (mittlere Differenz zum Beispiel 5 Punkte. Gemeinsame Standardabweichung 25,2 Punkte). Eine solche Streuung ist vergleichbar mit den Angaben von Cortes et al. 2015 (19).

Da außerdem die Analyse bestimmter Subgruppen, zum Beispiel Patientinnen mit viszeralen Metastasen, besonders relevant ist, wird eine Fallzahl angestrebt, die auch für Subgruppenanalysen ausreichend groß ist. Die Rekrutierung von 500 Patientinnen pro Gruppe wird als realistisch angesehen. Bei einer erwarteten Drop-Out-Rate von 20% resultiert dies in 400 vollständigen Datensätzen pro Behandlungsarm. Wenn ein minimaler klinisch relevanter Effekt von 5 Punkten Mittelwertunterschied auf der Fatigue-Skala des EORTC QLQ-C30 vorliegt, wie von Cocks et al. (18) berichtet (bei einer gemeinsamen Standardabweichung von 25), hat ein zweiseitiger t-Test mit einem Signifikanzniveau von 5% eine Power von 80,65%, einen Unterschied im Fatigue Score zwischen Interventions- und Behandlungsgruppe mit 400 Patientinnen pro Gruppe nachzuweisen. Diese Berechnung wurde mit nQuery (Version 8.3.1.0) durchgeführt. Die Fallzahl ist somit auch für die Detektion kleiner Effektstärken (0,2 oder größer) ausreichend und trägt der Anzahl relevanter Subgruppen in der Patientinnenpopulation Rechnung.

Da die geplante Stichprobengröße nicht im ursprünglich intendierten Rekrutierungszeitraum erreicht werden kann, wird der Rekrutierungszeitraum um vier Monate verlängert. Die insgesamt erwartete Stichprobengröße beträgt 400 Patientinnen pro Gruppe. Bei einer erwarteten Drop-out-Rate von 20 % ergibt dies etwa 320 vollständige Datensätze pro Behandlungsgruppe. Diese Fallzahl ist somit für den Nachweis von Effektgrößen von 0,2 mittels zweiseitigem t-Test für unabhängige Stichproben mit einem Signifikanzniveau von 5% und einer Power von 71,4% ausreichend. Wenn die Effektgröße 0,225 oder größer ist, kann eine Power von mindestens 81,1 % erreicht werden.

7.2 Variablen

In der untenstehenden Tabelle werden der primäre Outcome sowie die sekundären Outcomes der Studie aufgeführt. Weitere Variablen, die im Rahmen der der Randomisierung oder als mögliche Confounder relevant sind, werden für die Baseline-Befragung und das Studiendokumentationssystem (eCRF in PRO B Doc) definiert. Die Erhebung der Daten in PRO B Doc erfolgt alle drei Monate während der regulären klinischen Vorstellung im behandelnden Brustkrebszentrum.

Tabelle 2. Übersicht primärer Outcome und sekundäre Outcomes

Variable	Datenquelle	Erhebungszeitpunkt			
		Baseline	Wöchentlich in Interventionsgruppe; alle 3 Monate in Kontrollgruppe	6 Monate	12 Monate
**Primärer Outcome**					
Fatigue Score (EORTC)	Studienspezifischer Fragebogen aus EORTC Itembank	x	x	x	x
**Sekundäre Outcomes**					
Physical functioning score (EORTC)	Studienspezifischer Fragebogen aus EORTC Itembank	x	x	x	x
Health-related Quality of Life Summenscore (EORTC)	Studienspezifischer Fragebogen aus EORTC Itembank	x	x	x	x
Anzahl der Krankenhaus- und Rettungsstellenaufenthalte	PRO B Doc/ Krankenkassen-daten				x
Gesamtüberleben bei viszeraler Metastasierung	PRO B Doc, Krankenkassen-daten				x
Gesamtüberleben bei triple negativem Mammakarzinom	PRO B Doc, Krankenkassen-daten				x
Zeit bis zum ersten Wechsel der systemischen Therapie	PRO B Doc				x

7.3 Pretest

Die Probebefragung (Pretest) wird die für die Verlaufsbeobachtung studienspezifischen Messinstrumente beinhalten. Dazu werden teilweise Think-Aloud-Techniken angewandt, teilweise wird den Befragungen ein kurzer Evaluationsfragebogen angehängt, der eine Auswertung zu Befragungsinhalten und Belastungen durch die Beantwortung ermöglicht. Respondentinnen für die Probebefragung werden im Brustzentrum der Charité – Universitätsmedizin Berlin rekrutiert.

7.4 Statistische Methoden

Statistische Analyse der Hypothesen

Die Auswertung der Daten erfolgt durch das Institut für Biometrie und Klinische Epidemiologie (iBiKE) der Charité Universitätsmedizin Berlin.

Analyse der primären Hypothese

Die primäre Forschungsfrage wird in einer Intention-to-treat-Analyse für alle randomisierten Fälle untersucht, die mindestens eine PRO-Erhebung beantwortet haben. Patientinne, die nie auf die PRO-Erhebung antworten, werden nicht als vollständiger Analysesatz betrachtet (modified ITT-Population). Alle Stratifizierungsvariablen der Randomisation werden als Kovariaten in allen statistischen Modellen berücksichtigt.

Für die Berechnung der Fallzahl wurde ein zweiseitiger t-Test verwendet, obwohl in der Auswertung ein ANCOVA-Modell verwendet werden soll. Es wurde gezeigt, dass dies eine konservative Vorgehensweise für die Schätzung der Fallzahl von ANCOVA-Modellen ist, da ein ANCOVA-Modell mit (1 - 2ρ)*n Beobachtungen mindestens die gleiche Power wie ein t-Test mit n Beobachtungen hat (20), wobei ρ der Varianzinflationsfaktor (Korrelation zwischen der *Baseline*-Messung und der *Follow-up*-Messung) ist. Im schlechtesten Fall von ρ = 0 führt dies zu einer benötigten Fallzahl gleich dem t-Test. Die Analyse erfolgt im ANCOVA-Modell (gemischte lineare Regressionsmodelle) mit der Baseline-Messung sowie Arten der Metastasierung und Histologie als Kovariaten (analog der Stratifizierung der Randomisation). Außerdem wird die Analyse adjustiert für das Alter, Art und Anzahl der Chemotherapien, dem ECOG-Status bei Studieneinschluss, der Computer-Erfahrung und gegebenenfalls für weitere wichtige C*onfounder*. Die Heterogenität zwischen den Studienzentren wird durch *random effect* berücksichtigt (gemischtes lineares Regressions-Modell, *random intercept model*).

Analysen der Sekundären Hypothesen

Alle sekundären Hypothesen werden analog zur primären Hypothese und entsprechend des jeweiligen Outcomes mittels geeigneter statistischer Methoden (z. B. Zeit-Ereignis-Modelle, ANCOVA, logistische Regression, Cox Regression) analysiert. Wie bei der Analyse des primären Outcomes wird auch bei den sekundären Hypothesen für relevante Confounder adjustiert und die Heterogenität zwischen den Studienzentren statistisch angemessen berücksichtigt. Weitere Outcomes und insbesondere verschiedene Subgruppen können im Laufe der Studie noch zusätzlich in die Analyse aufgenommen werden. In diesem Fall werden sie im statistischen Analyseplan (SAP) aufgeführt und explorativ ausgewertet.

Umgang mit fehlenden Werten

Fehlende Werte werden unter der Annahme, dass sie „*missing at random*“ (MAR) sind, mittels multipler Imputationsmethoden imputiert. In jedem Fall werden die Gründe für das Fehlen der Werte so gut wie möglich dokumentiert und berichtet. Aufgrund der längeren Rekrutierungszeit von vier Monaten werden die spät rekrutierten Patientinnen nur 6 Monate lang vollständig beobachtet. Für diese Patientinnen werden keine sekundären Endpunkte nach 12 Monaten mittels Imputationsmethoden geschätzt.

Statistische Analysen der Subgruppen

Für die statistischen Analysen der Subgruppen wird zunächst im gemischte lineare Regressionsmodelle (Outcome: primäres oder sekundäres Outcome, Kovariablen: jeweilige Baseline Variable, Treatment-Variable, Stratifizierungsvariablen der Randomisation, random Intercept für die Zentren) zusätzlich ein Interaktionsterm der Treatment-Variable mit der Subgruppe eingeschlossen, um erste Hinweise für differentielle Treatment-Effekte in den Subgruppen zu analysieren. Zusätzlich werden aus den Modellen marginale Effektschätzer für jede Subgruppe und 95%-Konfidenzintervalle angegeben. Alle Subgruppenanalysen sind explorativ.

Definition von Subgruppen und Begründung der Bedeutung von Subgruppenanalysen

- **Metastasierung** (sekundär/therapiert, primär/Therapienaivität)
- **Tumorsubtyp** (HR+/HER2+, HR+/HER2–, HR–/HER2+, TNBC)
- **Ort der Metastasierung** (cerebral, viszeral, ossär, multipel)
- **Alter bei Randomisierung** (<50, >50)
- **Therapie bei Studieneinschluss** (Chemotherapie, endokrine Therapie, Anti-Her2-Therapie, Immuntherapie, zielgerichtete Therapie und andere)
- **Beziehungsstatus** (Partnerschaft/Ehe, ledig)
- **Einkommen** (Mediansplit)
- **Depression** (ja/ehemals, nein)
- **BMI** (<30, ≥30 kg/m^2)
- **Veränderung der Alarmkriterien** (vor und nach dem 01.09.2022)

Patientinnen mit **primär metastasiertem Brustkrebs** haben eine bessere Prognose als Patientinnen mit rezidivierter metastasierter Erkrankung. Dieser Unterschied wird darauf zurückgeführt, dass etwaige adjuvante Systemtherapien der letzteren ggf. zu Resistenzen führen, die bei therapienaiven Patientinnen, also primär Metastasierten nicht vorliegen (21, 22) . Innerhalb der Gruppe der **sekundär metastasierten Tumoren** gilt ein längeres disease free interval (DFI) als prognostisch günstig (21). Einen weiteren Prognose-entscheidenden Faktor stellt das **Alter** dar. So ist ein jüngeres Alter bei initialem Auftreten von Fernmetastasen mit einer besseren Prognose assoziiert (21). Grund für die schlechtere Überlebensrate der Patientinnen über 50 könnte in diesem Zusammenhang eine geringere Ansprechrate auf Chemotherapien mit ggf. mehr Nebenwirkungen und häufigerer Dosisreduktion sein (22). Weiterhin gilt die **Tumorbiologie** als wesentlicher prognostischer Faktor, da von ihr unter anderem auch das Streuungsmuster der neoplastischen Zellen abhängt. Die beste Prognose wird für Hormonrezeptor (HR) positive (Luminal A), gefolgt von HER2neu (HER) exprimierenden und die schlechteste für Luminal B und triple-negative Tumoren (TNBC) beschrieben (23). Diese Unterschiede werden unter anderem auf unterschiedliche Metastasentropismen zurückgeführt. Bei den Luminaltumoren (HR+/HER-) liegen vordergründig ossäre Metastasen vor, TNBC dagegen weisen weitaus häufiger viszerale und cerebrale Metastasen auf (24), wobei mit letzteren mithin das schlechteste Overall Survival (OS) assoziiert ist (23). Unabhängig von der Hormonrezeptorexpression liegt bei HER2neu positiven Tumoren am häufigsten eine viszerale Metastasierung vor, die ebenfalls mit einem kürzeren Gesamtüberleben als Knochenmetastasen einhergeht (23). HER2neu exprimierende Tumoren stellen zudem einen Risikofaktor für cerebrale Metastasen dar, da die zur Verfügung stehende HER2neu gerichtete Therapie (z. B. Trastuzumab) nicht liquorgängig ist (25).

Einen weiteren mitunter auch prognostischen Faktor stellt die für die PRO B Studie als primärer Endpunkt definierte Fatigue dar. Diese wird durch unterschiedliche Parameter beeinflusst. So zeigen sich z. B. **Therapie-abhängige Unterschiede** des Auftretens und Verlaufs Fatigue-spezifischer Symptome zwischen Patientinnen nach Chemotherapie und endokriner Therapie (26). Auch **soziodemographische Charakteristika** wie das Einkommen und der Beziehungsstatus der Betroffenen sind mit dem Auftreten Tumor-bedingter Fatigue assoziiert (27). In mehreren Studien wurde außerdem festgestellt, dass ein Zusammenhang von Fatigue mit **Depression** und **mangelnder körperlicher Aktivität/erhöhtem Body-Maß-Index** besteht (28, 29).

Gesundheitsökonomische Auswertung

Über den Studienzeitraum werden die Gesamtkosten der Behandlung über die Krankenkassendaten für alle teilnehmenden Frauen gesammelt und ausgewertet. Dabei wird die jeweilige Beobachtungszeit ebenfalls berücksichtigt (mittlere Behandlungskosten pro Jahr pro Patientin).

Um die schiefe Verteilung der Kostendaten zu berücksichtigen, führen wir eine Log-Transformation und ein lineares gemischtes Modell durch, bei dem das Studienzentrum als zufälliger Intercept verwendet wird. Weitere statistische Methoden der gesundheitsökonomischen Analyse werden im statistischen Analyseplan zu Beginn der Projektlaufzeit ausgeführt. Kosteneffektivitätsanalysen werden dann durchgeführt, wenn sich die Intervention als überlegen in Bezug auf „quality adjusted life years (QALYs)" erweist. Ein QALY berücksichtigt sowohl Lebenszeit als auch Lebensqualität von Interventionen. QALYs werden auf der Grundlage von Lebensqualitätsfragebögen (EQ-5D) berechnet, die zu Baseline und zu verschiedenen folgenden Zeitpunkten während der Projektlaufzeit ausgefüllt werden. Zwischen den Messzeitpunkten wird eine lineare zeitliche Veränderung der Lebensqualität angenommen. QALYs ergeben sich aus der Berechnung der Fläche unter der Kurve. Falls die Intervention zu besseren Lebensqualitätswerten bei gleichzeitig geringeren Kosten führt, gilt sie als kosteneffektiv. Falls es zu zusätzlichen Kosten in der Interventionsgruppe kommt, wird der „incremental cost-effctiveness ratio (ICER)" berechnet, indem die mittleren zusätzlichen Kosten für ein zusätzliches QALY geschätzt werden. Eine Grenze von 50.000 € pro extra QALY wird in Studien oft als Grenzwert für die Kosteneffektivität einer Intervention verwendet. Ist der ICER unter dieser Grenze, gilt die Intervention als kosteneffektiv.

8 Datenmanagement

8.1 Datenerhebung

Die Erhebung der patientinnenseitig berichteten Daten erfolgt mittels eines durch die Firma NeuroSys entwickelten Web-Tools (PatientConcept), auf das die teilnehmenden Patientinnen entweder via App oder im Verlauf ggf. über einen E-Mail-Link zugreifen können. Bei der initialen Anmeldung bei dem Web-Tool wird eine Identifikationsnummer generiert (Patient Concept ID, 6-stellig bestehend aus Zahlen sowie Groß- und Kleinbuchstaben), durch die die Befragungsdaten pseudonymisiert erhoben werden können und eine Verknüpfung mit den weiteren erhobenen Daten ermöglicht wird. Sowohl die Studienzentrale als auch das Studienzentrum haben ebenfalls Zugriff auf das Web-Tool: Das PatientCocept-Portal benachrichtigt das Studienzentrum und die Studienzentrale bei auffälligen Werten (Alarm), woraufhin das Studienzentrum Alarme quittieren sowie ggf. durchgeführte Maßnahmen dokumentieren kann.

Das Studienzentrum erhebt klinische, zertifizierungsrelevante, prozessbezogene und studienrelevante Patientinnenangaben über das Tumor- und Studiendokumentationssystem PRO B Doc. Die Eingaben im PatientConcept Portal (Befragungsdaten, Alarmquittierung, eventuelle Maßnahmen) werden zu definierten Zeitpunkten (in jedem Fall nach 6, 12 und nach 24 Monaten) vom Studienzentrum, bzw. von der Studienzentrale jeweils als XML- oder JSON Datei heruntergeladen. Danach wird die Summe aller Projektdaten (klin. Tumordaten, Studiendaten und Befragungsdaten) von jedem einzelnen Zentrum über die Funktionalitäten der OncoBoxqualitätsgeprüft. Die Datensätze aller Zentren werden dann durch OnkoZert mittels der OncoBox Research zusammengeführt. OnkoZert führt eine weitere Qualitätssicherung der Daten durch (Vollständigkeit etc.) und leitet sie über eine sichere Umgebung (z. B. Citrix-Umgebung) an die PRO B-Zentrale zur Auswertung weiter.

Zusätzlich werden durch die entsprechenden Krankenkassen der Patientinnen Routinedaten zur Verfügung gestellt und über eine Datenintegrationsstelle (siehe Kapitel 8.3) in der zentralen Studiendatenbank mit den anderen Daten verknüpft. Die Abbildungen 3 und 4 fassen den Datenfluss bei PRO B zusammen.

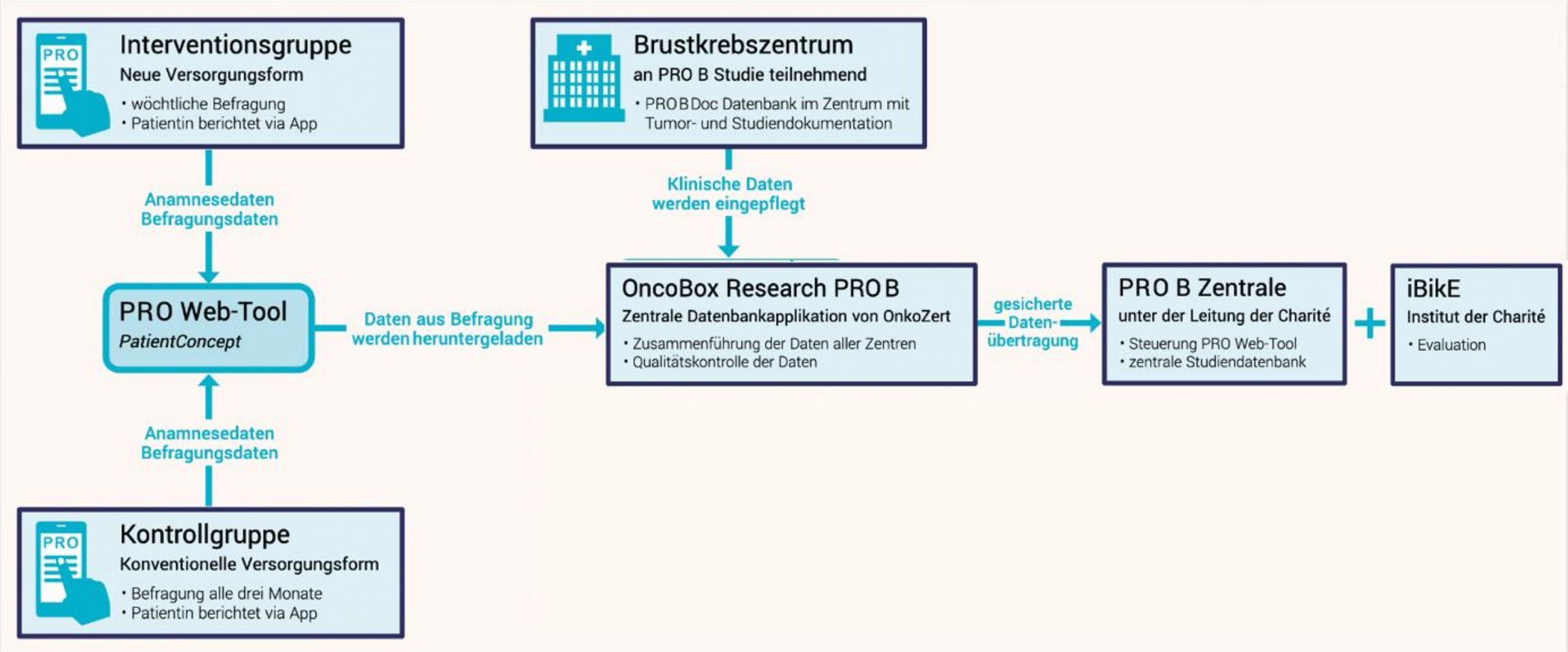

Abbildung 3. Datenfluss. *PRO* Patient-Reported Outcome, *iBiKE* Institut für Biometrie und Klinische Epidemiologie

8.2 Datenverarbeitung, Qualitätskontrolle, Monitoring

Wenn Daten während der Erhebung nicht verfügbar oder nicht zutreffend sind, wird dies entsprechend vermerkt. In Bezug auf Medikamente und Nebenwirkungen wird die Terminologie der internationalen Referenzlisten (WHO dictionary, MeDRA) beachtet.

Die Studiendokumentation wird durch das Charité-BIH Clinical Study Center monitoriert. Dazu führt das CSC pro Studienzentrum ein einmaliges in-person Monitoring durch, bei dem ein stichprobenartiger Quelldatenabgleich samt Überprüfung auf Vollständigkeit, Konsistenz und Plausibilität erfolgt. Sollten Mängel bei der Studiendokumentation festgestellt werden, wird das Studienzentrum zur Behebung dieser aufgefordert. In einem zweiten in-person Monitoring wird dann die Behebung der Dokumentationsmängel überprüft.

Vor dem Export der Datensätze überprüft PRO B Doc mithilfe der Funktionalitäten der OncoBox die Daten auf Plausibilität und Vollständigkeit (Qualitätssicherung). Zusätzlich führt OnkoZert analog der Kriterien der OncoBox eine Qualitätskontrolle der Datensätze durch.

Die Auswertung der Daten erfolgt durch das Institut für Biometrie und Klinische Epidemiologie (iBiKE) der Charité Universitätsmedizin Berlin.

8.3 Datenspeicherung und Datenschutz

Für die Studie liegt ein Datenschutzkonzept vor, welches durch die Kanzlei Beiten Burkhardt geprüft wurde.

Im Rahmen der PRO B-Studie werden patientinnenseitig berichtete Befragungsdaten sowie durch die Studienzentren dokumentierte klinische Daten erhoben und anschließend mit Routinedaten der Krankenkassen verknüpft.

Die Erhebung der Befragungsdaten erfolgt in pseudonymisierter Form über ein Web-Tool (PatientConcept-Portal), das durch die Firma NeuroSys programmiert wird. Die identifizierenden Daten, die zu einer Patient-ID gehören, liegen ausschließlich dem behandelnden Studienzentrum vor und werden nicht an die zentrale Studiendatenbank übermittelt. Die Datenverarbeitung und -speicherung im Rahmen des PatientConcept Portals erfolgt nach dem Datenschutzkonzept. Die beteiligten Zentren tragen dafür Sorge, dass nur Befugte personenbezogene Daten zur Kenntnis nehmen können, z. B. durch Zutrittskontrolle durch technische Maßnahmen in gesicherten Räumen, Benutzer*innenkontrolle durch Passwortregelung zur Legitimation und durch automatische Bildschirmsperrung sowie durch Zugriffskontrolle durch Vergabe unterschiedlicher Berechtigungen und differenzierter Zugriffsmöglichkeiten auf einzelne Felder (Vertraulichkeit). Sie gewährleisten zudem, dass personenbezogene Daten während der Verarbeitung unversehrt, vollständig und aktuell bleiben, z. B. indem sie unbefugte oder zufällige Datenverarbeitung durch Sperre des Zugriffs auf Betriebssysteme und/oder Verschlüsselung der Daten vermeiden und durch regelmäßige Kontrolle der Aktualität der Daten prüfen (Integrität); außerdem, dass personenbezogene Daten zeitgerecht zur Verfügung stehen und ordnungsgemäß für die Pseudonymisierung und nachfolgende Übermittlung verarbeitet werden können, z.B. durch klare und übersichtliche Ordnung des Datenbestandes und Vergabe von Zugriffsbefugnissen im erforderlichen Umfang (unter Abwägung gegenüber dem Gebot der Vertraulichkeit) (Verfügbarkeit). Des Weiteren stellen sie sicher, dass jederzeit personenbezogene Daten ihrem Ursprung zugeordnet werden können, z. B. durch Dokumentation der Ursprungsdaten und ihrer Herkunft und Nachvollziehbarkeit der Verarbeitungsschritte (Authentizität). Außerdem sorgen sie dafür, dass festgestellt werden kann, wer wann welche personenbezogenen Daten in welcher Weise verarbeitet hat, z. B. durch Festlegung klarer Zuständigkeiten und Verantwortlichkeiten sowie die Protokollierung der Eingabe und weiteren Verarbeitung der Daten (Revisionsfähigkeit). Die Verfahrensweisen bei der Verarbeitung personenbezogener Daten werden vollständig, aktuell und in einer Weise dokumentiert, dass sie in zumutbarer Zeit nachvollzogen werden können.

Die Routinedaten der Krankenkassen werden am Ende des Projekts in pseudonymisierter Form in die zentrale Studiendatenbank übermittelt (siehe Abbildung 4). Die Datenübermittlung erfolgt über eine Datentreuhandstelle (CHA-THS) sowie ein Datenintegrationszentrum: (1) Die Studienzentren übermitteln die PatientConcept-ID mit der Versichertennummer an die CHA-THS. (2) Die CHA-THS teilt jeder PatientConcept-ID ein neues Krankenkassen(KK)-Pseudonym zu und übermittelt dieses gemeinsam mit der Versichertennummer an die Krankenkassen. (3) In Verbindung mit dem KK-Pseudonym übermitteln die Krankenkassen die Sekundärdaten an das Datenintegrationszentrum. (4)

Das Datenintegrationszentrum erhält von der CHA-THS die PatientConcept-ID, das KK-Pseudonym und eine neue ID für die Evaluation (Evaluations-ID). (5) Im Anschluss erfolgt die Verknüpfung der KK-Daten mit den anderen Studiendaten aus der PRO B Zentrale. (6) Abschließend wird der vollständige Datensatz mit Evaluations-ID nach Aufbereitung durch die CRO an das evaluierende Institut (iBiKE) weitergeleitet.

Die Übermittlungen der verschiedenen Daten in die zentrale Studiendatenbank (in der Charité ansässig) erfolgen über sichere Umgebungen. Die Daten werden gemäß den geltenden Datenschutzbestimmungen und Datenschutzkonzepten der Charité verarbeitet und gespeichert (siehe Datenschutzkonzept).

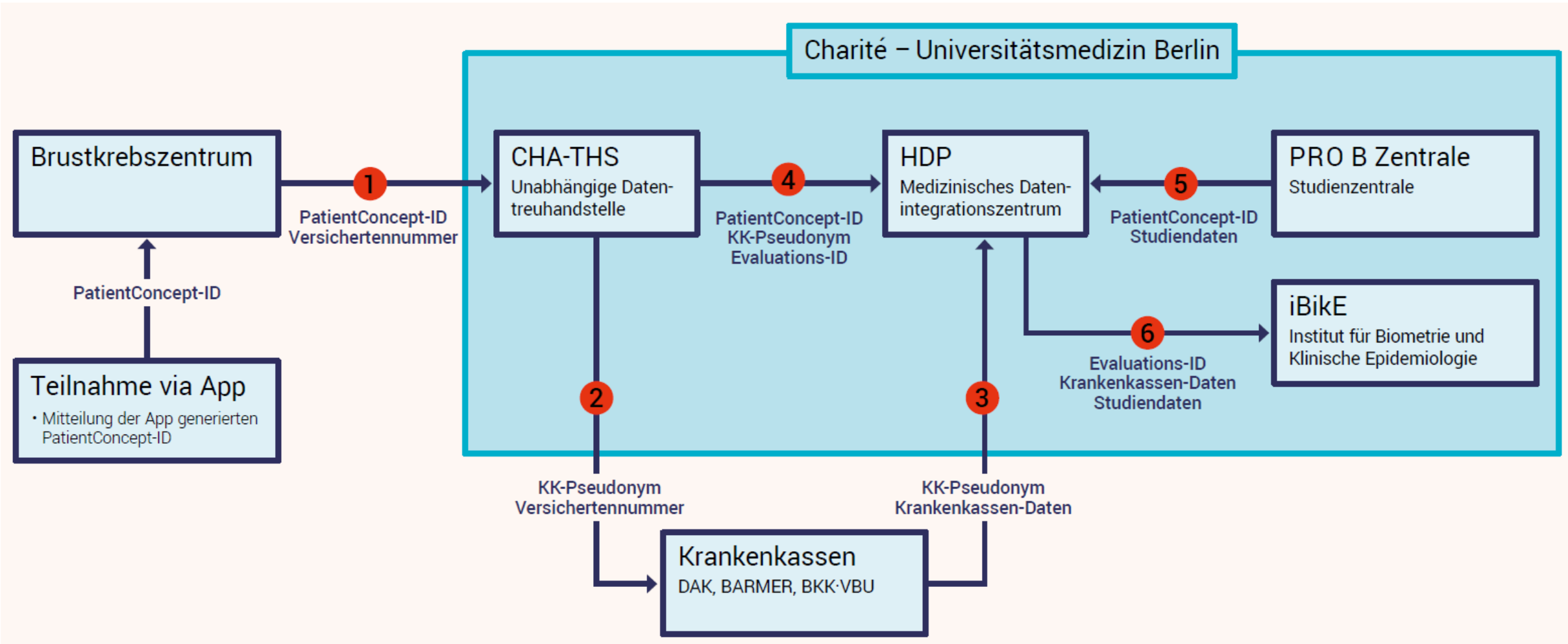

Abbildung 4. Übermittlung der Krankenkassendaten. *KK* Krankenkassen

9 Ethische und juristische Aspekte

9.1 Juristische Grundlage

Die Aufnahme in die Studie erfolgt nach informierter Einwilligung durch die Patientin gemäß anhängender Einwilligungserklärung (siehe Anlage 1). Die Intervention wird auf Rechtsgrundlage eines Vertrags zur Besonderen Versorgung gemäß § 140a SGBV durchgeführt.

9.2 Ethikkommission

Vor Beginn der Rekrutierung wird die Studie samt Patientinneninformation und Einwilligungserklärung sowie entsprechende weitere Dokumente einer erstvotierenden unabhängigen Ethikkommission vorgelegt. Nach Bestimmung der Studienzentren werden die Dokumente den entsprechenden lokalen Ethikkommissionen zur Beratung vorgelegt. Eine schriftliche positive Abstimmung der zuständigen Ethikkommission ist eine Voraussetzung für den Beginn dieser Studie.

9.3 Patientinneninformation und Einwilligungserklärung

Die Patientinneninformation, Datenschutzinformation und Einwilligungserklärung sind in Anlage 1 einsehbar und wurden nach Vorlage der Ethikkommission der Charité sowie der Medizininformatik-Initiative (MII)[1] erstellt.

[1] https://www.medizininformatik-initiative.de/de/medizininformatik-initiative-erhaelt-gruenes-licht-fuer-bundesweite-patienteneinwilligung

9.4 Aufwandsentschädigung

Die teilnehmenden Patientinnen erhalten keine Aufwandsentschädigung.

9.5 Versicherung

Ein studienspezifischer Versicherungsschutz ist nicht vorgesehen.

10 Administrative Aspekte

10.1 Studienfinanzierung

Die Studie wird durch Förderung des Innovationsauschusses des Gemeinsamen Bundesausschusses (Fördernummer 01NVF19013) finanziert.

10.2 Studienberichte

Das verantwortliche evaluierende Institut (iBiKE) wird gemeinsam mit der Studienleitung innerhalb von 6 Monaten nach Studienende einen Evaluationsbericht vorlegen.

10.3 Studienregistrierung

Die Studie wird vor Beginn der Rekrutierungsphase durch die Studienleitung im Deutschen Register Klinischer Studien (DRKS) und im Studienregister der U. S. National Library of Medicine (Clinicaltrials.gov) registriert.

10.4 Publikationen

Alle die Studie betreffenden Informationen sind vor Publikation vertraulich zu behandeln. Die Dissemination des primären Outcomes der Studie in Form von Präsentation(en) und/oder Publikation(en) sind der Studienleitung (PD Dr. med. Maria Margarete Karsten) oder einer von ihr gewählten Vertretung vorenthalten. Kooperierende, die maßgebend zur Konzeption und Durchführung der Studie und/oder zur Patientinnenrekrutierung beigetragen haben, können als Co-Autor*innen zur Publikation beitragen. Die Auswahl der Co-Autor*innen als auch die Reihenfolge der Autor*innenschaft werden bei etwaiger Beschränkung des ausgewählten Journals nach wissenschaftlichem Standard und in Rücksprache mit dem Konsortium durch die Studienleitung festgelegt. Weitere, die Studie betreffende Publikationen sind nur mit schriftlichem Einverständnis und nach Sichtung der Studienleitung gestattet, wobei eine Verhinderung oder Verzögerung nur mit triftigen Gründen möglich ist. Der Studienleitung ist daher mindestens einen Monat vor geplanter Einreichung oder Präsentation ein Entwurf des Abstracts bzw. Manuskripts vorzulegen.

Es sind mindestens zwei Publikationen in Peer-reviewed Journals geplant. Zudem soll die Studie in den Mitgliedermagazinen der kooperierenden Krankenkassen und der kooperierenden Fachgesellschaft (Deutsche Krebsgesellschaft e. V.) vorgestellt werden. Weiterhin sind Veröffentlichungen in Patientinnenmagazinen wie Mamma Mia und mamaZone vorgesehen.

11 Signatur der Studienleitung

Berlin, den 22.08.2023

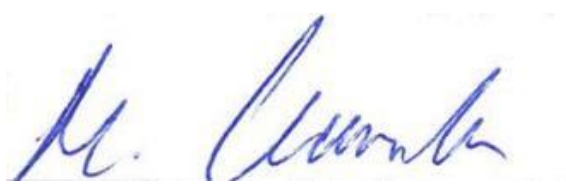

PD Dr. med. Maria Margarete Karsten

Literaturverzeichnis

1. Mariotto AB, Etzioni R, Hurlbert M, Penberthy L, Mayer M. Estimation of the Number of Women Living with Metastatic Breast Cancer in the United States. Cancer Epidemiol Biomarkers Prev. 2017;26(6):809-15.
2. Robert-Koch-Institut. Krebs in Deutschland für 2015/16. Berlin2019.
3. Caswell-Jin JL, Plevritis SK, Tian L, Cadham CJ, Xu C, Stout NK, Sledge GW, Mandelblatt JS, Kurian AW. Change in Survival in Metastatic Breast Cancer with Treatment Advances: Meta-Analysis and Systematic Review. JNCI cancer spectrum. 2018;2(4):pky062.
4. Guidance for industry: patient-reported outcome measures: use in medical product development to support labeling claims: draft guidance. Health Qual Life Outcomes. 2006;4:79.
5. Basch E, Iasonos A, McDonough T, Barz A, Culkin A, Kris MG, Scher HI, Schrag D. Patient versus clinician symptom reporting using the National Cancer Institute Common Terminology Criteria for Adverse Events: results of a questionnaire-based study. The Lancet Oncology. 2006;7(11):903-9.
6. Di Maio M, Gallo C, Leighl NB, Piccirillo MC, Daniele G, Nuzzo F, Gridelli C, Gebbia V, Ciardiello F, De Placido S, Ceribelli A, Favaretto AG, de Matteis A, Feld R, Butts C, Bryce J, Signoriello S, Morabito A, Rocco G, Perrone F. Symptomatic toxicities experienced during anticancer treatment: agreement between patient and physician reporting in three randomized trials. J Clin Oncol. 2015;33(8):910-5.
7. Di Maio M, Basch E, Bryce J, Perrone F. Patient-reported outcomes in the evaluation of toxicity of anticancer treatments. Nature reviews Clinical oncology. 2016;13(5):319-25.
8. Groenvold M, Petersen MA, Idler E, Bjorner JB, Fayers PM, Mouridsen HT. Psychological distress and fatigue predicted recurrence and survival in primary breast cancer patients. Breast cancer research and treatment. 2007;105(2):209-19.
9. Müller V, Nabieva N, Häberle L, Taran F-A, Hartkopf AD, Volz B, Overkamp F, Brandl AL, Kolberg H-C, Hadji P, Tesch H, Ettl J, Lux MP, Lüftner D, Belleville E, Fasching PA, Janni W, Beckmann MW, Wimberger P, Hielscher C, Fehm TN, Brucker SY, Wallwiener D, Schneeweiss A, Wallwiener M. Impact of disease progression on health-related quality of life in patients with metastatic breast cancer in the PRAEGNANT breast cancer registry. The Breast. 2018;37:154-60.
10. Park IH, Lee KS, Ro J. Effects of second and subsequent lines of chemotherapy for metastatic breast cancer. Clin Breast Cancer. 2015;15(1):e55-62.
11. Raskin W, Harle I, Hopman WM, Booth CM. Prognosis, Treatment Benefit and Goals of Care: What do Oncologists Discuss with Patients who have Incurable Cancer? Clin Oncol (R Coll Radiol). 2016;28(3):209-14.
12. Schwartz KL, Simon MS, Bylsma LC, Ruterbusch JJ, Beebe-Dimmer JL, Schultz NM, Flanders SC, Barlev A, Fryzek JP, Quek RGW. Clinical and economic burden associated with stage III to IV triple-negative breast cancer: A SEER-Medicare historical cohort study in elderly women in the United States. Cancer. 2018;124(10):2104-14.
13. Sorensen SV, Goh JW, Pan F, Chen C, Yardley D, Martín M, Knopf K, Benedict A, Giorgetti C, Iyer S. Incidence-based cost-of-illness model for metastatic breast cancer in the United States. Int J Technol Assess Health Care. 2012;28(1):12-21.
14. Basch E, Deal AM, Kris MG, Scher HI, Hudis CA, Sabbatini P, Rogak L, Bennett AV, Dueck AC, Atkinson TM, Chou JF, Dulko D, Sit L, Barz A, Novotny P, Fruscione M, Sloan JA, Schrag D. Symptom Monitoring With Patient-Reported Outcomes During Routine Cancer Treatment: A Randomized Controlled Trial. J Clin Oncol. 2016;34(6):557-65.
15. Basch E, Deal AM, Dueck AC, Scher HI, Kris MG, Hudis C, Schrag D. Overall Survival Results of a Trial Assessing Patient-Reported Outcomes for Symptom Monitoring During Routine Cancer Treatment. JAMA. 2017;318(2):197-8.
16. Denis F, Basch E, Septans A-L, Bennouna J, Urban T, Dueck AC, Letellier C. Two-Year Survival Comparing Web-Based Symptom Monitoring vs Routine Surveillance Following Treatment for Lung Cancer. JAMA. 2019;321(3):306-7.

17. Pocock SJ, Simon R. Sequential treatment assignment with balancing for prognostic factors in the controlled clinical trial. Biometrics. 1975;31(1):103-15.
18. Cocks K, King MT, Velikova G, de Castro G, Jr., Martyn St-James M, Fayers PM, Brown JM. Evidence-based guidelines for interpreting change scores for the European Organisation for the Research and Treatment of Cancer Quality of Life Questionnaire Core 30. Eur J Cancer. 2012;48(11):1713-21.
19. Cortes J, Hudgens S, Twelves C, Perez EA, Awada A, Yelle L, McCutcheon S, Kaufman PA, Forsythe A, Velikova G. Health-related quality of life in patients with locally advanced or metastatic breast cancer treated with eribulin mesylate or capecitabine in an open-label randomized phase 3 trial. Breast cancer research and treatment. 2015;154(3):509-20.
20. Borm GF, Fransen J, Lemmens WA. A simple sample size formula for analysis of covariance in randomized clinical trials. J Clin Epidemiol. 2007;60(12):1234-8.
21. Dawood S, Broglio K, Ensor J, Hortobagyi GN, Giordano SH. Survival differences among women with de novo stage IV and relapsed breast cancer. Annals of oncology : official journal of the European Society for Medical Oncology. 2010;21(11):2169-74.
22. Largillier R, Ferrero JM, Doyen J, Barriere J, Namer M, Mari V, Courdi A, Hannoun-Levi JM, Ettore F, Birtwisle-Peyrottes I, Balu-Maestro C, Marcy PY, Raoust I, Lallement M, Chamorey E. Prognostic factors in 1,038 women with metastatic breast cancer. Annals of oncology : official journal of the European Society for Medical Oncology. 2008;19(12):2012-9.
23. Kast K, Link T, Friedrich K, Petzold A, Niedostatek A, Schoffer O, Werner C, Klug SJ, Werner A, Gatzweiler A, Richter B, Baretton G, Wimberger P. Impact of breast cancer subtypes and patterns of metastasis on outcome. Breast cancer research and treatment. 2015;150(3):621-9.
24. Soni A, Ren Z, Hameed O, Chanda D, Morgan CJ, Siegal GP, Wei S. Breast Cancer Subtypes Predispose the Site of Distant Metastases. American Journal of Clinical Pathology. 2015;143(4):471-8.
25. Lin NU, Bellon JR, Winer EP. CNS Metastases in Breast Cancer. Journal of Clinical Oncology. 2004;22(17):3608-17.
26. Bower JE, Wiley J, Petersen L, Irwin MR, Cole SW, Ganz PA. Fatigue after breast cancer treatment: Biobehavioral predictors of fatigue trajectories. Health psychology : official journal of the Division of Health Psychology, American Psychological Association. 2018;37(11):1025-34.
27. Bower JE, Ganz PA, Desmond KA, Rowland JH, Meyerowitz BE, Belin TR. Fatigue in breast cancer survivors: occurrence, correlates, and impact on quality of life. J Clin Oncol. 2000;18(4):743-53.
28. Bower JE. Cancer-related fatigue--mechanisms, risk factors, and treatments. Nature reviews Clinical oncology. 2014;11(10):597-609.
29. Andrykowski MA, Donovan KA, Laronga C, Jacobsen PB. Prevalence, predictors, and characteristics of off-treatment fatigue in breast cancer survivors. Cancer. 2010;116(24):5740-8.

Abkürzungsverzeichnis

CRO: Clinical Research Organisation (Klinische Forschungsorganisation);

CSC: Clinical Study Centre (Klinisches Studienzentrum);

DKG: Deutsche Krebsgesellschaft;

ECOG: Eastern Cooperative Oncology Group;

EORTC: European Organisation for Research and Treatment of Cancer;

GCP: Good Clinical Practice (Gute klinische Praxis);

HER: Human epidermal growth factor receptor 2;

HR: Hormonrezeptor;

HRQoL: Health-related quality of life (Gesundheitsbezogene Lebensqualität);

iBiKE: Institut für Biometrie und Klinische Epidemiologie;

ICER: Incremental cost-effectiveness ratio (Inkrementelles Kosten-Nutzen-Verhältnis);

ICMJE: International Committee of Medical Journal Editors;

MAR: Missing at random;

MBC: Metastatic breast cancer (Metastasierter Brustkrebs);

PRO: Patient-reported outcome;

QALY: Quality-adjusted life year (Qualitätsadjustiertes Lebensjahr);

QoL: Quality of life (Lebensqualität);

SAP: Statistischer Analyseplan

Anlagen

1. Information für Patientinnen, Datenschutzinformation und Einwilligungserklärung
2. Datenschutzkonzept

STATISTISCHER ANALYSEPLAN

PRO B – Patient Reported Outcomes bei Brusterkrankungen

Neue Wege in der Versorgung bei metastasiertem Brustkrebs

Kurztitel: PRO B

Intervention: Wöchentliche PRO-Erhebung über eine Smartphone-App, Arztkontakt bei Verschlechterung, mögliche individuelle Behandlungsmaßnahmen (Interventionsgruppe)

Kontrolle: vierteljährliche PRO-Umfrage über eine Smartphone-App

Studienpopulation: Frauen mit metastasierter Brustkrebs

Förderung: Innovationsausschuss beim G-BA (Förderkennzeichen: 01NVF19013)

Registrierung der Studie: DRKS (Deutsches Register Klinischer Studien) DRKS00024015. Registrierung am den 15. Februar 2021

Analyseplan Version: Version 1.0 (Final)

Datum des Analyseplans: 18. Januar 2024

Studienleitung: PD Dr.med.Maria Margarete Karsten

Biostatistikerinnen:

PD Dr.phil.Ulrike Grittner **Unterschrift**

Dr.rer.medic. Pimrapat Gebert **Unterschrift**

Inhaltsverzeichnis

1. Einleitung ... 3
1.1 Ziel der Studie ... 3
1.2 Studienablauf ... 4
1.3 Einschluss- und Ausschlusskriterien ... 4
1.4 Randomisation ... 5
1.5 Studienverlauf ... 5
1.5.1 Interventionsgruppe ... 5
1.5.2 Kontrollgruppe ... 6
1.6 Fallzahlschätzung ... 6
2. Analysesets ... 7
2.1 Definitionen ... 7
2.2 Anwendung ... 7
3. Variablen für die Analyse ... 7
3.1 Primäre und sekundäre Outcomes ... 7
3.2 Definition für die Ereigniszeitanalyse ... 9
3.2.1 Letztes Kontaktdatum ... 9
3.2.2 Zeit bis zum ersten Wechsel der systemischen Therapie ... 9
3.3 Grundlegende Merkmale und mögliche Confounder ... 9
4. Umgang mit fehlenden Werten ... 11
5. Statistische Auswertungen ... 12
5.1 Analyse des primären Endpunkts ... 12
5.2 Analysen der sekundären Endpunkte ... 12
5.3 Sensitivitätsanalysen ... 14
5.4 Statistische Analysen der Subgruppen ... 14
6. Gesundheitsökonomische Auswertung ... 15
6.1 Ziele ... 15
6.2 Fallzahlschätzung ... 15
6.3 Gesundheitsökonomische Outcomes ... 17
6.4 Nutzung von Gesundheitsressourcen und Kosten ... 17
6.5 Statistische Auswertung ... 17
6.5.1 Analysen der Nutzung von Gesundheitsressourcen und der Kosten ... 18
6.5.2 Qualitätsangepasste Lebensjahre ... 18
6.5.3 Kosten-Effektivitäts-Analyse ... 19
6.5.4 Software ... 19
7. Beispiele für die Darstellung der Tabellen ... 20
8. Referenzen ... 24
9. Abkürzungsverzeichnis ... 26

1. Einleitung

1.1 Ziel der Studie

Brustkrebs ist die häufigste bösartige Erkrankung bei Frauen. Die Entwicklung von Metastasen bei Brustkrebspatientinnen beeinträchtigt das Gesamtüberleben und die Lebensqualität der Patientinnen. Heutzutage werden elektronische Patient berichtete Ergebnisse (PROs) verwendet, um Echtzeitdaten von Patienten zu erfassen, z. B. die Einnahme von Medikamenten, Symptome oder den Gesundheitszustand. Es hat sich gezeigt, dass eine intensivierte PRO-Überwachung die Lebensqualität verbessert und ungeplante Krankenhausaufenthalte und Besuche in der Notaufnahme bei Patientinnen mit metastasierendem Brustkrebs verringert [1]. Außerdem wurde ein positiver Einfluss einer intensivierten PRO-Überwachung auf das Überleben nachgewiesen [1, 2]. Daher könnte die frühzeitige Erkennung einer Verschlechterung der Lebensqualität den Patienten Behandlungszyklen mit einer nicht mehr wirksamen Therapie ersparen.

Die PRO B-Studie will untersuchen, ob der Nutzen des PRO-Monitorings aus einem monozentrischen Studiensetting in die deutsche Routineversorgung übertragbar ist und ob sich die positiven Effekte auf das Gesamtüberleben und die Lebensqualität auch bei Brustkrebspatientinnen zeigen lassen.

Die Studienziele laut Protokoll sind in Tabelle 1 dargestellt.

Tabelle 1. Ziele der Studie

**Primäres Ziel**
Nachweis, dass Patientinnen, die wöchentliche PRO-Erhebungen in Kombination mit einem Arztkontakt im Falle einer Verschlechterung erhalten, nach 6 Monaten geringere Fatigue-Scores aufweisen als Patientinnen, die vierteljährliche PRO-Erhebungen erhalten
**Sekundäres Ziel**
Patientinnen in der Interventionsgruppe haben im Vergleich zur Kontrollgruppe<br>… nach 6 und 12 Monaten einen höheren Summen-Score der gesundheitsbezogenen Lebensqualität (HRQoL)<br>… nach 6 und 12 Monaten einen höheren Score im Bereich körperliche Funktion<br>… nach 12 Monaten einen niedrigeren Score im Bereich Fatigue<br>… nach 6 und 12 Monaten eine geringere Anzahl an Krankenhaus- und Notaufnahmeaufenthalten<br>… eine verkürzte Zeit bis zum ersten Wechsel der systemischen Therapie aufgrund der frühzeitigen Erkennung des Fortschreitens der Krankheit haben<br>… nach 12 Monaten eine erhöhte Überlebensrate bei viszeraler Metastasierung<br>… nach 12 Monaten eine erhöhte Überlebensrate bei triple negativer Mammakarzinomerkrankung

1.2 Studienablauf

PRO B ist eine prospektive, multizentrische, randomisierte, kontrollierte, nicht verblindete zweiarmige Studie mit 1:1-Randomisierung. In der Interventionsgruppe erhalten die Patientinnen wöchentliche PRO-Erhebungen und werden bei einer Verschlechterung der PRO-Werte einen Alarm generiert. Dieser Alarm wird durch die kooperierenden Studienzentren innerhalb von 48 Stunden quittiert und dokumentiert. Der Arzt entscheidet gemeinsam mit der Patientin über mögliche weitere Interventionen. Die Patientinnen in der Kontrollgruppe erhalten alle 3 Monate PRO-Erhebungen und werden standardmäßig behandelt und ohne Alarm generiert.

Das gesamte Studiendesign ist in Abbildung 1 grafisch dargestellt.

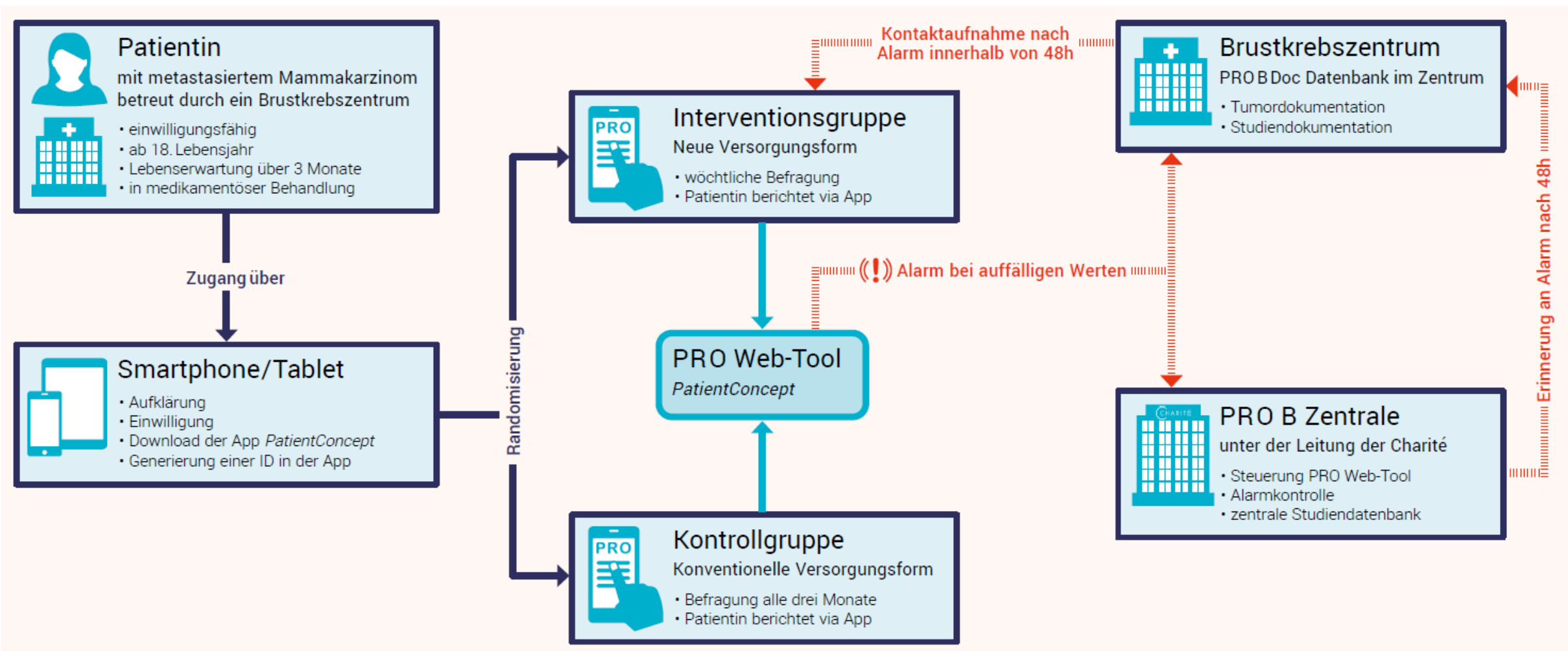

Abbildung 1. Individueller Studienablauf

1.3 Einschluss- und Ausschlusskriterien

Teilnahmeberechtigt sind Patientinnen, die weiblich, älter als 18 Jahre sind, Deutsch lesen und verstehen können, eine medikamentöse Behandlung für MBC mit einer Lebenserwartung von mehr als 3 Monaten zum Zeitpunkt der Teilnahme erhalten, bei einer der drei teilnehmenden Krankenkassen versichert sind und in einem teilnehmenden Brustkrebszentrum behandelt werden. Darüber hinaus sind ein Internetzugang über ein Smartphone oder Tablet, ein ECOG-Performance-Status (Eastern Cooperative Oncology Group) von 0 bis 2 und die Bereitschaft zur Teilnahme an einer wöchentlichen, online-basierten PRO-Erhebung erforderlich.

Ausgeschlossen werden Patientinnen, die keine aktive Tumortherapie erhalten (Comfort Care) oder nicht alle der oben genannten Einschlusskriterien erfüllen.

1.4 Randomisation

Eine stratifizierte 1:1-Randomisierung erfolgt nach den folgenden Strata:

- Zentren
- Arten der Fernmetastasierung (3 Gruppen)
  - ossäre Metastasen & Hautmetastasen & Lymphknotenmetastasen
  - viszerale Metastasen (ein Organ)
  - Gehirnmetastasen & multiple Metastasen
- histologischer Befund (2 Gruppen)
  - HR+/HER− oder HR+/HER+
  - HR−/HER+ oder HR−/HER−

Die adaptive Randomisierung wird mit der elektronischen Randomisierungssoftware secuTrial® durchgeführt, die vom Clinical Study Centre (CSC) der Charité - Universitätsmedizin Berlin verwaltet wird. Die Randomisierung erfolgt nach der schriftlichen Einverständniserklärung, dem Herunterladen der Anwendung und vor der App-basierten Basiserhebung (einschließlich soziodemografischer Informationen und Anamnese). Die Gruppenzuteilung durch secuTrial® erfolgt automatisch für einzelne Patienten ohne vordefinierte Reihenfolge nach Prüfung der Einschlusskriterien, Abgabe der Einverständniserklärung und Eingabe der Patientenmerkmale der Strata-Variablen durch das Studienteam.

1.5 Studienverlauf

1.5.1 Interventionsgruppe

Die Patientinnen der Interventionsgruppe erhalten eine wöchentliche PRO-Umfrage über die App (immer am selben Wochentag). Die PRO-Erhebung basiert auf der Operationalisierung von HRQoL durch die European Organisation of Research and Treatment of Cancer (EORTC) Quality of Life Group. Ihr Kerninstrument, der EORTC QLQ-C30, umfasst fünf funktionale Dimensionen (körperliche Funktion, Rollenfunktion, emotionale Funktion, kognitive Funktion, soziale Funktion) und neun Symptomskalen (Müdigkeit, Übelkeit, Schmerzen, Dyspnoe, Schlaflosigkeit, Appetitlosigkeit, Verstopfung, Durchfall, finanzielle Schwierigkeiten)[3]. Diese Bereiche werden mit speziell für die Studie optimierten Fragebögen mit Items aus der entsprechenden EORTC-Itembank erfasst [4]. Mithilfe der Item-Response-Theorie wurden Kurzformulare mit unterschiedlicher Länge und unterschiedlichem Item-Inhalt für jeden Bereich konstruiert, die so zugeschnitten sind, dass eine maximale Genauigkeit im erwarteten Bereich der Punktwerte erreicht wird. Die PRO-Werte werden automatisch mit den Werten der Vorwoche verglichen, um kurzfristige Veränderungen festzustellen, und mit dem Mittelwert der

Werte 3, 4 und 5 Wochen zuvor, um langfristige Veränderungen der HRQoL zu erkennen. Im Falle einer klinisch relevanten Verschlechterung der PRO-Scores wird ein Alarm ausgelöst.

1.5.2 Kontrollgruppe

Die Patientinnen im Kontrollarm erhalten alle 3 Monate eine PRO-Erhebung über die App, aber es wird kein Alarm ausgelöst, wenn sich die PRO-Werte verschlechtern, und somit kein Kontakt hergestellt.

1.6 Fallzahlschätzung

In einer Meta-Analyse zu Studien zu Veränderungen des EORTC QLQ-C30 analysierten Cocks et al. 35 Studien, die eine Verbesserung der Fatigue Skala Werte berichteten. Mittlere Verbesserungen der Werte um 5 Punkte wurden dort berichtet [5]. Cocks et al. erstellten Richtlinien für die Interpretation längsschnittlicher Veränderungen auf der Fatigue-Skala, wobei Werte ab durchschnittlich 4 Punkten Verbesserung als kleine Verbesserung und Werte ab 9 Punkten als mittlere Verbesserung einzuschätzen sind. In unserer Studie geht es allerdings um einen Gruppenvergleich zwischen Treatment und Kontrollgruppe. Mit den Effektschätzungen haben wir uns nach den Angaben von Cocks et al. gerichtet. Allerdings erwarten wir aufgrund des Zwei-Gruppen-Vergleichs sehr viel größere Streuungsmaße und schätzen damit konservativ eine zu erreichende standardisierte Effektstärke von etwa 0,2 (mittlere Differenz zum Beispiel 5 Punkte. Gemeinsame Standardabweichung 25,2 Punkte). Eine solche Streuung ist vergleichbar mit den Angaben von Cortes et al. 2015 [6].

Da außerdem die Analyse von bestimmten Subgruppen, zum Beispiel Patientinnen mit viszeralen Metastasen, besonders relevant ist, wird eine Fallzahl angestrebt, die auch für Subgruppenanalysen ausreichend groß ist. Die Rekrutierung von circa 500 Patientinnen pro Gruppe wird als realistisch angesehen. Bei einer erwarteten Drop-Out-Rate von 20% resultiert dies in ungefähr 400 vollständigen Datensätzen pro Behandlungsarm. Wenn ein minimaler klinisch relevanter Effekt von 5 Punkten Mittelwertunterschied auf der Fatigue-Skala des EORTC QLQ-C30 vorliegt, wie von Cocks et al. berichtet (bei einer gemeinsamen Standardabweichung von 25), hat ein zweiseitiger t-Test mit einem Signifikanzniveau von 5% eine Power von 80,65%. Diese Berechnung wurde mit nQuery (Version 8.3.1.0) [7] durchgeführt. Die Fallzahl ist somit auch für die Detektion kleiner Effektstärken (0,2 oder größer) ausreichend und trägt der Anzahl relevanter Subgruppen in der Patientinnen Population Rechnung.

Da die geplante Stichprobengröße nicht im ursprünglich intendierten Rekrutierungszeitraum erreicht werden kann, wird der Rekrutierungszeitraum um vier Monate verlängert. Die insgesamt erwartete Stichprobengröße beträgt 400 Patientinnen pro Gruppe. Bei einer erwarteten Drop-out-Rate von 20 % ergibt dies etwa 320 vollständige Datensätze pro

Behandlungsgruppe. Diese Fallzahl ist somit für den Nachweis von Effektgrößen von 0,2 mittels zweiseitigem t-Test für unabhängige Stichproben mit einem Signifikanzniveau von 5% und einer Power von 71,4% ausreichend. Wenn die Effektgröße 0,225 oder größer ist, kann eine Power von mindestens 81,1 % erreicht werden.

2. Analysesets

2.1 Definitionen

Der vollständige Analysesatz (full analysis set) umfasst alle Probanden, die randomisiert wurden und mindestens die PRO-Erhebung beantwortet haben. Patienten, die nie auf die PRO-Erhebung antworten, werden nicht als vollständiger Analysesatz betrachtet (modified ITT-Population). Im Falle fehlender Werte werden zur Schätzung der fehlenden Werte Methoden der multiplen Imputation verwendet. Der Analysesatz pro Protokoll (per protocol analysis set) umfasst alle Probanden, die wie geplant mindestens 6 Monate lang eine Interventions- oder Kontrollbehandlung erhielten.

2.2 Anwendung

Die primäre Analyse wird unter Verwendung des vollständigen Analysesatzes durchgeführt, einschließlich der geschätzten Werte aus mehrfachen Imputationen für fehlende Werte (ITT). Als Sensitivitätsanalyse wird eine Analyse des primären Ergebnisses aus dem Analyseset gemäß Protokoll verwendet.

3. Variablen für die Analyse

3.1 Primäre und sekundäre Outcomes

In der folgenden Tabelle 2 werden der primäre Endpunkt und die sekundären Endpunkte der Studie aufgeführt. Weitere Variablen, die im Zusammenhang mit der Randomisierung oder als mögliche Confounder relevant sind, werden für die Baseline-Befragung und das Studiendokumentationssystem (eCRF in PRO B Doc) definiert. Die Datenerhebung in PRO B Doc erfolgt alle drei Monate im Rahmen der regulären klinischen Vorstellung im behandelnden Brustkrebszentrum.

Die oben genannten Outcomes (nach 6 und 12 Monaten) liegen am Ende der Rekrutierungs- und Interventionsphase für alle eingeschlossenen Patientinnen (abzüglich der Dropouts) vor. Die primären und sekundären Outcomes werden aber auch für spätere Messzeitpunkte (bis max. 24 Monate nach Einschluss) analysiert, jeweils mit einer kleineren n, um den PRO-

Verlauf für metastasierte Brustkrebspatientinnen über einen möglichst langen Zeitraum abbilden zu können.

Hinweis zum Zeitpunkt der Messung der PROs

Falls die Patientin die wöchentliche PRO-Bewertung versäumt hat, werden die PRO-Werte durch die beobachteten PRO-Werte vor oder nach (+/- 1 Woche) den analysierten Zeitpunkten zu Beginn der Studie (0 Woche), nach 3 Monaten (12 Wochen), nach 6 Monaten (24 Wochen), nach 9 Monaten (36 Wochen) und nach 12 Monaten (48 Wochen) ersetzt. Wenn die Patientin beispielsweise die PRO-B-Erhebung in der 12. Woche nicht beantwortet hat, aber PRO-Daten in der 11. Woche beobachtet wurden, werden die PRO-Daten für die 12. Wenn beide Bewertungen beobachtet werden (vor und nach dem fehlenden Zeitpunkt), wird die vor dem fehlenden Zeitpunkt erhaltene Bewertung verwendet.

Tabelle 2. Übersicht primärer und sekundärer Outcomes

Variable	Datenquelle	Erhebungszeitpunkt				
		**Baseline**	**Wöchentlich in Interventionsgruppe ; alle 3 Monate in Kontrollgruppe**	**6 Monate**	**12 Monate**	**24 Monate**
**Primärer Outcome**						
Fatigue Score (EORTC)	Studienspezifischer Fragebogen aus EORTC Itembank	x	x	x	x	x
**Sekundäre Outcomes**						
Physical functioning score (EORTC)	Studienspezifischer Fragebogen aus EORTC Itembank	x	x	x	x	x
Health-related Quality of Life Summenscore (EORTC)	Studienspezifischer Fragebogen aus EORTC Itembank	x	x	x	x	x
Anzahl der Krankenhaus- und Rettungsstellenaufenthalte	Krankenkassen-daten				x	
Gesamtüberleben bei viszeraler Metastasierung	PRO B Doc, Krankenkassen-daten				x	x*
Gesamtüberleben bei triple negativem Mammakarzinom	PRO B Doc, Krankenkassen-daten				x	x*
Zeit bis zum ersten Wechsel der systemischen Therapie	PRO B Doc				x	x*
EQ-5D		x		x	x	x

*aus PRO B Doc

3.2 Definition für die Ereigniszeitanalyse

3.2.1 Letztes Kontaktdatum

Das Datum des letzten Kontakts wird für lebende Patientin auf der Grundlage des letzten der folgenden Daten ermittelt:

- Untersuchungsdaten (z. B. Labor, Entlassungsdatum usw.)
- Letztes Datum der Beantwortung der PRO-Umfrage
- Datum des Studienendes

Das Datum des letzten Kontakts wird für die Zensierung der Patientin in der Analyse der Zeit bis zum Ereignis verwendet.

3.2.2 Zeit bis zum ersten Wechsel der systemischen Therapie

Die Zeit bis zum ersten Wechsel der systemischen Therapie wird als die Zeitspanne zwischen dem Datum der Randomisierung und dem Datum des ersten Wechsels der systemischen Therapie definiert, und die Zensierung erfolgt zum Datum der letzten Nachuntersuchung oder zum Datum des Studienendes.

3.3 Grundlegende Merkmale und mögliche Confounder

Potenzielle Confounder-Faktoren, die bei allen Analysen berücksichtigt werden, sind im Folgenden aufgeführt:

- Alter bei Randomisierung (Jahre)
- Art der Metastasen (Knochen-, Lymphknoten-, Viszeral-, Gehirn- oder Mehrfachmetastasen)
- Hormonrezeptorstatus (HR+ oder HR-)
- Art der systemischen Therapie zum Zeitpunkt der Randomisierung (endokrin, Chemotherapie, Anti-Her2-Therapie, Immuntherapie, gezielte Therapie und andere)
- ECOG-Status bei Studienbeginn (ECOG 0, 1, 2)
- Wechselnde Alarmkriterien (vor und nach dem 01.09.2022)
- Sozioökonomische und anamnestische Parameter als mögliche Confounder einmalig bei der Ersterhebung:
    - Familienstand (mehrere Kategorien)
    - Vorhandensein von pflegebedürftigen Angehörigen oder minderjährigen Kindern (≤14 Jahre) im Haushalt (ja oder nein)
    - Bildungsstatus (mehrere Kategorien, gemäß ISCED [8])
    - Beschäftigungsstatus (ja oder nein)
    - Einkommen (mehrere Kategorien)
    - Migrationshintergrund (ja oder nein)

  - Komorbiditätsindex des National Cancer Institute (NCI) (numerisch)

Neben der Brustkrebs-spezifischen Anamnese (z. B. familiäre Krebsbelastung) ermöglicht die Befragung über die Smartphone-Applikation auch die Erhebung eventueller Vorerkrankungen (vorläufiger Fragebogen s. u.):

- kardiale und kardiovaskuläre Vorerkrankungen (arterielle Hypertonie, koronare Herzerkrankung, Angina pectoris, Myokardinfarkt, Herzinsuffizienz, Vorhofflimmern, Klappenvitien)
- vaskuläre Vorerkrankungen (tiefe Venenthrombose, periphere arterielle Verschlusskrankheit, Lungenarterienembolie)
- cerebrovaskuläre Erkrankungen (TIA, Apoplex)
- hämatologische Vorerkrankungen (Anämie)
- pneumologische Vorerkrankungen (Asthma bronchiale, COPD, Schlafapnoe)
- endokrine/ metabolische Vorerkrankungen (Hyperlipidämie, Diabetes mellitus (therapiebedürftig ohne und mit Endorganschäden), Hypothyreose, Adipositas)
- rheumatologische Vorerkrankungen (Polymyalgia rheumatica, Lupus erythematodes, rheumatoide Arthritis, Polymyositis)
- psychische Vorerkrankungen (Depression, Angststörung)
- neurologische Vorerkrankungen (Migräne, Krampfanfälle, Gleichgewichtsstörungen, Neuropathie, Hemiplegie, Demenz)
- gastrointestinale Vorerkrankungen (gastrointestinale Ulcera, chronisch entzündliche Darmerkrankung, Divertikulitis, Reflux, Reizdarmsyndrom, leichte Lebererkrankung (Leberzirrhose ohne portale Hypertonie), schwere Lebererkrankung (Leberzirrhose mit portaler Hypertonie, Ösophagusvarizenblutung))
- gynäkologische Vorerkrankungen (Infertilität, Abort, Dys- bzw. Hypermenorrhoe, Endometriose, Ovarialzysten)
- urogenitale Vorerkrankungen (Nephro- bzw. Urolithiasis, Niereninsuffizienz, Dialysepflicht, Miktionsbeschwerden, Harnwegsinfekte, Inkontinenz)
- Infektionserkrankungen (HIV, AIDS)
- onkologische Vorerkrankungen (andere Tumorerkrankungen (mit und ohne Metastasen), Leukämie, Lymphom)
- Voroperationen
- Suchtmittelanamnese (Alkohol, Nikotin)

Wir vermuten einen nicht unerheblichen Einfluss der oben beschriebenen Komorbiditäten auf den primären Endpunkt der Studie (Fatigue). Sie müssen daher sowohl in der Interpretation der Messwerte im laufenden Projekt als auch in der abschließenden Auswertung (z. B. als

Komorbiditätsindex des National Cancer Institute (NCI) (unterstrichene Erkrankungen)) berücksichtigt werden.

4. Umgang mit fehlenden Werten

Der Umgang mit fehlenden Daten basiert auf einem Estimand-Rahmen, der von der ICH-Leitlinie (International Council for Harmonization of Technical Requirements for Pharmaceuticals for Human Use) vorgeschlagen wird [9]. Tabelle 3 zeigt den Analyseplan der PRO B-Daten auf der Grundlage eines Schätzerrahmens für unser primäres Ergebnis. Für Patientinnen, die während des Studienzeitraums verstorben oder zu sehr schlecht fühlen sind, werden für Werte wie Lebensqualität oder körperliche Funktionsfähigkeit die niedrigstmöglichen Werte verwendet (missing not at random, MNAR) [10]. Fehlende Daten aufgrund von Abbruch oder Verlust der Nachverfolgung werden mittels multipler Imputation mit multipler Imputation mit chained equations (MICE) erfasst, wobei davon ausgegangen wird, dass die fehlenden Daten zufällig fehlen (missing at random, MAR oder missing completely at random, MCAR). Es werden dreißig vollständige Datensätze erstellt. MICE wird auf der Grundlage des Predictive-Mean-Matching für die kontinuierlichen Variablen, ordinale logistische Regressionsmodelle für ordinale Variablen und binäre logistische Regressionsmodelle für binäre Variablen durchgeführt. Aufgrund der längeren Rekrutierungszeit von 4 Monaten werden die spät rekrutierten Patientinnen nur 6 Monate lang vollständig beobachtet. Für diese Patientinnen werden keine sekundären Endpunkte nach 12 Monaten mittels Imputationsmethoden geschätzt.

Tabelle 3. Analyse der PRO B-Daten im Rahmen der Schätzung

**Wissenschaftliche Forschungsfrage**	**Ist der mit dem EORTC CAT Core gemessene durchschnittliche Ermüdungswert nach sechs Monaten in der Interventionsgruppe niedriger (besser) als in der Kontrollgruppe?**
**Merkmale der Schätzung**	
**Intervention**	Intervention: Patienten erhalten wöchentliche PRO-Erhebungen mit einem Alarmkontakt im Falle einer Verschlechterung der PRO-Werte<br>Kontrolle: Patienten erhalten vierteljährliche PRO-Erhebungen ohne Alarmkontakt im Falle einer Verschlechterung der PRO-Werte
**Zielpopulation**	Teilnahmeberechtigt sind Patientinnen, die weiblich, älter als 18 Jahre sind, Deutsch lesen und verstehen können, eine medikamentöse Behandlung für MBC mit einer Lebenserwartung von mehr als 3 Monaten zum Zeitpunkt der Teilnahme erhalten. Darüber hinaus sind ein Internetzugang über ein Smartphone oder Tablet, ein ECOG von 0 bis 2 und die Bereitschaft zur Teilnahme an einer wöchentlichen, online-basierten PRO-Erhebung erforderlich.
**Endpunkt von Interesse**	Fatigue-Scores nach 6 Monaten
**Behandlung von interkurrenten Ereignissen**	
**Abbruch der Behandlung**	
- Überlastung durch die PRO-Erhebung	Annahme fehlender Daten nach MAR, implizite Imputation der Daten nach der MICE mit *m* = 30 Imputationen
- nicht mehr Interesse oder Relevanz	Annahme fehlender Daten nach MAR, implizite Imputation der Daten nach der MICE mit *m* = 30 Imputationen
- Patienten fühlen sich zu krank, um weiter an der Studie teilzunehmen	Unter der Annahme, dass das Fehlen nicht zufällig ist, wird das Worst-Case-Szenario angewendet
**Verlust bis zur Nachbeobachtung**	Annahme fehlender Daten nach MAR, implizite Imputation der Daten nach der MI-Methode mit m = 30 Imputationen
**Tod**	Unter der Annahme, dass das Fehlen nicht zufällig ist, wird das Worst-Case-Szenario angewendet
**Schätzer**	Lineares gemischtes Modell, bereinigt um Basislinien- und Stratifikationsfaktoren. Das Studienzentrum wird als zufälliger Intercept gesetzt
**Zusammenfassende Maßnahme**	Mittlere Differenz der Müdigkeitswerte (IG - CG) und 95%CI (zweiseitig)
**Auswertung**	Die mittlere Differenz der Ermüdungswerte und die obere 95%CI-Grenze sind kleiner als 0, d.h. die Interventionsgruppe ist der Kontrollgruppe überlegen.

MAR= missing at random, MBC = metastatic breast cancer, ECOG = Eastern Cooperative Oncology Group, MICE = multiple imputation by chained equation, PRO = patient-reported outcome

5. Statistische Auswertungen

Für jede Gruppe werden deskriptive Statistiken und die Anzahl der in der Berechnung berücksichtigten Teilnehmer (n) angegeben. Für kontinuierliche Variablen werden je nach Verteilung Mittelwert mit Standardabweichung (SD), Median, Interquartilsbereich (IQR), Minimum und Maximum angegeben. Bei kategorialen Daten werden die absoluten und relativen Häufigkeiten für jede Kategorie angezeigt. Zur Visualisierung der Daten werden grafische Methoden verwendet, z. B. Box-Plots und Liniendiagramme.

Alle Ergebnisse werden im Rahmen einer Intention-to-treat-Analyse analysiert, wie in 2.2 beschrieben. Dies ist das primäre Analyseset für die Auswertungsanalysen.

5.1 Analyse des primären Endpunkts

Der primäre Endpunkt ist der EORTC-T-Score für Fatigue nach 6 Monaten, der auf einem maßgeschneiderten Kurzformular aus der EORTC-Itembank basiert. Die primäre Fragestellung wird im Rahmen einer Intention-to-Treat-Analyse durchgeführt. Alle Stratifizierungsvariablen der Randomisation werden als Kovariaten in allen statistischen Modellen berücksichtigt.

Für die Berechnung der Fallzahl wurde ein zweiseitiger t-Test verwendet, obwohl in der Auswertung ein ANCOVA-Modell verwendet werden soll. Es wurde gezeigt, dass dies eine konservative Vorgehensweise für die Schätzung der Fallzahl von ANCOVA-Modellen ist, da ein ANCOVA-Modell mit $(1 - 2\rho)*n$ Beobachtungen mindestens die gleiche Power wie ein t-Test mit n Beobachtungen hat [11], wobei ρ der Varianzinflationsfaktor (Korrelation zwischen der *baseline*-Messung und der *follow-up*-Messung) ist. Im schlechtesten Fall von $\rho = 0$ führt dies zu einer benötigten Fallzahl gleich dem t-Test. Die Analyse erfolgt im ANCOVA-Modell mit Baseline-Messung als Kovariate sowie Arten der Metastasierung und Histologie als Kovariaten (analog der Stratifizierung der Randomisation). Außerdem wird die Analyse adjustiert für das Alter, Art und Anzahl der Chemotherapien, dem ECOG-Status bei Studieneinschluss, der Computer-Erfahrung [1] und gegebenenfalls für weitere wichtige *Confounder*. Die Heterogenität zwischen den Studienzentren wird durch *random effects* berücksichtigt (gemischtes lineares Regressions-Modell, *random intercept model*).

5.2 Analysen der sekundären Endpunkte

Für die sekundären Endpunkte (Subskalen des EORTC-T-Scores auf der Grundlage einer angepassten Kurzform, die aus der EORTC-Itembank generiert wurde, z. B. globaler Gesundheitszustand/QoL, Funktionsfähigkeit, Symptomen zu verschiedenen Zeitpunkten) werden gemischte lineare Regressionsmodelle ähnlich dem oben beschriebenen Modell für den primären Endpunkt durchgeführt. Studienzentren und Patientinnen werden als zufällige Effekte (random intercept) berücksichtigt. Es wird ein Interaktionsterm zwischen

Studiengruppe und Zeitpunkten in die Modelle eingeschlossen, um zeitpunktspezifische Interventionseffekte schätzen zu können. Durch die Verkürzung der Nachbeobachtung bei einem Teil der Stichprobe in Bezug auf sekundäre Endpunkte, da die Patientinnen nicht 12 Monate lang vollständig beobachtet werden, ergeben sich fehlende Werte in den Outcomes. Für die sekundären Endpunkte werden wir für diese spät rekrutierten Patientinnen keine imputierten Daten schätzen, da wir überhaupt keine späteren Daten von ihnen erhoben haben. Daher werden die Analysen der sekundären Endpunkte nach 12 Monaten auf einem geringeren Stichprobenumfang beruhen als die Analysen für den primären Endpunkt.

Ein Cox-Proportional-Hazard-Modell wird durchgeführt, um das Gesamtüberleben (overall survival) und die Zeit bis zum ersten Wechsel der systemischen Therapie zwischen den Studiengruppen zu vergleichen, wobei die Art der Metastasen, die Histologie und andere potenziell störende Faktoren adjustiert werden. Zusätzlich wird ein zufälliger Effektterm (random intercept) für das Zentrum einbezogen, um die Heterogenität der Zentren zu berücksichtigen. Darüber hinaus werden den Time-to-Event-Analysen berücksichtigt, so dass die kürzeren Beobachtungszeiten der später rekrutierten Patientinnen adäquat adressiert werden. Es werden alle verfügbaren Informationen verwendet, und unterschiedliche Beobachtungszeiträume stellen kein Problem dar. Daher wird auch an dieser Stelle keine Schätzung für fehlende Daten vorgenommen. Es werden Hazard Ratios (HR) mit zweiseitigem 95%CI dargestellt. Als deskriptive Diagramme werden Kaplan-Meier-Kurven sowie adjustierte Überlebenskurven auf der Grundlage der Cox-Modelle dargestellt.

Wir werden das Unterverteilungs-Hazards-Ratio (SHR) der Zeit bis zum ersten systemischen Therapiewechsel und dessen 95%CI unter Verwendung der Unterverteilungs-Proportional-Hazards-Modelle von Fine und Gray schätzen, wobei wir davon ausgehen, dass der Tod ein konkurrierendes Risiko ist. Die kumulative Inzidenz für das Fortschreiten der Krankheit wird dargestellt. Wir werden das Cox-Proportional-Hazards-Modell anwenden, wenn das konkurrierende Ereignis weniger als 10 % oder nicht mehr als der Anteil der Krankheitsprogression beträgt, so dass das Modell des konkurrierenden Risikos nicht erforderlich ist [12].

Die primäre Hypothese wird mit einem zweiseitigen Signifikanzniveau von α=0,05 getestet. Alle sekundären Hypothesen werden im Rahmen einer explorativen Untersuchung analysiert. Für die Analysen der sekundären Ergebnisse wird keine Anpassung für Mehrfachtests vorgenommen.

Alle statistischen Tests werden mit Stata MP18 (StataCorp, 2023, College Station, TX, USA) durchgeführt.

5.3 Sensitivitätsanalysen

Wenn ein signifikantes Ergebnis für den primären Endpunkt festgestellt wird, wird eine Sensitivitätsanalyse durchgeführt, um die Robustheit des Ergebnisses der primären Analyse unter der Annahme eines nicht zufällig fehlenden Ergebnisses (MNAR) zu bewerten, indem ein eine referenzbasierte Sensitivitätsanalyse und ein Delta-anpassender Muster-Mischungs-Ansatz für die Tipping-Point-Analyse (TPA) verwendet wird [13, 14]. Bei der referenzbasierten Sensitivitätsanalyse handelt es sich um einen Imputationsansatz für Patienten, die die Intervention abbrechen, und die fehlenden Werte werden wie in der Kontrollgruppe imputiert [14] . Die TPA geht davon aus, dass ein systematischer Unterschied zwischen den bedingten Verteilungen der fehlenden und der beobachteten Daten besteht. Um einen solchen systematischen Unterschied widerzuspiegeln, wird ein Shift-Parameter δ auf das Imputationsmodell angewendet, wenn die fehlenden Datenpunkte imputiert werden. Für jeden Wert von δ werden mehrfach imputierte Datensätze erzeugt, jeder Datensatz wird nach derselben Methode für die Primäranalyse analysiert, und die Analyseergebnisse werden dann nach der Rubins Regel kombiniert. Durch Variation der Werte von δ werden die Auswirkungen fehlender Daten auf die Analyseergebnisse untersucht, um Kipppunkte zu ermitteln (d. h. die Werte, bei denen sich die Schlussfolgerungen zwischen den Studiengruppen von signifikant zu nicht signifikant ändern).

5.4 Statistische Analysen der Subgruppen

Die Subgruppenanalysen werden in einem explorativen Rahmen durchgeführt.

Die folgenden Subgruppen werden dabei auf mögliche differentielle Interventionseffekte analysiert:

- **Metastasierung** (sekundär/therapiert, primär/Therapienaivität)
- **Tumorsubtyp** (HR+/HER2+, HR+/HER2–, HR–/HER2+, TNBC)
- **Ort der Metastasierung** (cerebral, viszeral, ossär, multipel)
- **Alter bei Randomisierung** (<50, ≥50)
- **Therapie bei Studieneinschluss** (Chemotherapie, endokrine Therapie, Anti-Her2- Therapie, Immuntherapie, zielgerichtete Therapie und andere)
- **Beziehungsstatus** (Partnerschaft/Ehe, ledig)
- **Einkommen** (Mediansplit)
- **Depression** (ja/ehemals, nein)
- **BMI** (<30, ≥30 kg/m^2)
- **Veränderung der Alarmkriterien** (vor und nach dem 01.09.2022)

Für die statistischen Analysen der Subgruppen wird zunächst im gemischte lineare Regressionsmodelle (outcome primäres oder sekundäres Outcome, Kovariablen: jeweilige

Baseline Variable, Treatment Variable, Stratifizierungsvariablen der Randomisation, random Intercept für die Zentren) zusätzlich ein Interaktionsterm der Treatment Variable mit der Subgruppe eingeschlossen, um Hinweise für differentielle Treatment Effekte in den Subgruppen zu erhalten. Zusätzlich werden aus den Modellen marginale Effektschätzer für jede Subgruppe und 95% Konfidenzintervalle angegeben.

Subgruppenanalysen zum Gesamtüberleben zwischen Interventions- und Kontrollgruppe werden getrennt für Patientinnen mit viszeralen Metastasen und Patientinnen mit dreifach negativem Brustkrebs durchgeführt.

6. Gesundheitsökonomische Auswertung

Die gesundheitsökonomische Auswertung wird nur bei den Patientinnen durchgeführt, die von den Krankenkassen BARMER, DAK Gesundheit, und BKK·VBU versichert sind.

6.1 Ziele

- Vergleich der durchschnittlichen Kosten zwischen der Interventionsgruppe und der Kontrollgruppe
- Vergleich der Häufigkeit von Krankenhausaufenthalten und Besuchen in der Notaufnahme zwischen beiden Gruppen
- Vergleich der Kosteneffektivität zwischen der Interventionsgruppe und der Kontrollgruppe
- Vergleich der Dauer des Krankenhausaufenthalts zwischen den Studiengruppen

6.2 Fallzahlschätzung

Die Fallzahlschätzung für die gesundheitsökonomische Analyse beruht auf den Angaben von Basch et al. (2016) [1] zum Interventionseffekt für die Lebensqualität gemessen mit dem EQ-5D in Patientinnen und Patienten mit Krebserkrankung mit PRO-Monitoring gegenüber der Standard-Versorgung. Basch et al. (2016) berichten mittlere EQ-5D Werte von 84.8 (95% Konfidenzintervall: 83.2-86.4) in der Interventionsgruppe und 79.5 (95%KI: 76.7-82.2) in der Kontrollgruppe 6 Monate nach Interventionsbeginn (Baseline Werte: Intervention: 86.2, Kontrolle 86.6). Nach Skalierung der Werte auf eine Skala von 0-1 (statt 0-100) ergibt sich nach der Formel von Matthews et al. (1990) nach der Berechnung der QALYs (quality adjusted life years) als Fläche unter der Kurve ein Wert von 0.428 für die Interventionsgruppe ((0.848+0.862)*0.5*0.5 years) und von 0.415 für die Kontrollgruppe ((0.795+0.866)*0.5*0.5 years). Das resultiert in einen Interventionseffekt von 0.012 QALYs. Bezogen auf 12 Monate wäre das ein geschätzter Effekt von 0.0245 QALYs. Wir denken, dass wir einen solchen oder einen ähnlichen Effekt in unserer Studie erreichen können.

Wir gehen des Weiteren davon aus, dass für die neue Intervention etwa 1000 € zusätzlich an Kosten aufzubringen sind. Zur Berechnung des mittleren inkrementellen Kosten-Effektivitäts-Quotienten (ICER) wird die mittlere Kostendifferenz durch die geschätzte QALY Differenz geteilt: ICER=1000€ / 0.0245QALYs = 40,816€. Das ist die mittlere geschätzte Summe, die für ein zusätzliches QALY zu erbringen ist.

Die obere Grenze für diese Aufwendungen wird oft bei 50,000€ festgesetzt. Aus der Studie resultiert zunächst nur eine Schätzung des ICER, da sie auf mittleren Werten der Kostendifferenz und der QALY Differenz zwischen den Gruppen beruht. Somit ist für den ICER keine direkte Fallzahlschätzung möglich. Das 95% Konfidenzintervall (KI) für den ICER erfolgt über bootstrap Verfahren, indem das ICER separat für n=5000 bootstrap samples aus den Originaldaten berechnet wird.

Die Fallzahlschätzung für die gesundheitsökonomische Analyse stützt sich auf die EQ-5D Werte: Ausgehend von den Werten von Basch et al. (2016) bezüglich der EQ-5D Werte in beiden Gruppen nach 6 Monaten, lässt sich eine Effektstärke für den Interventionseffekt von 0.331 (Cohen's d) berechnen. Eine Stichprobengröße von 200 Personen in jeder Gruppe hat eine Power von mehr als 90 %, um einen Mittelwertunterschied von 5.3 (die Differenz zwischen dem Mittelwert der Interventionsgruppe von 84.8 und dem Mittelwert der Kontrollgruppe von 79.5) festzustellen, wenn man davon ausgeht, dass die gemeinsame Standardabweichung 16.01 (berechnet auf Grundlage der Angaben zu den 95%KI bei Basch et al. 2016 und der Stichprobengrößen) beträgt, wobei ein t-Test für zwei Gruppen mit einem zweiseitigen Signifikanzniveau von 5 % verwendet wird.

Aufgrund der zu erwartenden Drop-Out Rate von 20%, könnten bei angestrebten 400 einzuschließenden Patientinnen der beteiligten Krankenkassen nur 320 vollständige Datensätze gewonnen werden. Unvollständige Datensätze werden, insofern von fehlenden Werten im Rahmen der Mechanismen „completely missing at random" (MCAR) oder „missing at random" (MAR) ausgegangen werden kann, mittels multipler Imputationsmethoden [15] unter Einbeziehung der vorhandenen Daten geschätzt, sodass für die gesundheitsökonomische Fragestellung inklusive geschätzter Daten 400 Datensätze zur Verfügung stehen, auch wenn nur 320 vollständige Datensätze vorhanden sein sollten. Selbst wenn nur 320 vollständige Datensätze zur Verfügung stehen, ist die Aussagekraft der Studie größer als 80% (Aussagekraft = 83,9%). Demzufolge ist es ausreichend, wenn für die gesundheitsökonomische Fragestellung 400 Patientinnen der beteiligten Krankenkassen rekrutiert werden.

Da die ursprünglich geplante Stichprobengröße nicht erreicht wird, ist für die gesundheitsökonomische Analyse eine Stichprobengröße von etwa 150 Patientinnen pro Gruppe zu erwarten. Bei einer erwarteten Drop-out-Rate von 20 % ergibt dies etwa 120

vollständige Datensätze pro Gruppe. Die Fallzahl ist somit für den Nachweis von Effektgrößen von mindestens 0,331 mit einer Power von 72,4% ausreichend. Ist die tatsächliche Effektgröße 0,37 oder größer, so ist die Power der Studie mindestens 81,4%.

Wir gehen davon aus, dass diese Powerüberlegung für den EQ-5D, der als Grundlage für die Berechnung der QALYs verwendet wird, die Grundlage für eine robuste Schätzung des ICER bildet, für den keine direkte Fallzahlschätzung möglich ist.

6.3 Gesundheitsökonomische Outcomes

Folgende Aspekte sind von Interesse: alle Behandlungskosten und die Häufigkeit von Krankenhausaufenthalten und Besuchen in der Notaufnahme, die Dauer des Krankenhausaufenthalts und die Lebensqualität (EQ-5D).

6.4 Nutzung von Gesundheitsressourcen und Kosten

Die Nutzung von Gesundheitsressourcen (HRU) und die Kosten werden aus den Leistungsdaten der Krankenkassen berechnet.

Die Inanspruchnahme der Gesundheitsressourcen wurde anhand der Anzahl der ambulanten und stationären Besuche und der Aufenthaltsdauer (Tage) im Krankenhaus bewertet.

Patientenzuzahlungen werden nicht berücksichtigt. Zur Ermittlung direkter Kosten werden diese getrennt voneinander wie folgt berechnet: ambulante Versorgung, stationäre Versorgung (ambulant, teilstationär und stationär), Arzneimittelverordnungen, therapeutische Hilfs- und Heilmittel. Die Ambulante Pflege umfasst alle Kosten für Dienstleistungen. Die stationäre Pflege fasst alle Kosten für Dienstleistungen und verabreichte Medikamente während stationärer Aufenthalte zusammen. Medikamentenverordnungen beinhalten die Kosten für Medikamente im ambulanten Bereich. Hilfsmittel wie beispielsweise Gehhilfen oder Rollstühle sind Geräte zur Unterstützung des Patienten bei der Genesung und der täglichen Versorgung. Heilmittel sind Leistungen wie z. B. Massagen oder Physiotherapie, die durch medizinisches Personal erbracht werden. Insgesamt werden die Gesundheitskosten als Summe der Kosten aller Bereiche berechnet. Aufgrund der ungleichen Beobachtungszeiten werden alle Kosten über den Beobachtungszeitraum gemittelt, so dass die Kosten als Jahreskosten dargestellt werden.

6.5 Statistische Auswertung

Die Darstellung der Baseline-Charakteristika erfolgte mittels absoluter Häufigkeiten und Prozentsätze und für kategorische Variablen und Mittelwert, Standardabweichung (SD), Median, Interquartilsbereich (IQR) für kontinuierliche Variablen.

6.5.1 Analysen der Nutzung von Gesundheitsressourcen und der Kosten

Aufgrund der typischen rechtsschiefen Verteilung von LOS- und Kostendaten haben wir für die Analyse log-transformierte LOS (ln-LOS) und Kosten (ln-Cost) verwendet. Dabei entspricht der Exponent des mittleren beobachteten ln-LOS und ln-Cost dem geometrischen Mittel (GM) der LOS und Kosten. Es wird ein lineares gemischtes Modell (englisch Linear Mixed Models, LMM) durchgeführt, um den Unterschied von ln-LOS und ln-Kosten zwischen den Gruppen mit Patienten und Zentren als zufälligem Intercept zu vergleichen. Die Daten zu den Gesundheitsausgaben sind in der Regel durch einen erheblichen Anteil von Null-Kosten-Beobachtungen für Personen, die in einem bestimmten Zeitraum keine Gesundheitsressourcen in Anspruch genommen haben. Wenn der Anteil der Null-Kosten in der Studie hoch ist, wird ein zweiteiliges Modell verwendet [16, 17]. Im ersten Teil des Modells wird die Wahrscheinlichkeit, eine Gesundheitsdienstleistung in Anspruch zu nehmen, mit Hilfe eines logistischen Regressionsmodells geschätzt. Der zweite Teil des Modells, verallgemeinerte lineare Modelle (GLMs) mit einer Gamma-Verteilung und einem logarithmischen Zusammenhang, schätzt die Kosten unter der Bedingung, dass positive Kosten entstanden sind. Die Akaike-Informationskriterien (AIC), die die Anpassungsgüte messen, werden für den Vergleich der verschiedenen Modelle verwendet, z.B. Log-Transformation.

Die kumulative Inzidenzrate (IR) wird für jedes HRU-Ergebnis dargestellt. Die HRU-Ergebnisse werden mit Hilfe des Poisson-Regressionsmodells oder der negativen Binomialregression verglichen und die Ergebnisse werden als unbereinigte und bereinigte Inzidenzratenverhältnisse (IRRs) mit 95%-Konfidenzintervallen (KIs) dargestellt. Die Überdispersion wird durch den Vergleich der Modelle der Poisson-Regression und der negativen Binomialregression mit Hilfe des Likelihood-Ratio-Tests überprüft. Das am besten geeignete Modell wird ausgewählt.

6.5.2 Qualitätsangepasste Lebensjahre

Qualitätsangepasste Lebensjahre (Quality-adjusted life years; QALYs) sind ein Maß, das die gesundheitsbezogene Lebensqualität (HRQoL) und die Lebenserwartung in einem Index zusammenfasst. In dieser Studie werden die gewonnenen QALYs, die auf eine Verbesserung der Lebensqualität zurückzuführen sind, als QALY-Gewichte bezeichnet und mit den EuroQoL 5 Dimensions (EQ-5D) Werten bewertet.

Der EQ-5D wird zu Studienbeginn, 6 Monate und 12 Monate nach Studieneintritt erhoben. Die QALYs werden individuell für jeden Patienten als Fläche unter der Kurve (AUC) unter Verwendung der zeitgewichteten QALY [18]. Die gesamten QALYs werden als Mittelwert der zeitlich gewichteten individuellen QALYs geschätzt. Die QALYs werden auf 0 gesetzt, wenn

zu diesem Zeitpunkt ein vorzeitiger Tod eintritt. Darüber hinaus wird der Nutzwertindex anhand einer regressionsbasierten Anpassung für den Nutzwertindex zu Beginn der Studie geschätzt [18]. Unvollständige Lebensqualitätsdaten werden als fehlende Werte im Rahmen der Mechanismen "completely missing at random" (MCAR) oder "missing at random" (MAR) unter Verwendung von Imputationsmethoden angenommen [15]. Fehlende Werten zur Lebensqualität werden mittels multipler Imputation mit verketteter Gleichung (MICE) berechnet, wie in 4. Wenn der EQ-5D fehlt, aber EORTC beobachtet wurde, wird der geschätzte EQ-5D mit Hilfe des Mapping-Prozesses angewendet, um den EQ-5D aus den EORTC-Werten vorherzusagen.

Der AUC unter Verwendung des zeitgewichteten QALY wird wie folgt berechnet:

$$time - weighted\ QALY = \frac{1}{12}\sum_{i=0}^{2}\frac{(t_{i+1} - t_i)}{2}(y_i + y_{i+1})$$

y_i ist der Nutzenindexwert bei den Messungen i=0, 1, 2 und zum Zeitpunkt (t_i) =0, 6, 12 Monate

6.5.3 Kosten-Effektivitäts-Analyse

Die Kosten-Effektivität der Intervention wird auf der Grundlage des inkrementellen Kosten-Effektivitäts-Verhältnisses (ICER) mit der Kontrollgruppe verglichen. Das ICER ist definiert als die Kostendifferenz zwischen den beiden Gruppen, geteilt durch die Differenz ihrer Wirkungen. Somit kann die ICER als die mit einem zusätzlichen QALY verbundenen zusätzlichen Kosten interpretiert werden. Die ICER wird wie folgt berechnet:

$$ICER = \frac{Cost_{IG} - Cost_{KG}}{QALYs_{IG} - QALYs_{KG}}$$

In der Kosten-Effektivität-Analyse werden nur die direkten Gesundheitskosten berücksichtigt. Die Kosten und Gesundheitsergebnisse werden aufgrund des Zeithorizonts von einem Jahr nicht diskontiert.

6.5.4 Software

Alle statistischen Analysen werden mit Stata MP18 (StataCorp, 2023, College Station, TX, USA) Durchgeführt.

7. Beispiele für die Darstellung der Tabellen

Um die Richtlinien der Darstellung wissenschaftlicher Ergebnisse einzuhalten, werden die Daten mit den folgenden Tabellen dargestellt.

Tabelle 7.1. Grundlegende Merkmale

**Grundlegende Merkmale**	**Gesamt (n=…)**	**Interventions gruppe (n=…)**	**Kontrollgruppe (n=…)**
**Alter bei Studieneinschluss (Jahre)**			
Mittelwert (SD) [Min, Max]			
**BMI (kg/m²)** – Mean (SD)			
<30			
≥30			
**Beziehungsstatus**			
Verheiratet			
Ledig			
Geschieden/verwitwet			
**Bildungsstatus**			
Niedrige			
Mittel			
Hoch			
**ECOG**			
0			
1			
2			
**Metastasierung**			
Primär/Therapienaivität			
Sekundär/stattgehabte Therapien			
**Ort der Metastasierung**			
Cerebral			
Viszeral			
Ossär			
Multipel			
**Tumorsubtype**			
HR+/HER2+			
HR+/HER2-			
HR-/HER2+			
TNBC			
**Therapie bei Studieneinschluss**			
Chemotherapie			
Endokrine Therapie			
Antikörpertherapie/Hybridtherapie			

Tabelle 7.2. Primärer Endpunkt - Fatiguescore nach 6 Monaten

Gruppe	Fatiguescore nach 6 Monaten Mittelwert (95%CI)	Mittlere Differenz (IG – KG) (95% CI)	p-Werte
**Lineares gemischtes Modell**			
IG			
KG			
**Sensitivitätsanalyse**			
IG			
KG			

IG=Interventionsgruppe, KG=Kontrollgruppe

Tabelle 7.3. Sekundäre Endpunkte

EORTC	Interventions gruppe Mean (95%CI)	Kontrollgruppe Mean (95%CI)	Mittlere Differenz (IG – KG) (95% CI)	p-Werte
**Globaler Gesundheitszustand/QoL**				
Basislinie				
3-monatig				
6-monatig				
9-monatig				
12-monatig				
**Funktionale Skalen**				
Basislinie				
3-monatig				
6-monatig				
9-monatig				
12-monatig				
**Symptom-/Itemskalen**				
Basislinie				
3-monatig				
6-monatig				
9-monatig				
12-monatig				

Tabelle 7.4. Anzahl der Alarme und Kontakte pro Patient (nur Interventionsgruppe)

**Anzahl der Alarme**	
Mittelwert (SD)	
Median (Min, Max)	
**Anzahl der Kontakte**	
Mittelwert (SD)	
Median (Min, Max)	
**Dauer des Kontakts seit dem Alarm (Stunden)**	
Median (Min, Max)	
Sofort	
Innerhalb von 24h	
Innerhalb von 1 bis 3 Tagen	
Mehr als 3 Tage	
**Symptome oder Problembericht aus der Kontaktaufnahme**	**n (%)**
nicht relevant	
Lebensqualität / Gesundheitsstatus	
Körperliche Funktion	
Rollenfunktion	
Emotionale Funktion	
Kognitive Funktion	
Soziale Funktion	
Fatigue	
Übelkeit / Erbrechen	
Schmerz	
Dyspnoe	
Schlaflosigkeit	
Appetitverlust	
Verstopfung	
Durchfall	
Finanzielle Schwierigkeiten	
Andere	

Tabelle 7.5. Die EQ-5D-Nutzwerte nach Studiengruppe und Messzeitpunkt

Messezeitpunkt	Anzahl der Beobachtung	Anzahl der fehle Werten	Mittelwert (SD)	Minimum	Maximum
**Baseline**					
Interventionsgruppe					
Kontrollgruppe					
**6-monatig**					
Interventionsgruppe					
Kontrollgruppe					
**12- monatig**					
Interventionsgruppe					
Kontrollgruppe					

Tabelle 7.6. QALY-Gewichte auf der Grundlage des EQ-5D nach Studiengruppe

	**Interventionsgruppe (n=…)**	**Kontrollgruppe (n=…)**	**Mittlere Differenz (IG – KG) (95%CI)**	**p-Werte**
Baseline				
6-monatig				
12-monatig				
Mittlere time-weighted QALYs (95% CI)				

QALY=quality-adjusted life year

Tabelle 7.7. Ergebnisse der Kosten-Wirksamkeits-Analyse (Intervention vs. Kontrolle)

**Gruppe**	**Gesamtkosten (€)**	**Gesamte QALYs**	**Inkrementelle Kosten (€)**	**Inkrementelle QALY**	**ICER (€)**
Interventionsgruppe					
Kontrollgruppe					

8. Referenzen

1. Basch E, Deal AM, Kris MG, Scher HI, Hudis CA, Sabbatini P, et al. Symptom Monitoring With Patient-Reported Outcomes During Routine Cancer Treatment: A Randomized Controlled Trial. *J Clin Oncol* 2016; 34: 557-565. 2015/12/09. DOI: 10.1200/JCO.2015.63.0830.
2. Denis F, Basch E, Septans AL, Bennouna J, Urban T, Dueck AC, et al. Two-Year Survival Comparing Web-Based Symptom Monitoring vs Routine Surveillance Following Treatment for Lung Cancer. *JAMA* 2019; 321: 306-307. 2019/01/23. DOI: 10.1001/jama.2018.18085.
3. Aaronson NK, Ahmedzai S, Bergman B, Bullinger M, Cull A, Duez NJ, et al. The European Organization for Research and Treatment of Cancer QLQ-C30: A Quality-of-Life Instrument for Use in International Clinical Trials in Oncology. *JNCI: Journal of the National Cancer Institute* 1993; 85: 365-376. DOI: 10.1093/jnci/85.5.365.
4. Petersen MA, Aaronson NK, Arraras JI, Chie WC, Conroy T, Costantini A, et al. The EORTC CAT Core-The computer adaptive version of the EORTC QLQ-C30 questionnaire. *Eur J Cancer* 2018; 100: 8-16. 2018/06/25. DOI: 10.1016/j.ejca.2018.04.016.
5. Cocks K, King MT, Velikova G, de Castro G, Martyn St-James M, Fayers PM, et al. Evidence-based guidelines for interpreting change scores for the European Organisation for the Research and Treatment of Cancer Quality of Life Questionnaire Core 30. *European Journal of Cancer* 2012; 48: 1713-1721. DOI: https://doi.org/10.1016/j.ejca.2012.02.059.
6. Cortes J, Hudgens S, Twelves C, Perez EA, Awada A, Yelle L, et al. Health-related quality of life in patients with locally advanced or metastatic breast cancer treated with eribulin mesylate or capecitabine in an open-label randomized phase 3 trial. *Breast Cancer Res Treat* 2015; 154: 509-520. 2015/11/15. DOI: 10.1007/s10549-015-3633-7.
7. nQuery. Sample size and power calculation. Cork: Statistical Solutions Ltd, 2017.
8. UNESCO. *International Standard Classification of Education. ISCED 1997*. Paris 2006.
9. ICH. Integrated Addendum to ICH E6(R1); 2016. Guideline for Good Clinical Practice E6(R2). Available from: https://database.ich.org/sites/default/files/E6_R2_Addendum.pdf. Accessed 29 November 2023.
10. Grobler AC, Lee KJ, Wong A, Currow DC and Braat S. Handling Missing Data and Drop Out in Hospice/Palliative Care Trials Through the Estimand Framework. *J Pain Symptom Manage* 2022; 63: e431-e439. 20211223. DOI: 10.1016/j.jpainsymman.2021.12.022.
11. Borm GF, Fransen J and Lemmens WA. A simple sample size formula for analysis of covariance in randomized clinical trials. *J Clin Epidemiol* 2007; 60: 1234-1238. 20070606. DOI: 10.1016/j.jclinepi.2007.02.006.

12. Maradit Kremers H, Devick KL, Larson DR, Lewallen DG, Berry DJ and Crowson CS. Competing Risk Analysis: What Does It Mean and When Do We Need It in Orthopedics Research? *J Arthroplasty* 2021; 36: 3362-3366. 20210421. DOI: 10.1016/j.arth.2021.04.015.

13. Ratitch B, O'Kelly M and Tosiello R. Missing data in clinical trials: from clinical assumptions to statistical analysis using pattern mixture models. *Pharm Stat* 2013; 12: 337-347. 2013/01/08. DOI: 10.1002/pst.1549.

14. Bell ML, Floden L, Rabe BA, Hudgens S, Dhillon HM, Bray VJ, et al. Analytical approaches and estimands to take account of missing patient-reported data in longitudinal studies. *Patient Relat Outcome Meas* 2019; 10: 129-140. 20190416. DOI: 10.2147/PROM.S178963.

15. Rubin DB. *Multiple Imputation for Nonresponse in Surveys*. New York, NY: John Wiley & Sons, Inc, 1987.

16. Glick HA, Doshi JA, Sonnad SS and Polsky D. *Economic evaluation in clinical trials*. OUP Oxford, 2014.

17. Manning WG, Basu A and Mullahy J. Generalized modeling approaches to risk adjustment of skewed outcomes data. *Journal of health economics* 2005; 24: 465-488.

18. Manca A, Hawkins N and Sculpher MJ. Estimating mean QALYs in trial-based cost-effectiveness analysis: the importance of controlling for baseline utility. *Health Econ* 2005; 14: 487-496. 2004/10/22. DOI: 10.1002/hec.944.
