# Supplementary material for: Electronic Patient-Reported Outcome Monitoring With Alert-Based Interventions in Metastatic Breast Cancer: A Randomized Clinical Trial
Source: JAMA Oncol. 2026 Aug 6;12(10):1069–78. doi: 10.1001/jamaoncol.2026.2661 (PMC13448833; doi:10.1001/jamaoncol.2026.2661)
Supplement: Supplementary file 2 [file jamaoncol-e262661-s002.pdf]

Supplemental Online Content

Karsten MM, Gebert P, Pross T, et al. Electronic patient-reported outcome monitoring with alert-based interventions in metastatic breast cancer: a randomized clinical trial. *JAMA Oncol*. Published online August 6, 2026. doi:10.1001/jamaoncol.2026.2661

eMethods.

eTable 1. Items included in the EORTC CAT Core short forms

eTable 2. Deterioration cut-points of the EORTC T-score and health transition item

eTable 3. Exploring patients' characteristics at baseline by type of missing data and fully observed data

eTable 4. Comparison of patient characteristics at baseline in patients who had missing data or loss to follow-up between the study group

eTable 5. Baseline patient characteristics in observed data and after multiple imputation (imputed data set, m = 30)

eTable 6. Primary outcome: Fatigue T-score at 6 months

eTable 7. Subgroup analyses of fatigue T-score at 6 months post-randomization

eTable 8. The estimated marginal mean and 95% CI for each time point of the T-scores

eTable 9. Overall survival analysis

eTable 10. Hospitalizations and emergency room visits

eFigure 1. The bar graph represents the PRO questionnaire return rate at each follow-up time point

eFigure 2. Frequency of PRO domains triggering alerts (number of alerts = 3,019)

eFigure 3. Health-related quality of life (HRQoL) T-scores over 12 months

eFigure 4. Emotional functioning T-scores over 12 months

eFigure 5. Estimated marginal mean PRO T-scores over 12 months by study group, stratified by enrollment period

eFigure 6. Kaplan-Meier Curves of overall survival

eFigure 7. Time to first change of systemic therapy

eReferences.

This supplemental material has been provided by the authors to give readers additional information about their work.

© 2026 Karsten MM et al. *JAMA Oncology*.

© 2026 Karsten MM et al. *JAMA Oncology*.

eMethods

1. Stratification randomization factors

Patients were randomly assigned in a 1:1 ratio, using stratified randomization according to the following strata:

1.1 Metastatic site distribution at enrollment

- Bone metastases and/or skin metastases and/or lymph node metastases
- Visceral metastases in one organ
- Visceral metastases in multiple organs and/or brain metastases

1.2 Histological subtype

- HR+/HER2-, HR+/HER2+
- HR-/HER2+, HR-/HER2-

1.3 Study center (52 participating breast cancer centers)

Adaptive randomization was applied using the secuTrial© electronic randomization program. Following written informed consent, patients were assigned automatically to study groups in a non-predefined sequence.

2. Study instruments and data collection

2.1 Demographic data and clinical baseline characteristics

Irrespective of the randomization group, patients received the same set of additional questionnaires at enrollment, containing questions regarding their medical history and socioeconomic situation. Detailed information regarding these baseline characteristics has been published previously.[1-3] Trial-specific clinical data points were recorded at the local sites in a study-specific electronic case report form and monitored through on-site visits and digitally by the Clinical Study Center of the Charité (CSC).

Metastatic presentation (de novo vs recurrent) was classified based on timing of first documented distant metastasis relative to initial breast cancer diagnosis. De novo metastatic breast cancer was defined as documentation of distant metastasis at initial diagnosis or within 90 days thereafter; recurrent metastatic breast cancer was defined as distant metastasis occurring more than 90 days after initial diagnosis. Patients without a documented distant metastasis date were classified as unknown.

Detailed information on prior lines of metastatic therapy before study entry was not collected. Eligibility criteria required patients to be receiving systemic therapy at enrollment, and all subsequent treatment changes during follow-up were prospectively documented.

2.2 Patient-reported outcome measures in the PRO B Trial

The PRO B Trial used the European Organization of Research and Treatment of Cancer Quality of Life Group (EORTC) Computerized Adaptive Testing (CAT) Core items to measure health-related quality of life in both the intervention and control groups. This instrument contained 51 to 73 items (eTable 1), including the QLQ-C30 items, and encompassed five functional and nine symptom domains, as in the original QLQ-C30. We added more items in order to improve the measurement precision of the QLQ-C30. This was deemed necessary in particular for the ultra-short 1-item symptom scales, but we also added items to the functional scales to achieve greater measurement precision. Overall, we developed four different sets (four weeks) of additional items to enable variation between assessments in order to prevent or reduce assessment fatigue.

For the intervention group, the EORTC items varied by week: 51 items in week 1, 54 items in week 2, 51 items in week 3, and 57 items in week 4. Patients in this group received a weekly questionnaire on their personal smartphones, consistently on the same day of the week. Patients in the intervention group were given two days to complete the questionnaire, and notifications prompting patients to complete the questionnaire were sent automatically each day until the questionnaire was completed. The control group received the full set of EORTC items (57 items) every three months (eTable 1). Patients in the control group were given seven days to complete the questionnaire, with daily automated reminders sent until the questionnaire was returned. The application's algorithm did not allow patients in either group to skip questionnaire items.

A single health transition item was evaluated alongside the EORTC CAT Core, using the question: "*How would you describe your current health compared to last week?*" The response options were: much better, a little better, about the same as last week, a little worse, and much worse.

Deterioration cut-points of the functional and symptom scales were based on both measurement precision as well as clinical meaningfulness for the alert threshold. We translated available estimates of a minimal clinically important difference (MCID) to the T-metric score using the rule of thumb of half a standard deviation (SD) and weighted this against the potential measurement precision. This resulted in a lower threshold for longer functional scales, and a higher threshold for short and imprecise symptom scales. Therefore, an automated alert was created and sent to the responsible care team at the treating

breast center in the event that a PRO score deteriorated by 5 points (functional scales) or 10 points (symptom scales), or if there was an indication of deterioration in the single health transition item (eTable 2). Following review of alerts generated during early implementation, the alert criteria were revised on September 1, 2022, to increase alert sensitivity and improve clinical usability. Models were adjusted for enrollment period before vs after this amendment.

The local care team was then responsible for contacting the patient within the next 48 hours to evaluate the deterioration. The time point at which the patient was reached by phone and any action taken had to be documented by the local care team on the electronic case report form. The team had access to the patients' PRO scores, which were graphically displayed in a study-specific web tool. It was also possible for the study center to access the PRO values for the patients in the intervention group at any given time point during the study, independently of any alerts.

3. Patient recruitment

The study started enrollment in May 2021, with an initial plan to recruit 1000 patients by February 2023. However, due to challenges in meeting this target, partly because of restrictions imposed during the COVID-19 pandemic, the recruitment period was extended by 4 months (February 16 to June 15, 2023). Recruitment was stopped before the goal was achieved, primarily due to funding constraints. Patients were followed for up to 12 months after randomization; however, patients enrolled late in the recruitment period could not complete the 9- or 12-month assessments before the administrative study end date (February 15, 2024).

A total of 2008 patients were screened for the study; however, this number was likely underreported due to incomplete screening logs at several study centers. Out of those 2008 patients assessed, 422 were not interested, 287 had no documented reason for non-participation, 152 lacked access to a smartphone or the internet, 86 did not meet eligibility criteria, 78 had health-related limitations, 58 were excluded for other reasons (e.g., insufficient German language proficiency), and 1 was recruited in error.

4. Statistical analysis

Confounding factors

All linear mixed models included the following predefined confounding variables as fixed effects: randomization stratification factors (type of metastasis and HR status), study group, baseline PRO T-scores (numeric), age at randomization (numeric), systemic therapy at randomization (including endocrine, chemotherapy, anti-Her2 therapy, immunotherapy, targeted therapy, and other therapies, each coded as yes/no), patient-reported ECOG status (ordinal, 0 – 4), education level (low, medium, high), family income (<2500, 2500 – 4999, ≥5000 Euros), family status (single, married, separated/divorced/widowed), having children under 14 years old (yes/no), migration (yes/no), depression (yes/no), National Cancer Institute (NCI) comorbidity index (numeric), employment status (yes/no), and enrollment period (before vs after September 1, 2022; corresponding to the modification of alert criteria).

Subgroup analyses

Planned exploratory subgroup analyses[1,3] were performed for the primary outcome using linear mixed models with study center as a random intercept and baseline fatigue T-score as a covariate, the interaction terms between study group and the following factors were explored: type of metastasis (brain or multiple, bone/lymph nodes/skin, visceral), subtype (HR+ and HR-), age at randomization (<50 and ≥ 50 years), systemic therapy at randomization (including endocrine, chemotherapy, anti-Her2 therapy, immunotherapy, targeted therapy), education level (low, medium, high), family income (<2500, 2500 – 4999, ≥5000 Euros), family status (single, married, separated/divorced/widowed), depression (yes/ no), body mass index (BMI < 30 and ≥ 30 kg/m^2), and enrollment period (before vs after September 1, 2022). However, a planned analysis of primary metastasis was not conducted because this information was not fully documented by the study centers.

Additional exploratory subgroup analyses added later included metastatic presentation (De novo and recurrent), self-reported ECOG status, time since metastatic diagnosis (<12 months, 12-24 months, and > 24 months), type of breast cancer centers (outpatient/standard care, specialized care, and tertiary care/university), and enrollment period (May-December 2021, January-December 2022, January-June 2023). These later exploratory subgroup analyses were descriptive and were added after the original SAP-specified set of subgroups.

Estimated marginal means for each study group and estimated mean differences between study groups in each subgroup with 95% confidence interval (CI) were reported. Interaction effects were explored without multiplicity control as these were done under an exploratory framework.

© 2026 Karsten MM et al. *JAMA Oncology*.

Missing data and multiple imputation

Reasons for missing PRO data were documented within predefined study discontinuation categories and are presented in Figure 1. In the intervention group, discontinuations included death (n=49), loss to follow-up (n=66), and formal discontinuation (n=16), which comprised consent withdrawal, lack of interest, change of clinic, questionnaire burden, or being too ill to continue. In the control group, reasons included death (n=55), loss to follow-up (n=123), and formal discontinuation (n=7).

Formal discontinuation comprised the following reasons:

- Intervention group (n = 16): consent withdrawal (n = 5), lack of interest (n = 5), change of clinic (n = 2), questionnaire burden (n = 3), and being too ill to continue (n = 1).
- Control group (n = 7): consent withdrawal (n = 1), lack of interest (n = 3), change of clinic (n = 1), and being too ill to continue (n = 2).

Missing baseline characteristics were assumed to be missing at random and were imputed. For missing PRO data (e.g., fatigue T-score), the missing data mechanism was assumed to depend on the reasons for the missing data.[1] Missing PRO data due to reasons such as overload from the PRO survey, lack of interest, or loss to follow-up were assumed to be missing at random, whereas data from patients who discontinued due to feeling too ill or who died early were assumed to be missing not at random. eTable 3 presents a comparison of baseline characteristics between monotone missing data, non-monotone missing data, and fully observed PRO data. No differences were observed in general baseline characteristics unrelated to health between patients with completed the study and those who missing data (e.g., age, histology, education). However, when considering ECOG at baseline and EORTC T-scores, it was evident that patients who died early had worse EORTC T-scores at baseline. In addition, we compared baseline characteristics of patients with missing 6-month PRO data between the study group (eTable 4). Baseline fatigue T-scores among these patients were similar (control: 60.5 (SD 9.1); intervention 61.1 (SD 9.3); P value 0.70). Although missing data and loss to follow-up were more frequent in the control group (143 vs 62 patients), demographic, clinical, and baseline PRO characteristics were largely comparable between groups. Some differences were observed (e.g., self-reported ECOG, education, diarrhea), but no consistent pattern suggesting systematic baseline imbalance was identified. These findings support the plausibility of the missing at random assumption.

Missing data were addressed using multiple imputation (MI) using chained equations (MICE), under the assumption that the data were missing at random for patients who were lost to follow-up or dropped out, but had not died. Imputations were performed separately within each randomized group, with 30 imputed datasets generated (m = 30). MICE was performed based on predictive mean matching for continuous variables, ordinal logistic regression models for ordinal variables, and binary logistic regression models for binary variables. All potential confounding factors (as listed in the confounding factors above) were included in the imputation model, along with additional factors such as study centers and all PRO outcomes. Please note that patients who were not fully observed for a long-term outcome, particularly regarding late recruitment, were not considered as missing data, and no imputation was applied.

To account for fatigue T-scores in patients who died before six months, the worst possible value (the highest score) was assigned to the fatigue T-scores within each study group, but this was applied only in the primary outcome analysis.

In the longitudinal analysis of the fatigue T-score, MI was applied to the missing fatigue scores and baseline characteristics data, assuming the data were missing at random (e.g., due to incomplete questionnaires, missed visits, or early withdrawal). However, MI was not applied to patients who had died or were severely ill beyond that time point, as the missing data were considered not at random. Imputing data after death could misrepresent patient outcomes, but observed data collected before death were included in the analysis.[4] Sensitivity analyses were performed to assess the robustness of the primary analysis under various assumptions of missing not at random, including a reference-based analysis, tipping point analysis (TPA), randomized-arm MI with missing-at-random–based, and unimputed observed data (available case analysis). Detail of the sensitivity analyses were provided in a published SAP.[1]

For additional sensitivity analyses of the longitudinal analysis of the EORTC T-score, we applied the complete-case analysis and a post-hoc inverse probability of censoring weighting (IPCW) analysis. For IPCW, weights were derived from a logistic regression model predicting response at each visit, and cumulative weights were applied up to each patient's dropout time. These analyses were conducted to assess the robustness of results to differential attrition.

Alert frequency and alert rate

Alert frequency per patient was defined as the total number of alerts generated during the individual follow-up period. The median and interquartile range were calculated across all intervention patients. The alert rate per questionnaire was estimated using a Poisson model with the number of submitted follow-up questionnaires as the exposure variable.

© 2026 Karsten MM et al. *JAMA Oncology*.

Time-to-event analyses

Time to first change in systemic therapy and OS were assessed using Kaplan–Meier analysis and Cox proportional hazards models with shared frailty terms for center. Time to first change in systemic therapy was defined as initiation of a new systemic regimen, as documented in medical records at each center. Overall survival was defined as time from randomization to death from any cause, based on site-reported follow-up. Censoring was considered at either the last follow-up or study completion date (February 15, 2024).

5. Hospitalizations and emergency room visits: Subpopulation analysis using claims data

Data source

Hospitalization and emergency room visit data were derived from claims data provided by three participating health insurance companies: BARMER, DAK Gesundheit, and mkk – meine krankenkasse, formerly BKK·VBU. The analysis was restricted to participating patients insured by these three health insurance companies (n = 235), who were treated at 38 of 52 participating breast cancer centers (73.1%). This claims-data subpopulation represented 25.9% of the full PRO B Trial population (235/909), with similar proportions in the intervention and control groups (intervention, 118/456 [25.9%]; control, 117/453 [25.8%]).

Statistical analysis

Hospitalizations and emergency room visits were counted from randomization to 6 or 12 months. Observation time was defined as the time from randomization to the respective time point, with censoring at last follow-up, study completion date (February 15, 2024), or 6 or 12 months after randomization, whichever occurred first. Incidence rates (IRs) were calculated as the number of events per 100 person-months for each group. Incidence rate ratios (IRRs) and 95% CIs were estimated using multilevel mixed-effects negative binomial regression models with study center included as a random intercept. Negative binomial regression was used instead of Poisson regression to account for overdispersion in the count data, as the variance in hospitalization and emergency room visit counts exceeded the mean. Models were adjusted for age, type of metastasis, ECOG performance status at baseline, subtype, metastatic presentation, and time since metastatic diagnosis. Claims-based outcome data were complete for the claims-data subpopulation; missing covariate data were handled using complete case analysis because the number of missing cases was small (2/235 cases; 0.9%). All analyses were conducted using Stata MP/18 (StataCorp, 2023, College Station, Texas, USA).

© 2026 Karsten MM et al. *JAMA Oncology*.

eTable 1. Items included in the EORTC CAT Core short forms

EORTC CAT Core short forms	Intervention group				Control group
	Week 1	Week 2	Week 3	Week 4	
**Quality of Life (QL)[1]**					
QL1	X	X	X	X	X
QL2	X	X	X	X	X
**Physical Functioning (PF)[2]**					
PF15	X	X	X	X	X
PF28	-	X	-	-	X
PF24	X	X	X	X	X
PF23	-	X	-	-	X
PF19	X	X	X	X	X
PF31	-	X	-	-	X
PF13	X	X	X	X	X
PF5	X	X	X	X	X
**Role Functioning (RF)[2]**					
RF8	X	X	X	X	X
RF1	-	X	X	X	X
RF4	X	-	-	-	X
RF2	X	-	-	-	X
RF9	X	-	-	-	X
RF10	X	X	X	X	X
RF5	X	-	-	-	X
RF3	X	-	-	-	X
RF7	X	X	X	X	X
**Emotional Functioning (EF)[2]**					
EF1	X	X	X	X	X
EF2	-	X	-	-	X
EF8	-	X	-	-	X
EF16	X	X	X	X	X
EF10	-	X	-	-	X
EF17	X	X	X	X	X
EF19	X	X	X	X	X
EF20	-	X	-	-	X
**Cognitive Functioning (CF)[2]**					
CF11	X	X	X	X	X
CF14	-	-	X	-	X
CF34	X	X	X	X	X
CF26	-	-	X	-	X
CF13	X	X	X	X	X
CF29	-	-	X	-	X
CF18	X	X	X	X	X
CF27	-	-	X	-	X
**Social Functioning (SF)[2]**					
SF11	X	X	X	X	X
SF1	-	-	-	X	X
SF10	X	X	X	X	X
SF8	X	X	X	X	X
SF12	-	-	-	X	X
SF3	X	X	X	X	X
SF5	-	-	-	X	X
SF9	-	-	-	X	X
**Fatigue (FA)[2]**					
FA17	X	X	X	X	X
FA24	X	X	X	X	X
FA7	X	X	X	X	X
FA25	X	X	X	X	X
FA16	X	X	X	X	X

© 2026 Karsten MM et al. *JAMA Oncology*.

EORTC CAT Core short forms	Intervention group				Control group
	Week 1	Week 2	Week 3	Week 4	
FA22	X	X	X	X	X
FA27	X	X	X	X	X
FA31	X	X	X	X	X
**Nausea and Vomiting (NV)**[2]					
NV1	X	X	X	X	X
NV2	X	X	X	X	X
**Pain (PA)**[2]					
PA16	X	X	X	X	X
PA14	X	X	X	X	X
PA2	-	-	-	X	X
PA7	-	-	-	X	X
PA9	-	-	-	X	X
PA10	-	-	-	X	X
PA8	-	-	-	X	X
PA12	-	-	-	X	X
**Dyspnea (DY)**[2]					
DY21	X	X	X	X	X
DY25	X	X	X	X	X
**Sleep quality (SL)**[2]					
SL5	X	X	X	X	X
SL7	X	X	X	X	X
**Appetite Loss (AP)**[2]					
AP4	X	X	X	X	X
AP2	X	X	X	X	X
**Constipation (CO)**[2]					
CO9	X	X	X	X	X
CO2	X	X	X	X	X
**Diarrhea (DI)**[2]					
DI12	X	X	X	X	X
DI2	X	X	X	X	X
**Financial Difficulties (FI)**[2]					
FI1	X	X	X	X	X
FI6	X	X	X	X	X
**Total items**	**51**	**54**	**51**	**57**	**57**

Response options: ¹ Scale from 1 (very poor) to 7 (excellent); ² Scale: 1 = Not at all, 2 = A little, 3 = Quite a bit, 4 = Very much.

© 2026 Karsten MM et al. *JAMA Oncology*.

eTable 2. Deterioration cut-points of the EORTC T-score and health transition item

Domain of the EORTC	Abbreviation	Deterioration in current T-score compared with last week or last month
Global health status/QoL	QL	≤ -5
Physical functioning	PF	≤ -5
Role functioning	RF	≤ -5
Emotional functioning	EF	≤ -5
Cognitive functioning	CF	≤ -5
Social functioning	SF	≤ -5
Fatigue	FA	≥ + 10
Pain	PA	≥ + 10
Dyspnea	DY	≥ + 10
Insomnia	SL	≥ + 10
Financial impact	FI	≥ + 20
**Gastrointestinal symptoms [a]**		**2 of 4 subdomains**
Nausea/vomiting	NV	≥ + 10
Appetite loss	AP	≥ + 10
Constipation	CO	≥ + 10
Diarrhea	DI	≥ + 10
**Health transition item[b]**		
"*How would you describe your current health compared to last week?*"	HT	The response options<br>- much better<br>- a little better<br>- about the same as last week<br>- a little worse<br>- much worse.

[a] The gastrointestinal symptom domain of the EORTC consists of four subdomains. For assessing deterioration, it is considered as a single domain, with deterioration defined as an increase in the score, compared to the previous week or month, in at least two of the four subdomains.
[b] A single health transition item was evaluated alongside the EORTC CAT Core.

© 2026 Karsten MM et al. *JAMA Oncology*.

eTable 3. Exploring patients' characteristics at baseline by type of missing data and fully observed data

	Monotone missing data		Non-monotone missing data	Observed
	Dead (n = 54)	Loss F/U (n = 94)	(n = 111)	(n = 650)
Age at randomization (years) – Mean (SD)	51.5 (10.4)	47.5 (10.6)	50.0 (10.9)	51.4 (11.0)
Time since metastatic diagnosis (months) – Median (IQR)	14 (4-41)	16 (3-47)	11 (3-37)	18 (5-45)
Subtype				
HR+/HER- or HR+/HER+	36 (66.7%)	73 (77.7%)	84 (75.7%)	533 (82.0%)
HR-/HER+ or HR-/HER-	18 (33.3%)	21 (22.3%)	27 (24.3%)	117 (18.0%)
Site of metastases				
Brain or multiple metastases	37 (68.5%)	51 (54.3%)	62 (55.9%)	353 (54.3%)
Bone, lymph nodes, or skin	10 (18.5%)	29 (30.9%)	30 (27.0%)	203 (31.2%)
Visceral (only one organ)	7 (13.0%)	14 (14.9%)	19 (17.1%)	94 (14.5%)
Education				
Low	5 (10.2%)	11 (12.6%)	9 (8.4%)	60 (9.3%)
Medium	26 (53.1%)	45 (51.7%)	56 (52.3%)	359 (55.5%)
High	18 (36.7%)	31 (35.6%)	42 (39.3%)	228 (35.2%)
Employment status				
No	27 (55.1%)	42 (48.3%)	51 (47.7%)	291 (45.0%)
Yes	22 (44.9%)	45 (51.7%)	56 (52.3%)	356 (55.0%)
Migration background				
No	45 (91.8%)	68 (78.2%)	95 (88.8%)	608 (94.0%)
Yes	4 (8.2%)	19 (21.8%)	12 (11.2%)	39 (6.0%)
Family status				
Single	7 (14.3%)	7 (8.0%)	15 (14.0%)	89 (13.8%)
Married	32 (65.3%)	58 (66.7%)	70 (65.4%)	421 (65.1%)
Separated/divorced/widowed	10 (20.4%)	22 (25.3%)	22 (20.6%)	137 (21.2%)
Having children < 14 years	7 (14.3%)	21 (24.1%)	15 (14.0%)	105 (16.2%)
Family income (in Euro)				
<2500	20 (45.5%)	26 (38.2%)	34 (39.1%)	185 (33.6%)
2500-4999	15 (34.1%)	34 (50.0%)	38 (43.7%)	256 (46.5%)
≥5000	9 (20.5%)	8 (11.8%)	15 (17.2%)	109 (19.8%)
Having depression	0 (0.0%)	11 (12.6%)	14 (13.2%)	69 (10.6%)
NCI comorbidity index	0.1 (0.3)	0.1 (0.3)	0.1 (0.2)	0.1 (0.2)
Therapy at baseline				
Endocrine	14 (25.9%)	60 (63.8%)	54 (48.6%)	408 (62.8%)
Chemotherapy	35 (64.8%)	45 (47.9%)	54 (48.6%)	308 (47.4%)
Anti-Her2	5 (9.3%)	30 (31.9%)	28 (25.2%)	146 (22.5%)
Another immunotherapy	2 (3.7%)	5 (5.3%)	6 (5.4%)	19 (2.9%)
Targeted therapy	13 (24.1%)	19 (20.2%)	41 (36.9%)	244 (37.5%)
Other	9 (16.7%)	8 (8.5%)	28 (25.2%)	78 (12.0%)
Period of enrollment				
Before 01.09.2022	38 (70.4%)	45 (47.9%)	73 (65.8%)	441 (67.8%)
After 01.09.2022	16 (29.6%)	49 (52.1%)	38 (34.2%)	209 (32.2%)
Self-reported ECOG status				
ECOG 0	2 (4.1%)	22 (25.3%)	13 (12.3%)	141 (21.8%)
ECOG 1	25 (51.0%)	44 (50.6%)	58 (54.7%)	369 (56.9%)
ECOG 2	16 (32.7%)	14 (16.1%)	24 (22.6%)	115 (17.7%)
ECOG 3	6 (12.2%)	6 (6.9%)	11 (10.4%)	22 (3.4%)
ECOG 4	0 (0.0%)	1 (1.1%)	0 (0.0%)	1 (0.2%)
**EORTC T-score at baseline**				
Global health status/QoL	34.1 (3.3)	36.1 (4.8)	36.1 (4.5)	36.0 (3.7)
Physical functioning	35.9 (9.3)	40.9 (10.4)	40.4 (9.0)	41.8 (9.2)
Role functioning	34.9 (9.5)	39.5 (11.1)	38.4 (10.2)	41.1 (10.0)
Emotional functioning	38.6 (8.4)	39.5 (8.1)	39.7 (8.8)	42.1 (8.8)
Cognitive functioning	42.7 (10.0)	42.1 (10.5)	41.6 (11.0)	44.3 (9.8)
Social functioning	37.6 (7.8)	41.3 (9.0)	39.0 (8.4)	42.0 (8.9)

© 2026 Karsten MM et al. *JAMA Oncology*.

Fatigue	63.7 (8.3)	60.4 (9.8)	61.3 (8.8)	57.8 (9.3)
Nausea and vomiting	63.5 (11.2)	61.5 (11.5)	60.6 (11.2)	58.5 (10.3)
Pain	56.9 (10.1)	55.6 (10.1)	56.8 (10.4)	54.2 (9.8)
Dyspnea	64.1 (7.6)	59.3 (10.7)	61.0 (10.0)	57.4 (10.7)
Insomnia	57.6 (7.5)	58.8 (8.7)	59.6 (9.1)	56.9 (8.5)
Appetite loss	62.2 (13.3)	57.7 (11.2)	56.9 (11.2)	53.7 (11.3)
Constipation	57.7 (11.1)	54.2 (11.5)	54.7 (11.4)	52.2 (10.6)
Diarrhea	55.7 (11.3)	55.6 (11.3)	56.7 (12.7)	55.1 (11.7)
Financial difficulties	55.1 (11.7)	55.9 (12.2)	57.5 (11.1)	53.0 (10.0)

ECOG, Eastern Cooperative Oncology Group; NCI, National Cancer Institute; QoL, Quality of Life

eTable 4. Comparison of patient characteristics at baseline in patients who had missing data or loss to follow-up between the study group

	Control group (n = 143)	Intervention group (n = 62)
Age at randomization (years) – Mean (SD)	49 (11)	48 (12)
Time since metastatic diagnosis (months) – Median (IQR)	11 (3-41)	16 (3-43)
Subtype		
HR+/HER- or HR+/HER+	107 (74.8%)	50 (80.6%)
HR-/HER+ or HR-/HER-	36 (25.2%)	12 (19.4%)
Site of metastases		
Brain or multiple metastases	73 (51.0%)	40 (64.5%)
Bone, lymph nodes, or skin	43 (30.1%)	16 (25.8%)
Visceral (only one organ)	27 (18.9%)	6 (9.7%)
Education		
Low	10 (7.2%)	10 (17.9%)
Medium	79 (57.2%)	22 (39.3%)
High	49 (35.5%)	24 (42.9%)
Currently employed	74 (53.6%)	27 (48.2%)
Family status		
Single	14 (10.1%)	8 (14.3%)
Married	97 (70.3%)	31 (55.4%)
Separated/divorced/widowed	27 (19.6%)	17 (30.4%)
Having children < 14 years	29 (21.0%)	7 (12.5%)
Family income (in Euro)		
<2500	42 (37.2%)	18 (42.9%)
2500-4999	51 (45.1%)	21 (50.0%)
≥5000	20 (17.7%)	3 (7.1%)
Having depression	17 (12.4%)	8 (14.3%)
Therapy at baseline		
Endocrine	82 (57.3%)	32 (51.6%)
Chemotherapy	70 (49.0%)	29 (46.8%)
Anti-Her2	39 (27.3%)	19 (30.6%)
Another immunotherapy	8 (5.6%)	3 (4.8%)
Targeted therapy	39 (27.3%)	21 (33.9%)
Other	26 (18.2%)	10 (16.1%)
Period of enrollment		
Before 01.09.2022	83 (58.0%)	35 (56.5%)
After 01.09.2022	60 (42.0%)	27 (43.5%)
Self-reported ECOG status		
ECOG 0	29 (21.2%)	6 (10.7%)
ECOG 1	78 (56.9%)	24 (42.9%)
ECOG 2	20 (14.6%)	18 (32.1%)
ECOG 3	9 (6.6%)	8 (14.3%)
ECOG 4	1 (0.7%)	0 (0.0%)
**EORTC T-score at baseline**		
Global health status/QoL	36.2 (4.8)	35.8 (4.3)
Physical functioning	41.4 (9.7)	38.7 (9.3)
Role functioning	39.5 (10.8)	37.4 (9.9)
Emotional functioning	39.9 (8.7)	38.8 (7.9)
Cognitive functioning	41.8 (10.9)	41.8 (10.4)
Social functioning	39.9 (8.6)	40.3 (9.2)
Fatigue	61.1 (9.3)	60.5 (9.1)
Nausea and vomiting	62.0 (11.6)	58.6 (10.5)
Pain	56.1 (10.5)	56.7 (9.6)
Dyspnea	60.8 (10.1)	58.8 (10.9)
Insomnia	60.0 (8.8)	57.3 (9.1)
Appetite loss	57.3 (11.6)	57.2 (10.4)
Constipation	54.0 (11.1)	55.8 (12.2)
Diarrhea	57.4 (12.7)	53.2 (9.7)
Financial difficulties	56.9 (11.3)	56.4 (12.5)

© 2026 Karsten MM et al. *JAMA Oncology*.

eTable 5. Baseline patient characteristics in observed data and after multiple imputation (imputed data set, *m* = 30)

	Observed data			Multiple imputation (*m* = 30)		
	**Total (n=909)**	**Control (n=453)**	**Intervention (n=456)**	**Total (n=909)**	**Control (n=453)**	**Intervention (n=456)**
**Number of missing (%)**	**n=19 (2.09%)**	**n=9 (2.00%)**	**n=10 (2.19%)**			
Education						
Low	85 (9.6%)	42 (9.5%)	43 (9.6%)	9.8%	9.7%	9.8%
Medium	486 (54.6%)	257 (57.9%)	229 (51.3%)	54.5%	57.5%	51.4%
High	319 (35.8%)	145 (32.7%)	174 (39.0%)	35.7%	32.7%	38.7%
Employment status						
No	411 (46.2%)	207 (46.6%)	204 (45.7%)	46.3%	46.7%	46.0%
Yes	479 (53.8%)	237 (53.4%)	242 (54.3%)	53.7%	53.3%	54.0%
Migration background						
No	816 (91.7%)	402 (90.5%)	414 (92.8%)	91.3%	90.2%	92.5%
Yes	74 (8.3%)	42 (9.5%)	32 (7.2%)	8.7%	9.8%	7.5%
Family status						
Single	118 (13.3%)	49 (11.0%)	69 (15.5%)	13.4%	11.1%	15.7%
Married	581 (65.3%)	295 (66.4%)	286 (64.1%)	65.1%	66.2%	63.9%
Separated/divorced/ widowed	191 (21.5%)	100 (22.5%)	91 (20.4%)	21.5%	22.6%	20.4%
Having children < 14 years						
no	742 (83.4%)	369 (83.1%)	373 (83.6%)	83.3%	82.9%	83.8%
yes	148 (16.6%)	75 (16.9%)	73 (16.4%)	16.7%	17.1%	16.2%
Family income (in Euros)						
< 2500	265 (35.4%)	129 (34.6%)	136 (36.2%)	37.3%	35.9%	38.8%
2500-4999	343 (45.8%)	179 (48.0%)	164 (43.6%)	44.2%	46.6%	41.8%
≥ 5000	141 (18.8%)	65 (17.4%)	76 (20.2%)	18.4%	17.5%	19.4%
Having depression						
No	796 (89.4%)	404 (91.2%)	392 (87.7%)	89.3%	90.9%	87.7%
Yes	94 (10.6%)	39 (8.8%)	55 (12.3%)	10.7%	9.1%	12.3%
Self-reported ECOG status						
ECOG 0	178 (20.0%)	82 (18.5%)	96 (21.5%)	19.8%	18.3%	21.3%
ECOG 1	496 (55.7%)	256 (57.8%)	240 (53.7%)	55.6%	57.8%	53.5%
ECOG 2	169 (19.0%)	83 (18.7%)	86 (19.2%)	19.1%	18.9%	19.4%
ECOG 3	45 (5.1%)	20 (4.5%)	25 (5.6%)	5.2%	4.6%	5.7%
ECOG 4	2 (0.2%)	2 (0.5%)	0 (0.0%)	0.2%	0.4%	0.0%
**Number of missing (%)**	**n=54 (5.94%)**	**n=28 (6.18%)**	**n=26 (5.70%)**			
Health transition item at baseline						
Much better	56 (6.5%)	28 (6.6%)	28 (6.5%)	6.6%	6.8%	6.4%
A little better	187 (21.9%)	87 (20.5%)	100 (23.3%)	21.8%	20.6%	23.0%
About the same	517 (60.5%)	261 (61.4%)	256 (59.5%)	60.5%	61.2%	59.7%
A little worse	90 (10.5%)	45 (10.6%)	45 (10.5%)	10.5%	10.5%	10.6%
Much worse	5 (0.6%)	4 (0.9%)	1 (0.2%)	0.6%	0.9%	0.2%
EORTC T-score – Mean (SD)						
Global health status/QoL	35.9 (3.9)	36.0 (4.3)	35.9 (3.6)	35.9 (3.9)	36.0 (4.2)	35.9 (3.5)
Physical functioning	41.2 (9.4)	41.0 (9.1)	41.4 (9.7)	41.1 (9.4)	41.0 (9.0)	41.2 (9.7)
Role functioning	40.3 (10.2)	39.7 (9.9)	40.8 (10.5)	40.2 (10.1)	39.7 (9.8)	40.6 (10.4)
Emotional functioning	41.3 (8.8)	40.4 (8.4)	42.3 (9.1)	41.2 (8.8)	40.2 (8.4)	42.2 (9.1)
Cognitive functioning	43.7 (10.0)	42.5 (9.9)	44.8 (10.0)	43.5 (10.0)	42.4 (9.9)	44.6 (10.0)
Social functioning	41.3 (8.9)	40.2 (8.0)	42.4 (9.5)	41.2 (8.8)	40.2 (8.0)	42.3 (9.5)
Fatigue	58.8 (9.4)	60.2 (9.0)	57.5 (9.5)	58.9 (9.3)	60.2 (9.0)	57.6 (9.5)

© 2026 Karsten MM et al. *JAMA Oncology*.

Nausea and vomiting	59.3 (10.6)	61.3 (11.8)	57.4 (9.0)	59.3 (10.7)	61.3 (11.8)	57.4 (9.1)
Pain	54.8 (10.0)	55.7 (10.2)	53.9 (9.6)	54.9 (10.0)	55.8 (10.2)	54.0 (9.6)
Dyspnea	58.4 (10.6)	60.0 (10.1)	56.7 (10.8)	58.4 (10.6)	59.9 (10.1)	56.9 (10.8)
Insomnia	57.4 (8.6)	58.7 (8.6)	56.2 (8.5)	57.5 (8.6)	58.8 (8.6)	56.2 (8.5)
Appetite loss	55.0 (11.6)	56.9 (12.1)	53.1 (10.9)	55.0 (11.6)	56.9 (12.1)	53.1 (10.9)
Constipation	53.0 (10.9)	54.3 (11.3)	51.7 (10.4)	53.0 (10.9)	54.2 (11.2)	51.8 (10.4)
Diarrhea	55.3 (11.7)	57.3 (12.2)	53.4 (10.9)	55.4 (11.8)	57.3 (12.2)	53.4 (11.0)
Financial difficulties	54.0 (10.5)	55.9 (10.9)	52.1 (9.8)	54.1 (10.6)	56.0 (10.9)	52.1 (9.9)

ECOG, Eastern Cooperative Oncology Group; EORTC, European Organization for Research and Treatment of Cancer; NCI, National Cancer Institute; SD, standard deviation; QoL, Quality of Life

© 2026 Karsten MM et al. *JAMA Oncology*.

eTable 6. Primary outcome: Fatigue T-score at 6 months

Primary outcome	Marginal estimated mean (95% CI) Control (n = 453)	Intervention (n = 456)	Mean difference between Groups (95% CI)	*P* value
**Main analysis** (n_{obs} = 909)				
Randomized-arm MI + worst-case scenario[a]	59.9 (59.0 – 60.8)	54.5 (53.7 – 55.4)	-5.4 (-6.6 to -4.1)	< .001
**Sensitivity analyses**				
Complete case (n_{obs} = 541)	(n = 236)	(n = 305)		< .001
	59.2 (58.1 – 60.3)	54.5 (53.6 – 55.5)	-4.7 (-6.1 to -3.2)	
Randomized-arm MI (MAR-based) (n_{obs} = 909)	58.8 (57.8 – 59.8)	53.6 (52.7 – 54.4)	-5.2 (-6.4 to -4.0)	< .001
Reference-based MI[§] (n_{obs} = 909)	58.4 (57.5 – 59.3)	55.4 (54.5 – 56.3)	-3.0 (-4.2 to -1.8)	< .001
Tipping point analysis (TPA)[b] (n_{obs} = 909)				
Delta-adjusting: δ = 4	58.8 (57.8 – 59.8)	54.3 (53.4 – 55.2)	-4.5 (-5.7 to -3.2)	< .001
Delta-adjusting: δ = 8	58.8 (57.8 – 59.8)	55.1 (54.2 – 56.0)	-3.7 (-4.9 to -2.4)	< .001
Delta-adjusting: δ = 12	58.8 (57.8 – 59.8)	55.8 (54.9 – 56.7)	-2.9 (-4.2 to -1.6)	< .001
Delta-adjusting: δ = 18	58.8 (57.8 – 59.8)	57.0 (56.0 – 57.9)	-1.7 (-3.1 to -0.3)	.02
Delta-adjusting: δ = 20	58.8 (57.8 – 59.8)	57.4 (56.4 – 58.3)	-1.3 (-2.8 to 0.10)	.07
Change from baseline (n_{obs} = 909)	0.6 (-0.4 to 1.6)	-3.8 (-4.8 to -2.9)	-2.6 (-3.7 to -1.6)	< .001

[a] Missing data were addressed using multiple imputation using chained equations (MICE), assuming the data were missing at random, with separate imputation by randomized-arm MI (*m* = 30) and the worst-case scenario was applied only to deceased patients with the highest fatigue score within each study group.
[§]Reference-based MI: A multiple imputation method that imputes missing values by assuming that the behavior of intervention patients jumps to that of the control group.
[b] TPA starting from 4 to 20, a delta value that adjusts the imputed values for observations in the intervention group who died before 6 months.
Linear mixed model with random intercept by study center was performed to estimate the mean and 95% CI of the fatigue score. The models were adjusted for fatigue score at baseline, age, education, ECOG, employment status, family income, family status, having children < 14 years, migration, depression, NCI index, histologic findings, type of metastasis, treatment at baseline, and enrollment period (before vs after September 1, 2022).

© 2026 Karsten MM et al. *JAMA Oncology*.

eTable 7. Subgroup analyses of fatigue T-score at 6 months post-randomization

Subgroup analysis	Control group (n = 453)		Intervention group (n = 456)		Mean difference between groups (95% CI)	*P* value for interaction test
	Number [a]	Estimated marginal mean (95% CI)	Number [a]	Estimated marginal mean (95% CI)		
**Site of metastases**						.39
Brain or multiple metastases	250	59.7 (58.4, 60.9)	253	54.5 (53.4, 55.7)	-5.1 (-6.8, -3.5)	
Bone, lymph nodes, or skin	135	59.4 (57.7, 61.1)	137	54.6 (53.1, 56.2)	-4.8 (-7.1, -2.5)	
Visceral (only one organ)	68	61.8 (59.2, 64.4)	66	54.4 (52.2, 56.6)	-7.5 (-10.8, -4.1)	
**Clinical subtype**						.55
HR+/HER2+	73	59.1 (56.4, 61.8)	68	53.9 (51.3, 56.5)	-5.1 (-8.2, -2.1)	
HR+/HER2-	269	60.0 (58.7, 61.3)	287	54.6 (53.4, 55.7)	-5.5 (-7.0, -3.9)	
HR–/HER2+	25	61.5 (57.2, 65.8)	31	52.9 (49.1, 56.7)	-8.5 (-13.6, -3.5)	
TNBC	49	61.6 (58.5, 64.6)	38	54.3 (50.9, 57.7)	-7.3 (-11.2, -3.3)	
**Metastatic presentation**						.82
De novo	181	59.5 (58.1, 60.9)	197	54.3 (53.1, 55.6)	-5.2 (-7.0, -3.3)	
Recurrent	270	60.2 (59.0, 61.3)	256	54.7 (53.6, 55.8)	-5.5 (-7.0, -3.9)	
**Family status**						.19
Single	49	59.6 (56.9, 62.2)	69	54.0 (51.6, 56.4)	-5.6 (-9.2, -2.0)	
Married	295	59.6 (58.4, 60.7)	286	54.9 (53.9, 56.0)	-4.7 (-6.2, -3.1)	
Separated/Divorced/Widowed	100	61.2 (59.3, 63.0)	91	53.7 (51.7, 55.6)	-7.5 (-10.1, -4.9)	
**Family income (in Euros)**						.78
<2500	129	60.9 (59.2, 62.5)	136	54.9 (53.5, 56.3)	-5.9 (-8.0, -3.8)	
2500-4999	179	59.3 (57.9, 60.6)	164	54.4 (53.0, 55.8)	-4.9 (-6.8, -3.0)	
≥5000	65	59.6 (57.1, 62.1)	76	54.1 (52.2, 56.1)	-5.4 (-8.4, -2.3)	
**Having depression**						.86
No	404	60.0 (59.1, 61.0)	392	54.6 (53.7, 55.5)	-5.4 (-6.7, -4.1)	
Yes	39	58.8 (55.6, 62.0)	55	53.8 (51.4, 56.2)	-5.0 (-9.0, -1.0)	
**Period of enrollment** [b]						.38
Before 01.09.2022	189	59.6 (58.2, 61.1)	199	54.9 (53.6, 56.2)	-4.7 (-6.6, -2.8)	
After 01.09.2022	264	60.1 (58.9, 61.3)	257	54.3 (53.1, 55.4)	-5.8 (-7.5, -4.2)	
**Age at randomization (years)**						.39
<50	197	60.4 (58.8, 61.9)	211	54.4 (53.1, 55.6)	-6.0 (-7.9, -4.1)	
≥50	256	59.6 (58.3, 60.8)	245	54.7 (53.5, 55.9)	-4.9 (-6.5, -3.2)	
**BMI (kg/m^2)**						.47
<30	238	60.3 (59.1, 61.5)	259	54.5 (53.4, 55.7)	-5.8 (-7.4, -4.2)	
≥30	215	59.5 (58.1, 60.8)	197	54.5 (53.3, 55.8)	-4.9 (-6.7, -3.1)	

© 2026 Karsten MM et al. *JAMA Oncology*.

Subgroup analysis	Control group (n = 453)		Intervention group (n = 456)		Mean difference between groups (95% CI)	*P* value for interaction test
	Number [a]	Estimated marginal mean (95% CI)	Number [a]	Estimated marginal mean (95% CI)		
**Endocrine therapy**						**.65**
No	191	61.7 (60.2, 63.2)	182	55.9 (54.5, 57.4)	-5.7 (-7.6, -3.8)	
Yes	262	58.7 (57.4, 60.0)	274	53.5 (52.4, 54.7)	-5.1 (-6.8, -3.5)	
**Chemotherapy**						**.71**
No	234	59.8 (58.5, 61.2)	233	54.7 (53.5, 55.9)	-5.2 (-7.0, -3.3)	
Yes	219	60.0 (58.7, 61.3)	223	54.4 (53.1, 55.6)	-5.6 (-7.4, -3.9)	
**Anti-Her2 therapy**						**.67**
No	345	60.6 (59.5, 61.7)	355	55.1 (54.1, 56.0)	-5.5 (-6.9, -4.1)	
Yes	108	57.6 (55.7, 59.5)	101	52.7 (50.8, 54.6)	-4.9 (-7.5, -2.3)	
**Another immunotherapy**						**.21**
No	437	60.0 (59.0, 60.9)	440	54.7 (53.9, 55.6)	-5.2 (-6.5, -4.0)	
Yes	16	58.7 (53.4, 64.0)	16	49.0 (43.9, 54.1)	-9.7 (-16.5, -2.8)	
**Targeted therapy**						**.77**
No	303	60.2 (59.0, 61.3)	289	54.9 (53.9, 56.0)	-5.2 (-6.8, -3.7)	
Yes	150	59.4 (57.7, 61.1)	167	53.8 (52.4, 55.2)	-5.6 (-7.8, -3.5)	
**Self-reported ECOG**						**.11**
ECOG 0	87	59.4 (57.3, 61.5)	101	52.3 (50.3, 54.2)	-7.1 (-9.9, -4.4)	
ECOG 1	259	59.9 (58.8, 61.1)	242	55.3 (54.1, 56.4)	-4.7 (-6.3, -3.0)	
ECOG 2	84	59.6 (57.5, 61.7)	86	55.3 (53.4, 57.3)	-4.3 (-7.0, -1.6)	
ECOG ≥3	22	63.2 (59.0, 67.5)	25	53.1 (49.2, 57.1)	-10.1 (-15.6, -4.6)	
**Time since metastatic diagnosis**						**.91**
<12 months	202	60.0 (58.6, 61.5)	180	54.4 (53.0, 55.7)	-5.7 (-7.5, -3.8)	
12 – 24 months	65	59.4 (57.2, 61.7)	81	54.5 (52.5, 56.5)	-4.9 (-7.9, -1.9)	
> 24 months	182	60.0 (58.5, 61.5)	191	54.6 (53.3, 55.9)	-5.4 (-7.3, -3.5)	
**Type of breast cancer centers**						**.84**
Outpatient/standard care	55	59.7 (57.2, 62.2)	56	54.4 (52.0, 56.7)	-5.3 (-8.7, -2.0)	
Specialized care	97	60.6 (58.6, 62.6)	99	54.5 (52.7, 56.3)	-6.1 (-8.7, -3.4)	
Tertiary care/university	301	59.7 (58.7, 60.8)	301	54.6 (53.5, 55.6)	-5.2 (-6.7, -3.6)	
**Enrollment period**						**.28**
May – December 2021	117	59.4 (57.5, 61.3)	117	54.7 (53.0, 56.4)	-4.7 (-7.2, -2.3)	
January – December 2022	224	59.9 (58.6, 61.2)	230	55.0 (53.8, 56.1)	-4.9 (-6.7, -3.1)	
January – June 2023	112	60.5 (58.6, 62.4)	109	53.5 (51.8, 55.2)	-7.0 (-9.5, -4.5)	

[a] Numbers were presented before multiple imputation was applied. [b] Enrollment period, corresponding to the modification of alert criteria.
Linear mixed model with random intercept by study center was performed to estimate the mean and 95% CI of the fatigue T-score and interaction terms between study group and each factor were explored. The models were adjusted for fatigue T-score at baseline, age, education, ECOG, employment status, family income, family status, having children < 14 years, migration, depression, NCI index, histologic findings, type of metastasis, metastatic presentation, treatment at baseline, time since metastatic diagnosis, and enrollment period.

© 2026 Karsten MM et al. *JAMA Oncology*.

eTable 8. The estimated marginal mean and 95% CI for each time point of the T-scores

EORTC T-scores	Intervention group (IG) – mean (95% CI)	Control group (CG) – mean (95% CI)	Mean difference between IG – CG (95% CI)	*P* value	A post-hoc sensitivity analysis: Mean difference between IG – CG (95% CI)	
					Complete-case analysis	IPCW
**Fatigue**						
Baseline	57.6 (SD 9.5)	60.2 (SD 9.0)	-2.6 (-3.7; -1.6)	< .001	-2.7 (-3.8; -1.5)	-2.9 (-4.9; -1.0)
3 months	53.6 (52.6 - 54.7)	58.8 (57.6 - 60.0)	-5.2 (-6.6; -3.8)	< .001	-4.7 (-6.1; -3.2)	-4.7 (-6.1; -3.3)
6 months	53.2 (52.2 - 54.3)	58.7 (57.5 - 60.9)	-5.4 (-6.9; -3.9)	< .001	-4.8 (-6.3; -3.3)	-5.1 (-6.6; -3.5)
9 months	52.3 (50.9 - 53.7)	59.2 (57.3 - 61.0)	-6.8 (-9.0; -4.7)	< .001	-6.1 (-7.9; -4.4)	-6.6 (-8.8; -4.4)
12 months	53.9 (52.0 - 55.7)	59.2 (54.8 - 63.5)	-5.2 (-9.0; -1.4)	.004	-5.5 (-7.5; -3.6)	-5.4 (-8.6; -2.1)
**Physical functioning**						
Baseline	41.2 (SD 9.7)	41.0 (SD 9.0)	0.1 (-0.9; 1.1)	.82	0.2 (-0.8; 1.2)	0.4 (-1.4; 2.2)
3 months	44.2 (43.1 - 45.3)	40.5 (39.4 - 41.6)	3.7 (2.4; 5.0)	< .001	3.5 (2.1; 4.9)	4.1 (2.9; 5.3)
6 months	44.6 (43.6 - 45.7)	40.6 (39.4 - 41.9)	4.0 (2.6; 5.4)	< .001	4.2 (2.7; 5.7)	3.4 (1.7; 5.1)
9 months	44.6 (43.3 - 45.9)	39.5 (37.9 - 41.1)	5.1 (3.2; 7.1)	< .001	4.9 (3.3; 6.6)	4.6 (2.7; 6.6)
12 months	43.6 (41.9 - 45.3)	41.8 (37.2 - 46.3)	1.8 (-2.5; 6.1)	.73	4.1 (2.1; 6.0)	3.5 (0.2; 6.8)
**Health-related quality of life**						
Baseline	35.9 (SD 3.5)	36.0 (SD 4.2)	-0.2 (-0.6; 0.3)	.44	-0.3 (-0.8; 0.1)	-0.7 (-1.7; 0.4)
3 months	36.2 (35.8 - 36.7)	35.7 (35.1 - 36.2)	0.6 (-0.03; 1.1)	.07	0.4 (-0.2; 1.0)	0.3 (-0.2; 0.9)
6 months	36.3 (35.8 - 36.8)	35.5 (34.9 - 36.1)	0.8 (0.02; 1.5)	.04	0.8 (0.1; 1.4)	1.1 (0.04; 2.1)
9 months	36.3 (35.7 - 36.8)	35.3 (34.3 - 36.4)	0.9 (-0.1; 1.9)	.09	1.1 (0.4; 1.8)	0.7 (-0.3; 1.7)
12 months	35.7 (34.9 - 36.5)	35.3 (33.8 - 36.7)	0.4 (-1.0; 1.8)	.90	0.6 (-0.2; 1.5)	0.5 (-0.7; 1.8)
**Emotional functioning**						
Baseline	42.2 (SD 9.0)	40.2 (SD 8.4)	2.1 (1.0; 3.2)	< .001	1.8 (0.7; 2.9)	1.5 (-0.4; 3.5)
3 months	46.9 (45.8 - 47.9)	42.1 (41.0 - 43.3)	4.7 (3.4; 6.1)	< .001	4.2 (3.1; 5.3)	4.7 (2.9; 6.6)
6 months	47.1 (46.0 - 48.2)	42.8 (41.5 - 44.1)	4.3 (2.8; 5.8)	< .001	4.0 (2.9; 5.2)	3.9 (2.4; 5.4)
9 months	48.4 (47.1 - 49.7)	41.8 (40.1 - 43.4)	6.6 (4.7; 8.5)	< .001	6.6 (5.3; 7.9)	6.6 (4.5; 8.7)
12 months	48.2 (46.4 - 50.0)	43.8 (39.5 - 48.1)	4.4 (0.2; 8.5)	.03	5.3 (3.8; 6.7)	5.0 (2.0; 8.0)

Higher T-scores indicate better functioning for functional scales and greater symptom burden for symptom scales.
The T-scores were imputed using multiple imputation (MI) based on the randomized group (m = 30), MI was not applied to patients who died or were severely ill after that time point.
Linear mixed models with random intercepts for study centers and patients were used. The model was adjusted for the T-scores at baseline, age, education, ECOG, employment status, family income, family status, having children < 14 years, migration, depression, NCI index, subtype, type of metastases, systemic treatment at baseline, and enrollment period (before vs after September 1, 2022).
The estimated marginal mean and 95% CI for each time point were adjusted for multiple comparisons using the Sidak method. Complete-case analyses included all available observations at each time point, with no imputation of missing data. Additionally, models incorporating inverse-probability-of-censoring weighting (IPCW) were performed. For IPCW, weights were estimated from a logistic regression model predicting response (yes/no) at each time point, and the inverse of these probabilities was applied to observed cases. Cumulative weights were calculated up to each patient's dropout time. SD, standard deviation.

© 2026 Karsten MM et al. *JAMA Oncology*.

eTable 9. Overall survival analysis

	Overall survival		Bivariate analysis			Multivariable analysis		
	6-month	12-month	Unadj. HR	95% CI	*P* value	Adj. HR	95% CI	*P* value
**Total study population**			**n = 909**			**n = 831**		
Intervention	94.7%	87.6%	0.82	(0.61, 1.12)	.21	0.72[a]	(0.52, 0.99)	.04
Control	92.0%	84.7%						
**Subgroup analyses**								
**Site of metastases**								
**Brain or multiple metastases**			**n = 503**			**n = 465**		
Intervention	92.1%	81.2%	0.94	(0.65, 1.34)	.72	0.79[b]	(0.54, 1.16)	.22
Control	91.4%	81.5%						
**Bone, lymph nodes, or skin**			**n = 272**			**n = 242**		
Intervention	98.5%	96.6%	0.60	(0.29, 1.24)	.17	0.42[b]	(0.17, 1.04)	.06
Control	93.3%	89.2%						
**Visceral (only one organ)**			**n = 134**			**n = 132**		
Intervention	96.9%	93.3%	0.57	(0.21, 1.56)	.27	0.37[b]	(0.12, 1.15)	.09
Control	91.9%	87.7%						
**Metastatic presentation (missing = 5)**								
**De novo**			**n = 378**			**n = 378**		
Intervention	93.6%	90.1%	0.97	(0.56, 1.70)	.92	1.00	(0.57, 1.77)	.99
Control	93.8%	91.2%						
**Recurrent**			**n = 526**			**n = 519**		
Intervention	95.1%	84.8%	0.79	(0.55, 1.14)	.22	0.77	(0.53, 1.12)	.18
Control	90.2%	80.8%						
**Subtype (missing = 69)**								
**HR+/HER2+**								
Intervention	95%	93%	NA			NA		
Control	100%	100%						
**HR+/HER2-**			**n = 556**			**n = 551**		
Intervention	95.3%	88.5%	0.68	(0.47, 0.99)	.04	0.69[c]	(0.47, 1.00)	.05
Control	90.4%	82.7%						
**HR-/HER2+**			**n = 56**			**n = 56**		
Intervention	100.0%	96.3%	0.21	(0.02, 2.04)	.18	0.33[c]	(0.02, 4.69)	.42
Control	94.4%	88.5%						
**Triple-negative**			**n = 87**			**n = 87**		
Intervention	91.7%	78.5%	0.75	(0.35, 1.64)	.48	0.73[c]	(0.30, 1.78)	.48
Control	84.4%	67.2%						

Adj. = adjusted, ECOG = Eastern Cooperative Oncology Group; HR = hazard ratio, NA = not applicable due to no event in the control group, Unadj. = unadjusted

© 2026 Karsten MM et al. *JAMA Oncology*.

[a]The model was adjusted for age, type of metastases, ECOG at baseline, subtype, metastatic presentation, and time since metastatic diagnosis.
[b]The model was adjusted for age, ECOG at baseline, subtype, metastatic presentation, and time since metastatic diagnosis. In the visceral subgroup, subtype was excluded because of the small sample size.
[c]The model was adjusted for age, type of metastases, ECOG at baseline, metastatic presentation, and time since metastatic diagnosis. In the HR-/HER2+, only age was included due to the small sample size.
The hazard ratios (HR) were estimated using a Cox proportional hazards model with a study center as a random shared frailty term.
The Interactions were explored (P value: group*metastasis = 0.49, group*metastatic presentation =0.79, and group*subtype =0.14).

eTable 10. Hospitalizations and emergency room visits

	Number of patients	Number of visits	Person-months	IR (95% CI) per 100 person-months	Crude IRR (95% CI) (n = 235)	Adjusted IRR (95% CI) [a] (n = 233)
**Hospitalizations**						
**6-month**						
Control group	117	107	594	18.0 (14.8 – 21.8)	1.00 (Reference)	1.00 (Reference)
Intervention	118	105	681	15.4 (12.6 – 18.7)	0.65 (0.42 – 1.01)	0.60 (0.40 – 0.92)
**12-month**						
Control group	117	176	1034	17.0 (14.6 – 19.7)	1.00 (Reference)	1.00 (Reference)
Intervention	118	165	1223	13.5 (11.5 – 15.7)	0.53 (0.34 – 0.85)	0.52 (0.45 – 0.78)
**Emergency room visits**						
**6-month**						
Control group	117	216	594	36.4 (31.7 – 41.6)	1.00 (Reference)	1.00 (Reference)
Intervention	118	216	681	31.7 (27.6 – 36.2)	0.88 (0.72 – 1.07)	0.87 (0.71 -1.06)
**12-month**						
Control group	117	406	1034	39.3 (35.6 – 43.3)	1.00 (Reference)	1.00 (Reference)
Intervention	118	378	1223	30.9 (27.9 – 34.2)	0.74 (0.61 – 0.90)	0.76 (0.63 – 0.92)

[a] The IRRs were estimated using multilevel mixed-effects negative binomial models with random intercept for study center and adjusted for age, type of metastases, ECOG at baseline, subtype, metastatic presentation, and time since metastatic diagnosis. CI, confidence interval; IR, incidence rate; IRR, incidence rate ratio.

© 2026 Karsten MM et al. *JAMA Oncology*.

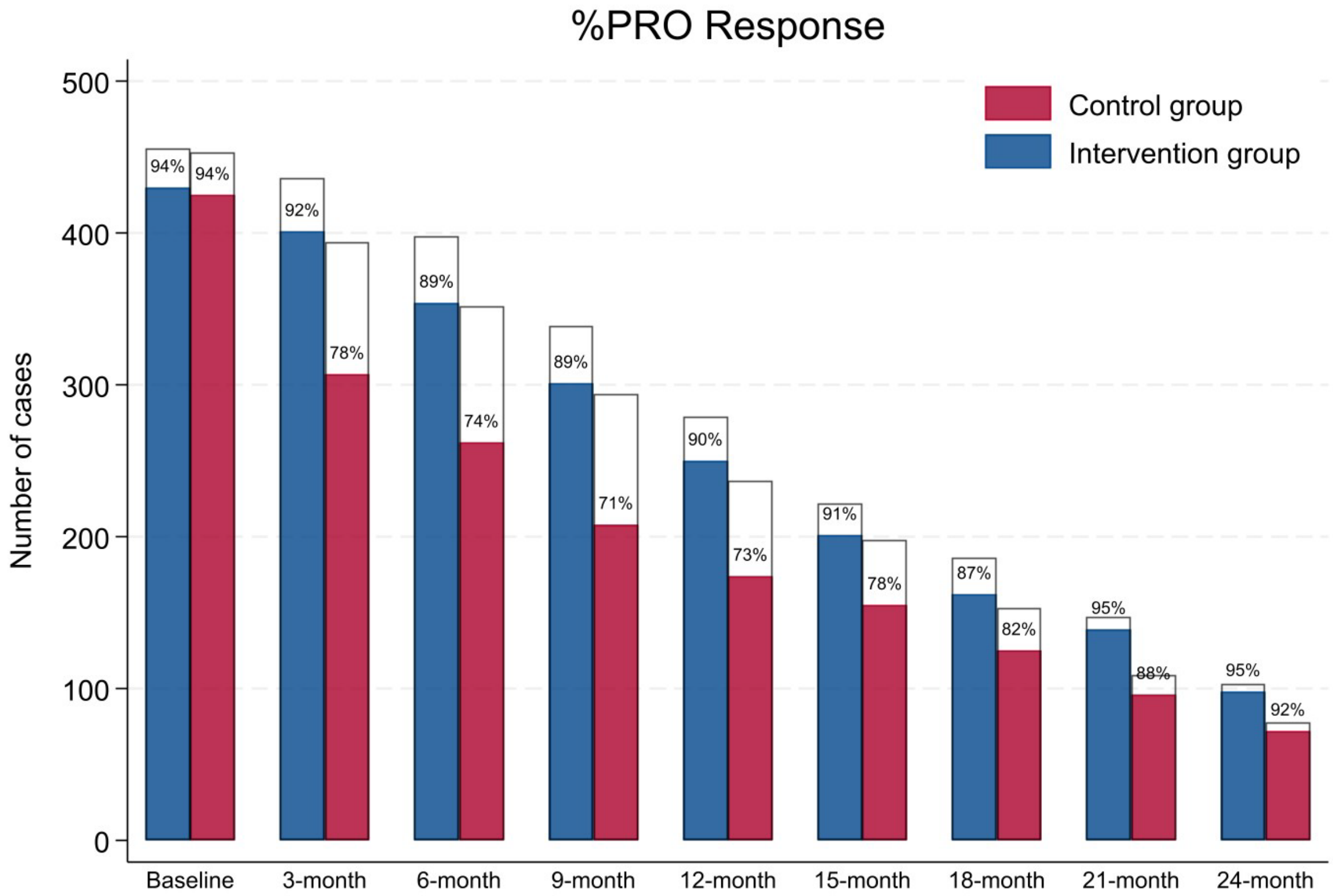

eFigure 1. The bar graph represents the PRO questionnaire return rate at each follow-up time point.
The y-axis shows the number of patients at each study visit, while the x-axis represents the study visits. The total height of each bar corresponds to the total number of patients at a given study visit, and the filled portion of the bar indicates the number of patients who returned the PRO questionnaires.

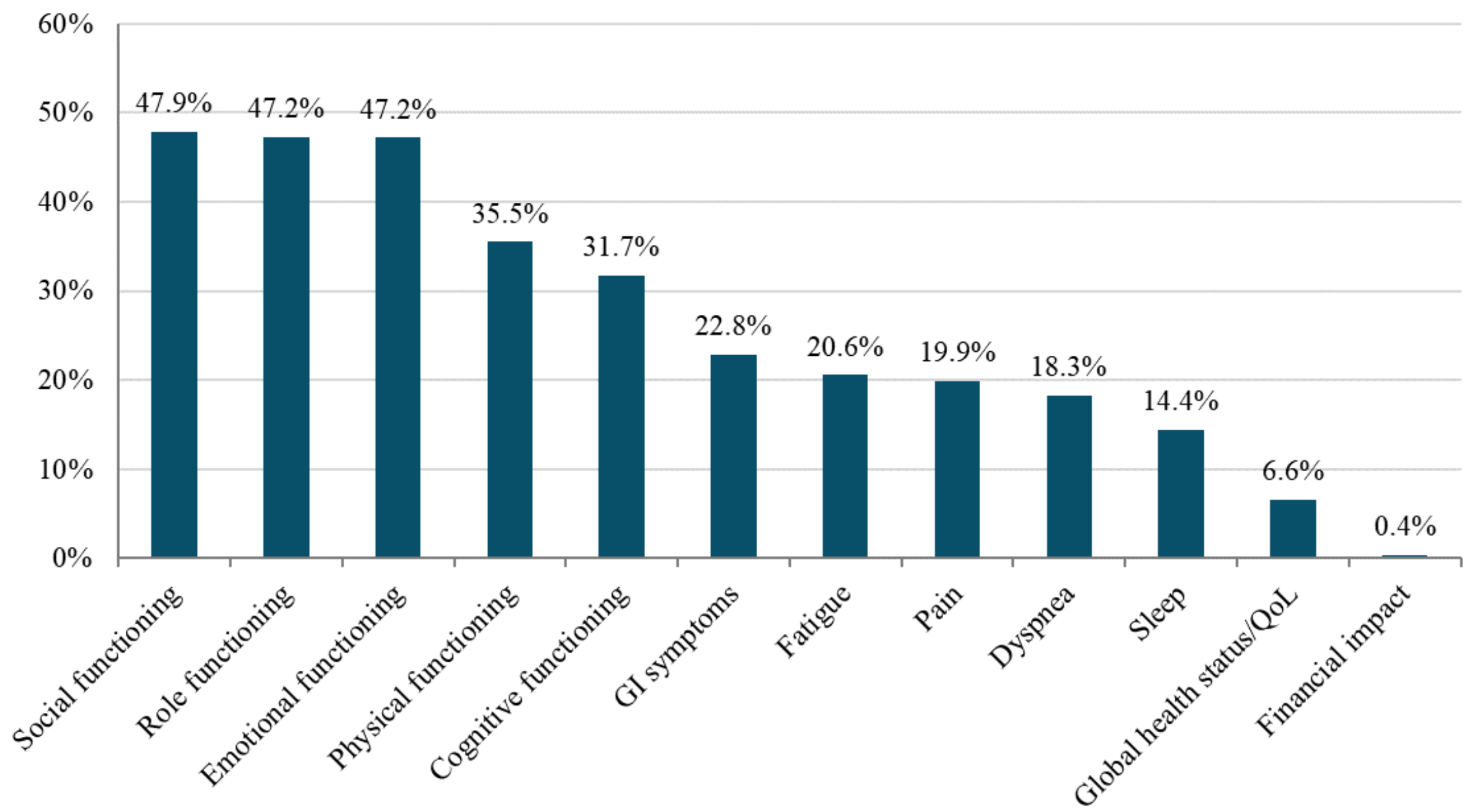

eFigure 2. Frequency of PRO domains triggering alerts (number of alerts = 3,019).

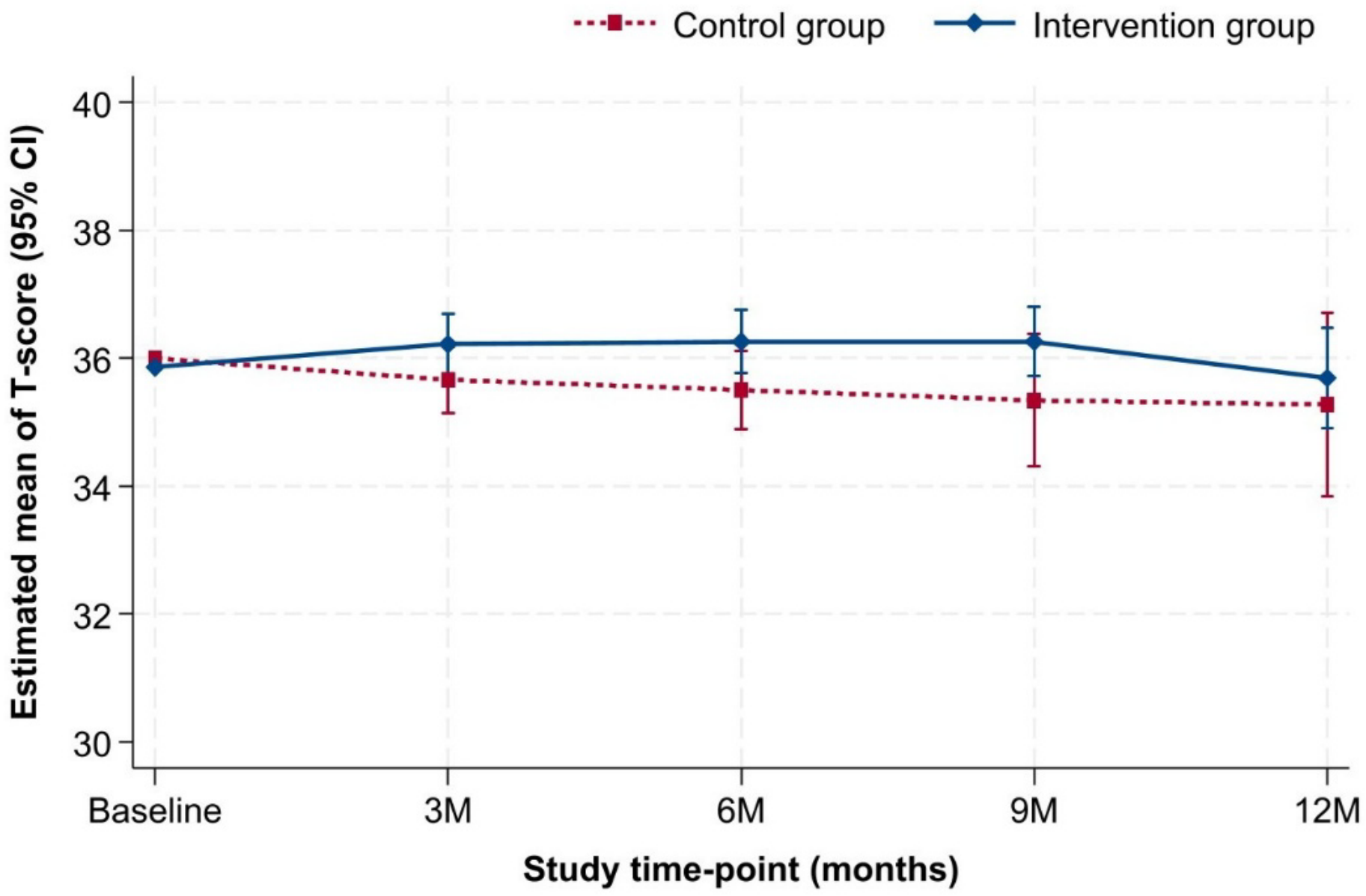

Study group	Baseline	Estimated mean T-score (95% CI)			
		3-month	6-month	9-month	12-month
Intervention	35.9 (SD 3.5) [n = 456]	36.2 (35.8 to 36.7) [n = 449]	36.3 (35.8 to 36.8) [n = 435]	36.3 (35.7 to 36.8) [n = 415]	35.7 (34.9 to 36.5) [n = 406]
Control	36.0 (SD 4.2) [n = 453]	35.7 (35.1 to 36.2) [n = 439]	35.5 (34.9 to 36.1) [n = 420]	35.3 (34.3 to 36.4) [n = 408]	35.3 (33.8 to 36.7) [n = 396]
Mean difference (IG - CG)	-0.2 (-0.6 to 0.3)	0.6 (-0.03 to 1.1)	0.8 (0.02 to 1.5)	0.9 (-0.1 to 1.9)	0.4 (-1.0 to 1.8)
P value	.44	.07	.04	.09	.90
		Estimated mean change in T-score from baseline (95% CI)			
Intervention	-	0.3 (-0.1 to 0.6)	0.3 (-0.1 to 0.7)	0.3 (-0.1 to 0.8)	-0.2 (-0.8 to 0.4)
Control	-	-0.4 (-0.8 to -0.005)	-0.5 (-1.0 to -0.1)	-0.7 (-1.5 to 0.1)	-0.7 (-1.8 to 0.3)
Mean difference (IG – CG)	-	0.7 (0.1 to 1.2)	0.8 (0.2 to 1.5)	1.0 (0.2 to 1.9)	0.5 (-0.6 to 1.6)

eFigure 3. Health-related quality of life (HRQoL) T-scores over 12 months

A higher T-score represents better HRQoL. CG, control group; CI, confidence interval; IG, intervention group; SD, standard deviation.

© 2026 Karsten MM et al. *JAMA Oncology*.

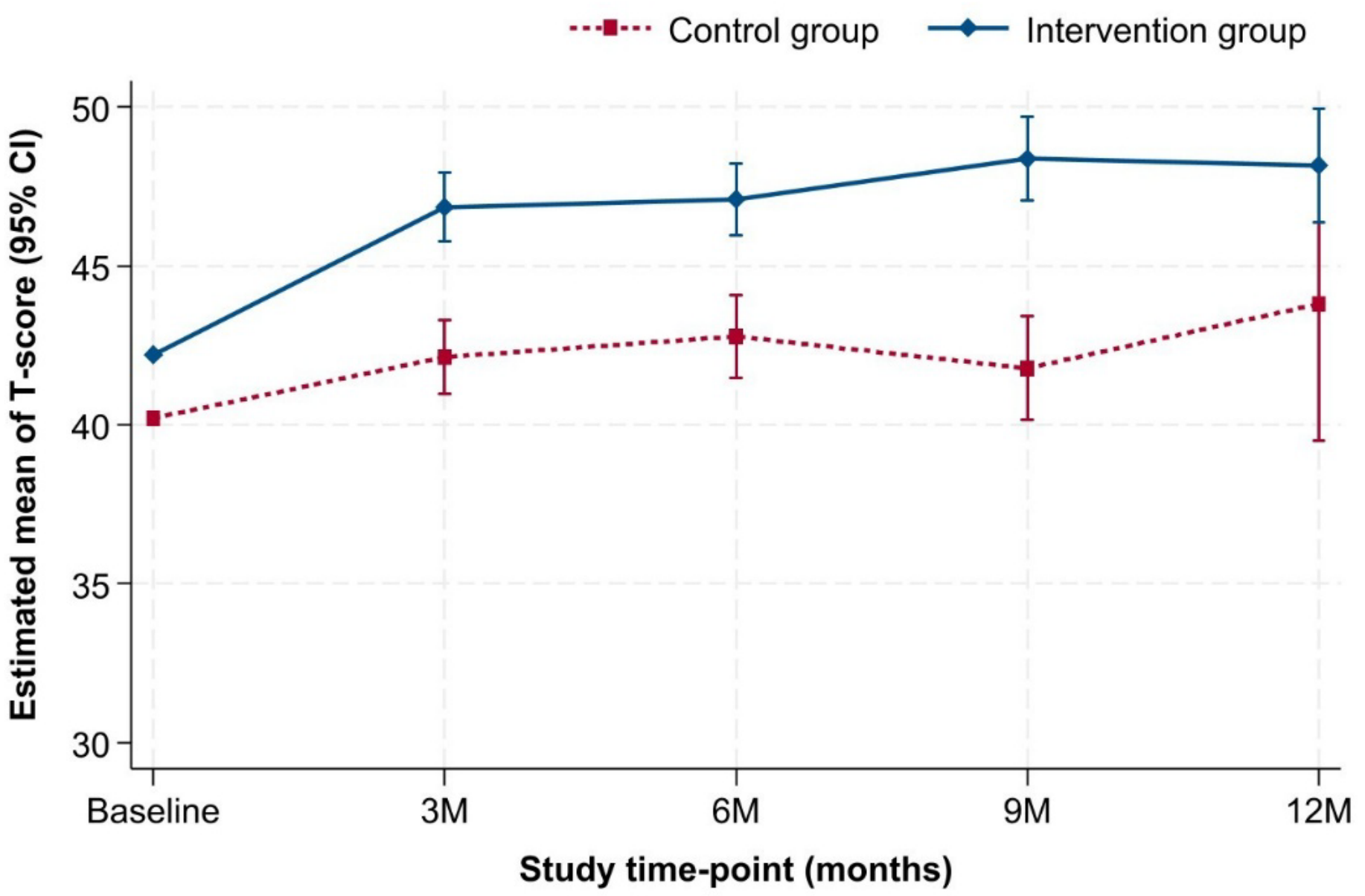

Study group	Baseline	Estimated mean T-score (95% CI)			
		**3-month**	**6-month**	**9-month**	**12-month**
Intervention	42.2 (SD 9.0)<br>[n = 456]	46.9 (45.8 to 47.9)<br>[n = 449]	47.1 (46.0 to 48.2)<br>[n = 435]	48.4 (47.1 to 49.7)<br>[n = 415]	48.2 (46.4 to 50.0)<br>[n = 406]
Control	40.2 (SD 8.4)<br>[n = 453]	42.1 (41.0 to 43.3)<br>[n = 439]	42.8 (41.5 to 44.1)<br>[n = 420]	41.8 (40.1 to 43.4)<br>[n = 408]	43.8 (39.5 to 48.1)<br>[n = 396]
Mean difference (IG - CG)	2.1 (1.0 to 3.2)	4.7 (3.4 to 6.1)	4.3 (2.8 to 5.8)	6.6 (4.7 to 8.5)	4.4 (0.2 to 8.5)
P value	< .001	< .001	< .001	< .001	.032
		**Estimated mean change in T-score from baseline (95% CI)**			
Intervention	-	5.0 (4.2 to 5.9)	5.3 (4.4 to 6.2)	6.6 (5.5 to 7.7)	6.4 (5.0 to 7.8)
Control	-	1.3 (0.4 to 2.2)	2.0 (1.0 to 3.0)	1.0 (-0.2 to 2.3)	3.1 (0.02 to 6.1)
Mean difference (IG – CG)	-	3.7 (2.5 to 4.9)	3.3 (2.0 to 4.6)	5.6 (4.0 to 7.2)	3.3 (0.2 to 6.5)

eFigure 4. Emotional functioning T-scores over 12 months

A higher T-score represents better emotional functioning. CG, control group; CI, confidence interval; IG, intervention group; SD, standard deviation.

© 2026 Karsten MM et al. *JAMA Oncology*.

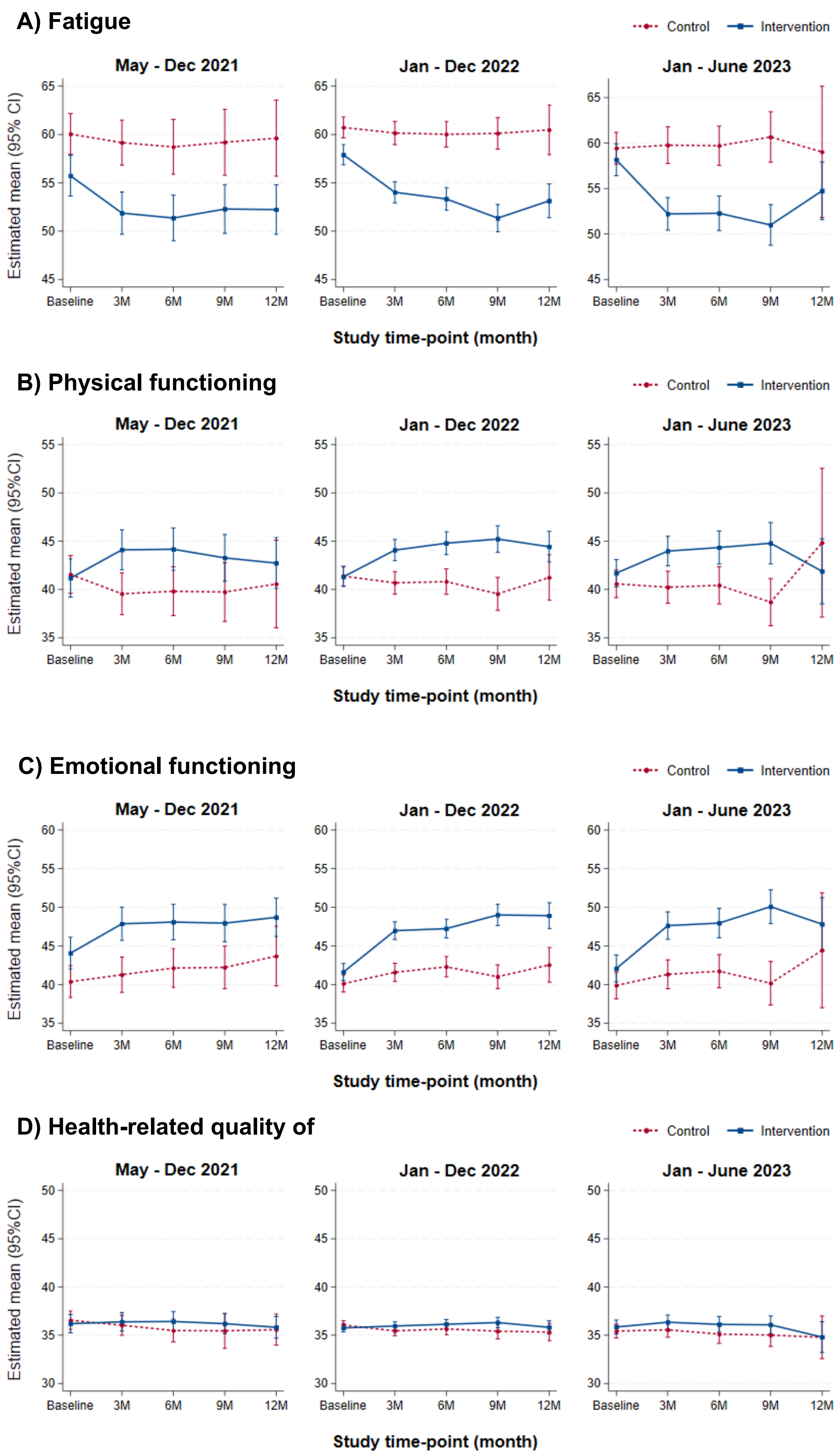

eFigure 5. Estimated marginal mean PRO T-scores over 12 months by study group, stratified by enrollment period.
(A) Fatigue; (B) Physical functioning; (C) Emotional functioning; (D) Health-related quality of life.
Error bars represent 95% confidence intervals (CI). Higher T-scores indicate better functioning for functional scales and greater symptom burden for symptom scales.

© 2026 Karsten MM et al. *JAMA Oncology*.

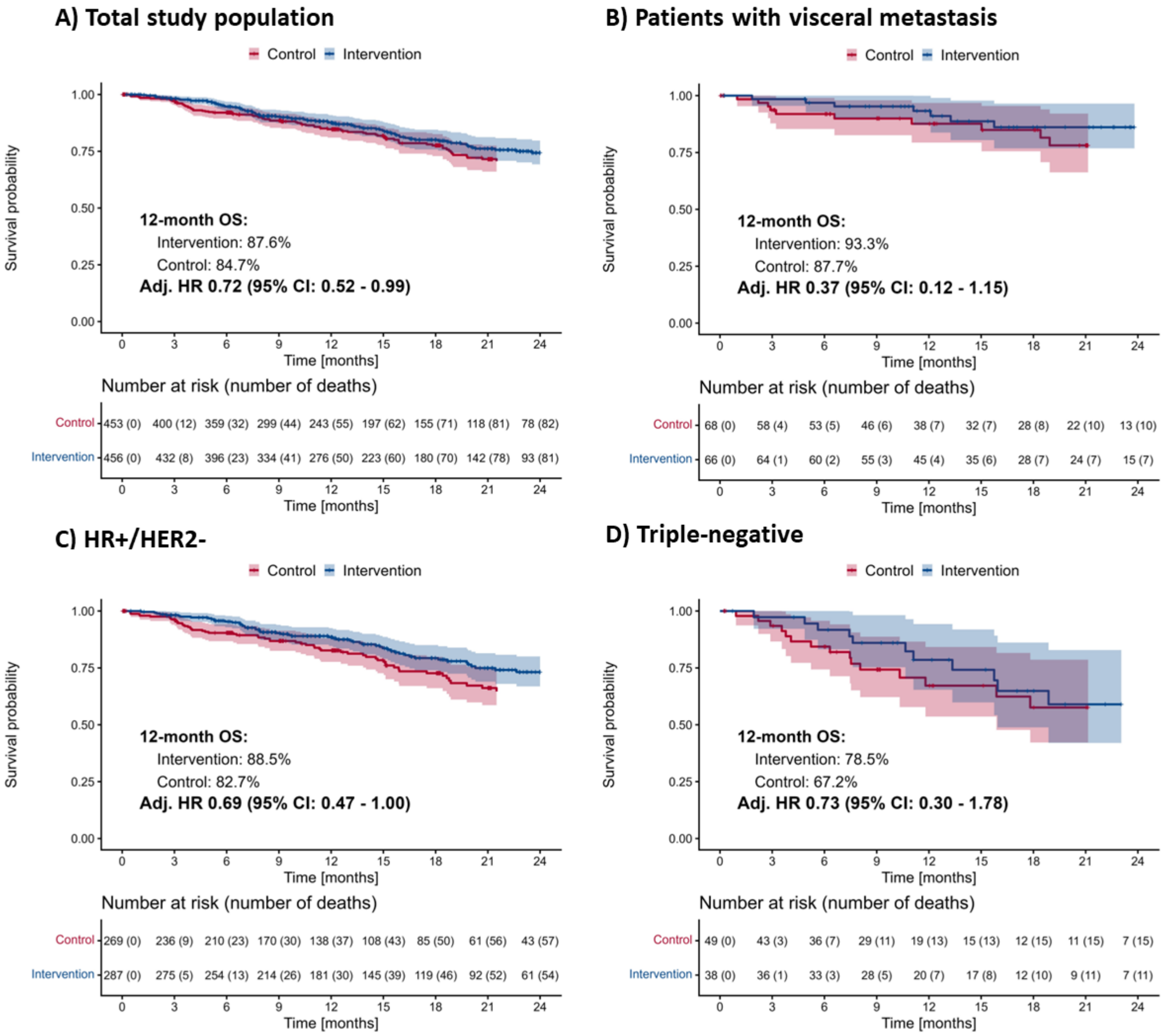

eFigure 6. Kaplan-Meier Curves of overall survival

The hazard ratios were estimated using a Cox proportional hazards model with a random shared frailty term for study centers.
Adj, adjusted; HR, hazard ratio; OS, overall survival

© 2026 Karsten MM et al. *JAMA Oncology*.

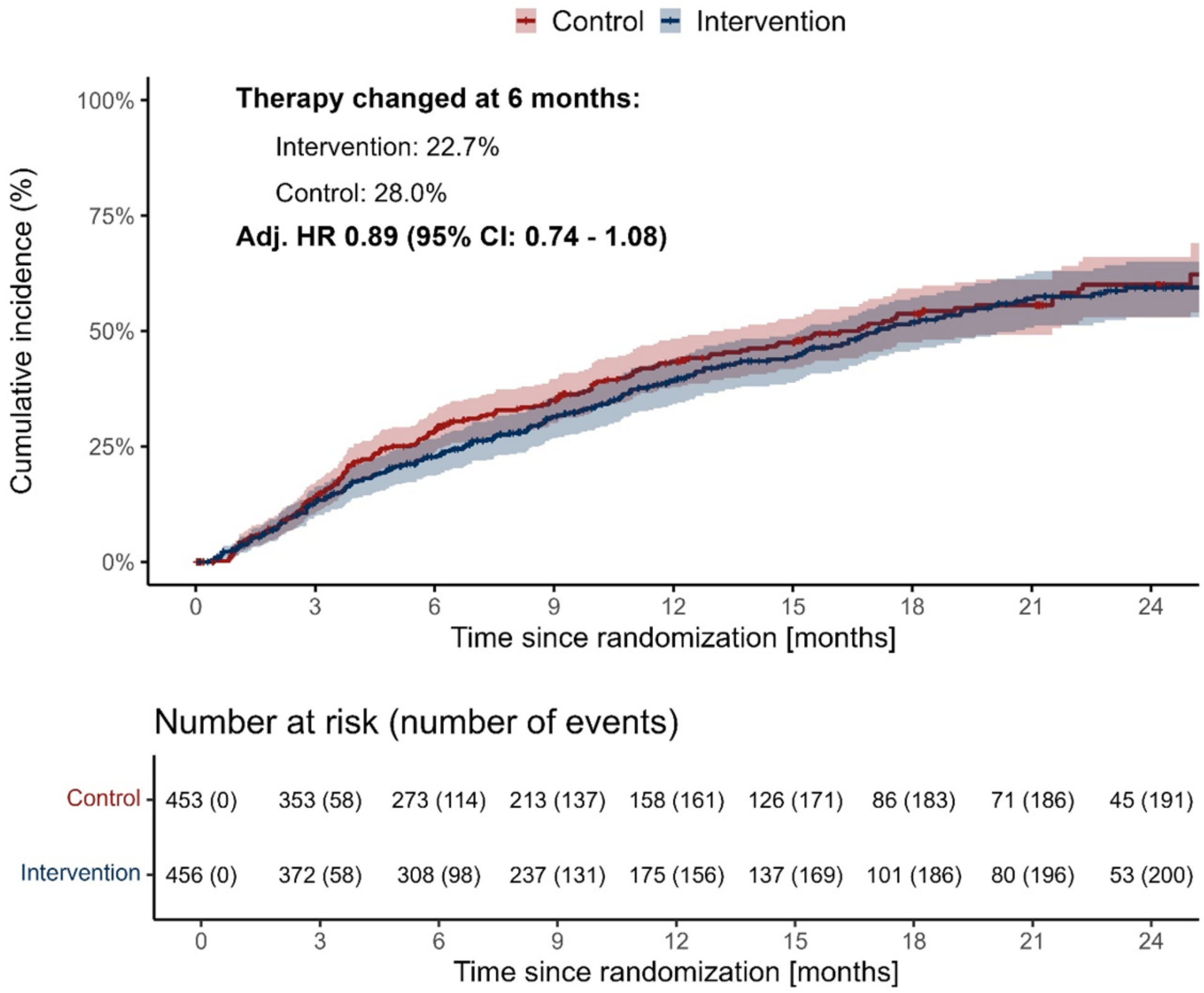

eFigure 7. Time to first change of systemic therapy

Adj. = adjusted; HR = hazard ratio.
The hazard ratios (HR) were estimated using a Cox proportional hazards model with study center as a random shared frailty term. The model was adjusted for age, type of metastases, metastatic presentation, time since metastatic diagnosis, and systemic therapy at baseline (including endocrine, chemotherapy, anti-Her2, another immunotherapy, and targeted therapy).

© 2026 Karsten MM et al. *JAMA Oncology*.

References

1. Gebert P, Karsten MM, Hage AM, Dordevic AD, Grittner U. Statistical analysis plan for the PRO B study: open-label, superiority randomised controlled trial of alarm-based patient-reported outcome monitoring in patients with metastatic breast cancer. *Trials* 2024; **25**(1): 171.
2. Hage AM, Gebert P, Kuhn F, Pross T, Grittner U, Karsten MM. Examining the Feasibility of an Application-Based Patient-Reported Outcome Monitoring for Breast Cancer Patients: A Pretest for the PRO B Study. *Int J Environ Res Public Health* 2022; **19**(14).
3. Karsten MM, Kuhn F, Pross T, et al. PRO B: evaluating the effect of an alarm-based patient-reported outcome monitoring compared with usual care in metastatic breast cancer patients-study protocol for a randomised controlled trial. *Trials* 2021; **22**(1): 666.
4. Coens C, Pe M, Dueck AC, et al. International standards for the analysis of quality-of-life and patient-reported outcome endpoints in cancer randomised controlled trials: recommendations of the SISAQOL Consortium. *Lancet Oncol* 2020; **21**(2): e83–e96.

© 2026 Karsten MM et al. *JAMA Oncology*.
