# Supplementary material for: Electronic Patient-Reported Outcome Monitoring With Alert-Based Interventions in Metastatic Breast Cancer: A Randomized Clinical Trial
Source: JAMA Oncol. 2026 Aug 6;12(10):1069–78. doi: 10.1001/jamaoncol.2026.2661 (PMC13448833; doi:10.1001/jamaoncol.2026.2661)
Supplement: Supplementary file 3 [file jamaoncol-e262661-s003.pdf]

Data Sharing Statement

Karsten. Electronic Patient-Reported Outcome Monitoring With Alert-Based Interventions in Metastatic Breast Cancer. *JAMA Oncol*. Published August 06, 2026.
doi:10.1001/jamaoncol.2026.2661

Data
Additional Information: German Clinical Trials Register: DRKS00024015. Registered on 15 February 2021, https://drks.de/search/de/trial/DRKS00024015
Data available: Yes
Data types: Deidentified participant data
How to access data: Deidentified participant data will be shared upon reasonable request to the corresponding author, subject to the study's data privacy agreement
When available: With publication

Supporting Documents
Document types: None

Additional Information
Who can access the data: researchers whose proposed use of the data has been approved
Types of analyses: research only
Mechanisms of data availability: after approval of proposal
Any additional restrictions: It is all subject to the privacy restrictions mandated by the German data protection and signed study documents
